# Regulatory function and mechanism research for m6A modification WTAP via SUCLG2-AS1- miR-17-5p-JAK1 axis in AML

**DOI:** 10.1186/s12885-023-11687-4

**Published:** 2024-01-17

**Authors:** Miaomiao Liu, Bingxin Yu, Yong Tian, Fan Li

**Affiliations:** 1 Department of Pathogenobiology, The Key Laboratory of Zoonosis, Chinese Ministry of Education, College of Basic Medicine, Jilin University, No.126 Xinmin Street, Changchun, Jilin 130021 P.R. China; 2 Department of Ultrasonography, The Third Hospital of Jilin University, Changchun, Jilin 130033 P.R. China; 3 Department of Human Anatomy, Chinese Ministry of Education, College of Basic Medicine, Jilin University, Changchun, Jilin 130021 P.R. China; 4 The Key Laboratory for Bionics Engineering, Ministry of Education, Jilin University, Changchun, 130021 P.R. China; 5 Engineering Research Center for Medical Biomaterials of Jilin Province, Jilin University, Changchun, 130021 P.R. China; 6 Key Laboratory for Health Biomedical Materials of Jilin Province, Jilin University, Changchun, 130021 P.R. China; 7 State Key Laboratory of Pathogenesis, Prevention and Treatment of High Incidence Diseases in Central Asia, Urumqi, Xinjiang 830017 P.R. China

**Keywords:** AML, lncRNA SUCLG2-AS1, miR-17-5p, WTAP, JAK1

## Abstract

Acute myeloid leukemia (AML), characterized by the abnormal accumulation of immature marrow cells in the bone marrow, is a malignant tumor of the blood system. Currently, the pathogenesis of AML is not yet clear. Therefore, this study aims to explore the mechanisms underlying the development of AML. Firstly, we identified a competing endogenous RNA (ceRNA) SUCLG2-AS1-miR-17-5p-JAK1 axis through bioinformatics analysis. Overexpression of SUCLG2-AS1 inhibits proliferation, migration and invasion and promotes apoptosis of AML cells. Secondly, luciferase reporter assay and RIP assay validated that SUCLG2-AS1 functioned as ceRNA for sponging miR-17-5p, further leading to JAK1 underexpression. Additionally, the results of MeRIP-qPCR and m6A RNA methylation quantification indicted that SUCLG2-AS1(lncRNA) had higher levels of m6A RNA methylation compared with controls, and SUCLG2-AS1 is regulated by m6A modification of WTAP in AML cells. WTAP, one of the main regulatory components of m6A methyltransferase complexes, proved to be highly expressed in AML and elevated WTAP is associated with poor prognosis of AML patients. Taken together, the WTAP-SUCLG2-AS1-miR-17-5p-JAK1 axis played essential roles in the process of AML development, which provided a novel therapeutic target for AML.

**Supplementary Information:**

The online version contains supplementary material available at 10.1186/s12885-023-11687-4.

## Introduction

Acute myeloid leukemia (AML) is a heterogeneous clonal tumor caused by cumulative genetic aberration [1], characterized by increased proliferation and blocked differentiation of leukemia cells [2]. Current treatments for AML include chemotherapy and hematopoietic stem cell transplantation [3]. Although treatment has improved for some specific subtypes of AML, it is still not ideal for the vast majority of AML patients. Therefore, finding novel treatments that can more effectively target AML remains critical. The occurrence and development of AML is related to many factors, including the genetic abnormalities [4], and the treatment responses and prognosis of AML vary greatly [5]. In recent years, with the development of molecular biology, especially genomics technology and second-generation sequencing technology [6], our understanding of the molecular heterogeneity of genes has been constantly improved. Transcriptome datas are mainly genes expression datas at the RNA level [7], which are mainly used to analyse genes expression and regulation rules at the transcription level. Among them, transcription factors, miRNA, lncRNA and circRNA are all factors influencing genes transcription regulation [8–10], and they can participate in many important cellular biological processes. These include cell proliferation, cell, differentiation, angiogenesis, apoptosis and immune responses [11]. These transcriptional regulatory molecules play an extremely important role in the development of AML and are an indispensable part of the molecular mechanism of AML.

N6-methyladenosine (m6A) methylation is one of the most common RNA modifications [12], and recent studies have demonstrated that abnormal expression of m6A regulators can influence the development of cancer [13]. M6A modifications regulates cell proliferation and metastasis [14], stem cell differentiation and homeostasis in cancer by influencing cell biological functions, as well as the cleavage, transport, stability and degradation of non-coding RNAs themselves. M6A methyltransferase regulates the function of lncRNAs [15]. For example, XIST (lncRNA, the transcriptional silencing of an X chromosome in female mammals requires XIST recruitment specific proteins to regulate gene silencing) [16] is a target of RBM15/15B-mediated m6A methylation modification. WTAP (m6A methyltransferase) is a protein associated with XIST, and RBM15/15B can, also bind METTL3 through WTAP protein to form m6A methylase complex and act on XIST to affect its function. Some studies suggest that the expression of WTAP in the AML group was significantly higher than that in the healthy control group [17], and the difference was showed statistically significant (*P* < 0.01). A large amount of evidence indicates that both m6A and lncRNAs play certain roles in the occurrence and development of tumors [18], but how WTAP regulates lncRNAs in AML has not been reported.

Long non-coding RNAs (lncRNAs) are a class of RNA molecules with transcripts over 200-100000nt long that do not encode proteins [19]. LncRNAs are involved in a variety of biological regulation and play an important role in life activities [20]. Recent studies have shown that some specific lncRNAs can modify the epigenetic states of DNA/RNA and histones by recruiting chromatin modification complexes. For example, HOXC gene cluster transcription of lncRNA HOTAIR [21] will collect chromatin modification complex PRC2 and locate it to the HOXD gene cluster site, change the chromatin modification state in this region, and then inhibit HOXD gene expression [22]. Forthermore, lncRNA SUCLG2-AS1 can be used as a prognostic predictor of clear cell renal cell carcinoma [23], with shorter survival time and progression-free survival in patients with low expression. LncRNAs are also directly involved in the post-transcriptional regulation of mRNA [24], including variable shearing, RNA editing, protein translation and transport. LncRNAs also affect the expression of target genes by controlling microRNAs (miRNAs) expression [25]. In some tumor cells and specific tissues, some lncRNAs carry “seed sequences” of certain miRNAs and bind miRNAs like sponges [26], thus preventing miRNAs from binding to their target mRNAs.

**Fig. 1:**
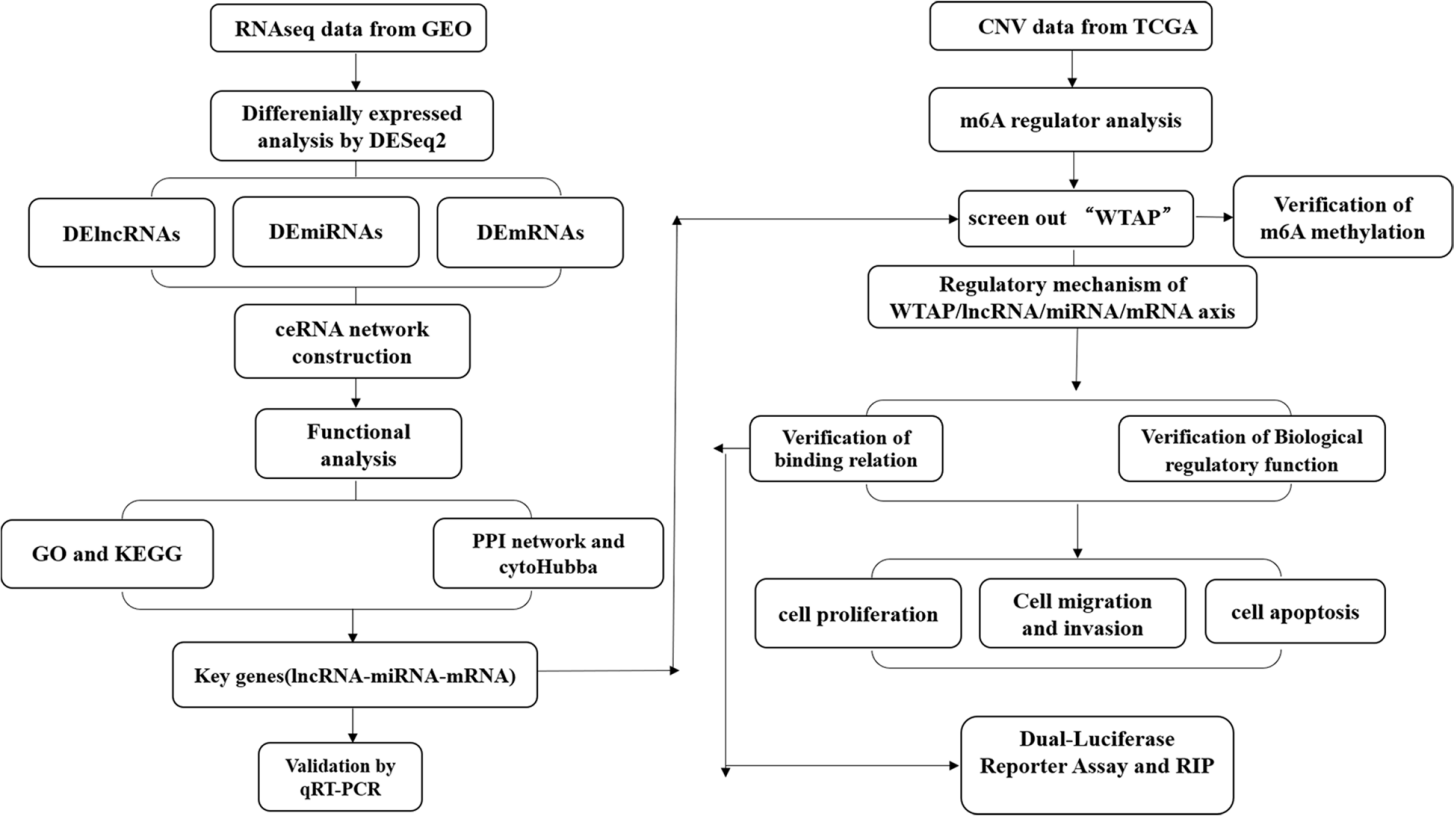
Flowchart of this study

In this study, we find WTAP-SUCLG2-AS1-miR-17-5p-JAK1 axis that has an important significance to improve the therapeutic effect of AML. The flow chart of this study is shown in Fig. 1.

## Materials and methods

### Database download

The RNA-seq datasets of the miRNA expression (dataset GSE142699), mRNA expression, and lncRNA expression (dataset GSE96535) were downloaded from the National Center of Biotechnology Information Gene Expression Omnibus (GEO, http://www.ncbi.nlm.nih.gov/geo/). Somatic mutation information was obtained from the TCGA database (http://cancergenome.nih.gov/), and datasets were obtained for copy number variant (CNV) assays [27]. The datasets in this study was in Supplementary Table S1.

### GEO dataset processing

The original expression matrix was normalized and processed by R [28]. The limma package was used to screen out differentially expressed genes [29]. The GEO query function of the Bioconductor package was employed to download the RNA-seq expression value datasets. We used the “limma” package containing a linear model and empirical Bayes statistics to filter out nonspecific expression data. For mRNAs, lncRNAs and miRNAs, *p* < 0.05 and |log2 FC|≥1 were considered as the cutoff criteria for differential expression and mRNAs, lncRNAs and miRNAs that met these criteria were clustered following hierarchical clustering analysis with R package “ggplot2” [30].

### Prediction of lncRNA-miRNA and miRNA-mRNA interactions

The RNA expression profile data downloaded from the GEO were analysed with the “DESeq2” R package. By setting the |log2 FC|> 1 with an adjusted false discovery rate (FDR) of *P* < 0.05, the differentially expressed lncRNAs (DElncRNAs), mRNAs (DEmRNAs), and miRNAs (DEmiRNAs) were screened for subsequent analysis.

DElncRNAs, DEmiRNAs, and DEmRNAs were used to construct a regulatory network. The experimental module DIANA-LncBase Version 2 (http://www.microRNA.gr/LncBase) was used for the lncRNA-miRNA predicted interactions. TargetScan (http://www.targetscan.org), miRDB (http://www.mirdb.org/), and DIANA-TarBase Version 8 (http://www.microrna.gr/tarbase) were used for obtaining predicted interactions between DEmiRNAs and DEmRNAs. The predicted interactions between DElncRNAs, DEmiRNAs, and DEmRNAs were used to construct a lncRNA-miRNA-mRNA ceRNA network with Cytoscape Version 3.7.2 [31], showing how lncRNAs can affect the function of miRNAs and act as miRNA sponges to regulate mRNA expression.

### Gene Ontology and the Kyoto Encyclopedia of Genes and Genomes pathway

#### Enrichment analysis

The biological processes in Gene Ontology (GO) and the Kyoto Encyclopedia of Genes and Genomes (KEGG) pathways (*P* < 0.05) were used for the biological functional analysis of DEmRNAs in the ceRNA network. The significant enrichment results were visualized using the ggplot2 R package.

#### Integration of protein-protein interaction (PPI) network and module analysis

To further investigate the function of DEmRNAs in the ceRNA network at the protein level, the Search Tool for the Retrieval of Interacting Genes (STRING) database was used to construct PPI network [32]. DEmRNAs were mapped to STRING, and the median confidence score was 0.8, the protein interactions were evaluated. Cytoscape version 3.7.2 was used to construct the PPI network, and the plug-in “cytoHubba” was used to analyse the sub-network.

### CNV dataset processing

R package “limma” was utilized to perform differential expression analysis of the 23 m6A regulators. The results were assayed using R 3.7.2 and R Bioconductor software. The R package from RCircos was used to map CNVs in the 23 m6A modulators. The waterfall function of the maftools package was employed to present the mutations in patients. Kaplan–Meier curves and log-rank tests were conducted for survival analysis. In all cases, *p* < 0.05 was considered statistically significant.

### Cell culture

The cells used in this study include normal human bone marrow stromal cell line HS-5, and the AML cell lines THP-1 and HL-60. The source of all cell lines used in the study: THP-1 and HS-5 cell lines were frozen stored in pathogenic biology laboratory of Basic Medical College of Jilin University, and HL-60 cell lines was donated by immunology laboratory of Basic Medical College of Jilin University. The cells were cultured in RPMI 1640 (containing 10% fetal bovine serum, 100 g/mL streptomycin and 100 u/mL penicillin) in an incubator at 37 °C and 5% CO2.

### RNA extraction and qRT-PCR

With regard to miRNA, according to miRcute miRNA isolation kit from TIANGEN, extracting total RNAs from 1 × 10^6^ cells based on the provided directions, cDNA was then synthesized using the miRcute Plus miRNA First-Strand cDNA Kit, and a miRcute Plus miRNA qPCR Kit (SYBR Green) was employed to conduct real-time PCR. U6 small nuclear RNA was applied as the internal control [33]. The primer sequence of miR-17-5p was designed by Shenggong Company. For mRNA and lncRNA, a total RNA purification kit was used to extract total RNAs from cells, and NovoScript Plus All-in-one 1st Strand cDNA Synthesis SuperMix (gDNA Purge) was applied to obtain cDNA. FastStart Universal SYBR Green Master Mix (Rox) was employed to conduct real-time PCR. GAPDH was adopted as the internal control. The primer sequences are detailed below (Table 1).

**Table 1:** Real-time quantitative PCR primer sequences used in this study

Primer name	Forward (5′ to 3′)	Reverse (5′ to 3′)
*GAPDH*	AAGGTGAAGGTCGGAGTCAA	AATGAAGGGGTCATTGATGG
*SUCLG2-AS1*	AGGCTGATGACTTGACTGCAACTAAC	CTGGTAAAGTGGATGGCTTCTCTATGG
*JAK1*	GTCAGCATTAACAAGCAGGACAACAAG	CATCTACCAGGGACACAAAGGACAAG
*WTAP*	GCAACACAACCGAAGATGACTTTCC	CCTCCTCTGCCAGTTCTCTCCTC
*U6*	CTCGCTTCGGCAGCACA	AACGCTTCACGAATTTGCGT

### M6A RNA methylation quantification

The m6A RNA Methylation Quantification Kit (A&D Technology) was used to detect total m6A levels of the extracted RNA [34].

### Immunofluorescence (IF)

AML cells were fixed, permeabilized, and blocked, and then incubated with anti-WTAP antibody or goat anti-rabbit IgG (H + L) antibody. DAPI was used to stain nuclei. Cells were observed using a confocal microscopy (Nikon, Japan) [35].

### RNA immunoprecipitation (RIP) assays

Magna RIP RNA-Binding Protein Immunoprecipitation kit (Millipore) was used [36]. Total RNA was used as an input control. IgG was used as an isotype control. The cells were obtained to carry out RNA immunoprecipitation (RIP) experiments using a m6A antibody (Abcam) or Ago2 antibody (Abcam). RNAs were isolated for qRT-PCR assay.

### Gene silencing/overexpression

Small short hair RNA (shRNA) targeting WTAP (sh-WTAP-1, sh-WTAP-2) and its negative control (sh-NC), SUCLG2-AS1 overexpression plasmid (pcDNA3.1 SUCLG2-AS1) and its negative control (pcDNA3.1 vector). JAK1 overexpression plasmid (pcDNA3.1 JAK1) and its negative control (pcDNA3.1 vector). All plasmids including the miR-17-5p inhibitor and miR-17-5p mimics were purchased from PPL Biotechnology Company.

### Cell transfection

AML cells were inoculated into 6-well cell culture plates at a concentration of 5 × 10^5^ /mL per well. When the cells growth density reached more than 70%, plasmids were transfected using X-tremeGENE HP DNA transfection kit (Roche) according to the manufacturer’s instructions [37].

### CCK-8 assay

CCK-8 reagent (Meilunbio) was used to determine the proliferation of AML cells in each group [38]. The following steps were performed at 24 h, 48 h, 72 h and 96 h after cell transformation: The cells were washed twice with 1×PBS, and then the 10% CCK-8 medium was added to each well in the form of liquid exchange, and incubated for 30 min in the dark; The absorbance at 450 nm was measured with a microplate reader and recorded.

### EdU assay

AML cells were treated with 100 µL of EdU solution (20 µM, EdU-488 kit, Meilunbio) in 6-well plates for 2 h at 37 °C with 5% CO2 [39]. After washing, the cells were fixed 4% paraformaldehyde and then 1% Triton 100 for 45 min. Hoechst33342 solution was used to stain the nuclei. The images were collected by fluorescence microscope (Cytation5, BioTek). The cells were counted by ImageJ software, and the green EdU and blue Hoechst images at the same position were merged and analysed.

### Dual-luciferase reporter assay

Bioinformatics databases DIANA-lncbase Version 2, miRDB and Annolnc were used to predict the possible binding miRNAs and binding sites of SUCLG2-AS1 and DIANA-Tarbase Version 8, miRDB, Starbase and TargetScan were used to predict the mRNA and binding sites of miR-17-5p. In brief SUCLG2-AS1 (miR-17-5p)-WT, SUCLG2-AS1 (miR-17-5p)-MUT, JAK1-3UTR (miR-17-5p)-WT, JAK1-3UTR (miR-17-5p)-MUT, miR-17-5p mimic and inhibitor were used to cotransfected into 293T cells to measure luciferase activity [40].

### Transwell assay

Transwell assays were used to detect the migration and invasion of AML cells [41]. The AML cells were collected 48 h after transfection. Serum-free medium was used to resuspend cells. The cell concentration of each group was adjusted to 1 × 10^5^ cells/mL. 200 µl cell suspension was added to the upper transwell chamber. Then 600 µl complete medium containing 20% fetal bovine serum was added to the lower chamber. After 24 h of culture, cells invaded to the lower surface were fixed with 4% methanol for 15–30 min followed by staining with Giemsa working solution at room temperature for 30 min. Finally, the number of invaded cells was counted under a microscope (Nikon, Japan). For invasion detection, 40 µl of VitroGel 3D-RGD gel solution was used to precoat the upper of the membrane first. The other procedures were similar as above in the migration detection methods.

### Western blot analysis

A Column Tissue & Cell Protein Extration Kit was applied to extract the total proteins derived from AML cells, and a BCA Protein Assay Kit (Sigma) was applied to detect the protein concentration. According to the PAGE Gel Quick Preparation Kit, proteins were fractionated by 10% sodium dodecyl sulfate/polyacrylamide gel electrophoresis (SDS/PAGE). Then we placed the protein into a polyvinylidene fluoride (PVDF) membrane after separation. Primary antibodies were incubated with β-actin, E-cadherin, N-cadherin, JAK1, and WTAP at 4 °C overnight, and all antibodies were purchased from Abcam. Subsequently, blotted membranes were incubated for 1 h with HRP-conjugated secondary antibody at ambient temperature. ECL Substrates (Millipore) were used to visualize the signals.

### EMT (epithelial-mesenchymal transition) assay

In the progression of tumor malignancy, epithelial-mesenchymal transition (EMT) plays an extremely important role, not only assisting tumor cells in completing metastasis and invasion, but also enabling tumor cells to escape cell apoptosis induced by certain factors. It is known that E-cadherin expression is decreased and N-cadherin expression is increased as a hallmark change in the EMT process [42]. To verify whether the migration and invasion ability of AML were related to the EMT process, a Western blot analysis was performed to detect the changes in the protein expression levels of E-cadherin and N-cadherin.

### Flow cytometry

The cells were collected by centrifugation at 160 × g at 4˚C for 5 min and washed twice with cold PBS after transfection for 48 h. Then 5 µl FITC Annexin V and 5 µl PI Binding Buffer were added to 100 µl cell suspension. A flow cytometer (CytoFLEX, Beckman) was employed to obtain fluorescence signals and ascertain the apoptosis rate. FlowJo software was used to further analyse the detection results.

### Statistical analysis

Graphpad Prism 8.0 software was used for statistical analysis and plotting of the experimental data obtained. Each group of experiments was repeated for 3 times, and the data was expressed as mean ± SD. At the same time, Student’s T-test was used for statistical analysis between the two groups. One-way ANOVA was used for comparative analysis between multiple groups, and *P* < 0.05 was considered statistically significant.

## Results

### Differentially expressed lncRNAs, miRNAs, and mRNAs

**Fig. 2:**
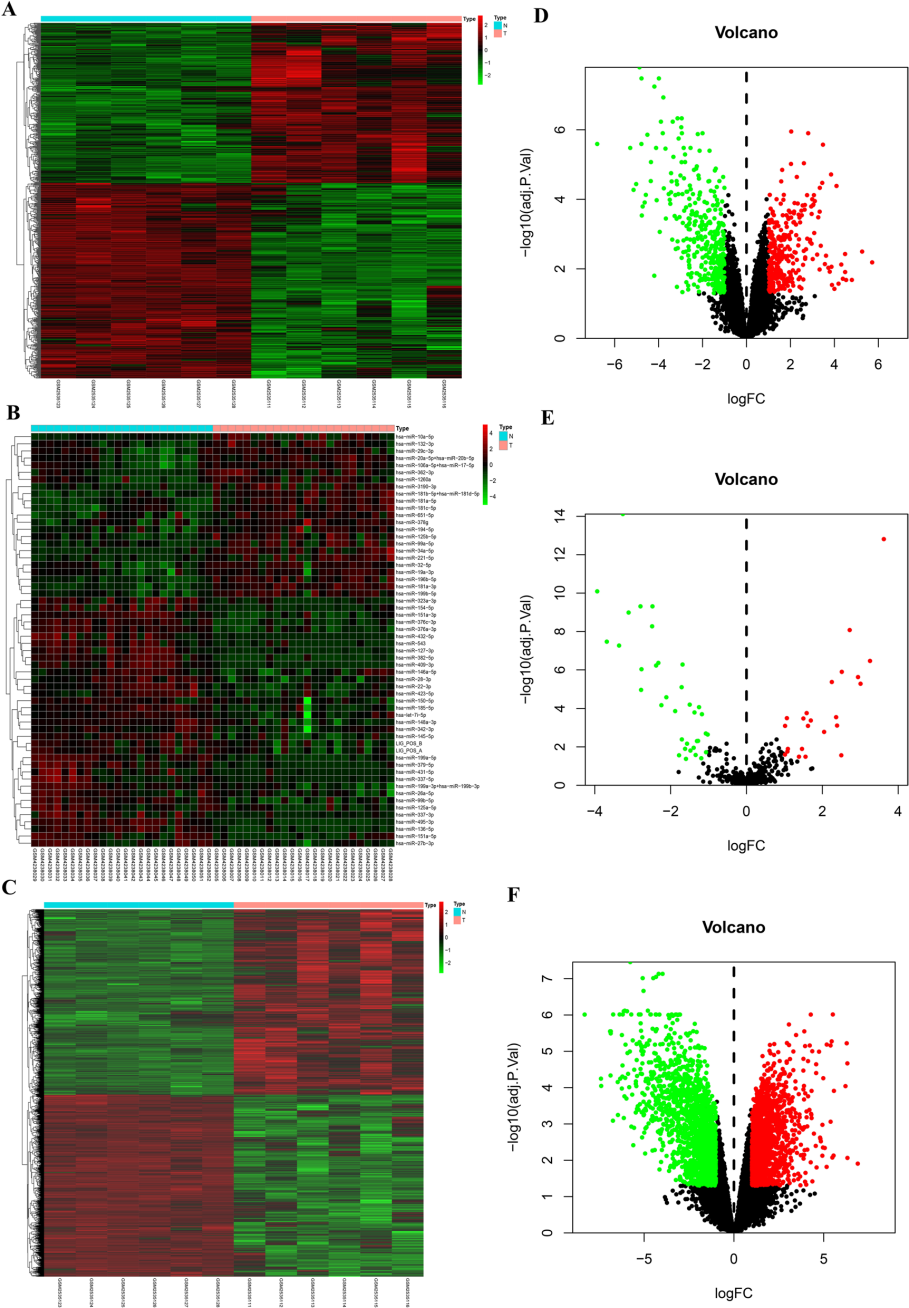
Hierarchical heatmaps and volcano plots presenting differentially expressed lncRNAs, miRNAs, and mRNAs. Left panels, heatmaps for all differentially expressed **A** lncRNAs, **B** miRNAs, and **C** mRNAs in AML; Right panels, volcano plots showing **D** lncRNAs, **E** miRNAs, and **F** mRNAs with fold change ≥ 1 (*P* < 0.05). Green, downregulated; red, upregulated; black, not differentially expressed. lncRNA: long noncoding RNA; miRNA: microRNA

A total of 664 DElncRNAs, 58 DEmiRNAs, and 3700 DEmRNAs were identified in AML patients at |log2 FC| > 1 and an adjusted FDR of *P* < 0.001. The differential expression of lncRNAs, miRNAs, and mRNAs among the samples is shown by hierarchical clustering (Fig. 2). Figure 2A, B and C were heatmaps of lncRNAs, miRNAs, and mRNAs, and Fig. 2D, E and F were volcano plots of lncRNAs, miRNAs, and mRNAs. The differentially expressed genes of lncRNAs and mRNAs were shown in Supplementary Tables S2 and S3, respectively.

### Screening out key genes from ceRNA network and functional analysis

**Fig. 3:**
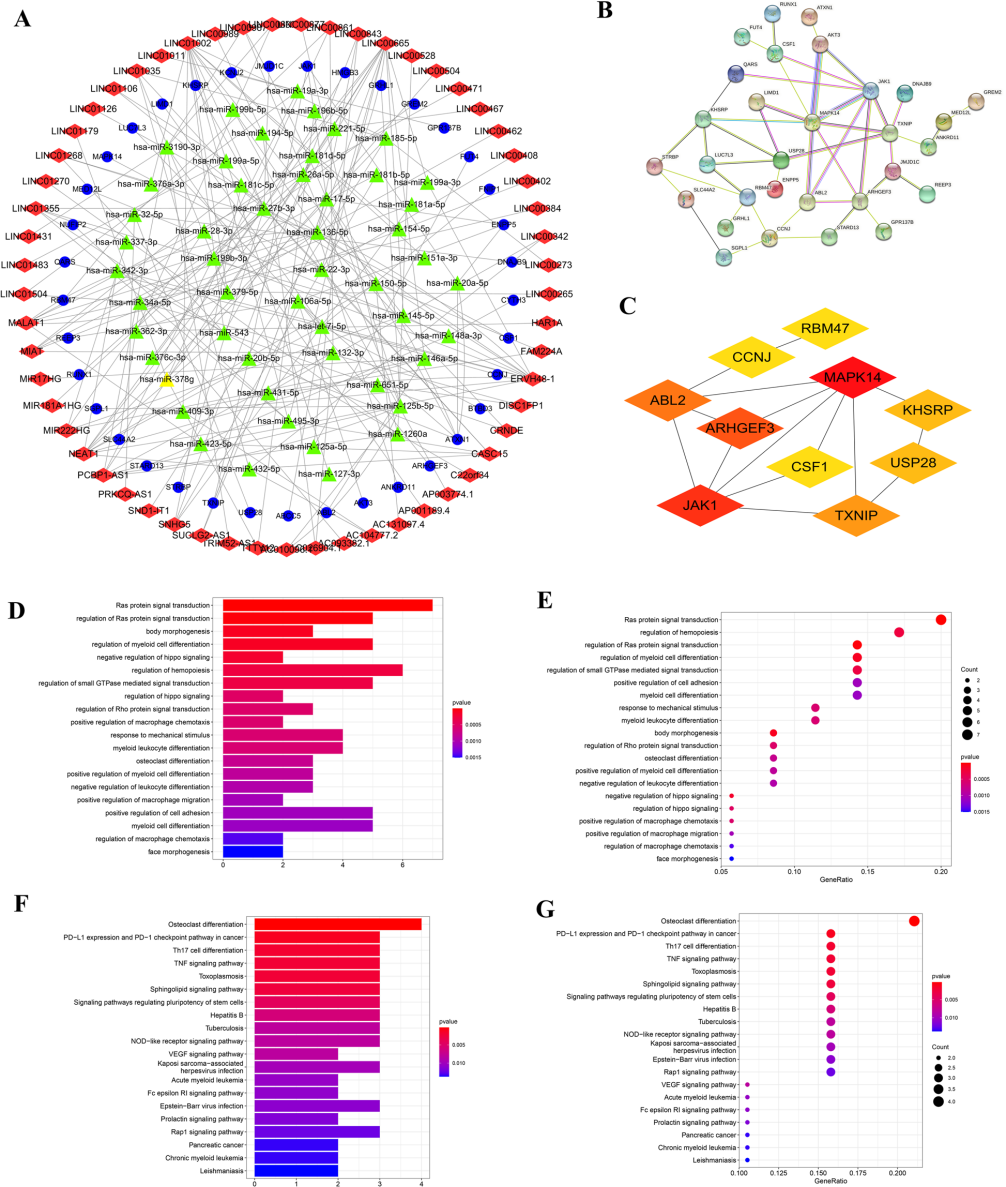
Screening key genes from ceRNA network and functional analysis. **A** Construction of a lncRNA-miRNA-mRNA ceRNA network for AML. In the ceRNA network, the blue and red nodes show decreased and increased expression of RNAs, respectively, while the colors are related to the absolute value of the fold change. Diamonds represent lncRNAs, ellipses represent miRNAs, rectangles represent mRNAs, and gray edges represent interactions among the lncRNAs-miRNAs and mRNAs. **B** PPI network of ceRNA network-related DEmRNAs. The nodes denote DEmRNAs (confidence score > 0.4) and the size of the nodes represents the degree of each node. **C** Top ten genes from the sub-network modularized by the plug-in cytoHubba. **D**, **E** Top 20 GO terms (*P* < 0.05) of the ceRNA-related DEmRNAs, respectively. **F**, **G** Top 20 KEGG pathways (*P* < 0.05) of the ceRNA-related DEmRNAs, respectively. Interactions and overlap among the important KEGG pathways. ceRNA: competing endogenous RNA;GO: Gene Ontology; KEGG: Kyoto Encyclopedia of Genes and Genomes

The lncRNAs and mRNAs targeted by miRNAs were screened based on the interactions between the DElncRNAs, DEmRNAs, and DEmiRNAs described above. Only 51 of 58 DEmiRNAs were predicted to target 57 of the 664 DElncRNAs based on DIANA-LncBase Version 2 experimental module. Using TargetScan, miRDB, and DIANA-TarBase Version 8, the target mRNAs of the 51 selected DEmiRNAs were selected. The predicted mRNAs were then compared with 3700 specific DEmRNAs, and only 37 mRNAs from both groups were targeted by the 51 miRNAs. The representative interactions among lncRNAs, miRNAs, and mRNAs are shown in Tables 2 and 3. Based on the interactions between lncRNA-miRNA and miRNA-mRNA, a lncRNA-miRNA-mRNA ceRNA network was constructed consisting of 57 lncRNAs, 51 miRNAs, and 37 mRNAs with a total of 90 interactions (Fig. 3A).

**Table 2:** Representative interactions between the miRNAs and lncRNAs for AML

miRNA	lncRNA
hsa-miR-19a-3p	PCBP1-AS1,
hsa-miR-106a-5p	SUCLG2-AS1
hsa-miR-17-5p	SUCLG2-AS1
hsa-miR-32-5p	PRKCQ-AS1
hsa-miR-125b-5p	AC010096.1
hsa-miR-34a-5p	DISC1FP1
hsa-let-7i-5p	NEAT1
hsa-miR-495-3p	NEAT1
hsa-miR-146a-5p	MIR222HG
hsa-miR-22-3p	LINC01035
hsa-miR-27b-3p	NEAT1
hsa-miR-136-5p	LINC01268
hsa-miR-125a-5p	LINC00843

**Table 3:** Representative interactions between the miRNAs and mRNAs for AML

miRNA	mRNA
hsa-miR-19a-3p	KCNJ2, GPR137B
hsa-miR-106a-5p	ENPP5, ATXN1, AKT3, REEP3, ARHGEF3
hsa-miR-17-5p	ENPP5, ATXN1, TXNIP, JAK1
hsa-miR-32-5p	RBM47, USP28, DNAJB9
hsa-miR-125b-5p	SGPL1
hsa-miR-34a-5p	SLC44A2, GREM2
hsa-let-7i-5p	LIMD1, BTBD3, CCNJ, QARS, LUC7L3, ABCC5, FNIP1
hsa-miR-495-3p	NUFIP2
hsa-miR-146a-5p	ABL2
hsa-miR-22-3p	MAPK14
hsa-miR-27b-3p	STRBP, KHSRP, JMJD1C, HMGB3, RUNX1, CSF1
hsa-miR-136-5p	ANKRD11
hsa-miR-125a-5p	FUT4

Based on the STRING database, we built a PPI network with the ceRNA-related DEmRNAs to investigate the function of DEmRNAs at the protein level and filter the functional genes (Fig. 3B). The PPI network consisted of 55 nodes and 112 edges. The plug-in “cytoHubba” was used to analyse the sub-network and mRNAs were chosen for further analysis. The top five mRNAs of MAPK14, JAK1, ARHGEF3, ABL2, and TXNIP were chosen for further analysis from the sub-network (Fig. 3C). The ceRNA regulatory axis (lncRNA-miRNA-mRNA) associated with these mRNAs were LINC01035-hsa-miR-22-3p-MAPK14, SUCLG2-AS1-hsa-miR-17-5p-JAK1, SUCLG2-AS1-hsa-miR-106a-5p- ARHGEF3, MIR222HG-hsa-miR-146a-5p-ABL2, and SUCLG2-AS1-hsa-miR-17-5p- TXNIP.

Since the expression of JAK1 has been reported to be closely related to AML [43], and SUCLG2-AS1 is a relatively important lncRNA among these ceRNA regulatory axes, so SUCLG2-AS1-hsa-miR-17-5p-JAK1 axis was selected among the top important genes for further study.

Using DAVID along with Metascape bioinformatic tools, we performed GO and KEGG pathway analyses with the 37 DEmRNAs. The top 20 GO terms (Fig. 3D, E) and top 20 KEGG pathways (Fig. 3F, G) were chosen for biological function analysis. The biological process of GO terms were “regulation of hemopoiesis” and “regulation of myeloid cell differentiation”. Among these pathways, “Th17 cell differentiation” and the “TNF signaling pathway” have been reported to be correlated with the proliferation, invasion, and metastasis of cancer in patients [44] (Supplementary Table S4).

### The expression of significant genes in AML cells by qRT-PCR

**Fig. 4:**
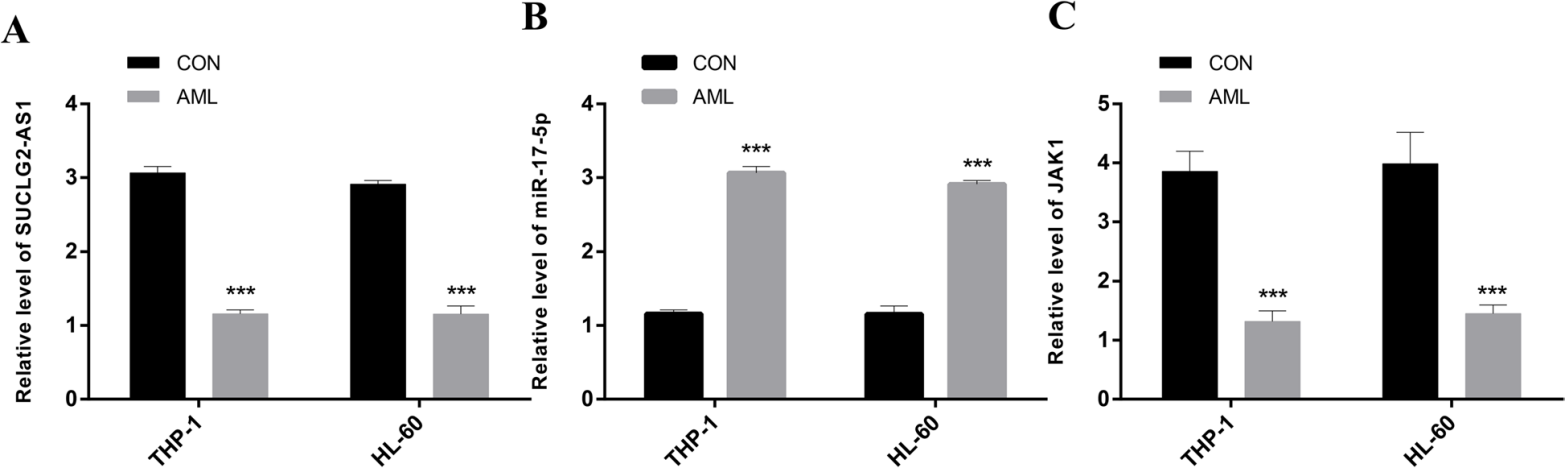
The expression of key genes by qRT-PCR. **A** SUCLG2-AS1. **B** miR-17-5p. **C** JAK1

To study the role of SUCLG2-AS1, miR-17-5p and JAK1 in AML cells, we analysed the expression of all of the above genes using qRT-PCR. The results showed that SUCLG2-AS1 and JAK1 were downregulated in AML cells compared with normal cells, while miR-17-5p were upregulated in AML cells compared with normal cells (Fig. 4A, B, C). This result is consistent with what bioinformatics predicted, and this result is consistent with the theoretical mechanism of ceRNA.

### Overexpression of SUCLG2-AS1 inhibits proliferation, migration and invasion and promotes apoptosis of AML cells

**Fig. 5:**
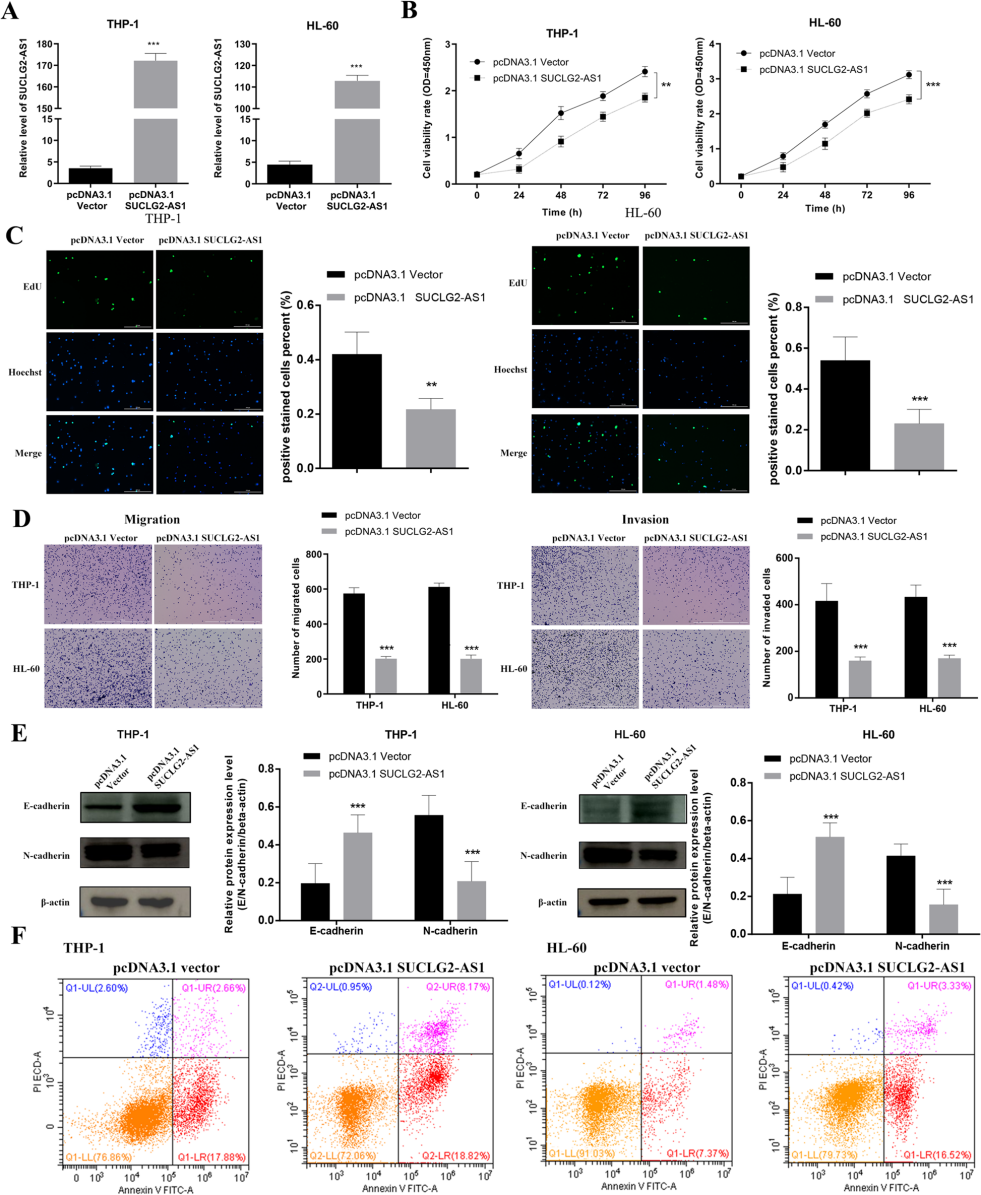
Overexpression of SUCLG2-AS1 inhibits proliferation, migration and invasion and promotes apoptosis of AML cells. **A** Validation of SUCLG2-AS1 overexpression in AML cells by qRT-PCR. **B** The viability was detected after overexpression of SUCLG2-AS1 by CCK-8 assay. **C** The cell proliferation was detected after overexpression of SUCLG2-AS1 by EdU assay. **D** Detection of cell migration and invasion after overexpression of SUCLG2-AS1 by Transwell assay. **E** Detection of cell invasion after overexpression of SUCLG2-AS1 by EMT assay. **F** Detection of cell apoptosis after overexpression of SUCLG2-AS1 by flow cytometry

To assess the impact of SUCLG2-AS1 on AML cells, we transfected THP-1 and HL-60 cells with pcDNA3.1 SUCLG2-AS1 (Supplementary Figure 1) [45] and confirmed the transfection efficiency by qRT-PCR analysis. The results showed that the transfection of SUCLG2-AS1 was successful, and the expression of pcDNA3.1 SUCLG2-AS1 group was significantly higher than that of the nontransfected (pcDNA3.1 Vector)group (Fig. 5A). CCK-8 assays revealed that the proliferation of AML cells transfected with pcDNA3.1 SUCLG2-AS1 was significantly decreased compared to that of the pcDNA3.1 Vector group (Fig. 5B). EdU assay and Transwell assay showed that overexpression of SUCLG2-AS1 inhibits proliferation, migration and invasion in AML cells (Fig. 5C, D). As we all know, downregulated expression of E-cadherin and upregulated expression of N-cadherin are an important marker of EMT. In this study, WB experiment showed upregulated expression of E-cadherin and downregulated expression of N-cadherin in pcDNA3.1 SUCLG2-AS1 group compared to that of the pcDNA3.1 Vector group (Fig. 5E), which is exactly the opposite to the process of EMT, indicating that overexpression of SUCLG2-AS1 inhibits cell invasion and metastasis. In addition, the apoptotic rate of AML cells transfected with pcDNA3.1 SUCLG2-AS1 was increased compared to that of the pcDNA3.1 Vector group (Fig. 5F). Taken together, these findings suggested that overexpression of SUCLG2-AS1 inhibits proliferation, migration and invasion and promotes apoptosis of AML cells.

### SUCLG2-AS1 regulates the occurrence and development of AML through miR-17-5p

**Fig. 6:**
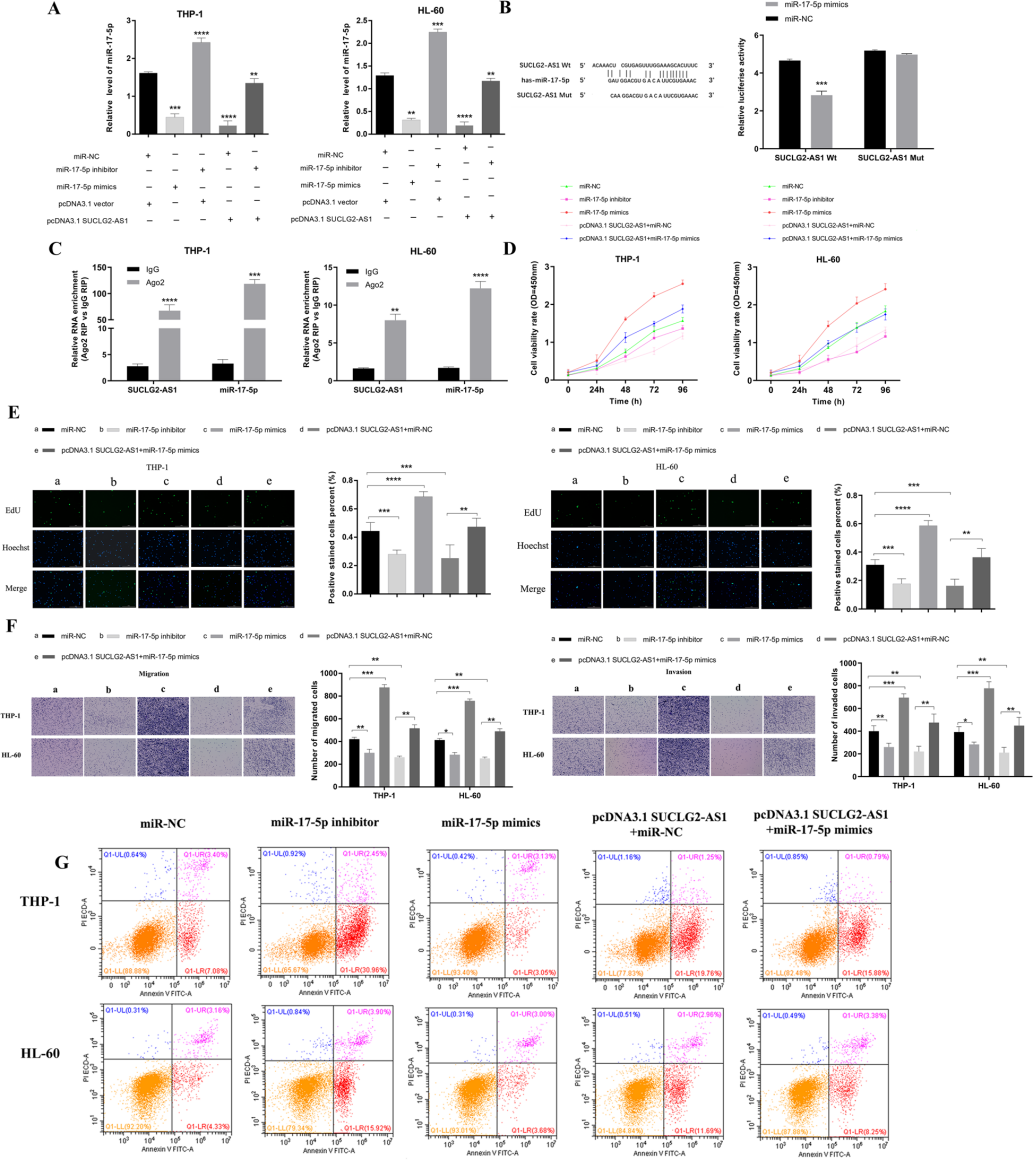
SUCLG2-AS1 functioned via negatively regulating miR-17-5p expression in AML cells. **A** The regulatory function of SUCLG2-AS1 on the expression level of miR-17-5p in AML cells. **B** The luciferase reporter assay validated the relationships between SUCLG2-AS1 and miR-17-5p. **C** Ago2 RNA-binding protein immunoprecipitation assay to verify SUCLG2-AS1 binding with miR-17-5p. **D**, **E** SUCLG2-AS1 can affect the proliferation of AML cells through miR-17-5p by CCK-8 assay and EdU assay. **F** SUCLG2-AS1 can affect the migration and invasion of AML cells through miR-17-5p by Transwell assay. **G** SUCLG2-AS1 can regulate the apoptosis of AML cells through miR-17-5p by flow cytometry

Bioinformatics databases Diana-lncbase Version 2, miRDB and Annolnc were used to predict the possible binding miRNAs and binding sites of SUCLG2-AS1.Combined with the lncRNA/miRNA/mRNA coregulatory network of AML constructed in previous studies, we found that SUCLG2-AS1 may bind to miR-17-5p and act as ceRNA. To observe the relationship between SUCLG2-AS1 and miR-17-5p in AML cells, and to study the effect of SUCLG2-AS1 on the expression level of miR-17-5p, AML cells THP-1 and HL-60 were transfected with (a) miR-NC, (b) miR-17-5p inhibitor, (c) miR-17-5p mimics, (d) pcDNA3.1 SUCLG2-AS1 + miR-NC,and (e) pcDNA3.1 SUCLG2-AS1 + miR-17-5p mimics. qRT-PCR analysis suggested that SUCLG2-AS1 overexpression can inhibit the expression of miR-17-5p in AML cells, and there may be an interaction relationship between SUCLG2-AS1 and miR-17-5p (Fig. 6A). To further verify the combination of SUCLG2-AS1 and miR-17-5p, we performed a dual-luciferase reporter assay and RIP assay. A dual-luciferase reporter gene detection experiment markedly confirmed the binding between SUCLG2-AS1 and miR-17-5p (Fig. 6B). According to RIP assays, in AML cells THP-1 and HL-60, Ago2 antibody significantly enriched SUCLG2-AS1 and miR-17-5p compared with the negative control IgG group (Fig. 6C). CCK-8 assays and EdU assay revealed that SUCLG2-AS1 regulates the proliferation of AML cells through miR-17-5p (Fig. 6D, E). Transwell assay showed that SUCLG2-AS1 regulates the migration and invasion of AML cells through miR-17-5p (Fig. 6F). Additionally, flow cytometry assays showed that the apoptotic rate of AML cells was increased by pcDNA3.1 SUCLG2-AS1, and partially reversed through cotransfection with miR-17-5p mimics (Fig. 6G). These results indicated that SUCLG2-AS1 functioned by negatively regulating miR-17-5p expression in AML cells.

### SUCLG2-AS1 regulates the expression of JAK1 through competitive binding of miR-17-5p

**Fig. 7:**
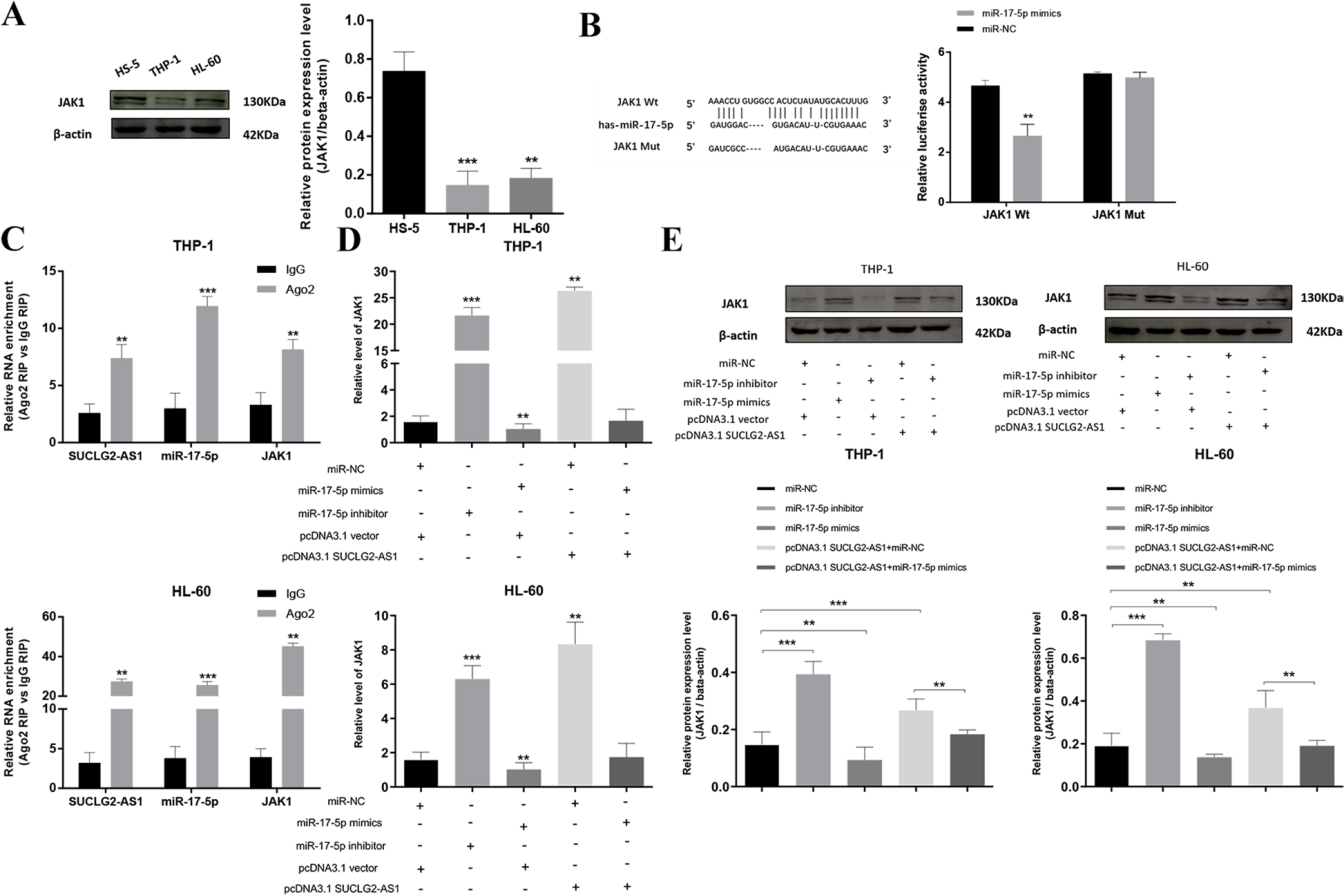
SUCLG2-AS1 regulates the expression of JAK1 through competitive binding of miR-17-5p. **A** The validation of JAK1 expression in AML cells and normal cells by WB. **B** The luciferase reporter assay validated the relationships between miR-17-5p and JAK1. **C** Ago2 RNA-binding protein immunoprecipitation assay to verify miR-17-5p binding with JAK1. **D** The regulation of SUCLG2-AS1 on the expression level of JAK1 through miR-17-5p in AML cells by qRT-PCR. **E** The regulation of SUCLG2-AS1 on the expression level of JAK1 through miR-17-5p in AML cells by WB

WB assay validated that the expression of JAK1 was lower in AML cells than that in normal cells (Fig. 7A). According to the dual-luciferase reporter assay, miR-17-5p mimics significantly inhibited the luciferase activity of JAK1-WT compared with the control group JAK1-WT + miR-NC (*P* < 0.001). miR-17-5p mimics did not significantly inhibit the luciferase activity of JAK1-MUT, suggesting that miR-17-5p could bind to JAK1 through this binding site (Fig. 7B). RIP assays showed that miR-17-5p can achieve complementary pairing binding with JAK1 through RISC, and SUCLG2-AS1 may regulate JAK1 by binding to miR-17-5p (Fig. 7C). To further observe whether miR-17-5p can silence the expression of JAK1 in AML cells, and the regulation of SUCLG2-AS1 on the expression level of JAK1 by binding to miR-17-5p, mRNA and protein expression of JAK1 were detected by qRT-PCR and WB respectively after transfection. Accoding to Fig. 7D and E, the expression levels of JAK1 in THP-1 and HL-60 cells were significantly increased after miR-17-5p knockdown, while they were significantly decreased after miR-17-5p overexpression, suggesting that miR-17-5p can downregulate the expression of JAK1.After SUCLG2-AS1 overexpression, the expression of JAK1 was significantly increased, indicating that SUCLG2-AS1 could upregulate the expression of JAK1. However, after cotransfection of pcDNA3.1 SUCLG2-AS1 and miR-17-5p mimics, the expression of JAK1 in cells could be significantly restored and approached to the normal level, suggesting that miR-17-5p mimics could inhibit the upregulation of JAK1 by SUCLG2-AS1 overexpression. This also suggests that SUCLG2-AS1 regulates the expression of JAK1 through miR-17-5p.

### miR-17-5p regulates the occurrence and development of AML through JAK1

**Fig. 8:**
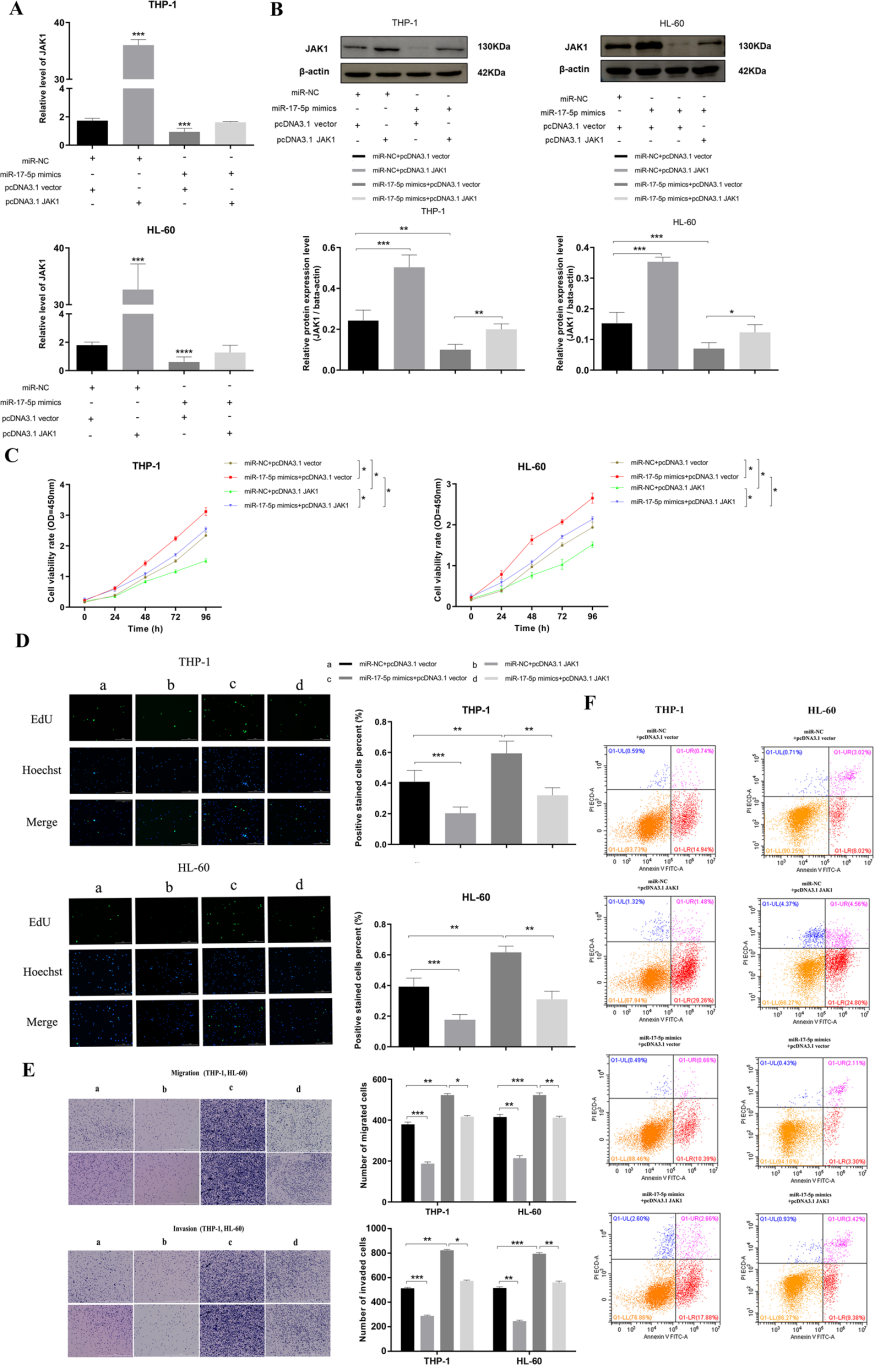
miR-17-5p regulates the occurrence and development of AML through JAK1. **A** The negative regulatory effect of miR-17-5p on JAK1 expression levels in AML cells was detected by qRT-PCR. **B** The negative regulatory effect of miR-17-5p on JAK1 expression levels in AML cells was detected by WB. **C**, **D** SUCLG2-AS1 can affect the proliferation of AML cells through miR-17-5p by CCK-8 assay and EdU assay. **E** miR-17-5p can affect the migration and invasion of AML cells through JAK1 by Transwell assay. **F** miR-17-5p can regulate the apoptosis of AML cells through JAK1 by flow cytometry

In order to further verify the regulatory effect of miR-17-5p on JAK1, the JAK1 overexpression plasmid pcDNA3.1 JAK1 (Supplementary Figure 2) was constructed using the pcDNA3.1 (+) vector, and transfected into AML cells THP-1 and HL-60. Then the expression of JAK1 was detected by qRT-PCR and WB. Accoding to Fig. 8A and B, compared with the control group, the expression levels of JAK1 mRNA and protein in pcDNA3.1JAK1 cells were significantly increased after transfection, and this phenomenon was significantly reversed after cotransfection with miR-17-5p mimics, which promoted the expression of JAK1 to be close to normal. This further confirmed the negative regulation of miR-17-5p on JAK1 at the posttranscriptional level. CCK-8 and EdU assay revealed that miR-17-5p regulates the proliferation of AML cells through JAK1 (Fig. 8C, D). Transwell assay showed that miR-17-5p regulates the migration and invasion of AML cells through JAK1 (Fig. 8E). As in the previous experiment, the results of apoptosis showed that overexpression of miR-17-5p can inhibit the apoptosis of AML cells, however, after cotransfection of miR-17-5p mimics and PCDNA3.1 JAK1, the apoptosis rate of AML cells was restored to the normal level, suggesting that the regulation process of miR-17-5p on AML cell apoptosis is closely related to JAK1 (Fig. 8F). All quantitative graph of apoptosis results was in Supplementary Figure 3.

### SUCLG2-AS1 is regulated by m6A modification of WTAP in AML cells

**Fig. 9:**
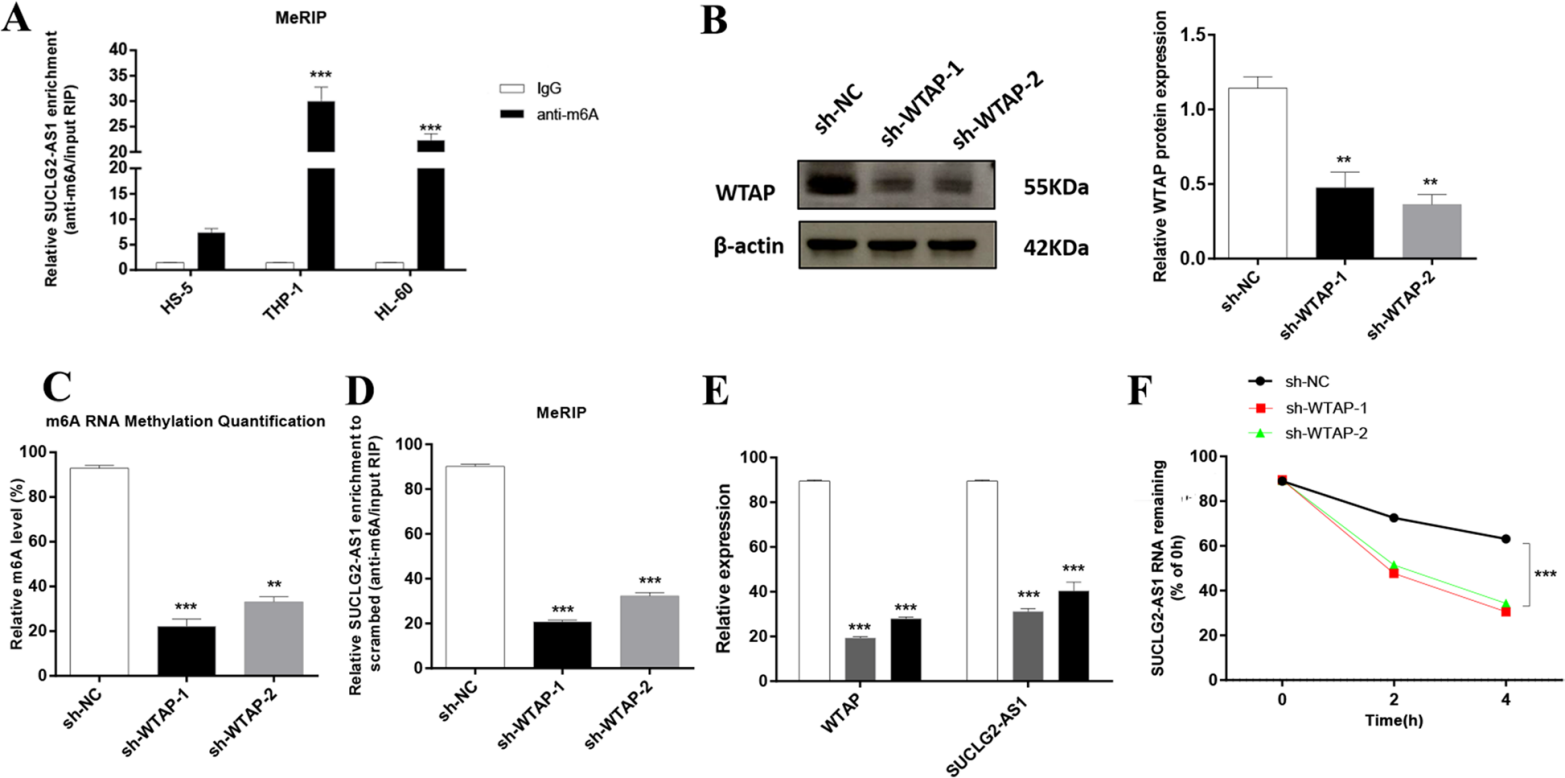
The m6A modification was enriched in SUCLG2-AS1 and improved its transcripts stability. **A** The m6A methylation level of SUCLG2-AS1 in AML cells and control cells were determined by MeRIP-qPCR. **B** The knockdown effect of sh-WTAP was verified by Western blot (WB) analysis in THP-1 cells. **C** m6A methylation level in THP-1 cells after WTAP was knocked down. **D** Changes in m6A modified SUCLG2-AS1 levels upon WTAP was knockdown in THP-1 cells. **E** Transcript levels of WTAP and SUCLG2-AS1 in negative control and sh-WTAP THP-1 cells. **F** Reduction of SUCLG2-AS1 RNA stability in WTAP knockdown THP-1 cells as compared to control. Cells were treated with actinomycin D and RNA was isolated at 0, 2, and 4 h. Data represent the mean ± SD. *P < 0.05, **P < 0.01, ***P < 0.001. The experiments were independently repeated at least three times

We checked the m6A methylation level of SUCLG2-AS1 in AML cells by MeRIP-qPCR. According to the results, the m6A methylation level was more enriched within SUCLG2-AS1 in THP-1 and HL-60 cells than that in controls (Fig. 9A). As WTAP is a crucial m6A methyltransferase, we then performed shRNA-mediated silencing of WTAP (Fig. 9B) and found that downregulation of WTAP resulted in the decreased m6A levels of both total RNA and SUCLG2-AS1 in THP-1 cells (Fig. 9C, D). Then we explored whether m6A modification could affect SUCLG2-AS1 RNA metabolism and found that knockdown of WTAP led to lower expression of SUCLG2-AS1 in THP-1 cells (Fig. 9E). After new RNA synthesis was blocked with actinomycin D, we measured the loss of SUCLG2-AS1. The results indicated that SUCLG2-AS1 showed lower RNA stability after silencing of WTAP in THP-1 cells (Fig. 9F). It was suggested that the m6A level of SUCLG2-AS1 was higher in AML cells, and its modification in SUCLG2-AS1 improved transcripts stability.

### Elevated WTAP is associated with poor prognosis of AML patients

WTAP is an important m6A methylation modification transferase, some studies confirmed that WTAP methylation modification was closely related to AML [46]. Therefore, we use bioinformatics methods to verify whether m6A methylation modification is associated with AML.

**Fig. 10:**
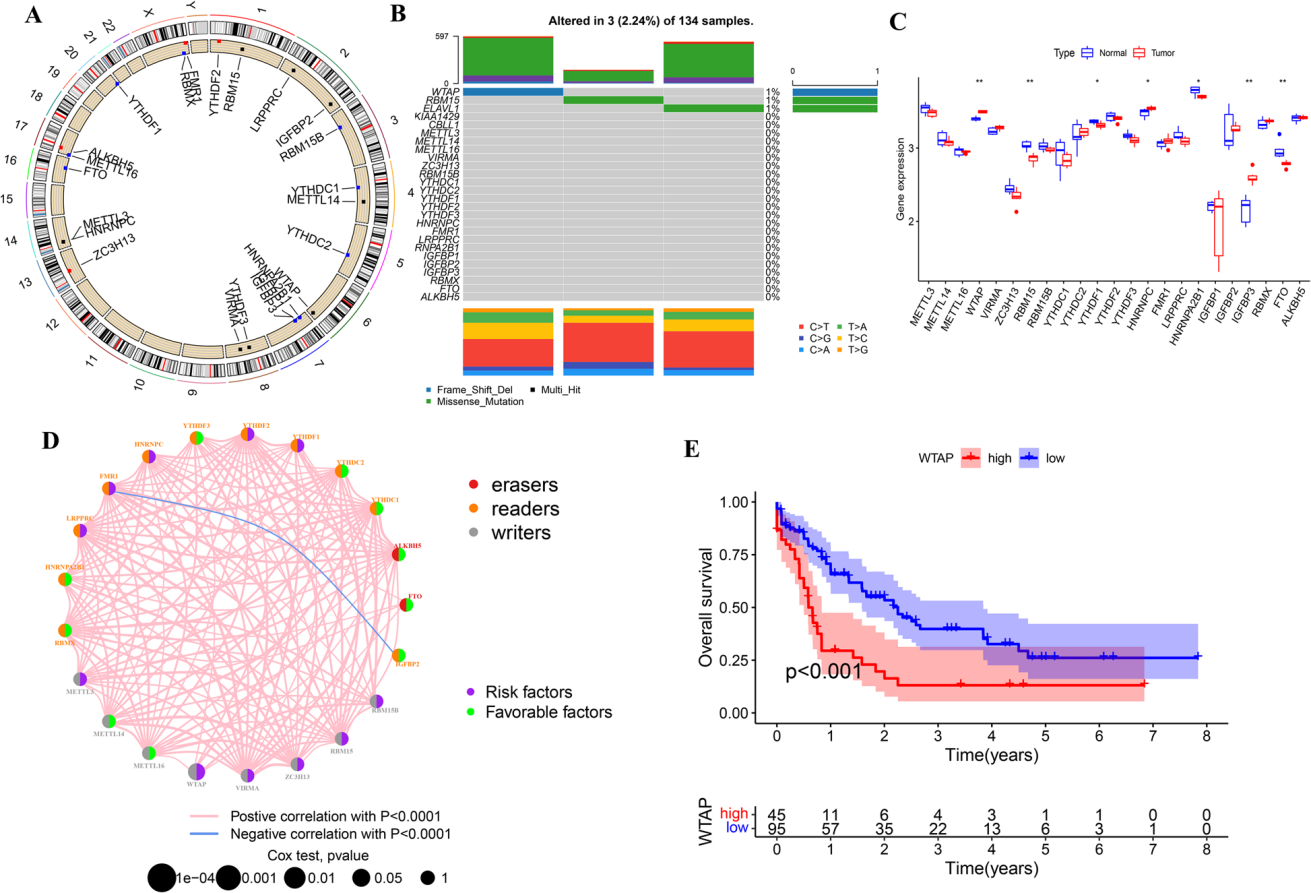
Overview of m6A gene locus and gene information. **A** The location of mutations in m6A regulators. **B** Waterfall plot of m6A regulators mutation genes and mutation types. **C** Comparison of gene expression levels of 23 regulators between the normal and tumor tissue cohorts. **D** Landscape and inner crosslink between 23 m6A regulators. **E** The Kaplan–Meier survival analysis of WTAP. **P < 0.01, ***P < 0.001. m6A, N6-methyladenosine

The transcription levels of 23 m6A regulators, which included 13 readers, 8 writers, and 2 erasers, as well as the copy number variations (CNVs) and somatic mutations of these regulators, have been described in a previous study [47]. The chromosomal locations of the amplification mutations were distributed randomly and unequally across the chromosomes, as shown in Fig. 10A. Of the 134 AML samples used in this study, 3 contained mutations related to m6A (2.24% mutation rate). The assay of AML specimens found that only WTAP (1%), RBM15 (1%) and ELAVL1 (1%) showed any mutation frequency, while the other genes did not (Fig. 10B). The differential expression of m6A regulators in normal and tumor samples were shown by hierarchical clustering analysis in Fig. 10C. The m6A regulators of WTAP, RBM15, IGFBP3, and FTO were highly expressed in AML samples compared with normal samples. The m6A regulators of WTAP were the risk genes in AML (Fig. 10D, *P* < 0.05), and AML patients with high WTAP showed poor overall survival (Fig. 10E). So elevated WTAP is associated with poor prognosis of AML patients.

### WTAP and its m6A methylation are significantly increased in AML cells

**Fig. 11:**
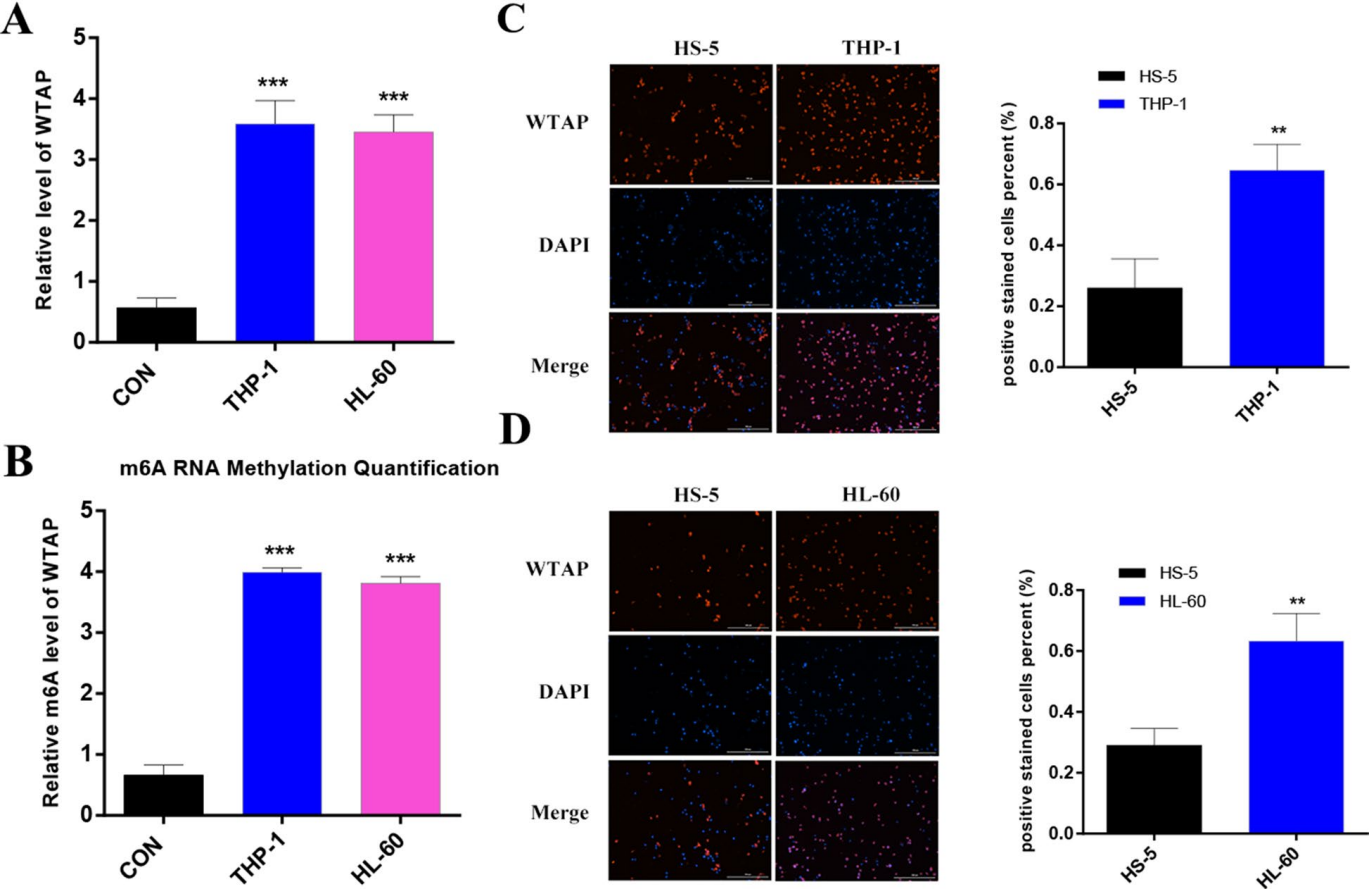
WTAP expression and m6A modification in AML cells. **A** The expression of WTAP in AML cells and control cells were determined by qRT-PCR. **B** The m6A level of WTAP in AML cells. **C**, **D** WTAP expression in AML Cells were stained for WTAP (red), and nuclei were stained with DAPI (blue). Scale bar: 100 μm. *P < 0.05, ***P < 0.001. ns, no significance

To investigate the role of WTAP in AML, we first checked the expression of WTAP and its m6A levels in AML (THP-1 and HL-60) cells and control HS-5 cells. The data suggested that AML significantly increased WTAP level compared with that of controls (Fig. 11A). Meanwhile, the m6A level of WTAP was also significantly increased in AML compared with that of controls (Fig. 11B). We also measured the expression of WTAP in AML and control cells by IF. As shown in Fig. 11C and D, AML cells exhibited a significantly higher expression of WTAP. These findings indicate that AML increases WTAP, and this result is consistent with what bioinformatics predicted.

## Discussion

In recent years, studies have found that the ceRNA mechanism is widely present in various cancers such as gastric cancer [48], colon cancer and bladder cancer, and plays a role in tumor gene regulation and biological processes such as tumor cell proliferation, invasion, metastasis, apoptosis and cell cycle. According to the ceRNA mechanism, lncRNAs regulate miRNAs by binding their target sites on protein-coding mRNA molecules [49]. In this study, we used bioinformatics methods for prediction and analysis and used qRT-PCR to verify these results. The results showed that SUCLG2-AS1 and JAK1 were expressed at low levels, while miR-17-5p was highly expressed in AML cells. This is in accordance with the ceRNA principle.

A large number of studies have reported that lncRNAs can participate in the regulation of malignant tumor progression by affecting the growth and proliferation, migration and invasion of tumor cells and other cell behaviors [50]. In this study, SUCLG2-AS1-overexpressing AML cells were constructed by transfection of the SUCLG2-AS1 overexpression vector, and the changes in malignant characteristics of AML cells after SUCLG2-AS1 overexpression were further observed through various in vitro experiments. In CCK-8 and EdU experiments, the results consistently showed that overexpression of SUCLG2-AS1 could significantly inhibit the proliferation of AML cells. In Transwell cell migration/invasion assay, it was found that SUCLG2-AS1-overexpressing AML cells showed a significant downward trend in migration and invasion, suggesting that SUCLG2-AS1 could inhibit the migration and invasion of AML cells. In addition, by detecting the expression of E-cadherin and N-cadherin proteins in SUCLG2-AS1-overexpressing AML cells, it was observed that SUCLG2-AS1 could affect the EMT process. Since the EMT process is related to tumor invasion and metastasis, we speculated that the effect of SUCLG2-AS1 on the migration and invasion ability of AML cells might be related to the EMT process. During the detection of cell apoptosis, it was found that the apoptosis rate of AML cells overexpressing SUCLG2-AS1 showed an increasing trend, and both the early and late apoptosis rates increased, which also suggested that SUCLG2-AS1 may promote the apoptosis process of AML cells. All the above in vitro experiments suggested that SUCLG2-AS1 could be used as a protective lncRNA to inhibit the malignant biological behavior of AML cells.

In order to further verify the targeted binding relationship between SUCLG2-AS1 and miR-17-5p, we overexpressed SUCLG2-AS1. The results showed that miR-17-5p expression was significantly downregulated after overexpression of SUCLG2-AS1, while SUCLG2-AS1 was not significantly changed after overexpression or knockdown of miR-17-5p. It can be speculated that SUCLG2-AS1 has a certain inhibitory effect on the expression of miR-17-5p in AML cells, and the binding relationship between the two was verified by a luciferase reporter gene assay. We further verified the binding relationship between SUCLG2-AS1 and miR-17-5p through Ago2 RIP experiments, and found that the Ago2 antibody could significantly enrich SUCLG2-AS1 and miR-17-5p simultaneously. Comprehensive analysis showed that SUCLG2-AS1 could bind to miR-17-5p through AGO2-containing RISC, and then play a regulatory role in its target genes. Similarly, we used a luciferase reporter gene assay and an Ago2 RIP experiment to verify the binding relationship between miR-17-5p and JAK1.The results showed that miR-17-5p in AML cells targeted to bind JAK1 and inhibited its expression, and SUCLG2-AS1 interfered with the inhibitory effect of miR-17-5p on JAK1 through competitive binding of miR-17-5p.

An increasing number of studies have found that miR-17-5p is upregulated in a variety of malignant tumors and plays an obvious cancer-promoting role [51], including breast cancer, colon cancer, and thyroid cancer, etc. Moreover, miR-17-5p can bind to a variety of lncRNAs to regulate the biological processes of different tumors [52]. In this study, in order to verify the function of miR-17-5p in AML and whether SUCLG2-AS1 plays a regulatory role in AML cells through miR-17-5p, we conducted a series of experiments for verification. In CCK-8 and EdU experiments, miR-17-5p was consistently found to promote the proliferation of AML cells, and SUCLG2-AS1 was found to regulate the malignant proliferation of AML cells through competitive binding of miR-17-5p. In the Transwell cell migration/invasion assay, it was observed that miR-17-5p overexpression could promote the migration or invasion of AML cells, while cotransfection of miR-17-5p mimics could reverse the inhibitory regulation of SUCLG2-AS1 overexpression on the migration or invasion of AML cells. It is speculated that SUCLG2-AS1 can inhibit the migration and invasion of AML cells by targeting miR-17-5p. In previous studies, we found that SUCLG2-AS1 could affect the EMT process of AML cells. In this chapter, we confirmed the promoting effect of miR-17-5p on the EMT process through Western Blot. It was also confirmed that SUCLG2-AS1 could affect the EMT process through miR-17-5p. At the same time, miR-17-5p was detected to inhibit the apoptosis of AML cells, and the effect of SUCLG2-AS1 on apoptosis was also accomplished through miR-17-5p. Our study further confirmed that miR-17-5p can also act as a cancer-promoting molecule in AML to regulate the malignant progression of AML, and it was also confirmed that SUCLG2-AS1 plays a regulatory function through miR-17-5p. Using the same experimental method, we overexpressed JAK1, and the results confirmed that miR-17-5p can affect the biological processes of AML cells by inhibiting JAK1.

N6-methyladenosine (m6A) modification has attracted increasing attention, especially in human cancer tumorigenesis [53]. Studies have shown that more than 7 000 different mRNAs and more than 300 lncRNAs molecules are m6A methylated [54, 55], meaning that m6A methylation may affect genes expression extensively. Its regulation is coregulated by methyltransferase (METTL3, METTL14, WTAP, etc.)/demethylase (FTO, ALKBH5, etc.) and some RNA-binding proteins (YTHDF1/2/3, ELAVL1, etc.) [56]. Wilms’ tumor 1-associating protein (WTAP) has been shown to regulate recruitment of the m6A methyltransferase complex to mRNA targets [57]. WTAP is considered as a pervasive internal modification of mRNA and plays critical roles in the progression of a variety of human diseases including cancers. Results showed that knocking down WTAP significantly decreased proliferation and increased apoptosis by affecting alternative splicing in AML cells [46]. In this study, it was discovered that m6A methylation was increased within SUCLG2-AS1 in AML cells. Additionally, WTAP regulated the m6A modification in SUCLG2-AS1, thus affecting its RNA stability. It was thus speculated that the enhancement of SUCLG2-AS1 in AML may be attributed to the m6A modification.

However, there are a few limitations of this study. First, the effect of the SUCLG2-AS1- miR-17-5p-JAK1 axis on AML has not been studied at the animal level. Second, further studies of these genes are needed in clinical trials to confirm our conclusions. We will work on them in the future.

## Conclusion

Our study indicates that SUCLG2-AS1 is downregulated in AML cells. Overexpression of SUCLG2-AS1 inhibits the proliferation, migration and invasion of AML cells, and promotes the apoptosis of AML cells. MiR-17-5p was highly expressed in AML cells, and JAK1, the target gene of miR-17-5p, was expressed at low levels in AML cells. SUCLG2-AS1 inhibits the silencing effect of miR-17-5p on JAK1 through competitive binding of miR-17-5p, thus playing a regulatory role in the occurrence and development of AML. Meanwhile, SUCLG2-AS1 is regulated by the m6A modification of WTAP. In conclusion, the WTAP-SUCLG2-AS1- miR-17-5p-JAK1 axis may be critical in regulating the development and progression of AML and may be a therapeutic target for intervention in AML.

### Supplementary Information

**Additional file 1: Supplementary Table S1.** The datasets in this study.

** Additional file 2: Supplementary Table S2.** The differentially expressed genes of lncRNA.

** Additional file 3: Supplementary Table S3.** The differentially expressed genes of mRNA.

** Additional file 4: Supplementary Table S4.** GO biological process terms and KEGG enriched pathways for ceRNA network-related DEmRNAs.

** Additional file 5: Supplementary Figure 1.** The overexpression plasmid pcDNA3.1 SUCLG2-AS1. **Supplementary Figure 2.** The overexpression plasmid pcDNA3.1 JAKI. **Supplementary Figure 3.** Percentage of apoptotic cells.

** Additional file 6.**

## Data Availability

All data are fully available without restrictions. Publicly available datasets were analyzed in this study. The following information was supplied regarding data availability: GEO (http://www.ncbi.nlm.nih.gov/geo/): GSE96535, GSE142699; copy number variant (CNV): https://portal.gdc.cancer.gov/cart.

## Abbreviations

AML: Acute myeloid leukemia
WTAP: Wilms tumor 1-associating protein
m6A: N6-methyladenosine
ceRNA: competing endogenous RNA
lncRNA: Long noncoding RNAs
RIP: RNA Binding Protein Immunoprecipitation
JAK1: a tyrosine protein kinase
SUCLG2-AS1: a long noncoding RNAs
miR−17−5p: a microRNAs
XIST: a long noncoding RNAs
RBM15/15B: RNA binding motif protein 15/15B
METTL3: Methyltransferase Like 3
HOXD: a mRNA
PRC2: Polycomb repressive complex 2
miRNAs: microRNAs
GEO: Gene Expression Omnibus
GO: Gene Ontology
KEGG: Kyoto Encyclopedia of Genes and Genomes
PPI: Integration of protein-protein interaction
STRING: Search Tool for the Retrieval of Interacting Genes
CNV: copy number variant
circRNA: Circular RNA
EMT: epithelial-mesenchymal transition
WB: Western Blot
