# Supplementary material for: Regulatory function and mechanism research for m6A modification WTAP via SUCLG2-AS1- miR-17-5p-JAK1 axis in AML
Source: BMC Cancer. 2024 Jan 17;24:98. doi: 10.1186/s12885-023-11687-4 (PMC10795285; doi:10.1186/s12885-023-11687-4)
Supplement: Supplementary file 1 — Additional file 1: Supplementary Table S1. The datasets in this study. [file 12885_2023_11687_MOESM1_ESM.docx]

| Disease name | Chip properties | Data sources | Amount | Control | Case |
| --- | --- | --- | --- | --- | --- |
| AML(Acute myeloid leukemia) | lncRNA-seq | GEO(GSE96535) | 12 | 6 | 6 |
| AML | miRNA-seq | GEO(GSE142699) | 48 | 24 | 24 |
| AML | mRNA-seq | GEO(GSE96535) | 12 | 6 | 6 |
| AML | CNV(copy number variant ) | TCGA | 23 m6A modulatos | - | - |

Supplementary Table S1 The datasets in this study.
