# Supplementary material for: Regulatory function and mechanism research for m6A modification WTAP via SUCLG2-AS1- miR-17-5p-JAK1 axis in AML
Source: BMC Cancer. 2024 Jan 17;24:98. doi: 10.1186/s12885-023-11687-4 (PMC10795285; doi:10.1186/s12885-023-11687-4)
Supplement: Supplementary file 2 — Additional file 2: Supplementary Table S2. The differentially expressed genes of lncRNA. [file 12885_2023_11687_MOESM2_ESM.docx]

**Supplementary Table S2 The differentially expressed genes of lncRNA**

| id | logFC | AveExpr | t | P.Value | adj.P.Val | B |
| --- | --- | --- | --- | --- | --- | --- |
| AC090409.2 | -4.869216824 | 4.20897129 | -27.8192845 | 2.46E-12 | 1.60E-08 | 18.03575612 |
| AC103736.1 | -3.986306838 | 5.760042749 | -24.47121705 | 1.13E-11 | 3.33E-08 | 16.81402108 |
| AC104241.2 | -4.777978407 | 4.138478812 | -23.84420018 | 1.54E-11 | 3.33E-08 | 16.55801997 |
| AC010247.1 | -4.191832218 | 4.037210321 | -22.23742084 | 3.52E-11 | 5.70E-08 | 15.85692928 |
| LINC01736 | -3.784504152 | 4.38985955 | -20.5287158 | 9.03E-11 | 1.17E-07 | 15.03226663 |
| LINC01644 | -2.948415193 | 3.409308469 | -17.87565422 | 4.58E-10 | 4.68E-07 | 13.56043357 |
| AL161785.1 | -3.138598171 | 5.884302082 | -17.72847936 | 5.05E-10 | 4.68E-07 | 13.4710061 |
| AL031283.1 | -3.357331552 | 5.144600096 | -17.11371501 | 7.62E-10 | 5.85E-07 | 13.08764353 |
| AC139720.1 | -3.894396645 | 5.453665695 | -17.02338925 | 8.11E-10 | 5.85E-07 | 13.02994559 |
| AC022706.1 | -2.968841975 | 7.738924918 | -16.3432192 | 1.30E-09 | 8.45E-07 | 12.58366271 |
| AC087463.4 | 2.034902449 | 3.56595354 | 15.81947271 | 1.90E-09 | 1.12E-06 | 12.22518339 |
| LINC00861 | -2.939898508 | 6.053998298 | -15.54839531 | 2.32E-09 | 1.26E-06 | 12.0343184 |
| AC099687.1 | 2.807905185 | 3.093453111 | 15.28282757 | 2.83E-09 | 1.26E-06 | 11.84367984 |
| AL139246.3 | -3.810393622 | 4.348657228 | -15.26238492 | 2.88E-09 | 1.26E-06 | 11.82885223 |
| AC105094.2 | -2.000564189 | 6.316747718 | -15.2501844 | 2.90E-09 | 1.26E-06 | 11.81999236 |
| AL137802.1 | -2.217504725 | 12.5266168 | -15.08805958 | 3.28E-09 | 1.33E-06 | 11.70150778 |
| LINC00239 | -4.510133192 | 6.220303277 | -14.95336333 | 3.64E-09 | 1.39E-06 | 11.60199409 |
| AC090409.1 | -3.089519741 | 7.460802049 | -14.55898329 | 4.95E-09 | 1.79E-06 | 11.30487829 |
| AL954642.1 | -6.793252307 | 5.805500013 | -14.03377331 | 7.54E-09 | 2.56E-06 | 10.89537943 |
| PTCSC3 | -4.782892587 | 7.575036149 | -13.97924631 | 7.89E-09 | 2.56E-06 | 10.85192854 |
| MIR181A1HG | 3.480424023 | 5.842514101 | 13.86226863 | 8.68E-09 | 2.68E-06 | 10.7581026 |
| HRAT92 | -5.291051668 | 5.367400235 | -13.53171336 | 1.14E-08 | 3.33E-06 | 10.48838659 |
| LINC01724 | -2.546151418 | 2.843253833 | -13.49099662 | 1.18E-08 | 3.33E-06 | 10.45468691 |
| AL451164.2 | -2.858815531 | 3.010739489 | -13.42499893 | 1.25E-08 | 3.33E-06 | 10.3998373 |
| AC092919.1 | -4.114510154 | 8.625492893 | -13.39784972 | 1.28E-08 | 3.33E-06 | 10.37719252 |
| TPRG1-AS1 | -3.687444451 | 4.424740965 | -13.29642948 | 1.40E-08 | 3.49E-06 | 10.29217594 |
| LINC00544 | -2.13463425 | 4.91995058 | -13.06116466 | 1.71E-08 | 4.01E-06 | 10.09235495 |
| LINC02432 | -1.681799081 | 5.523027893 | -13.04949833 | 1.73E-08 | 4.01E-06 | 10.0823501 |
| LINC01550 | -2.796743721 | 5.881380467 | -12.72967496 | 2.29E-08 | 5.12E-06 | 9.804460357 |
| AC233976.1 | -3.326928163 | 5.6386583 | -12.51326107 | 2.78E-08 | 6.01E-06 | 9.612380096 |
| LINC01503 | -2.794462934 | 7.372329727 | -12.06951613 | 4.17E-08 | 8.48E-06 | 9.207930408 |
| TRBV11-2 | -4.347581329 | 5.668693896 | -12.06787816 | 4.18E-08 | 8.48E-06 | 9.206410394 |
| AL135818.2 | -2.953799064 | 3.213264614 | -12.02658954 | 4.34E-08 | 8.54E-06 | 9.168028068 |
| LINC01341 | 2.612650275 | 6.646247369 | 11.91893301 | 4.81E-08 | 9.17E-06 | 9.067338828 |
| AC025031.4 | 2.025079381 | 5.529229926 | 11.83957932 | 5.18E-08 | 9.60E-06 | 8.992550457 |
| LINC01685 | -2.260733359 | 2.782147001 | -11.64221235 | 6.25E-08 | 1.13E-05 | 8.804406973 |
| AC004816.1 | -2.291114862 | 4.745129259 | -11.44113703 | 7.59E-08 | 1.31E-05 | 8.609541445 |
| AL121983.2 | -1.397555567 | 6.026044345 | -11.42999534 | 7.67E-08 | 1.31E-05 | 8.598648227 |
| AC002428.2 | 1.623887774 | 2.777069117 | 11.29493541 | 8.76E-08 | 1.42E-05 | 8.465790477 |
| AC007349.3 | -2.390276744 | 5.642895592 | -11.29307962 | 8.78E-08 | 1.42E-05 | 8.463954463 |
| LINC02541 | -1.439322323 | 3.978583604 | -11.22637485 | 9.37E-08 | 1.48E-05 | 8.397770292 |
| AC008083.2 | -3.43247863 | 6.591734471 | -11.0260994 | 1.14E-07 | 1.77E-05 | 8.196809146 |
| MIR222HG | 3.848563169 | 5.461598327 | 10.91710295 | 1.28E-07 | 1.93E-05 | 8.085999944 |
| DKFZp779M062 | -2.185515323 | 5.732490531 | -10.82824498 | 1.40E-07 | 2.06E-05 | 7.994901051 |
| AC243830.1 | -3.005256656 | 5.06490376 | -10.80607005 | 1.43E-07 | 2.06E-05 | 7.972059059 |
| AC007249.2 | -1.062523251 | 5.609601707 | -10.67461623 | 1.64E-07 | 2.28E-05 | 7.835757559 |
| AC124657.1 | 2.290222311 | 4.761699921 | 10.66846979 | 1.65E-07 | 2.28E-05 | 7.829346801 |
| LINC01506 | -2.845562199 | 10.3682357 | -10.56219655 | 1.84E-07 | 2.49E-05 | 7.717966094 |
| HAR1A | -2.163354607 | 7.267150227 | -10.52416428 | 1.91E-07 | 2.54E-05 | 7.677857662 |
| PCED1B-AS1 | -4.355569338 | 9.189982726 | -10.34556373 | 2.31E-07 | 2.99E-05 | 7.487733175 |
| AC005670.1 | -1.233834237 | 5.440283855 | -10.3282106 | 2.35E-07 | 2.99E-05 | 7.469102919 |
| LINC00494 | -1.6945015 | 4.162999187 | -10.30184518 | 2.42E-07 | 2.99E-05 | 7.44074326 |
| LINC02513 | -3.180314111 | 3.461852161 | -10.29349626 | 2.44E-07 | 2.99E-05 | 7.431749264 |
| LINC00462 | 1.580657062 | 5.790448432 | 10.23536173 | 2.60E-07 | 3.05E-05 | 7.368941343 |
| AC079466.2 | -1.730856592 | 4.832218535 | -10.23130333 | 2.61E-07 | 3.05E-05 | 7.364544798 |
| AL139352.1 | -3.405779419 | 5.114321341 | -10.22218409 | 2.63E-07 | 3.05E-05 | 7.354660047 |
| AC005833.2 | 3.447394418 | 6.556841081 | 10.11465025 | 2.96E-07 | 3.37E-05 | 7.23750336 |
| LINC02422 | -5.02069049 | 7.133642951 | -10.02669015 | 3.25E-07 | 3.64E-05 | 7.140848015 |
| LINC02195 | -3.158445024 | 5.411208707 | -9.991662156 | 3.38E-07 | 3.72E-05 | 7.102148961 |
| BX284668.5 | -2.57739486 | 12.05467925 | -9.903798787 | 3.72E-07 | 4.02E-05 | 7.004550299 |
| AL031705.1 | 4.096780258 | 4.973214266 | 9.864081745 | 3.88E-07 | 4.13E-05 | 6.960183679 |
| LINC02580 | -1.351134192 | 6.634103866 | -9.786707999 | 4.23E-07 | 4.43E-05 | 6.873303161 |
| AC122719.1 | -1.00541349 | 4.289819969 | -9.714206621 | 4.58E-07 | 4.68E-05 | 6.791351154 |
| AC026471.4 | 3.336527651 | 8.436576553 | 9.708851976 | 4.61E-07 | 4.68E-05 | 6.785277577 |
| AP005671.1 | -3.762201612 | 3.740737082 | -9.665695765 | 4.84E-07 | 4.83E-05 | 6.736221366 |
| AL109936.2 | -2.538544135 | 3.45665205 | -9.595296756 | 5.24E-07 | 5.15E-05 | 6.655792267 |
| LINC01871 | -5.139437896 | 8.866034329 | -9.547715658 | 5.52E-07 | 5.35E-05 | 6.601145314 |
| AC245014.1 | -1.619215056 | 12.07148157 | -9.483473988 | 5.94E-07 | 5.67E-05 | 6.526993981 |
| LINC01451 | -1.470537579 | 6.54192753 | -9.407382984 | 6.48E-07 | 6.04E-05 | 6.438611723 |
| AC005082.1 | -2.698135581 | 3.505947265 | -9.392990584 | 6.59E-07 | 6.04E-05 | 6.421826504 |
| LINC02190 | -2.728698726 | 4.348514995 | -9.39087612 | 6.60E-07 | 6.04E-05 | 6.419358671 |
| LINC01011 | -3.432806742 | 4.765329453 | -9.347023692 | 6.94E-07 | 6.26E-05 | 6.368071748 |
| MAPKAPK5-AS1 | -2.111953267 | 9.937090799 | -9.322554696 | 7.14E-07 | 6.35E-05 | 6.339366371 |
| AL355073.1 | -3.101762598 | 3.817164717 | -9.218467732 | 8.05E-07 | 7.06E-05 | 6.216548892 |
| LINC00534 | -2.436466705 | 4.770217714 | -9.187768854 | 8.34E-07 | 7.08E-05 | 6.180104858 |
| AP003774.3 | -3.540398 | 8.425890621 | -9.183243632 | 8.39E-07 | 7.08E-05 | 6.174724204 |
| LINC00989 | -2.915685551 | 9.590720919 | -9.182787497 | 8.39E-07 | 7.08E-05 | 6.174181721 |
| AL591848.4 | 1.060176081 | 6.407483511 | 9.127245371 | 8.96E-07 | 7.45E-05 | 6.107957625 |
| AL451069.1 | -2.134580774 | 3.990220645 | -9.102232316 | 9.22E-07 | 7.45E-05 | 6.078025068 |
| AC005280.2 | -4.580640727 | 6.432205015 | -9.098692808 | 9.26E-07 | 7.45E-05 | 6.073783936 |
| AF279873.3 | 1.698891233 | 3.403428755 | 9.095243904 | 9.30E-07 | 7.45E-05 | 6.069650059 |
| LINC00402 | -3.23826988 | 5.986671938 | -9.074641086 | 9.52E-07 | 7.54E-05 | 6.04492845 |
| AC136944.1 | 2.757284511 | 3.322183718 | 9.05719237 | 9.72E-07 | 7.59E-05 | 6.023955325 |
| FAM224A | 3.068429978 | 12.43957582 | 9.048807419 | 9.82E-07 | 7.59E-05 | 6.013864903 |
| AP002884.1 | -1.436659153 | 5.386826926 | -8.96705044 | 1.08E-06 | 8.16E-05 | 5.915074892 |
| AL136307.1 | -2.604659614 | 3.519937307 | -8.813926312 | 1.30E-06 | 9.66E-05 | 5.72805866 |
| LINC02273 | -2.176507408 | 7.607290567 | -8.806555422 | 1.31E-06 | 9.66E-05 | 5.718990205 |
| AL606970.2 | 1.368987946 | 7.611671125 | 8.773405436 | 1.36E-06 | 9.94E-05 | 5.678129913 |
| AC087386.1 | 2.992355561 | 5.321710387 | 8.753108117 | 1.40E-06 | 9.96E-05 | 5.653050431 |
| LINC01079 | 1.678029001 | 6.950149506 | 8.709827455 | 1.47E-06 | 0.000103134 | 5.599416671 |
| AL133444.1 | -3.492039119 | 7.712942791 | -8.70639617 | 1.48E-06 | 0.000103134 | 5.595155488 |
| AC090844.3 | -1.342651188 | 5.962763033 | -8.694863393 | 1.50E-06 | 0.000103473 | 5.580823538 |
| AC108136.1 | -4.088140766 | 6.877232582 | -8.664319024 | 1.56E-06 | 0.000106253 | 5.542792288 |
| MIR17HG | 3.109082392 | 6.03723322 | 8.608154912 | 1.67E-06 | 0.000112595 | 5.472582623 |
| LINC01270 | -1.106681157 | 4.746759023 | -8.581025909 | 1.72E-06 | 0.000115194 | 5.4385392 |
| LINC01934 | -1.911105085 | 5.259518278 | -8.548861401 | 1.79E-06 | 0.000118606 | 5.398066664 |
| LINC02356 | -4.633544344 | 7.512902998 | -8.508185834 | 1.88E-06 | 0.000123433 | 5.346712816 |
| LINC00877 | -2.337151704 | 4.595298055 | -8.475058988 | 1.96E-06 | 0.000127297 | 5.304747003 |
| AC097375.1 | -1.79966337 | 3.10830797 | -8.454751049 | 2.01E-06 | 0.000127988 | 5.278957028 |
| SNHG4 | 2.186199315 | 4.124699664 | 8.454673669 | 2.01E-06 | 0.000127988 | 5.278858667 |
| AL627443.1 | 2.331790002 | 6.796687465 | 8.415112377 | 2.11E-06 | 0.000132107 | 5.228478672 |
| AC012236.1 | -1.306008463 | 3.566660032 | -8.4134814 | 2.12E-06 | 0.000132107 | 5.226397727 |
| PRR31 | 1.558810036 | 2.650481911 | 8.396267218 | 2.16E-06 | 0.00013368 | 5.204415218 |
| LINC01259 | -3.467174354 | 4.428517756 | -8.381706706 | 2.20E-06 | 0.000134841 | 5.185794145 |
| AL138963.1 | 3.04340488 | 3.414978885 | 8.336438535 | 2.33E-06 | 0.000141348 | 5.127741525 |
| AP001208.2 | 2.658499874 | 8.319139418 | 8.304159813 | 2.43E-06 | 0.000145472 | 5.086198113 |
| LINC00266-1 | -1.360709996 | 7.075769493 | -8.29870373 | 2.44E-06 | 0.000145472 | 5.079163751 |
| CERNA1 | -2.5760606 | 4.421603276 | -8.275359966 | 2.52E-06 | 0.000147463 | 5.049027196 |
| CU638689.5 | 3.028785952 | 3.591202407 | 8.273411805 | 2.52E-06 | 0.000147463 | 5.046509195 |
| FILNC1 | -1.525933933 | 3.721229844 | -8.250534896 | 2.60E-06 | 0.000149086 | 5.016906738 |
| SPATA41 | -1.333185276 | 4.703783465 | -8.243071422 | 2.62E-06 | 0.000149175 | 5.00723551 |
| AC114546.1 | 2.553775362 | 3.526642636 | 8.230059648 | 2.66E-06 | 0.000150326 | 4.990358749 |
| AC244131.2 | 2.946509169 | 3.738931756 | 8.156519235 | 2.92E-06 | 0.000160813 | 4.894590411 |
| SMIM25 | -4.725026773 | 6.211563149 | -8.156487951 | 2.92E-06 | 0.000160813 | 4.894549532 |
| SMILR | -2.750957344 | 5.07635188 | -8.102716703 | 3.13E-06 | 0.000170766 | 4.824110701 |
| AC002511.1 | 1.099046533 | 5.292566547 | 8.029537752 | 3.44E-06 | 0.000185983 | 4.727681278 |
| LINC01476 | -1.179231032 | 7.523920314 | -7.981133867 | 3.66E-06 | 0.000196309 | 4.663536964 |
| AL353780.1 | 2.645599923 | 6.503172476 | 7.966631948 | 3.73E-06 | 0.000198381 | 4.64426286 |
| AL121894.2 | -1.419893687 | 5.066572807 | -7.95828213 | 3.77E-06 | 0.000198903 | 4.633153537 |
| AC027031.2 | -1.684392183 | 4.2587719 | -7.937118282 | 3.87E-06 | 0.000202779 | 4.604956596 |
| AC092660.1 | 1.927725154 | 2.828095401 | 7.896480875 | 4.08E-06 | 0.000212051 | 4.55065865 |
| AC084032.1 | -1.742415995 | 4.013482728 | -7.889131861 | 4.12E-06 | 0.000212388 | 4.540817247 |
| LINC02361 | -2.836507092 | 9.089181836 | -7.880903888 | 4.17E-06 | 0.000212395 | 4.52979079 |
| AL590068.2 | 2.497449508 | 5.961447766 | 7.877011952 | 4.19E-06 | 0.000212395 | 4.524572189 |
| AL445471.2 | -2.474315314 | 3.079974161 | -7.849007738 | 4.34E-06 | 0.000218587 | 4.48696616 |
| LINC01046 | 2.166618827 | 2.840844556 | 7.819530097 | 4.51E-06 | 0.000225228 | 4.447275297 |
| AC020743.3 | 1.195296384 | 2.476544877 | 7.814357506 | 4.55E-06 | 0.000225228 | 4.44029928 |
| AC127024.2 | 3.330331974 | 5.530386464 | 7.797696317 | 4.65E-06 | 0.000226739 | 4.417806271 |
| AL353804.1 | 1.989370424 | 5.427446811 | 7.755048475 | 4.91E-06 | 0.000238021 | 4.360071112 |
| LINC01127 | -2.260261503 | 4.00923092 | -7.746336738 | 4.97E-06 | 0.000238985 | 4.34824918 |
| AC104964.3 | 2.159422004 | 4.792734608 | 7.692906005 | 5.33E-06 | 0.000253378 | 4.275532368 |
| AC010982.1 | 1.18779789 | 5.661156023 | 7.690934176 | 5.35E-06 | 0.000253378 | 4.272841851 |
| TRIM52-AS1 | -1.163591721 | 9.803564251 | -7.661468477 | 5.56E-06 | 0.000261565 | 4.232577496 |
| LINC02482 | -2.39899894 | 7.705907479 | -7.65531991 | 5.61E-06 | 0.000261812 | 4.224161608 |
| AC234772.2 | 2.964989676 | 8.395311335 | 7.64473569 | 5.69E-06 | 0.000263625 | 4.209663065 |
| AC010737.1 | -2.504533998 | 3.513905693 | -7.609128411 | 5.96E-06 | 0.000272535 | 4.160781924 |
| AC007064.2 | 2.373946407 | 5.535933191 | 7.593164429 | 6.09E-06 | 0.000276459 | 4.138813961 |
| AC103808.2 | 1.24747622 | 6.900476592 | 7.554845529 | 6.41E-06 | 0.000286991 | 4.085949583 |
| AC139099.2 | 2.516168554 | 7.461750024 | 7.541494562 | 6.53E-06 | 0.000287677 | 4.06748621 |
| AL606491.1 | -1.905847505 | 6.634714257 | -7.539226905 | 6.55E-06 | 0.000287677 | 4.064347925 |
| AP001172.2 | 1.651611981 | 3.288189354 | 7.531631455 | 6.61E-06 | 0.000287677 | 4.05383148 |
| AP001033.1 | -2.286307504 | 5.595070866 | -7.52578911 | 6.67E-06 | 0.000287677 | 4.045737256 |
| AC024909.1 | -2.267053836 | 3.558608818 | -7.522326531 | 6.70E-06 | 0.000287677 | 4.040937969 |
| AL359881.2 | 1.819858119 | 5.210872619 | 7.520865666 | 6.71E-06 | 0.000287677 | 4.038912681 |
| AC026904.1 | -2.326401846 | 2.755336792 | -7.517957678 | 6.74E-06 | 0.000287677 | 4.034880333 |
| AC245128.3 | -4.763503204 | 8.304238858 | -7.501810703 | 6.88E-06 | 0.000292076 | 4.012470252 |
| AC009495.3 | -3.270001362 | 4.28065966 | -7.472653069 | 7.16E-06 | 0.000301818 | 3.971917152 |
| LINC01191 | -2.095125953 | 3.255097336 | -7.401955811 | 7.88E-06 | 0.000324845 | 3.873129724 |
| HAR1B | -3.047494204 | 4.083117742 | -7.399464502 | 7.91E-06 | 0.000324845 | 3.869636621 |
| AL162408.1 | 3.265752979 | 3.698009778 | 7.385832288 | 8.06E-06 | 0.000328843 | 3.850508273 |
| AC011445.2 | -2.062016466 | 10.54716882 | -7.379846835 | 8.12E-06 | 0.000329462 | 3.842101948 |
| AL356310.1 | 2.033180984 | 3.723048994 | 7.364427635 | 8.29E-06 | 0.000334368 | 3.820424634 |
| AL136115.2 | 1.898444777 | 5.912720726 | 7.357756361 | 8.37E-06 | 0.000335114 | 3.811036036 |
| LINC02397 | -1.848170427 | 3.542453261 | -7.353747152 | 8.42E-06 | 0.000335114 | 3.805390992 |
| AC017071.1 | 2.412765368 | 3.169598494 | 7.347970012 | 8.48E-06 | 0.000335708 | 3.797252945 |
| AC239800.2 | -1.460254512 | 13.39983469 | -7.308428947 | 8.95E-06 | 0.000352222 | 3.741434776 |
| AL353611.1 | -2.631763079 | 3.010572813 | -7.301197715 | 9.04E-06 | 0.000353589 | 3.731204477 |
| AP001257.1 | -3.669693632 | 4.499396323 | -7.2883209 | 9.20E-06 | 0.000357738 | 3.712970032 |
| MDS2 | -3.180833652 | 5.516318574 | -7.275672408 | 9.37E-06 | 0.000361842 | 3.695037535 |
| AC004223.2 | 1.908749137 | 4.720646485 | 7.260798434 | 9.56E-06 | 0.000365821 | 3.673922714 |
| AL136141.1 | -2.587544899 | 4.925303204 | -7.259121002 | 9.58E-06 | 0.000365821 | 3.671539621 |
| AC010445.1 | 1.140040461 | 3.305146082 | 7.21204693 | 1.02E-05 | 0.000388086 | 3.604509942 |
| AP002793.1 | 1.027787148 | 5.855157666 | 7.179637512 | 1.07E-05 | 0.000403539 | 3.558189962 |
| AC100810.3 | -2.311380107 | 3.346153266 | -7.136772412 | 1.13E-05 | 0.000425828 | 3.496710911 |
| FLJ42351 | -2.019565784 | 5.386250881 | -7.111390667 | 1.18E-05 | 0.000438626 | 3.460191165 |
| NUP50-DT | -1.676983532 | 6.208354301 | -7.100182925 | 1.19E-05 | 0.000442997 | 3.444037702 |
| LINC02458 | -2.346979431 | 4.006957215 | -7.093857384 | 1.20E-05 | 0.00044439 | 3.434913385 |
| LINC00997 | 2.161029948 | 4.467472709 | 7.079208035 | 1.23E-05 | 0.000451025 | 3.413761657 |
| AL357558.1 | 1.392054441 | 5.988417771 | 7.056958343 | 1.27E-05 | 0.000461009 | 3.381580711 |
| AL691403.1 | 2.438884123 | 3.298634334 | 7.055558872 | 1.27E-05 | 0.000461009 | 3.379554346 |
| LINC02077 | 2.136253574 | 2.853709971 | 7.046410447 | 1.29E-05 | 0.000462847 | 3.366301369 |
| LINC00665 | 1.091360537 | 6.744526628 | 7.044804283 | 1.29E-05 | 0.000462847 | 3.363973414 |
| LINC02396 | 1.231122994 | 3.967603455 | 7.035179109 | 1.31E-05 | 0.000464948 | 3.350015493 |
| LINC00273 | 1.316458413 | 7.748592838 | 7.033755803 | 1.31E-05 | 0.000464948 | 3.347950427 |
| LINC00957 | -1.735135919 | 5.939533906 | -7.002914402 | 1.37E-05 | 0.000477431 | 3.303135481 |
| LINC002481 | -2.898368633 | 5.405257735 | -7.001270407 | 1.37E-05 | 0.000477431 | 3.300743013 |
| C16orf82 | -1.262127428 | 4.478734848 | -6.999131029 | 1.38E-05 | 0.000477431 | 3.297629075 |
| AC011840.3 | -2.589130135 | 5.411359721 | -6.993394283 | 1.39E-05 | 0.000477431 | 3.289275982 |
| AC097110.1 | 1.042420682 | 4.757203795 | 6.992022625 | 1.39E-05 | 0.000477431 | 3.287278093 |
| LINC02503 | 1.802186934 | 3.964147169 | 6.980384982 | 1.41E-05 | 0.000480255 | 3.27031701 |
| LINC00886 | -1.648078561 | 3.603800204 | -6.973877217 | 1.43E-05 | 0.000482054 | 3.260824362 |
| LINC01976 | -1.248244331 | 4.286260761 | -6.970365344 | 1.43E-05 | 0.000482054 | 3.255699327 |
| AC126177.4 | -1.137993518 | 8.992824605 | -6.961613644 | 1.45E-05 | 0.00048554 | 3.242920282 |
| AC108488.1 | -1.075675847 | 3.879792253 | -6.923055444 | 1.53E-05 | 0.000510176 | 3.186494308 |
| AC147067.1 | -2.029008792 | 7.666556076 | -6.902915723 | 1.58E-05 | 0.000522306 | 3.156941324 |
| AP001781.1 | 1.38923746 | 11.88856145 | 6.89506782 | 1.59E-05 | 0.000525489 | 3.145410335 |
| FIRRE | 2.249241791 | 5.025091317 | 6.878713369 | 1.63E-05 | 0.000532105 | 3.121353556 |
| ZBTB20-AS2 | 2.253502779 | 7.682918524 | 6.872211556 | 1.65E-05 | 0.000532105 | 3.111779474 |
| AC009041.1 | -2.629575957 | 5.681333696 | -6.872173141 | 1.65E-05 | 0.000532105 | 3.111722889 |
| LINC00265 | 1.380479361 | 8.226497638 | 6.867345584 | 1.66E-05 | 0.00053313 | 3.104610422 |
| LINC02453 | -1.975807049 | 5.082887471 | -6.856398216 | 1.69E-05 | 0.000538858 | 3.088469781 |
| LY6E-DT | -2.364234933 | 6.266665309 | -6.84252065 | 1.72E-05 | 0.000546956 | 3.067985295 |
| LINC00299 | -1.04333724 | 6.199094924 | -6.829756271 | 1.75E-05 | 0.00055432 | 3.049120652 |
| LINC01269 | -1.226858438 | 4.340631313 | -6.81471239 | 1.79E-05 | 0.000560025 | 3.026858391 |
| AC079209.1 | -1.854820357 | 4.6844461 | -6.812468053 | 1.79E-05 | 0.000560025 | 3.023534508 |
| AC018521.5 | -1.839836321 | 7.10051225 | -6.799489326 | 1.83E-05 | 0.000567821 | 3.004299318 |
| AC007834.1 | 1.451104293 | 4.343382073 | 6.77687556 | 1.89E-05 | 0.000583782 | 2.970729109 |
| AL035563.1 | -2.730815998 | 7.549093658 | -6.768756357 | 1.91E-05 | 0.000587843 | 2.958658945 |
| AC109826.1 | -2.113781076 | 4.90203894 | -6.750829961 | 1.96E-05 | 0.000600378 | 2.931977043 |
| AC108062.1 | 1.832457814 | 4.304432587 | 6.72271428 | 2.04E-05 | 0.000622314 | 2.890039944 |
| LINC02322 | -2.069484166 | 3.817761279 | -6.717909249 | 2.06E-05 | 0.000623725 | 2.882861877 |
| AC090192.2 | -1.264676825 | 4.947544121 | -6.70331654 | 2.10E-05 | 0.000634074 | 2.86104277 |
| AC087463.1 | 1.280132165 | 2.235721336 | 6.695112621 | 2.13E-05 | 0.000638684 | 2.848763283 |
| AC040963.1 | 1.016611271 | 3.208857688 | 6.613542371 | 2.39E-05 | 0.000706071 | 2.726162711 |
| AC090503.2 | -1.300428343 | 3.554998257 | -6.606229403 | 2.42E-05 | 0.000710423 | 2.715126136 |
| AP005482.1 | -1.875395576 | 5.0120487 | -6.59702207 | 2.45E-05 | 0.000716805 | 2.701220046 |
| AL161935.3 | -2.629775675 | 4.40244526 | -6.586557402 | 2.49E-05 | 0.000724599 | 2.685400637 |
| AC093627.6 | -2.579153908 | 3.656354254 | -6.575584821 | 2.53E-05 | 0.000733049 | 2.668797036 |
| AC007029.1 | -2.033761092 | 3.588565235 | -6.561593438 | 2.58E-05 | 0.000744915 | 2.647601077 |
| LINC02197 | 1.568091977 | 3.604160136 | 6.540155198 | 2.66E-05 | 0.000765337 | 2.61507068 |
| LINC02298 | -1.411855535 | 6.120847833 | -6.459137569 | 3.00E-05 | 0.000846455 | 2.491554514 |
| AC092650.1 | -1.081616836 | 2.136507822 | -6.45704427 | 3.01E-05 | 0.000846455 | 2.48835098 |
| C8orf31 | -3.947504974 | 5.573682362 | -6.439235546 | 3.09E-05 | 0.00086535 | 2.461072103 |
| MIR200CHG | -1.61219897 | 3.223480864 | -6.426658631 | 3.15E-05 | 0.000870604 | 2.441780359 |
| AL031281.3 | -1.4748243 | 7.834292752 | -6.424331126 | 3.16E-05 | 0.000870604 | 2.438207763 |
| AC092127.1 | 2.036860441 | 6.040513468 | 6.421241113 | 3.18E-05 | 0.000870604 | 2.433463583 |
| AC073188.4 | 1.128181545 | 2.414470549 | 6.420796967 | 3.18E-05 | 0.000870604 | 2.432781565 |
| AC126177.2 | -1.301667527 | 5.835980239 | -6.382667633 | 3.36E-05 | 0.000912538 | 2.374127854 |
| DISC1FP1 | -1.044146725 | 3.291323419 | -6.379898198 | 3.38E-05 | 0.000912538 | 2.369859722 |
| AC105415.1 | -1.426810997 | 3.299606109 | -6.377255745 | 3.39E-05 | 0.000912538 | 2.365786284 |
| AP005271.1 | -1.41176439 | 4.413295128 | -6.373749602 | 3.41E-05 | 0.000912538 | 2.360379921 |
| LINC02107 | -2.196560154 | 3.152290003 | -6.372418718 | 3.42E-05 | 0.000912538 | 2.358327287 |
| AC005498.2 | -2.248420204 | 5.31768685 | -6.356378826 | 3.50E-05 | 0.000930537 | 2.333569228 |
| LINC02595 | 3.082604767 | 3.353035175 | 6.35084345 | 3.53E-05 | 0.000930537 | 2.325016808 |
| AC136944.2 | 1.042654403 | 7.304961291 | 6.348418977 | 3.54E-05 | 0.000930537 | 2.321269523 |
| SUCLG2-AS1 | -1.669500924 | 3.102551069 | -6.325674194 | 3.66E-05 | 0.000956267 | 2.286074717 |
| LINC01703 | -1.310410357 | 5.271997337 | -6.296310963 | 3.83E-05 | 0.000994076 | 2.240530912 |
| AC135178.5 | 1.699248144 | 5.508658755 | 6.256223865 | 4.07E-05 | 0.001051714 | 2.178157625 |
| AC017104.1 | -1.404039953 | 8.364891176 | -6.242126505 | 4.15E-05 | 0.001070068 | 2.156169037 |
| SNHG9 | -1.046866285 | 12.47156161 | -6.235827469 | 4.19E-05 | 0.001072482 | 2.146334945 |
| AL391244.3 | 1.780285759 | 10.62183233 | 6.234110366 | 4.20E-05 | 0.001072482 | 2.143653222 |
| AP003548.1 | 1.180036922 | 4.446311261 | 6.232802376 | 4.21E-05 | 0.001072482 | 2.14161016 |
| AP001109.1 | 2.261861298 | 4.688948867 | 6.22882473 | 4.24E-05 | 0.001074736 | 2.13539565 |
| LINC00843 | 1.93577647 | 6.946972981 | 6.224477417 | 4.27E-05 | 0.001075299 | 2.128601034 |
| AC020656.2 | 1.882741719 | 14.31016213 | 6.196916831 | 4.45E-05 | 0.001114874 | 2.085463212 |
| AC034199.1 | -1.533536022 | 5.399520084 | -6.170424514 | 4.63E-05 | 0.001156137 | 2.043896274 |
| AC119428.2 | -2.077112007 | 4.559920774 | -6.167916741 | 4.65E-05 | 0.001156137 | 2.039956392 |
| LINC00408 | 1.473558481 | 2.467657778 | 6.142775613 | 4.83E-05 | 0.001187589 | 2.00040882 |
| AC104777.2 | 1.81371881 | 3.392233782 | 6.136012838 | 4.88E-05 | 0.001195367 | 1.989755564 |
| AC110079.2 | -1.875776936 | 3.564948444 | -6.128182221 | 4.94E-05 | 0.001200272 | 1.977412076 |
| SNHG16 | 1.040795439 | 8.478997438 | 6.117614518 | 5.02E-05 | 0.001211125 | 1.960740337 |
| AC136475.5 | -2.213100138 | 5.422196155 | -6.099769084 | 5.16E-05 | 0.001235433 | 1.932551291 |
| AL512631.1 | -1.463548561 | 4.814029972 | -6.093512917 | 5.21E-05 | 0.00123818 | 1.922658243 |
| MALAT1 | 1.945201766 | 11.95330647 | 6.087309148 | 5.26E-05 | 0.001241706 | 1.912842583 |
| SND1-IT1 | 1.620827673 | 7.565025837 | 6.084519029 | 5.28E-05 | 0.001241706 | 1.908426256 |
| AC009154.1 | 1.449017754 | 2.555532906 | 6.082119737 | 5.30E-05 | 0.001241779 | 1.904627667 |
| AC006504.1 | 1.763051048 | 6.673475695 | 6.061979875 | 5.47E-05 | 0.001276128 | 1.872709857 |
| LINC02247 | -2.192511172 | 3.603340292 | -6.04541186 | 5.61E-05 | 0.001299675 | 1.846409697 |
| LINC01771 | -1.540116485 | 4.216855528 | -6.042854896 | 5.63E-05 | 0.001300151 | 1.842347296 |
| AC104986.2 | -1.449579951 | 9.053555184 | -6.029790137 | 5.74E-05 | 0.001321847 | 1.821576095 |
| AC068756.1 | 1.162548686 | 5.60642366 | 6.022358813 | 5.81E-05 | 0.001332338 | 1.809750514 |
| AC010183.2 | -1.471557558 | 2.964078978 | -6.017673733 | 5.85E-05 | 0.001335673 | 1.802291055 |
| AC106738.2 | -1.007145332 | 6.506254377 | -6.00580723 | 5.96E-05 | 0.001352437 | 1.783383625 |
| LINC02175 | 2.764474059 | 5.344594308 | 5.993011847 | 6.08E-05 | 0.001356952 | 1.762973833 |
| LINC00944 | -2.721182472 | 5.09332224 | -5.991950101 | 6.09E-05 | 0.001356952 | 1.761279211 |
| LINC02021 | 1.452366141 | 5.661926796 | 5.990314874 | 6.10E-05 | 0.001356952 | 1.758668961 |
| AC090457.1 | 1.161976032 | 2.874067303 | 5.990201535 | 6.10E-05 | 0.001356952 | 1.758488028 |
| GTSCR1 | -2.217014769 | 6.471285997 | -5.984520236 | 6.16E-05 | 0.001364247 | 1.749416154 |
| SERPINB9P1 | -1.474147437 | 6.197199166 | -5.967873737 | 6.32E-05 | 0.001389773 | 1.722808789 |
| AC009812.4 | 1.976721054 | 5.534697436 | 5.965953886 | 6.34E-05 | 0.001389773 | 1.719737621 |
| PSMB8-AS1 | -2.385118512 | 9.945553138 | -5.956921659 | 6.43E-05 | 0.001404609 | 1.705281857 |
| SOD2-OT1 | 1.235021717 | 5.499366584 | 5.950655387 | 6.49E-05 | 0.00141204 | 1.695246123 |
| AC096711.2 | 2.38900677 | 6.673915405 | 5.949188743 | 6.50E-05 | 0.00141204 | 1.692896421 |
| AL513314.2 | 1.720051264 | 6.153870567 | 5.931295815 | 6.69E-05 | 0.001441168 | 1.664205742 |
| LINC00920 | -2.556914836 | 3.496727044 | -5.927473899 | 6.73E-05 | 0.001441168 | 1.658071562 |
| LINC01431 | -1.160869448 | 6.965476437 | -5.920019014 | 6.81E-05 | 0.001441726 | 1.646100517 |
| AL022310.1 | 1.67875874 | 2.643772649 | 5.916118521 | 6.85E-05 | 0.001441726 | 1.639833975 |
| AC006449.6 | -2.01724619 | 6.939099211 | -5.915606519 | 6.85E-05 | 0.001441726 | 1.639011233 |
| AC091488.1 | -3.379092757 | 6.261105427 | -5.912535772 | 6.89E-05 | 0.001441726 | 1.634076027 |
| AC091057.4 | 2.893139964 | 4.718136048 | 5.90313939 | 6.99E-05 | 0.001458263 | 1.618966179 |
| AC073321.1 | -1.080393253 | 8.060110377 | -5.880669987 | 7.24E-05 | 0.001501862 | 1.582783633 |
| AC141586.2 | 1.572145159 | 5.064007115 | 5.873182379 | 7.32E-05 | 0.001513398 | 1.570710456 |
| AL390036.1 | -2.864422368 | 5.930936073 | -5.867515224 | 7.39E-05 | 0.001522005 | 1.561567345 |
| LINC01891 | -1.102508522 | 2.36637128 | -5.862021867 | 7.45E-05 | 0.001530268 | 1.5527003 |
| AC108693.2 | -1.143618164 | 4.767984847 | -5.85942168 | 7.48E-05 | 0.001531653 | 1.548501746 |
| LINC01857 | -3.137247174 | 7.273270074 | -5.818968547 | 7.97E-05 | 0.0016216 | 1.483058549 |
| AP005264.1 | 1.28720677 | 4.00016944 | 5.814301043 | 8.03E-05 | 0.001628416 | 1.475492804 |
| LINC02574 | -2.381267601 | 4.110044516 | -5.791698532 | 8.32E-05 | 0.001666455 | 1.438811983 |
| LINC00504 | -1.238307538 | 3.519270832 | -5.784884663 | 8.41E-05 | 0.001679232 | 1.427739852 |
| AC084033.3 | 2.897684888 | 10.05727301 | 5.779785862 | 8.48E-05 | 0.00168454 | 1.419450319 |
| AC092112.1 | -2.385537656 | 4.170718721 | -5.773952147 | 8.55E-05 | 0.001686292 | 1.40996148 |
| AC020909.2 | -1.779077183 | 3.639893462 | -5.772524333 | 8.57E-05 | 0.001686292 | 1.407638336 |
| LINC01215 | -1.074276878 | 7.767758415 | -5.7390107 | 9.04E-05 | 0.00175889 | 1.353026986 |
| AC123595.1 | -1.292707798 | 5.468601213 | -5.73715702 | 9.07E-05 | 0.00175889 | 1.350001746 |
| LINC02391 | -1.369365003 | 6.147974646 | -5.732121281 | 9.14E-05 | 0.001765232 | 1.341780887 |
| AC092902.2 | 1.170079457 | 5.324072774 | 5.702663939 | 9.57E-05 | 0.001844019 | 1.293620176 |
| AC121764.3 | 1.123409067 | 4.373149109 | 5.69414198 | 9.70E-05 | 0.001852617 | 1.279664599 |
| AP000688.2 | 2.963369823 | 4.412328247 | 5.681084372 | 9.91E-05 | 0.001885872 | 1.258261631 |
| AC010754.1 | -1.255243174 | 3.206488054 | -5.676330361 | 9.98E-05 | 0.001889089 | 1.250463294 |
| AC106892.1 | -1.067064717 | 5.368420754 | -5.65391197 | 0.000103448 | 0.001943839 | 1.213646069 |
| AL392172.1 | -1.793994911 | 7.984065277 | -5.652893918 | 0.000103616 | 0.001943839 | 1.211972471 |
| AC106900.1 | -1.845105525 | 5.074285516 | -5.646586918 | 0.000104662 | 0.001957806 | 1.201601012 |
| AL031056.1 | -1.389220155 | 4.432014644 | -5.637221978 | 0.000106236 | 0.001981537 | 1.186190675 |
| LINC02413 | -1.795350616 | 4.615126977 | -5.630730276 | 0.000107341 | 0.001996336 | 1.175501137 |
| AC011481.1 | 1.290219991 | 10.42475111 | 5.628964371 | 0.000107644 | 0.001996336 | 1.172592293 |
| OSER1-DT | -1.166999021 | 6.403792701 | -5.605219687 | 0.000111805 | 0.002061725 | 1.133437039 |
| AC017002.1 | 2.357493225 | 6.004439155 | 5.58600653 | 0.000115296 | 0.002120079 | 1.101696582 |
| AC026304.1 | -1.584468344 | 6.703562474 | -5.565820149 | 0.000119088 | 0.002169788 | 1.068292774 |
| AC087894.2 | -1.385069765 | 6.766538515 | -5.564039476 | 0.000119429 | 0.002169788 | 1.065343439 |
| LINC01228 | -1.822682468 | 3.528503556 | -5.560470003 | 0.000120115 | 0.002169788 | 1.059429978 |
| AC011840.2 | 1.072320001 | 9.300987251 | 5.558134124 | 0.000120567 | 0.002169788 | 1.05555922 |
| AC025171.4 | 1.596685429 | 6.249164516 | 5.55786773 | 0.000120618 | 0.002169788 | 1.055117734 |
| AL672277.1 | 3.380486185 | 4.55173551 | 5.555858275 | 0.000121008 | 0.002169788 | 1.051787206 |
| AL133279.2 | 1.51406318 | 2.808146117 | 5.543826213 | 0.00012337 | 0.002199989 | 1.031833147 |
| AC011524.1 | 1.194270618 | 4.768190401 | 5.533109067 | 0.000125515 | 0.002232107 | 1.01404277 |
| AL354979.1 | -1.518852255 | 7.169827263 | -5.530506317 | 0.000126042 | 0.002235352 | 1.009719811 |
| LINC01968 | 1.041673445 | 3.398756772 | 5.522207463 | 0.000127737 | 0.002255584 | 0.995929786 |
| AC008555.5 | -2.195152027 | 5.103720749 | -5.521525628 | 0.000127878 | 0.002255584 | 0.994796369 |
| AC006504.5 | 1.133124613 | 5.985853398 | 5.513964101 | 0.000129445 | 0.002277046 | 0.982222487 |
| TP53TG1 | -2.638063213 | 9.944078805 | -5.509378603 | 0.000130406 | 0.002287742 | 0.974593498 |
| AC004951.1 | 1.879006881 | 7.093187078 | 5.505941681 | 0.000131131 | 0.002294257 | 0.968873505 |
| LINC02134 | 1.099699352 | 3.858274048 | 5.471655179 | 0.000138598 | 0.002411907 | 0.911721459 |
| AL928742.1 | -1.371653659 | 5.274424873 | -5.457367197 | 0.000141841 | 0.002461742 | 0.887856772 |
| AL135925.1 | 1.330829812 | 10.95430251 | 5.443548455 | 0.000145054 | 0.002505509 | 0.864748972 |
| AP006621.3 | -1.12777769 | 8.337663589 | -5.44320367 | 0.000145135 | 0.002505509 | 0.864172082 |
| C15orf54 | -3.127098898 | 3.32840467 | -5.432663363 | 0.000147638 | 0.002535241 | 0.846528263 |
| AL592494.1 | -1.975937118 | 3.627247566 | -5.425386556 | 0.000149393 | 0.002558606 | 0.83433839 |
| AC092902.3 | -1.638036189 | 6.763657926 | -5.399063727 | 0.000155927 | 0.002639646 | 0.790182275 |
| LINC01948 | -1.020289688 | 3.661805614 | -5.398152157 | 0.000156158 | 0.002639646 | 0.788651421 |
| AL365203.2 | -3.219846814 | 5.253519259 | -5.384043174 | 0.000159789 | 0.002685819 | 0.764942809 |
| AC087318.1 | -1.153542002 | 2.525569352 | -5.381148692 | 0.000160545 | 0.002685819 | 0.760075564 |
| AC107373.1 | 1.743658657 | 2.588399258 | 5.377675675 | 0.000161457 | 0.00269413 | 0.754233962 |
| AL590648.3 | -1.028789165 | 6.224561067 | -5.372161251 | 0.000162916 | 0.002711505 | 0.744955317 |
| FGF14-AS2 | -1.845013068 | 8.329580322 | -5.369230579 | 0.000163697 | 0.002717537 | 0.740022432 |
| URB1-AS1 | -2.045713946 | 7.718480501 | -5.367528945 | 0.000164152 | 0.002718008 | 0.737157713 |
| LINC01648 | -1.196530184 | 3.234924062 | -5.36521107 | 0.000164775 | 0.002718008 | 0.733254907 |
| LINC02256 | 2.709209475 | 5.56576033 | 5.359916282 | 0.000166206 | 0.002731241 | 0.724336857 |
| AC005546.1 | 1.964211008 | 5.342285819 | 5.357242202 | 0.000166933 | 0.002736272 | 0.719831423 |
| AC064805.1 | -1.42474087 | 5.984755158 | -5.333537236 | 0.000173532 | 0.002830146 | 0.679849244 |
| AL513348.1 | 1.476067275 | 2.843686077 | 5.328077845 | 0.000175091 | 0.002848404 | 0.670630238 |
| AL158071.3 | -2.625983133 | 8.10392686 | -5.316967973 | 0.000178308 | 0.002883706 | 0.651856978 |
| NEAT1 | 2.606397805 | 9.362672965 | 5.269453307 | 0.000192784 | 0.003059556 | 0.571378031 |
| AC005180.2 | 1.039724715 | 6.114762121 | 5.257820382 | 0.000196513 | 0.003103563 | 0.551627849 |
| LINC01355 | 1.280310178 | 3.627074341 | 5.24584578 | 0.000200431 | 0.003157758 | 0.531278457 |
| LINC01911 | 5.262047179 | 9.944931066 | 5.237344183 | 0.000203262 | 0.003186897 | 0.516819267 |
| AL365361.1 | -2.767831802 | 5.91133223 | -5.232600764 | 0.00020486 | 0.003204213 | 0.508747604 |
| AC005261.1 | 1.62525489 | 8.247466111 | 5.226390912 | 0.000206972 | 0.003229466 | 0.498175996 |
| AC103831.1 | -1.417349356 | 2.311909313 | -5.215249563 | 0.000210819 | 0.003281603 | 0.479196033 |
| LINC01504 | -2.247663048 | 5.413346166 | -5.212085671 | 0.000211925 | 0.003290929 | 0.473803108 |
| AC091563.1 | 2.182085121 | 8.727352666 | 5.205491354 | 0.00021425 | 0.003303946 | 0.462558627 |
| LINC01353 | -1.694808439 | 6.168501208 | -5.204390442 | 0.000214641 | 0.003303946 | 0.460680808 |
| AL158210.1 | -2.871963066 | 5.785972993 | -5.203852381 | 0.000214832 | 0.003303946 | 0.459762981 |
| AC024579.1 | 1.489022792 | 3.815784172 | 5.200276901 | 0.000216108 | 0.003304705 | 0.453662926 |
| AC012055.2 | 1.156170379 | 4.011391482 | 5.199526983 | 0.000216376 | 0.003304705 | 0.452383288 |
| CU634019.4 | 2.235617793 | 8.520890984 | 5.174386186 | 0.000225585 | 0.003413216 | 0.409440204 |
| AC017053.1 | 1.203426191 | 10.1949593 | 5.165159285 | 0.000229067 | 0.00345784 | 0.393658509 |
| LINC01282 | -1.069583123 | 4.545151068 | -5.124901846 | 0.000244932 | 0.00366326 | 0.324669595 |
| AC008429.3 | 1.504863951 | 3.068493117 | 5.122680629 | 0.000245841 | 0.003668393 | 0.320856849 |
| AC125603.2 | 4.487292149 | 3.88086538 | 5.107218803 | 0.000252262 | 0.003746987 | 0.294298356 |
| AL158152.1 | 1.49828999 | 6.779373115 | 5.100601681 | 0.000255064 | 0.003771343 | 0.2829226 |
| AC100791.1 | -1.064560197 | 4.736654377 | -5.092601954 | 0.000258495 | 0.003813386 | 0.269162247 |
| AL390728.6 | 1.6295764 | 10.17371648 | 5.08519081 | 0.000261716 | 0.003852158 | 0.256406801 |
| CRNDE | 3.215975581 | 5.19112942 | 5.077365768 | 0.000265164 | 0.00389407 | 0.242931153 |
| LINC01761 | 1.34030888 | 9.987715424 | 5.070331055 | 0.000268304 | 0.003914634 | 0.230809685 |
| AL160313.1 | -2.375646991 | 5.13414613 | -5.070175873 | 0.000268373 | 0.003914634 | 0.230542219 |
| LINC02553 | -1.499658541 | 5.38364352 | -5.062076998 | 0.000272038 | 0.003959186 | 0.216578903 |
| LINC01179 | -1.818764823 | 3.732109014 | -5.056476937 | 0.000274602 | 0.003987567 | 0.206918788 |
| AC005606.2 | -1.009268774 | 5.063229014 | -5.044318813 | 0.000280257 | 0.004051558 | 0.1859319 |
| AC008467.1 | -1.187051013 | 2.674365654 | -5.037526895 | 0.000283469 | 0.004088889 | 0.174199559 |
| AC073172.1 | -2.654484922 | 2.999222524 | -5.029381297 | 0.000287373 | 0.004126852 | 0.160120953 |
| AC087222.1 | 2.027942042 | 5.319810087 | 5.022247024 | 0.000290838 | 0.004167392 | 0.147783207 |
| AC244502.3 | 3.619480548 | 5.865779139 | 5.016724209 | 0.00029355 | 0.00418777 | 0.138227722 |
| AC008549.2 | -1.863556617 | 3.094588618 | -5.007509419 | 0.000298135 | 0.004243852 | 0.12227564 |
| AL021368.2 | 1.337027349 | 7.861547232 | 5.005645072 | 0.000299072 | 0.004247871 | 0.119046861 |
| AC021752.1 | 1.87806035 | 9.044944404 | 5.004210151 | 0.000299795 | 0.004248844 | 0.116561479 |
| AC245884.8 | 1.757807427 | 6.02509589 | 4.991493839 | 0.000306284 | 0.004317153 | 0.094524327 |
| LINC02352 | -1.347129136 | 4.398914092 | -4.990861395 | 0.00030661 | 0.004317153 | 0.093427768 |
| LINC02018 | 1.332209004 | 4.952823508 | 4.971371041 | 0.000316854 | 0.00441779 | 0.059609302 |
| LINC01920 | 1.350644286 | 2.909181846 | 4.970797876 | 0.000317161 | 0.00441779 | 0.058614043 |
| AL158166.2 | -2.36855362 | 3.974587989 | -4.959096102 | 0.000323489 | 0.004474354 | 0.038285556 |
| LINC01416 | -1.532999016 | 5.326321391 | -4.950758338 | 0.000328079 | 0.004513964 | 0.023790386 |
| LINC02328 | -1.336495089 | 5.348874485 | -4.940482044 | 0.00033383 | 0.004570207 | 0.005912882 |
| LINC01252 | 1.695327078 | 4.298786096 | 4.939321958 | 0.000334486 | 0.004570207 | 0.003893853 |
| TUSC8 | 2.094895785 | 4.757309692 | 4.937529913 | 0.000335502 | 0.004570207 | 0.000774618 |
| AL390816.1 | 1.238365419 | 4.110484458 | 4.936920169 | 0.000335848 | 0.004570207 | -0.000286796 |
| AC026979.4 | 2.672762556 | 6.982155642 | 4.918341726 | 0.000346582 | 0.00467706 | -0.032649928 |
| BISPR | -1.995337549 | 6.661789088 | -4.90672841 | 0.000353474 | 0.004730727 | -0.052902213 |
| AC007114.1 | -1.579015655 | 5.303421023 | -4.900656845 | 0.000357135 | 0.004760085 | -0.063497106 |
| PCBP1-AS1 | -1.715036946 | 7.202209622 | -4.89666342 | 0.000359564 | 0.004763119 | -0.070468173 |
| AC007878.1 | 1.628215796 | 6.032150433 | 4.892821772 | 0.000361917 | 0.004776198 | -0.077176189 |
| AC022400.5 | 1.183128535 | 7.201075908 | 4.892649509 | 0.000362023 | 0.004776198 | -0.077477026 |
| Z97832.2 | 1.463085336 | 6.134838694 | 4.88676616 | 0.000365659 | 0.004804638 | -0.087753839 |
| AP000317.1 | -1.119705912 | 3.609088354 | -4.88351702 | 0.000367683 | 0.004821478 | -0.09343118 |
| AC007342.5 | -1.282320204 | 3.250132421 | -4.881800375 | 0.000368758 | 0.004825817 | -0.09643127 |
| LINC01781 | -2.951356463 | 5.538282342 | -4.879196756 | 0.000370393 | 0.004837469 | -0.100982186 |
| AC096666.1 | 1.519085771 | 3.707624507 | 4.86537836 | 0.000379202 | 0.004903193 | -0.125149822 |
| AP003774.1 | -1.523410932 | 5.476288493 | -4.853374935 | 0.000387033 | 0.00498458 | -0.146162521 |
| AC068020.1 | 2.669085442 | 4.796152357 | 4.831289986 | 0.000401884 | 0.005155399 | -0.184870309 |
| PCAT18 | 3.013245902 | 3.084883687 | 4.819575646 | 0.000410003 | 0.005235017 | -0.205426261 |
| AL158175.1 | -1.308838493 | 6.093428804 | -4.8181861 | 0.000410977 | 0.005235017 | -0.207865717 |
| AC140658.2 | 1.833234687 | 4.444896597 | 4.814376239 | 0.000413661 | 0.005235017 | -0.214555438 |
| LINC02219 | -1.083554927 | 6.008214583 | -4.814269956 | 0.000413737 | 0.005235017 | -0.214742086 |
| AL442647.1 | 1.140417173 | 3.775497062 | 4.811415598 | 0.00041576 | 0.005250382 | -0.219755248 |
| AL445193.2 | 1.381340846 | 3.197605209 | 4.80951427 | 0.000417113 | 0.005257245 | -0.223095144 |
| AC104653.1 | -1.583258221 | 5.697052446 | -4.805681578 | 0.000419855 | 0.005281553 | -0.229829044 |
| AC015911.2 | 1.594471956 | 5.10027345 | 4.804543805 | 0.000420673 | 0.005281603 | -0.231828416 |
| AC010551.1 | 2.291727168 | 4.589849655 | 4.772914723 | 0.000444084 | 0.005501042 | -0.28747254 |
| AC004585.1 | -2.612888949 | 6.195073985 | -4.768219121 | 0.000447673 | 0.005534949 | -0.295743758 |
| LINC01336 | -1.581604753 | 2.790682657 | -4.746443869 | 0.000464722 | 0.005713091 | -0.334135336 |
| AC024028.1 | -1.041671087 | 3.21931131 | -4.736320759 | 0.000472878 | 0.005791415 | -0.352002671 |
| AC096759.2 | 1.056139371 | 3.386854984 | 4.733350295 | 0.000475299 | 0.005799186 | -0.357247888 |
| AL731563.3 | 1.913511597 | 3.694699532 | 4.710627246 | 0.000494256 | 0.006001522 | -0.397406907 |
| AC015813.3 | 1.036374885 | 3.013106157 | 4.710157364 | 0.000494656 | 0.006001522 | -0.39823799 |
| AC092368.3 | -2.630732089 | 10.3141378 | -4.695091061 | 0.000507671 | 0.006123677 | -0.424899741 |
| LINC01514 | 1.108060735 | 8.280498456 | 4.693963437 | 0.000508659 | 0.006123677 | -0.426896295 |
| AF165147.1 | -1.228909273 | 3.945439762 | -4.692090246 | 0.000510305 | 0.006123677 | -0.430213273 |
| AC138035.1 | 1.503407409 | 8.06324348 | 4.691999557 | 0.000510385 | 0.006123677 | -0.430373873 |
| AC006357.1 | 1.038076352 | 3.997193025 | 4.676730791 | 0.000524015 | 0.006213862 | -0.45742682 |
| AC017048.1 | 1.084060857 | 2.621754551 | 4.675946902 | 0.000524725 | 0.006213862 | -0.458816442 |
| LINC01474 | -1.507986867 | 3.321596766 | -4.675594348 | 0.000525045 | 0.006213862 | -0.459441448 |
| AC240565.2 | 1.487316797 | 7.869120322 | 4.675026391 | 0.00052556 | 0.006213862 | -0.460448348 |
| AL592164.1 | -1.371749685 | 4.256282995 | -4.65572158 | 0.000543394 | 0.006401397 | -0.494695271 |
| U62317.4 | -1.117499003 | 7.341671809 | -4.649441346 | 0.000549331 | 0.006447932 | -0.505845831 |
| LINC01518 | 1.027251305 | 5.024490676 | 4.647542869 | 0.000551139 | 0.006457477 | -0.50921748 |
| AC068282.1 | -1.104410931 | 4.936486606 | -4.646012837 | 0.0005526 | 0.006462936 | -0.511935084 |
| AL022344.2 | -1.147926129 | 5.898114771 | -4.640847428 | 0.000557565 | 0.006482941 | -0.521111755 |
| AC008892.1 | -1.856744796 | 3.390416817 | -4.639880612 | 0.000558499 | 0.006482941 | -0.522829706 |
| AC106712.1 | -1.378553166 | 3.30716953 | -4.639714034 | 0.00055866 | 0.006482941 | -0.523125714 |
| AC125603.1 | 5.718596367 | 5.311888983 | 4.634766325 | 0.000563469 | 0.006519569 | -0.531919167 |
| TAB2-AS1 | -1.01650553 | 2.034796017 | -4.622855994 | 0.000575223 | 0.006608351 | -0.553098712 |
| LINC01238 | 1.449356473 | 7.234364092 | 4.60817788 | 0.00059006 | 0.006731253 | -0.579222459 |
| AL592431.1 | 1.263198378 | 2.71115602 | 4.604602935 | 0.000593734 | 0.00674944 | -0.585588788 |
| LINC00968 | -1.632867411 | 3.143151996 | -4.602762642 | 0.000595635 | 0.006758505 | -0.588866584 |
| AC010280.3 | -1.496842038 | 7.925061285 | -4.59692774 | 0.000601703 | 0.006792441 | -0.599261833 |
| LINC01545 | 2.121234364 | 2.881070908 | 4.578776901 | 0.000620994 | 0.006961786 | -0.631623377 |
| AC017015.2 | 1.14636527 | 2.87250964 | 4.54341299 | 0.000660455 | 0.007224357 | -0.694780801 |
| AC008269.2 | -1.259500217 | 3.471516992 | -4.542844417 | 0.000661111 | 0.007224357 | -0.69579737 |
| Z97192.2 | 3.567489194 | 3.875702367 | 4.534203468 | 0.000671154 | 0.007297259 | -0.711251185 |
| AC114550.2 | 1.383463391 | 2.582292391 | 4.530017533 | 0.000676077 | 0.007338489 | -0.718740442 |
| AC011478.1 | -1.006495805 | 3.529011754 | -4.52052031 | 0.000687386 | 0.007448782 | -0.735739521 |
| LINC02573 | 4.313496934 | 6.008251475 | 4.514179615 | 0.000695045 | 0.007519231 | -0.747094236 |
| AL513548.4 | 2.574283989 | 4.345801159 | 4.498174701 | 0.000714777 | 0.007669367 | -0.775774827 |
| AC008753.2 | 1.117117489 | 5.984089286 | 4.493605063 | 0.000720517 | 0.00771762 | -0.783968659 |
| LINC02469 | 1.130368 | 5.967520094 | 4.490004311 | 0.000725074 | 0.007740883 | -0.79042677 |
| AL353803.5 | -2.03894052 | 4.009485193 | -4.487180046 | 0.000728669 | 0.007766491 | -0.795493195 |
| AC012531.1 | -2.606037491 | 5.000444489 | -4.483089903 | 0.000733909 | 0.007809513 | -0.802831989 |
| LINC00471 | 1.076828412 | 3.706975894 | 4.433534221 | 0.000800598 | 0.008368252 | -0.891889663 |
| AL359502.1 | 1.092471836 | 2.495195972 | 4.429819418 | 0.000805845 | 0.008408062 | -0.898576059 |
| ERVH48-1 | 1.376787119 | 5.355521079 | 4.415306058 | 0.000826689 | 0.008558272 | -0.924712912 |
| AL121910.1 | -1.126368399 | 3.753585829 | -4.407929827 | 0.000837496 | 0.00864259 | -0.938005001 |
| AL023284.4 | 3.868301371 | 5.539368203 | 4.40111571 | 0.000847611 | 0.008733083 | -0.950289132 |
| AC095032.2 | 1.067336418 | 2.729648705 | 4.399800687 | 0.000849577 | 0.008739472 | -0.952660335 |
| AL121899.1 | -2.050916274 | 5.425912468 | -4.391825812 | 0.000861605 | 0.008829826 | -0.967044142 |
| AC023824.5 | 1.437297533 | 3.576599469 | 4.38131888 | 0.000877723 | 0.008972125 | -0.986004768 |
| AC110769.2 | 1.156906353 | 5.737741077 | 4.374154198 | 0.000888893 | 0.009057777 | -0.998940448 |
| AC027237.2 | 1.040955457 | 4.618522409 | 4.362940011 | 0.000906674 | 0.009193341 | -1.01919781 |
| AC009041.2 | -2.595076698 | 6.564870306 | -4.362448934 | 0.000907461 | 0.009193341 | -1.020085181 |
| AP003733.3 | 1.05656207 | 3.011209074 | 4.355156625 | 0.000919232 | 0.009265112 | -1.033265166 |
| AC018450.1 | -1.605731542 | 7.176501165 | -4.349099472 | 0.000929129 | 0.00933052 | -1.044216782 |
| AC131097.4 | 3.726056992 | 5.762006125 | 4.348549582 | 0.000930033 | 0.00933052 | -1.045211188 |
| PRKCQ-AS1 | -1.404284969 | 7.794259961 | -4.346370602 | 0.000933625 | 0.009352094 | -1.049151896 |
| AC093382.1 | -1.195024973 | 4.662425801 | -4.332092166 | 0.000957516 | 0.009547216 | -1.074986203 |
| AC097713.1 | -1.253569584 | 9.227144081 | -4.32773604 | 0.000964931 | 0.00960639 | -1.082871833 |
| AL954650.1 | -1.263050254 | 4.076389915 | -4.311671581 | 0.000992794 | 0.009808561 | -1.11196835 |
| AL356489.1 | 1.186765428 | 4.134704594 | 4.293125122 | 0.001026003 | 0.010060098 | -1.145591373 |
| AC016383.1 | 1.352192263 | 3.028005849 | 4.28447244 | 0.001041889 | 0.010185088 | -1.161289145 |
| LINC01268 | 3.326369939 | 5.018396018 | 4.268469868 | 0.001071944 | 0.010455001 | -1.19033982 |
| AC006012.1 | 1.016034668 | 2.439062389 | 4.259248027 | 0.001089671 | 0.010572579 | -1.207091833 |
| AC027682.4 | 1.30143258 | 3.109717101 | 4.257863293 | 0.001092359 | 0.01058284 | -1.209607967 |
| AC245088.1 | -1.883079111 | 2.706211842 | -4.255932319 | 0.001096119 | 0.01060344 | -1.213116948 |
| AC025165.5 | 1.14366502 | 4.929303964 | 4.253113699 | 0.001101631 | 0.010625094 | -1.218239583 |
| AC015468.2 | -1.163959274 | 2.933322742 | -4.232190886 | 0.001143458 | 0.010933881 | -1.256288136 |
| LINC01035 | 1.524531921 | 3.534996892 | 4.232045991 | 0.001143754 | 0.010933881 | -1.25655177 |
| FP236383.1 | 2.36832259 | 3.918820803 | 4.228057403 | 0.001151915 | 0.010963457 | -1.26380969 |
| LINC00528 | -1.266981229 | 6.324817571 | -4.220622278 | 0.001167289 | 0.011058515 | -1.277343023 |
| AC009869.1 | 2.111572806 | 4.961650522 | 4.21993675 | 0.001168717 | 0.011058515 | -1.278591064 |
| AL022316.1 | -1.439615315 | 3.289867646 | -4.216356033 | 0.001176206 | 0.011075445 | -1.285110646 |
| LINC00384 | -1.190041404 | 3.402089866 | -4.215820156 | 0.001177331 | 0.011075445 | -1.286086441 |
| SNHG25 | 1.377629747 | 8.864729368 | 4.207579006 | 0.001194775 | 0.011207058 | -1.301096255 |
| C22orf34 | 1.553003986 | 7.681105129 | 4.197617335 | 0.001216218 | 0.011344681 | -1.319247773 |
| AC012615.1 | -1.383536184 | 11.64589315 | -4.171840053 | 0.001273576 | 0.011776044 | -1.366257972 |
| AC012588.1 | 3.7882087 | 3.418956772 | 4.15212316 | 0.001319335 | 0.012090466 | -1.402254569 |
| AC073912.1 | 4.48887521 | 9.170010795 | 4.13672639 | 0.001356248 | 0.0123636 | -1.430386957 |
| AP005203.1 | -1.016712965 | 7.615832863 | -4.132079677 | 0.001367597 | 0.012432876 | -1.438881152 |
| AC005899.5 | 1.943624587 | 4.305501559 | 4.126505754 | 0.001381341 | 0.012517352 | -1.449072649 |
| AP000365.1 | 1.501545151 | 5.649831326 | 4.125967755 | 0.001382675 | 0.012517352 | -1.450056477 |
| LINC00899 | -1.266869952 | 4.361073335 | -4.117712705 | 0.001403312 | 0.01265125 | -1.465155287 |
| FP236315.1 | 1.318773416 | 2.79241375 | 4.099152631 | 0.001450884 | 0.013014142 | -1.499122674 |
| AC080011.1 | 1.126257142 | 3.441149032 | 4.098811481 | 0.001451774 | 0.013014142 | -1.499747283 |
| AC068491.2 | 1.579977285 | 2.391664073 | 4.098162037 | 0.001453469 | 0.013014142 | -1.500936373 |
| AC090630.1 | 2.24893904 | 4.919834248 | 4.097348893 | 0.001455595 | 0.013014142 | -1.502425235 |
| AL162414.1 | -1.486611752 | 2.779006825 | -4.089662512 | 0.001475849 | 0.013087072 | -1.516501556 |
| AC091138.1 | -2.490772977 | 4.493190832 | -4.080163457 | 0.001501283 | 0.013258267 | -1.533903964 |
| OLMALINC | -1.29938725 | 4.833860483 | -4.07637274 | 0.001511558 | 0.01327676 | -1.540850596 |
| AP001107.1 | 1.296554859 | 2.265932138 | 4.069586516 | 0.001530136 | 0.013385596 | -1.553289412 |
| AC005532.1 | -1.324830948 | 3.175819989 | -4.068702412 | 0.001532573 | 0.013388875 | -1.554910195 |
| AC243960.1 | -2.27835566 | 6.877017227 | -4.067413591 | 0.001536134 | 0.013396249 | -1.557273033 |
| AC004253.1 | 1.998047599 | 3.204693621 | 4.064782047 | 0.001543431 | 0.013396249 | -1.562097931 |
| AC016968.1 | -1.212084703 | 12.89687512 | -4.059865772 | 0.001557159 | 0.013476689 | -1.571113279 |
| LNCTAM34A | 1.45377943 | 6.524291809 | 4.05555085 | 0.001569311 | 0.013522935 | -1.57902741 |
| AC135782.2 | 1.258067956 | 7.148076543 | 4.054276533 | 0.001572919 | 0.013522935 | -1.581364945 |
| AC144652.1 | -2.238160446 | 8.42383153 | -4.053025329 | 0.001576469 | 0.013535533 | -1.583660204 |
| AC011450.1 | -1.420757596 | 6.715989987 | -4.049271999 | 0.00158717 | 0.013552101 | -1.590546179 |
| AC005332.4 | 1.01842637 | 10.29126944 | 4.048570418 | 0.001589179 | 0.013552101 | -1.59183344 |
| LINC01851 | -1.159302255 | 2.265171365 | -4.04796077 | 0.001590926 | 0.013552101 | -1.592952052 |
| GS1-124K5.4 | -1.73064159 | 8.036303218 | -4.040133736 | 0.001613538 | 0.013708736 | -1.607315973 |
| AP000253.1 | 1.359429447 | 5.358571616 | 4.031879853 | 0.001637743 | 0.01384191 | -1.622468204 |
| AP001057.1 | -2.648937751 | 4.798049302 | -4.006520537 | 0.001714481 | 0.014378157 | -1.669053472 |
| AC092683.2 | 1.602467562 | 6.522901376 | 3.985889178 | 0.001779644 | 0.014734269 | -1.706987582 |
| AC079322.1 | 1.625963162 | 3.511261432 | 3.983211473 | 0.001788286 | 0.014773895 | -1.711913174 |
| AC007848.2 | 1.028887671 | 4.06047676 | 3.972511595 | 0.001823256 | 0.014942873 | -1.731600378 |
| CCDC18-AS1 | 1.388750107 | 6.934213816 | 3.971800026 | 0.001825607 | 0.014943269 | -1.732909906 |
| AC116351.2 | 1.525008225 | 3.872210431 | 3.970692405 | 0.001829271 | 0.014954409 | -1.734948374 |
| AL355338.1 | -2.128421483 | 5.261104888 | -3.954529421 | 0.001883622 | 0.015244985 | -1.764704225 |
| CCDC26 | 1.722909813 | 4.015823365 | 3.953847012 | 0.001885953 | 0.015244985 | -1.765960917 |
| AC127024.5 | 1.167107931 | 6.711945938 | 3.952171977 | 0.001891688 | 0.015272319 | -1.769045715 |
| AC010880.1 | 2.01262617 | 2.879696687 | 3.940261688 | 0.001932982 | 0.015526998 | -1.79098544 |
| LINC00565 | -1.199685198 | 5.380598843 | -3.938183248 | 0.001940283 | 0.015526998 | -1.794815051 |
| AC078925.1 | 1.115656883 | 3.595072312 | 3.937466835 | 0.001942806 | 0.015526998 | -1.796135136 |
| AC023794.2 | -1.55608693 | 4.142065907 | -3.936750012 | 0.001945334 | 0.015526998 | -1.79745601 |
| AP002852.1 | -1.899457298 | 3.555294833 | -3.928433815 | 0.001974911 | 0.015630366 | -1.812782514 |
| AC092546.1 | -1.152107073 | 3.067449052 | -3.927133767 | 0.001979576 | 0.015630366 | -1.815178866 |
| LINC01664 | -4.196306227 | 5.463254292 | -3.92023231 | 0.002004533 | 0.015790002 | -1.827901986 |
| AL033381.2 | 1.181445939 | 2.6299147 | 3.914610562 | 0.002025102 | 0.015894723 | -1.838268135 |
| AC124290.1 | -1.173880395 | 4.719338148 | -3.905478753 | 0.002058977 | 0.016082817 | -1.85511083 |
| AL359715.1 | 1.51041131 | 3.651405654 | 3.896838558 | 0.002091566 | 0.016220256 | -1.87105153 |
| AC092687.3 | -1.483346558 | 5.9945953 | -3.889121208 | 0.002121123 | 0.01641605 | -1.885293468 |
| LINC01002 | 1.177574913 | 8.856304384 | 3.884519717 | 0.00213895 | 0.016511899 | -1.893786965 |
| AC240565.1 | 1.220786229 | 7.6059795 | 3.876292906 | 0.002171208 | 0.016704991 | -1.908975261 |
| LINC01572 | 1.057132545 | 4.425071549 | 3.876070684 | 0.002172086 | 0.016704991 | -1.909385582 |
| DSCR4 | 1.427772968 | 4.582296896 | 3.86690132 | 0.002208646 | 0.016906037 | -1.926318801 |
| AC087257.1 | 1.103067561 | 2.49700871 | 3.864050162 | 0.002220142 | 0.01695405 | -1.931585073 |
| AL355922.1 | -1.360817265 | 10.98576433 | -3.863398235 | 0.002222779 | 0.016954243 | -1.93278929 |
| AC233266.2 | -1.147559931 | 8.395752967 | -3.856842488 | 0.00224948 | 0.017117672 | -1.94490018 |
| AC245595.1 | 1.995425497 | 3.990817974 | 3.845002352 | 0.002298541 | 0.017308391 | -1.966779418 |
| LINC02285 | -2.523667076 | 6.846391861 | -3.840679173 | 0.002316728 | 0.017404957 | -1.974770104 |
| AP001476.3 | -1.101107139 | 2.742079427 | -3.83481724 | 0.002341625 | 0.017513984 | -1.985606551 |
| AC007365.1 | -1.476495496 | 4.545120151 | -3.83350021 | 0.002347256 | 0.017513984 | -1.988041488 |
| AC006272.1 | -1.33192326 | 4.194858959 | -3.833457988 | 0.002347437 | 0.017513984 | -1.98811955 |
| AL359378.1 | 2.429455795 | 4.803897546 | 3.830868279 | 0.002358551 | 0.017556601 | -1.992907703 |
| LINC02455 | -1.000763196 | 2.92955806 | -3.829996361 | 0.002362306 | 0.017564405 | -1.994519886 |
| LINC01827 | -1.437322272 | 3.177812266 | -3.823175059 | 0.002391889 | 0.017703251 | -2.007133925 |
| AC010680.4 | 1.436992511 | 6.556761358 | 3.811347955 | 0.002444087 | 0.018007458 | -2.029010549 |
| AC027328.1 | 1.222548179 | 2.430592345 | 3.799225182 | 0.002498805 | 0.018327396 | -2.051441521 |
| LINC01765 | -1.331815397 | 5.71415838 | -3.792425116 | 0.002530048 | 0.0185147 | -2.064027024 |
| AC068672.3 | 1.280611909 | 3.848333755 | 3.788293339 | 0.002549226 | 0.018592165 | -2.071675194 |
| AL121672.2 | 1.615790658 | 3.073709104 | 3.773671566 | 0.002618303 | 0.018961594 | -2.098747596 |
| LINC00330 | 2.070049198 | 3.579985802 | 3.769492635 | 0.002638396 | 0.019032982 | -2.106486798 |
| AL591468.1 | -1.777837493 | 4.979849868 | -3.769058872 | 0.00264049 | 0.019032982 | -2.107290157 |
| AC130352.1 | 1.866455556 | 3.980337399 | 3.768760932 | 0.00264193 | 0.019032982 | -2.107841966 |
| AC005154.3 | 1.037257163 | 3.297301246 | 3.768156318 | 0.002644854 | 0.019032982 | -2.108961773 |
| AC073188.3 | 1.342956923 | 3.654259763 | 3.762842119 | 0.002670699 | 0.019197685 | -2.118804942 |
| LINC02217 | -1.509160017 | 4.317454598 | -3.759965452 | 0.002684798 | 0.019277678 | -2.124133757 |
| AC007036.1 | -1.090782583 | 2.563267194 | -3.758916845 | 0.002689956 | 0.01929063 | -2.126076318 |
| AL031599.1 | 4.385853318 | 5.214298762 | 3.757788541 | 0.002695517 | 0.01929063 | -2.128166575 |
| AC008753.3 | -2.062335154 | 4.008521413 | -3.7531831 | 0.00271834 | 0.019347308 | -2.136699045 |
| AC002091.1 | -2.009886296 | 4.144215505 | -3.752532657 | 0.002721579 | 0.019349147 | -2.137904194 |
| AC099552.2 | -1.303149986 | 2.451564416 | -3.747721435 | 0.002745663 | 0.019477704 | -2.146819057 |
| AP000820.1 | -1.820356633 | 2.508261309 | -3.74579819 | 0.002755351 | 0.019525094 | -2.150382984 |
| AL355353.1 | -1.576778963 | 7.417739462 | -3.742928222 | 0.002769874 | 0.019563931 | -2.155701561 |
| LINC02005 | 1.716744534 | 4.033740212 | 3.736454074 | 0.002802923 | 0.019732945 | -2.167700649 |
| GOLGA8M | 2.372579447 | 7.871433322 | 3.732094794 | 0.002825404 | 0.01982609 | -2.175781076 |
| AC009806.1 | 1.051934022 | 8.42662735 | 3.727585042 | 0.002848855 | 0.019912935 | -2.184141257 |
| AC018755.1 | 1.110232833 | 3.803768049 | 3.720037415 | 0.002888551 | 0.020096019 | -2.198134926 |
| AC026726.1 | -1.702544553 | 5.515249865 | -3.718225136 | 0.002898167 | 0.020119097 | -2.201495324 |
| AC244502.1 | 2.5866116 | 2.860255579 | 3.713096869 | 0.002925554 | 0.020244959 | -2.211005068 |
| AL138781.1 | -1.867681219 | 5.010277578 | -3.699848239 | 0.002997542 | 0.020655036 | -2.235577798 |
| AC007922.1 | -2.012065345 | 4.098701169 | -3.695176266 | 0.003023358 | 0.02070748 | -2.24424468 |
| AC080078.1 | 1.089980901 | 3.562497492 | 3.695007674 | 0.003024294 | 0.02070748 | -2.244557446 |
| AC012150.1 | 1.736078456 | 4.781290785 | 3.693092169 | 0.003034947 | 0.020714872 | -2.248111108 |
| SLC2A1-AS1 | -1.042767843 | 5.061589527 | -3.685987426 | 0.003074799 | 0.020848147 | -2.261293066 |
| LINC02148 | 4.795515867 | 4.996262176 | 3.685605493 | 0.003076957 | 0.020848147 | -2.262001746 |
| AC007861.2 | 1.106692444 | 3.890510198 | 3.68349966 | 0.00308888 | 0.020885332 | -2.265909236 |
| AF038458.2 | 4.533420961 | 7.45935033 | 3.680576476 | 0.003105509 | 0.020925973 | -2.271333626 |
| AC008060.3 | -1.319449935 | 2.761088808 | -3.669322075 | 0.003170393 | 0.021264929 | -2.292220586 |
| AL133383.1 | -1.404356672 | 2.324033317 | -3.665859946 | 0.003190631 | 0.021328922 | -2.298646796 |
| LINC00987 | -2.126095312 | 7.85777645 | -3.657594489 | 0.003239482 | 0.021633205 | -2.313990284 |
| AC010636.2 | -2.12733707 | 6.443571631 | -3.648674844 | 0.003293058 | 0.021856072 | -2.33055064 |
| AL353597.1 | -1.395277207 | 5.391462101 | -3.626687129 | 0.003429031 | 0.022596791 | -2.371383764 |
| AC009502.1 | 1.921978692 | 7.124656216 | 3.622013297 | 0.003458666 | 0.022768966 | -2.380065284 |
| AC026415.1 | 1.732279239 | 4.043182512 | 3.594234422 | 0.003640282 | 0.023618656 | -2.431675738 |
| LINC01565 | 1.710177455 | 3.095951452 | 3.593931291 | 0.003642316 | 0.023618656 | -2.432239033 |
| AC136475.9 | -2.737140411 | 6.741723009 | -3.573848336 | 0.003779735 | 0.024284929 | -2.469562888 |
| AP000547.3 | 1.216555867 | 9.898681275 | 3.558453766 | 0.003888647 | 0.024708752 | -2.498179188 |
| AP001189.4 | -1.076391152 | 2.874085465 | -3.557109963 | 0.003898305 | 0.024708752 | -2.500677341 |
| LINC02245 | 2.092577532 | 7.464145241 | 3.556948961 | 0.003899464 | 0.024708752 | -2.500976648 |
| AC113382.2 | 1.469876499 | 2.963050314 | 3.556821235 | 0.003900383 | 0.024708752 | -2.501214095 |
| AC012360.3 | 1.10651983 | 6.586552424 | 3.556762223 | 0.003900808 | 0.024708752 | -2.5013238 |
| TTTY12 | -1.262311756 | 2.977151576 | -3.556684896 | 0.003901365 | 0.024708752 | -2.501467555 |
| LINC01792 | 1.428496669 | 3.859032496 | 3.556626885 | 0.003901783 | 0.024708752 | -2.501575399 |
| AL450442.1 | 1.032236481 | 2.923305804 | 3.553444138 | 0.003924775 | 0.024739281 | -2.50749234 |
| AL078605.1 | 1.44066789 | 3.33893648 | 3.551427378 | 0.003939415 | 0.024801887 | -2.511241728 |
| FOCAD-AS1 | -1.06945792 | 4.545869797 | -3.548784774 | 0.003958684 | 0.024899045 | -2.516154737 |
| AL732372.1 | 1.807588109 | 3.135957628 | 3.52135639 | 0.004164438 | 0.025916938 | -2.567154918 |
| C15orf32 | -1.26033921 | 5.22330723 | -3.520761086 | 0.004169022 | 0.025920617 | -2.568261943 |
| AC021979.1 | -1.329987352 | 2.554758443 | -3.511763204 | 0.004238946 | 0.026207584 | -2.584994913 |
| AL391987.4 | -1.299626965 | 7.243402263 | -3.504278827 | 0.004298017 | 0.026369025 | -2.598914007 |
| CASC15 | 1.45670371 | 4.617771063 | 3.499337746 | 0.004337474 | 0.026560888 | -2.608103524 |
| AC004944.1 | 4.188375242 | 4.266987911 | 3.494392477 | 0.004377333 | 0.02674265 | -2.617301068 |
| AC245297.2 | -1.469717215 | 7.260863294 | -3.491748832 | 0.004398794 | 0.026835121 | -2.622217989 |
| AC074050.4 | -1.546396751 | 5.96157625 | -3.490628063 | 0.004407924 | 0.026865573 | -2.624302527 |
| AC087203.3 | -1.296226907 | 3.010455617 | -3.486664783 | 0.004440367 | 0.026987284 | -2.631673987 |
| AC092620.1 | -1.732814246 | 6.318482187 | -3.469305236 | 0.004585355 | 0.027687012 | -2.663963004 |
| LINC00324 | -1.536176706 | 5.807477488 | -3.465735371 | 0.004615763 | 0.027818863 | -2.670603224 |
| CNNM3-DT | -1.487682038 | 7.048334121 | -3.451248424 | 0.00474128 | 0.028408424 | -2.697550566 |
| AC027801.1 | 1.079060569 | 2.73640921 | 3.449537595 | 0.00475633 | 0.028408424 | -2.700732939 |
| AC027243.2 | -1.182864186 | 3.036929347 | -3.449496403 | 0.004756693 | 0.028408424 | -2.700809561 |
| LINC02332 | -2.389743678 | 5.872343695 | -3.44892497 | 0.004761731 | 0.028408455 | -2.701872506 |
| AL353764.1 | 1.137947545 | 3.149649544 | 3.44143896 | 0.004828236 | 0.028726012 | -2.715797563 |
| AL354977.2 | 1.704642908 | 5.210824469 | 3.439569896 | 0.004844987 | 0.028799278 | -2.7192743 |
| AC003005.2 | 1.21510338 | 5.529772714 | 3.430202978 | 0.00492983 | 0.02909048 | -2.736698158 |
| AC020551.1 | 3.889806611 | 5.210396486 | 3.42861144 | 0.004944395 | 0.029097072 | -2.739658648 |
| LINC00526 | -1.541533944 | 6.502488778 | -3.410186026 | 0.005116232 | 0.029864624 | -2.773932157 |
| AC104350.1 | 1.134933963 | 3.955643407 | 3.400534166 | 0.005208653 | 0.03026801 | -2.791885259 |
| AC097634.1 | 2.127954641 | 2.893973092 | 3.397471162 | 0.005238336 | 0.03041327 | -2.797582541 |
| LINC01715 | -1.014504999 | 2.903994684 | -3.39658489 | 0.005246956 | 0.030436096 | -2.799231022 |
| AC020687.1 | -1.020424734 | 2.940224322 | -3.394557684 | 0.005266728 | 0.030458433 | -2.803001641 |
| AC015468.3 | -2.652628353 | 4.715780675 | -3.393652972 | 0.005275577 | 0.030458433 | -2.804684405 |
| LINC01483 | 1.877263802 | 4.305975809 | 3.387802135 | 0.005333165 | 0.030689339 | -2.815566803 |
| AC134312.6 | -1.333627876 | 5.27840669 | -3.385100966 | 0.005359967 | 0.030761754 | -2.820590814 |
| AC009292.1 | -1.298166007 | 3.494259429 | -3.379911679 | 0.00541184 | 0.030977296 | -2.830242392 |
| AP003086.1 | 1.551210081 | 6.545818492 | 3.376107433 | 0.005450191 | 0.031114505 | -2.837317767 |
| AC005823.2 | 1.042823778 | 5.644478563 | 3.369323974 | 0.005519262 | 0.031398359 | -2.849933697 |
| AC005394.1 | 1.058926935 | 2.616549989 | 3.364820737 | 0.005565604 | 0.031578963 | -2.858308571 |
| AP000439.3 | 1.502270571 | 4.479425816 | 3.363808464 | 0.005576075 | 0.031610745 | -2.860191107 |
| LINC01126 | 1.112540536 | 9.09989654 | 3.355050006 | 0.005667513 | 0.032073085 | -2.876478781 |
| CEP83-DT | 1.216488211 | 3.741773863 | 3.353868083 | 0.005679968 | 0.03211557 | -2.878676667 |
| LINC00342 | 1.639179917 | 8.878331097 | 3.342422588 | 0.005802024 | 0.032606873 | -2.899959489 |
| CXXC5-AS1 | 1.178590622 | 3.621644067 | 3.341386107 | 0.005813208 | 0.032641462 | -2.901886721 |
| AC026150.1 | 2.412435789 | 6.209225718 | 3.336553837 | 0.005865636 | 0.032891937 | -2.910871601 |
| AC007272.1 | -1.21231347 | 10.82834384 | -3.336341545 | 0.00586795 | 0.032891937 | -2.911266318 |
| AC024084.1 | -1.399051416 | 4.374158893 | -3.333851152 | 0.005895167 | 0.03293075 | -2.915896668 |
| AC090204.1 | -3.165206427 | 7.637799822 | -3.330847013 | 0.005928169 | 0.033029826 | -2.921482075 |
| TRERNA1 | -2.221384129 | 5.361433142 | -3.328028201 | 0.005959306 | 0.033131934 | -2.926722769 |
| AL138720.1 | -1.064904356 | 4.96597884 | -3.303120196 | 0.00624173 | 0.034451588 | -2.973024543 |
| AC008592.2 | 1.052292353 | 3.000940763 | 3.299155531 | 0.006287917 | 0.034618208 | -2.980393266 |
| AP003059.1 | 1.595674643 | 3.033436037 | 3.292306458 | 0.006368522 | 0.034943428 | -2.993122069 |
| AC104794.3 | -1.029894083 | 4.248352555 | -3.290511763 | 0.006389815 | 0.035007475 | -2.996457267 |
| AC079054.1 | -1.016022354 | 3.302500443 | -3.288485544 | 0.006413942 | 0.035044528 | -3.000222623 |
| C17orf51 | -1.102447561 | 5.356651505 | -3.281926143 | 0.006492681 | 0.035394227 | -3.012411342 |
| AC123912.4 | -1.073119059 | 6.293117174 | -3.273942907 | 0.00658983 | 0.035787138 | -3.027244283 |
| AC125494.2 | 1.518578223 | 4.248123125 | 3.26276262 | 0.006728358 | 0.036243793 | -3.04801434 |
| AP000924.1 | 2.499460229 | 3.248003888 | 3.259188439 | 0.006773261 | 0.036425215 | -3.05465345 |
| AC011815.1 | 1.603731008 | 4.855831041 | 3.251836312 | 0.00686658 | 0.036744409 | -3.068308917 |
| HIF1A-AS2 | 1.731931416 | 4.059217245 | 3.241491882 | 0.007000081 | 0.037282263 | -3.087519161 |
| AC010336.3 | 4.000426959 | 6.352732883 | 3.231848715 | 0.00712689 | 0.037702234 | -3.105423816 |
| LINC01106 | 1.111632712 | 3.598972572 | 3.222575026 | 0.007251025 | 0.038048829 | -3.122639294 |
| AC011228.1 | 1.213234648 | 4.551087431 | 3.217114294 | 0.007325138 | 0.038313837 | -3.132774965 |
| LINC01385 | 1.095644094 | 4.166551868 | 3.215200739 | 0.007351289 | 0.038364546 | -3.136326443 |
| AL050344.2 | 1.129103255 | 2.78554809 | 3.215107606 | 0.007352564 | 0.038364546 | -3.136499291 |
| AC016266.1 | -1.30092852 | 6.649437081 | -3.21350575 | 0.007374532 | 0.038386597 | -3.139472154 |
| AC010096.1 | -1.106698301 | 2.20435624 | -3.198229456 | 0.00758738 | 0.03914919 | -3.167817989 |
| AL160290.1 | 1.01787854 | 2.54007717 | 3.193000124 | 0.007661654 | 0.039398689 | -3.177518996 |
| AC104794.2 | -2.653036637 | 6.401964862 | -3.192689904 | 0.007666083 | 0.039398689 | -3.178094452 |
| AL513542.1 | 1.447551854 | 4.671843705 | 3.188376511 | 0.007727935 | 0.039622451 | -3.186095333 |
| AC112721.1 | -1.465605548 | 2.437572076 | -3.187034972 | 0.007747274 | 0.039690255 | -3.188583575 |
| AC007221.1 | 1.025152663 | 3.18856322 | 3.179193969 | 0.007861285 | 0.040147603 | -3.20312518 |
| AL023754.1 | 1.210571177 | 3.758145509 | 3.167843146 | 0.008029329 | 0.040717483 | -3.22417088 |
| SNHG5 | -1.186796842 | 14.07956786 | -3.160406905 | 0.008141375 | 0.041157058 | -3.237955126 |
| AL133410.1 | -1.001222538 | 6.460781033 | -3.143245913 | 0.008405984 | 0.042198952 | -3.269755014 |
| AL390066.1 | 1.974654459 | 6.196743247 | 3.137300844 | 0.008499655 | 0.042570417 | -3.280767794 |
| AL121894.1 | -1.227555327 | 3.01845036 | -3.136104693 | 0.008518628 | 0.042632547 | -3.282983339 |
| LINC00102 | 1.302926205 | 2.484074781 | 3.127777083 | 0.008651902 | 0.043100151 | -3.298405766 |
| AC018521.7 | 1.048582762 | 2.472657779 | 3.121989012 | 0.008745765 | 0.04346766 | -3.309122746 |
| LINC00467 | -1.269264669 | 6.721632275 | -3.110308912 | 0.008938303 | 0.044282656 | -3.330743234 |
| MIAT | -1.148859156 | 5.656426376 | -3.095689547 | 0.009185297 | 0.04506558 | -3.357792796 |
| AC017002.3 | -2.162441479 | 8.326952435 | -3.085508944 | 0.009361331 | 0.045448315 | -3.376621532 |
| AC025254.1 | 1.270531796 | 4.900945142 | 3.083254318 | 0.009400771 | 0.045605683 | -3.380790486 |
| AC092111.2 | 1.080652261 | 2.173311296 | 3.081077613 | 0.009439006 | 0.045677941 | -3.384815039 |
| AC009804.2 | 1.070183335 | 2.935468554 | 3.080334214 | 0.0094521 | 0.045677941 | -3.386189452 |
| LINC01943 | -2.916977347 | 7.503735998 | -3.065208472 | 0.009722507 | 0.046651835 | -3.414146123 |
| AC093274.1 | -1.037285346 | 4.22860906 | -3.049766886 | 0.010006555 | 0.04754945 | -3.442670014 |
| DIRC3 | -1.18707953 | 4.578788306 | -3.03796325 | 0.010229272 | 0.048465842 | -3.464462068 |
| AL451060.1 | -1.48689257 | 3.052608557 | -3.021281854 | 0.010552506 | 0.04985176 | -3.495241354 |
