# Supplementary material for: Regulatory function and mechanism research for m6A modification WTAP via SUCLG2-AS1- miR-17-5p-JAK1 axis in AML
Source: BMC Cancer. 2024 Jan 17;24:98. doi: 10.1186/s12885-023-11687-4 (PMC10795285; doi:10.1186/s12885-023-11687-4)
Supplement: Supplementary file 3 — Additional file 3: Supplementary Table S3. The differentially expressed genes of mRNA. [file 12885_2023_11687_MOESM3_ESM.docx]

**Supplementary Table S3 The differentially expressed genes of mRNA**

| id | logFC | AveExpr | t | P.Value | adj.P.Val | B |
| --- | --- | --- | --- | --- | --- | --- |
| ENKUR | -5.770702787 | 5.115562921 | -26.03498294 | 2.30E-12 | 3.49E-08 | 18.04516418 |
| UPP1 | -4.191009 | 7.247300216 | -23.04895733 | 1.04E-11 | 7.37E-08 | 16.83115198 |
| SLC4A10 | -3.971257202 | 3.790171003 | -22.43112361 | 1.46E-11 | 7.37E-08 | 16.55154638 |
| PID1 | -4.336617664 | 6.724027986 | -21.5772726 | 2.36E-11 | 8.93E-08 | 16.14710761 |
| KRT2 | -4.489305872 | 3.794769689 | -20.79922592 | 3.71E-11 | 9.69E-08 | 15.75920147 |
| WLS | -5.079736593 | 4.467699795 | -20.74165339 | 3.84E-11 | 9.69E-08 | 15.7297285 |
| ASGR1 | -5.032990947 | 9.428736622 | -19.1770968 | 1.00E-10 | 2.18E-07 | 14.88528975 |
| PVALB | -4.791378505 | 7.467971171 | -16.9596128 | 4.50E-10 | 7.62E-07 | 13.52694999 |
| CES1 | -6.075239399 | 5.242799664 | -16.95267708 | 4.52E-10 | 7.62E-07 | 13.52236798 |
| SCGB1C2 | -5.949295572 | 5.668742247 | -16.69259994 | 5.46E-10 | 7.89E-07 | 13.34892762 |
| G0S2 | -6.129665579 | 8.626819397 | -16.62595832 | 5.73E-10 | 7.89E-07 | 13.3039711 |
| TREML1 | -6.721277767 | 6.929817312 | -16.19681949 | 7.87E-10 | 9.67E-07 | 13.00931867 |
| CXCR2 | -5.289873988 | 4.967832627 | -15.93077822 | 9.61E-10 | 9.67E-07 | 12.82205478 |
| HGD | -5.71466876 | 7.476556122 | -15.93021809 | 9.61E-10 | 9.67E-07 | 12.82165672 |
| DEFA4 | -6.189108143 | 9.47718402 | -15.66485095 | 1.18E-09 | 9.67E-07 | 12.63123996 |
| NRG1 | -5.234468754 | 5.018232751 | -15.6521741 | 1.19E-09 | 9.67E-07 | 12.6220514 |
| ALDH2 | -4.649965182 | 9.173803177 | -15.6046496 | 1.23E-09 | 9.67E-07 | 12.58752859 |
| PLA2G7 | -6.363804433 | 6.292159316 | -15.53915916 | 1.30E-09 | 9.67E-07 | 12.5397584 |
| LIM2 | -3.465043508 | 6.424099419 | -15.51009487 | 1.33E-09 | 9.67E-07 | 12.51848492 |
| TNNC2 | -4.626256955 | 8.210627731 | -15.44799401 | 1.39E-09 | 9.67E-07 | 12.47287861 |
| MLH3 | -4.355246056 | 8.822051974 | -15.38228979 | 1.47E-09 | 9.67E-07 | 12.42439951 |
| MYB | 5.506831849 | 7.240515827 | 15.38209097 | 1.47E-09 | 9.67E-07 | 12.42425245 |
| NRGN | -5.197145657 | 10.82835931 | -15.38070106 | 1.47E-09 | 9.67E-07 | 12.42322439 |
| CNTNAP2 | -3.246694402 | 3.360185018 | -15.25484517 | 1.62E-09 | 9.73E-07 | 12.32969612 |
| RBP7 | -8.310041225 | 7.643590575 | -15.25300964 | 1.62E-09 | 9.73E-07 | 12.32832564 |
| PRTFDC1 | -3.044535897 | 4.469493888 | -15.19058963 | 1.71E-09 | 9.73E-07 | 12.28160997 |
| PADI2 | -3.193721564 | 5.485018042 | -15.15428766 | 1.76E-09 | 9.73E-07 | 12.25434208 |
| TRIM7 | -3.002427332 | 5.519763344 | -15.08131014 | 1.86E-09 | 9.73E-07 | 12.19930363 |
| PDE5A | -4.328052878 | 5.709778009 | -15.06401891 | 1.89E-09 | 9.73E-07 | 12.18621922 |
| CDK6 | 4.27868539 | 9.66130611 | 15.00210336 | 1.98E-09 | 9.73E-07 | 12.13922933 |
| 4-Sep | -4.145863313 | 7.781327927 | -14.99637536 | 1.99E-09 | 9.73E-07 | 12.13487123 |
| TNFRSF18 | -3.117549063 | 6.125921762 | -14.92506814 | 2.11E-09 | 9.98E-07 | 12.08046198 |
| 10-Sep | -3.647876995 | 3.830575383 | -14.87945917 | 2.19E-09 | 1.00E-06 | 12.0455094 |
| C15orf48 | -3.396623051 | 6.044656401 | -14.77923165 | 2.37E-09 | 1.06E-06 | 11.96828051 |
| MB21D2 | -3.596228829 | 3.629639349 | -14.56154337 | 2.83E-09 | 1.23E-06 | 11.7985333 |
| TNFSF12-TNFSF13 | -3.155304539 | 5.715547684 | -14.39950046 | 3.24E-09 | 1.36E-06 | 11.6703583 |
| HNMT | -3.411433683 | 3.330884301 | -14.12650862 | 4.07E-09 | 1.67E-06 | 11.45083038 |
| CLDN5 | -5.192279031 | 7.528494607 | -14.07530301 | 4.25E-09 | 1.70E-06 | 11.40914197 |
| ZDHHC18 | -2.179456568 | 7.935136356 | -14.00654402 | 4.51E-09 | 1.75E-06 | 11.35290509 |
| WDR90 | 3.062113255 | 9.674148895 | 13.9298133 | 4.82E-09 | 1.82E-06 | 11.2897971 |
| ENPP5 | -3.780661514 | 3.739122317 | -13.74356701 | 5.65E-09 | 2.09E-06 | 11.13505739 |
| SLPI | -4.52586211 | 7.276320207 | -13.55969633 | 6.64E-09 | 2.39E-06 | 10.98008859 |
| LEFTY1 | -4.296460358 | 4.490298353 | -13.47793244 | 7.13E-09 | 2.46E-06 | 10.91046198 |
| DTHD1 | -3.516478546 | 3.557498075 | -13.47752331 | 7.13E-09 | 2.46E-06 | 10.91011246 |
| SULT1B1 | -5.169419295 | 5.226075958 | -13.33544693 | 8.09E-09 | 2.72E-06 | 10.7880597 |
| ACRBP | -4.350533221 | 10.6771376 | -13.28024268 | 8.50E-09 | 2.80E-06 | 10.74026795 |
| TAGAP | -2.265107661 | 9.903696485 | -13.25644236 | 8.68E-09 | 2.80E-06 | 10.71959937 |
| S100A12 | -6.852211906 | 11.88084368 | -13.19587968 | 9.16E-09 | 2.82E-06 | 10.6668309 |
| TRPV1 | 3.917993009 | 3.941789826 | 13.19184439 | 9.20E-09 | 2.82E-06 | 10.66330599 |
| CAMP | -6.894676075 | 9.60279084 | -13.1772154 | 9.32E-09 | 2.82E-06 | 10.65051785 |
| GNG8 | -2.07479561 | 7.154388756 | -13.11099941 | 9.89E-09 | 2.94E-06 | 10.59244921 |
| CYP2S1 | -2.454711133 | 6.574598974 | -13.00821401 | 1.09E-08 | 3.16E-06 | 10.50170633 |
| SCIMP | -3.304384399 | 5.903077387 | -12.98675329 | 1.11E-08 | 3.16E-06 | 10.48266655 |
| CYP27A1 | -6.858348373 | 8.369331779 | -12.94894927 | 1.15E-08 | 3.21E-06 | 10.44904812 |
| ABCD2 | -2.661569814 | 3.066648062 | -12.91280876 | 1.18E-08 | 3.26E-06 | 10.41681444 |
| S100A9 | -6.236550179 | 12.99084781 | -12.78976481 | 1.33E-08 | 3.57E-06 | 10.30637334 |
| MYCT1 | -3.618210686 | 3.542343479 | -12.77209374 | 1.35E-08 | 3.57E-06 | 10.29042298 |
| ZNF618 | 3.461049094 | 6.750342946 | 12.75849102 | 1.36E-08 | 3.57E-06 | 10.27812946 |
| FSCN3 | -2.572226583 | 5.92311574 | -12.70844874 | 1.43E-08 | 3.67E-06 | 10.23278816 |
| SIRPD | -2.982961294 | 5.527168345 | -12.56008714 | 1.64E-08 | 4.11E-06 | 10.09728886 |
| PRSS23 | -5.100415901 | 5.126369661 | -12.55158035 | 1.65E-08 | 4.11E-06 | 10.08947046 |
| NUAK2 | -3.711608593 | 8.819215397 | -12.49065997 | 1.75E-08 | 4.28E-06 | 10.0333229 |
| ARPIN | -3.500960923 | 3.948965005 | -12.45987183 | 1.80E-08 | 4.34E-06 | 10.00484166 |
| CXCR1 | -3.783192941 | 7.385217905 | -12.32741924 | 2.05E-08 | 4.84E-06 | 9.881500934 |
| MYCL | -2.407740152 | 6.434724931 | -12.28004451 | 2.14E-08 | 4.99E-06 | 9.837062485 |
| BATF3 | -4.702521374 | 7.459005689 | -12.24644506 | 2.21E-08 | 5.01E-06 | 9.805441579 |
| IGSF6 | -5.631246741 | 7.474776977 | -12.24410016 | 2.21E-08 | 5.01E-06 | 9.803231533 |
| CPXM1 | 5.441938265 | 7.572761511 | 12.16587662 | 2.39E-08 | 5.32E-06 | 9.729263901 |
| IFNG | -5.255499834 | 5.575241415 | -12.1014023 | 2.54E-08 | 5.58E-06 | 9.667941246 |
| SCO2 | -3.439467331 | 11.2476078 | -12.0653601 | 2.63E-08 | 5.69E-06 | 9.633519633 |
| KLHL23 | 2.951826952 | 4.456773252 | 12.01127114 | 2.77E-08 | 5.92E-06 | 9.581671511 |
| FCMR | -3.089290846 | 8.552347177 | -11.99250168 | 2.82E-08 | 5.94E-06 | 9.563625779 |
| NTN1 | 6.279578031 | 4.629099775 | 11.96672835 | 2.90E-08 | 6.01E-06 | 9.538800837 |
| CTSZ | -3.363127425 | 9.70875208 | -11.93696477 | 2.98E-08 | 6.09E-06 | 9.510066935 |
| ANG | -4.84451764 | 6.907301872 | -11.92647891 | 3.01E-08 | 6.09E-06 | 9.499927051 |
| QPCT | -3.967079365 | 8.27833588 | -11.87713839 | 3.16E-08 | 6.29E-06 | 9.452096745 |
| SMPDL3A | -5.417780111 | 4.865625304 | -11.8659797 | 3.20E-08 | 6.29E-06 | 9.441252587 |
| AHI1 | 2.2450292 | 5.124194152 | 11.84779448 | 3.25E-08 | 6.32E-06 | 9.423558545 |
| SMKR1 | -4.096251319 | 5.539397015 | -11.81709122 | 3.35E-08 | 6.43E-06 | 9.393624182 |
| PRSS57 | 5.167893112 | 12.83636744 | 11.78501196 | 3.46E-08 | 6.52E-06 | 9.362267006 |
| HRASLS | -5.194697425 | 5.251344139 | -11.77805849 | 3.49E-08 | 6.52E-06 | 9.355459067 |
| FPR1 | -6.035270793 | 8.658268143 | -11.75642144 | 3.56E-08 | 6.55E-06 | 9.334249776 |
| SLC15A3 | -4.395242656 | 9.549926703 | -11.73964099 | 3.62E-08 | 6.55E-06 | 9.317774883 |
| SMCO4 | -3.622833951 | 9.8822726 | -11.72849661 | 3.66E-08 | 6.55E-06 | 9.30682076 |
| AL163636.2 | -4.786966285 | 5.389394583 | -11.7248347 | 3.68E-08 | 6.55E-06 | 9.303219155 |
| GEMIN5 | 2.004111056 | 7.483776609 | 11.69471571 | 3.79E-08 | 6.67E-06 | 9.273554611 |
| ZBED3 | 3.068368117 | 8.940286726 | 11.67369737 | 3.87E-08 | 6.74E-06 | 9.252809421 |
| CLEC11A | 4.22102911 | 12.9074793 | 11.62669988 | 4.05E-08 | 6.91E-06 | 9.206291363 |
| SMIM24 | 5.379825275 | 8.858763292 | 11.62459316 | 4.06E-08 | 6.91E-06 | 9.20420188 |
| NSMCE3 | -1.982715329 | 7.671756058 | -11.59431985 | 4.19E-08 | 7.01E-06 | 9.174135629 |
| CTSH | -5.166977038 | 9.145097335 | -11.58912326 | 4.21E-08 | 7.01E-06 | 9.16896696 |
| CBX2 | 3.962156052 | 6.16353936 | 11.55388952 | 4.36E-08 | 7.18E-06 | 9.133863358 |
| CLIC3 | -4.948233549 | 8.081812184 | -11.52919685 | 4.47E-08 | 7.28E-06 | 9.109200324 |
| TMEM132A | -2.911014271 | 7.264386186 | -11.47361264 | 4.73E-08 | 7.62E-06 | 9.053496273 |
| EFHD2 | -2.055254855 | 12.66957556 | -11.42214347 | 4.98E-08 | 7.84E-06 | 9.0016846 |
| MYL9 | -2.169959701 | 7.024463897 | -11.41868246 | 5.00E-08 | 7.84E-06 | 8.998192526 |
| ZMAT4 | -1.779354935 | 5.390177953 | -11.41479651 | 5.02E-08 | 7.84E-06 | 8.994270502 |
| NEXN | -5.130221564 | 5.783321413 | -11.39311703 | 5.13E-08 | 7.93E-06 | 8.972366281 |
| SERTAD3 | -1.910174709 | 8.361112779 | -11.34565279 | 5.39E-08 | 8.18E-06 | 8.924270591 |
| CCDC151 | -2.435499137 | 6.158674761 | -11.3433134 | 5.40E-08 | 8.18E-06 | 8.92189511 |
| ZGLP1 | -1.630798411 | 8.914606329 | -11.32565849 | 5.50E-08 | 8.22E-06 | 8.903952792 |
| FCAR | -3.328897436 | 7.098161408 | -11.31896657 | 5.53E-08 | 8.22E-06 | 8.897144963 |
| SIGLEC7 | -3.712804132 | 6.379814734 | -11.22663457 | 6.09E-08 | 8.95E-06 | 8.80282099 |
| SETMAR | 1.981218849 | 6.593885818 | 11.13202766 | 6.71E-08 | 9.70E-06 | 8.705407412 |
| CD1E | -2.715731329 | 3.125549864 | -11.12315371 | 6.77E-08 | 9.70E-06 | 8.696230143 |
| S100A8 | -5.154791668 | 15.37442066 | -11.12094121 | 6.79E-08 | 9.70E-06 | 8.693940933 |
| DNLZ | 4.361376891 | 10.44604757 | 11.08813478 | 7.02E-08 | 9.95E-06 | 8.65994679 |
| LAIR2 | -5.792395162 | 9.000981678 | -11.05255635 | 7.29E-08 | 1.01E-05 | 8.622973186 |
| BAHCC1 | 3.308437549 | 8.710345289 | 11.04697015 | 7.33E-08 | 1.01E-05 | 8.617157781 |
| CD3G | -5.032823114 | 5.934356749 | -11.04486399 | 7.35E-08 | 1.01E-05 | 8.614964488 |
| C20orf96 | 4.738219441 | 8.288090807 | 11.0248854 | 7.50E-08 | 1.02E-05 | 8.594139831 |
| MCM7 | 2.46673039 | 13.38028388 | 10.98754464 | 7.80E-08 | 1.06E-05 | 8.555122763 |
| CXCL10 | -4.432683136 | 4.137600463 | -10.92778084 | 8.31E-08 | 1.11E-05 | 8.492417307 |
| AGAP3 | -2.765724039 | 8.921509128 | -10.91007747 | 8.47E-08 | 1.12E-05 | 8.473781131 |
| C3orf14 | -5.925204887 | 5.300878026 | -10.90622213 | 8.50E-08 | 1.12E-05 | 8.469718908 |
| ZNF683 | -3.299402851 | 9.353590902 | -10.89886403 | 8.57E-08 | 1.12E-05 | 8.461962263 |
| PFAS | 2.037197194 | 5.368990197 | 10.87412952 | 8.79E-08 | 1.14E-05 | 8.435852275 |
| CCL5 | -4.353119178 | 12.1504438 | -10.85286812 | 8.99E-08 | 1.15E-05 | 8.413364379 |
| CLEC4E | -5.081956454 | 4.397125257 | -10.79391757 | 9.57E-08 | 1.22E-05 | 8.350798816 |
| ZNF8 | 2.233633762 | 6.589661622 | 10.75437628 | 9.99E-08 | 1.26E-05 | 8.308655321 |
| MSRB1 | -1.954313873 | 8.079828324 | -10.68251532 | 1.08E-07 | 1.34E-05 | 8.231698034 |
| ALDOC | -3.094021324 | 6.914855751 | -10.68059236 | 1.08E-07 | 1.34E-05 | 8.229632166 |
| ST6GALNA2 | -2.98891221 | 6.212463695 | -10.66002372 | 1.10E-07 | 1.36E-05 | 8.20751349 |
| ADNP | 1.591935719 | 9.543966157 | 10.65637139 | 1.11E-07 | 1.36E-05 | 8.203581824 |
| CCR5 | -2.774103822 | 6.191838991 | -10.61076262 | 1.17E-07 | 1.41E-05 | 8.154380562 |
| TNFSF10 | -4.214191527 | 8.356552419 | -10.56505563 | 1.22E-07 | 1.47E-05 | 8.104878813 |
| HS3ST1 | -3.093874545 | 4.612164403 | -10.56074694 | 1.23E-07 | 1.47E-05 | 8.10020232 |
| MNS1 | 3.869918093 | 4.078734074 | 10.46621635 | 1.36E-07 | 1.61E-05 | 7.997162594 |
| OASL | -4.146368691 | 8.52052165 | -10.44111584 | 1.40E-07 | 1.63E-05 | 7.969660662 |
| INKA1 | 3.121570433 | 13.91858258 | 10.44094729 | 1.40E-07 | 1.63E-05 | 7.969475786 |
| CLU | -3.24435369 | 4.865530906 | -10.38994442 | 1.48E-07 | 1.70E-05 | 7.913407699 |
| CLEC4G | -2.682084112 | 7.032991839 | -10.38525334 | 1.49E-07 | 1.70E-05 | 7.90823826 |
| FCER1G | -2.397067249 | 10.26168228 | -10.3822074 | 1.49E-07 | 1.70E-05 | 7.904880585 |
| FGFBP2 | -5.412665029 | 9.009330818 | -10.35346369 | 1.54E-07 | 1.74E-05 | 7.873151331 |
| LCK | -4.235004321 | 9.971136985 | -10.34887721 | 1.55E-07 | 1.74E-05 | 7.86808112 |
| TIGIT | -3.522334285 | 6.16212319 | -10.31982729 | 1.60E-07 | 1.77E-05 | 7.835920305 |
| GIMAP4 | -4.369256984 | 9.626643063 | -10.31940289 | 1.60E-07 | 1.77E-05 | 7.835449856 |
| LSM10 | -2.277565708 | 11.0314899 | -10.2778009 | 1.68E-07 | 1.84E-05 | 7.78924913 |
| CD200R1 | -3.029986829 | 3.759578318 | -10.26093549 | 1.71E-07 | 1.85E-05 | 7.770471609 |
| PPP2R2B | -2.492028275 | 5.507030587 | -10.258659 | 1.71E-07 | 1.85E-05 | 7.767934903 |
| CKAP4 | -2.005401756 | 8.741306896 | -10.2448971 | 1.74E-07 | 1.85E-05 | 7.752589222 |
| CLEC4C | -3.114051587 | 4.744491741 | -10.24303128 | 1.74E-07 | 1.85E-05 | 7.75050725 |
| PM20D2 | 2.548319535 | 7.909264952 | 10.24048768 | 1.75E-07 | 1.85E-05 | 7.747668444 |
| SOX4 | 4.125415454 | 9.747958031 | 10.22920343 | 1.77E-07 | 1.86E-05 | 7.73506693 |
| PKD2L1 | -4.134012352 | 4.112501688 | -10.20863121 | 1.81E-07 | 1.89E-05 | 7.712061232 |
| LYPD2 | -3.149008557 | 6.389931048 | -10.19602864 | 1.84E-07 | 1.91E-05 | 7.697947458 |
| RRAS | -1.626865168 | 11.49030722 | -10.18955199 | 1.85E-07 | 1.91E-05 | 7.690688131 |
| ZNF850 | 2.935873478 | 4.181927187 | 10.1793313 | 1.87E-07 | 1.92E-05 | 7.679223939 |
| CCDC126 | -1.871707153 | 6.891518079 | -10.14751602 | 1.94E-07 | 1.96E-05 | 7.643472129 |
| FASLG | -3.457970816 | 5.550829292 | -10.14091573 | 1.95E-07 | 1.96E-05 | 7.636042694 |
| SLC4A4 | -3.503974439 | 3.581422888 | -10.14021479 | 1.95E-07 | 1.96E-05 | 7.635253446 |
| SIGLEC1 | -4.605556192 | 4.272310942 | -10.11157602 | 2.02E-07 | 2.01E-05 | 7.602965223 |
| MTMR11 | -4.732935217 | 5.490833731 | -10.1028088 | 2.04E-07 | 2.02E-05 | 7.593064564 |
| GIMAP1 | -3.044640268 | 7.255566035 | -10.08372821 | 2.08E-07 | 2.05E-05 | 7.571490893 |
| MYO15B | 2.94022987 | 6.855824277 | 10.06905703 | 2.12E-07 | 2.07E-05 | 7.554878177 |
| BAG3 | -3.914891454 | 4.18078901 | -10.0595161 | 2.14E-07 | 2.08E-05 | 7.544063137 |
| DCAF16 | 2.239117904 | 10.13603211 | 10.02488983 | 2.23E-07 | 2.15E-05 | 7.504736605 |
| MPO | 6.31520501 | 12.68008799 | 10.01832049 | 2.24E-07 | 2.15E-05 | 7.497261994 |
| ABCC3 | -5.094848767 | 6.574854627 | -10.01329668 | 2.25E-07 | 2.15E-05 | 7.49154299 |
| TP53BP1 | 1.887792902 | 7.352448983 | 9.997351976 | 2.30E-07 | 2.16E-05 | 7.473375087 |
| RAB5C | -2.287455061 | 9.062661308 | -9.996135302 | 2.30E-07 | 2.16E-05 | 7.471987723 |
| CCDC152 | -3.202729596 | 3.66451829 | -9.976554115 | 2.35E-07 | 2.20E-05 | 7.449638993 |
| ZNF660 | 4.578933174 | 5.020693659 | 9.940419674 | 2.45E-07 | 2.28E-05 | 7.408296082 |
| TMEM45B | -4.053062042 | 4.458336137 | -9.904002822 | 2.55E-07 | 2.36E-05 | 7.366496567 |
| CCDC170 | -3.522213628 | 4.974210652 | -9.890581397 | 2.59E-07 | 2.37E-05 | 7.35105745 |
| WASF3 | -3.349056142 | 3.470463707 | -9.889422982 | 2.60E-07 | 2.37E-05 | 7.349724028 |
| ABI3 | -2.021307255 | 9.466889867 | -9.845977465 | 2.73E-07 | 2.45E-05 | 7.299616339 |
| CLEC10A | -3.821669782 | 8.332719283 | -9.845005067 | 2.73E-07 | 2.45E-05 | 7.298492625 |
| OLFM1 | -2.527865023 | 3.934841728 | -9.842113652 | 2.74E-07 | 2.45E-05 | 7.295150702 |
| AQP9 | -6.202069957 | 8.035772533 | -9.837659393 | 2.75E-07 | 2.45E-05 | 7.290000757 |
| NAGA | -2.349073602 | 8.520180166 | -9.83293359 | 2.77E-07 | 2.45E-05 | 7.284534639 |
| ZNF100 | 2.193552373 | 4.977809623 | 9.822008095 | 2.80E-07 | 2.47E-05 | 7.27188887 |
| E2F5 | -1.486846617 | 6.030207185 | -9.818087584 | 2.82E-07 | 2.47E-05 | 7.267348072 |
| CD160 | -2.579169828 | 5.485790232 | -9.804160995 | 2.86E-07 | 2.49E-05 | 7.25120533 |
| EPB41L3 | -2.543307494 | 7.798330737 | -9.800022749 | 2.88E-07 | 2.49E-05 | 7.246404723 |
| RGS18 | -4.20412232 | 11.79217062 | -9.78805281 | 2.92E-07 | 2.51E-05 | 7.232508985 |
| KHSRP | 1.694361268 | 11.38751553 | 9.779150329 | 2.95E-07 | 2.52E-05 | 7.222164657 |
| AKAP7 | -1.846083575 | 8.80464571 | -9.763483634 | 3.00E-07 | 2.55E-05 | 7.20394075 |
| SULF2 | -4.312513028 | 10.35377874 | -9.760153153 | 3.01E-07 | 2.55E-05 | 7.200063383 |
| ARHGAP32 | -2.773080349 | 3.354089307 | -9.712374702 | 3.18E-07 | 2.68E-05 | 7.144313137 |
| PPM1J | -2.863973504 | 5.985850372 | -9.702842501 | 3.22E-07 | 2.69E-05 | 7.133162175 |
| P2RY12 | -3.639929518 | 6.423469114 | -9.685358111 | 3.28E-07 | 2.73E-05 | 7.112684018 |
| IL2 | -3.047361128 | 3.090151342 | -9.628241844 | 3.51E-07 | 2.90E-05 | 7.045565706 |
| NSD2 | 2.007203045 | 6.795533848 | 9.609596872 | 3.59E-07 | 2.95E-05 | 7.023581706 |
| FBXO6 | -1.971973307 | 8.462531827 | -9.568451643 | 3.76E-07 | 3.07E-05 | 6.974938574 |
| CLEC4A | -5.199309414 | 9.392306854 | -9.567573758 | 3.77E-07 | 3.07E-05 | 6.973898766 |
| FLVCR2 | -3.866847617 | 4.324953639 | -9.554275628 | 3.83E-07 | 3.07E-05 | 6.95813788 |
| TMEM106A | 2.715653364 | 7.748427368 | 9.551949812 | 3.84E-07 | 3.07E-05 | 6.955379411 |
| IL4I1 | -2.611366966 | 7.702054344 | -9.549689054 | 3.85E-07 | 3.07E-05 | 6.952697554 |
| MRPL57 | 2.074862292 | 9.673738891 | 9.548560871 | 3.85E-07 | 3.07E-05 | 6.951359027 |
| STK33 | -2.98561074 | 3.103796951 | -9.528398819 | 3.94E-07 | 3.11E-05 | 6.927415136 |
| TSHZ3 | -4.085615674 | 4.173154167 | -9.527702533 | 3.95E-07 | 3.11E-05 | 6.926587475 |
| KLRD1 | -5.549058237 | 8.617286409 | -9.496232373 | 4.10E-07 | 3.22E-05 | 6.889125779 |
| CTNS | 3.307476253 | 12.68919592 | 9.469517102 | 4.23E-07 | 3.30E-05 | 6.857241446 |
| BTN3A2 | -2.31221309 | 9.64874919 | -9.458925799 | 4.28E-07 | 3.33E-05 | 6.844579757 |
| JADE3 | 1.850385227 | 7.443525359 | 9.441409784 | 4.37E-07 | 3.38E-05 | 6.82361336 |
| GOSR1 | 1.90463563 | 9.123274249 | 9.428491135 | 4.44E-07 | 3.39E-05 | 6.808128859 |
| FAM50A | -2.002550553 | 9.443540961 | -9.42036205 | 4.48E-07 | 3.39E-05 | 6.798376019 |
| GPBAR1 | -3.205886999 | 9.497328312 | -9.420031747 | 4.48E-07 | 3.39E-05 | 6.797979589 |
| TMEM204 | -2.493964073 | 8.247935392 | -9.419344817 | 4.49E-07 | 3.39E-05 | 6.797155097 |
| REXO5 | 3.865134964 | 6.323795009 | 9.416423745 | 4.50E-07 | 3.39E-05 | 6.793648498 |
| SLC43A2 | -3.257865952 | 8.827929579 | -9.362369245 | 4.80E-07 | 3.60E-05 | 6.728592815 |
| HTRA1 | -4.617257517 | 4.897778994 | -9.322798589 | 5.03E-07 | 3.74E-05 | 6.680768307 |
| MLPH | 2.804608319 | 5.149299917 | 9.322182349 | 5.04E-07 | 3.74E-05 | 6.680022184 |
| DNASE1L3 | -4.476938141 | 6.18446074 | -9.315289523 | 5.08E-07 | 3.75E-05 | 6.67167376 |
| GPSM2 | 2.289373759 | 7.051734164 | 9.31003393 | 5.11E-07 | 3.76E-05 | 6.665304838 |
| TYMP | -1.537007381 | 13.88061066 | -9.269144612 | 5.37E-07 | 3.93E-05 | 6.615650743 |
| EMID1 | 4.578379134 | 7.648118567 | 9.256663008 | 5.45E-07 | 3.97E-05 | 6.600457245 |
| NCR3 | -3.623991866 | 8.956679161 | -9.241919853 | 5.54E-07 | 4.02E-05 | 6.582488824 |
| ASGR2 | -3.839955079 | 8.979617252 | -9.213473474 | 5.74E-07 | 4.11E-05 | 6.547751926 |
| ABLIM3 | -3.406247929 | 6.015343427 | -9.209736396 | 5.76E-07 | 4.11E-05 | 6.543181827 |
| RORC | -3.87006832 | 5.217209649 | -9.209640508 | 5.76E-07 | 4.11E-05 | 6.543064544 |
| TLR5 | -5.187773077 | 5.310154336 | -9.206531176 | 5.79E-07 | 4.11E-05 | 6.539260914 |
| RTTN | 2.837479584 | 7.594115614 | 9.202631772 | 5.81E-07 | 4.11E-05 | 6.534489285 |
| SLC6A12 | -3.23324058 | 4.489494854 | -9.196558702 | 5.86E-07 | 4.11E-05 | 6.527054442 |
| ANKRD55 | -4.440384257 | 4.310627435 | -9.195226343 | 5.87E-07 | 4.11E-05 | 6.525422781 |
| ANKRD35 | -3.133073982 | 6.9190459 | -9.179513162 | 5.98E-07 | 4.17E-05 | 6.506164994 |
| POLQ | 2.702114057 | 5.916531083 | 9.17335185 | 6.02E-07 | 4.18E-05 | 6.498606352 |
| CASP5 | -5.892772532 | 5.57285412 | -9.171398542 | 6.04E-07 | 4.18E-05 | 6.496209174 |
| GNAZ | -5.118526084 | 8.405704122 | -9.160259196 | 6.12E-07 | 4.21E-05 | 6.482530445 |
| SIRPA | -2.741133529 | 7.886464776 | -9.146392446 | 6.22E-07 | 4.25E-05 | 6.465483337 |
| FSIP1 | -2.653284453 | 4.096503463 | -9.146344942 | 6.22E-07 | 4.25E-05 | 6.465424901 |
| ADAM20 | 3.127442253 | 3.534386981 | 9.129792181 | 6.35E-07 | 4.30E-05 | 6.445047695 |
| CNNM1 | 2.606707011 | 5.834178537 | 9.129448194 | 6.35E-07 | 4.30E-05 | 6.444623908 |
| GAS2L1 | -2.085229176 | 4.872745231 | -9.109529931 | 6.51E-07 | 4.38E-05 | 6.420062436 |
| OST4 | -2.016227824 | 14.20703706 | -9.106977967 | 6.53E-07 | 4.38E-05 | 6.416912381 |
| MRPS16 | 1.694745483 | 12.08316685 | 9.101262912 | 6.57E-07 | 4.39E-05 | 6.40985528 |
| ADGRE3 | -4.492793538 | 6.285181957 | -9.07827722 | 6.76E-07 | 4.49E-05 | 6.381435097 |
| BST1 | -3.746835419 | 9.244828136 | -9.066659668 | 6.86E-07 | 4.54E-05 | 6.367048326 |
| CX3CR1 | -4.593064645 | 9.477863173 | -9.055369026 | 6.95E-07 | 4.58E-05 | 6.353051896 |
| PTCRA | -6.867934701 | 8.411402204 | -9.042688545 | 7.06E-07 | 4.63E-05 | 6.337315497 |
| CASP2 | 2.171023425 | 9.172557552 | 9.025659492 | 7.21E-07 | 4.69E-05 | 6.316154111 |
| FPR2 | -3.942062377 | 6.187049441 | -9.024784285 | 7.22E-07 | 4.69E-05 | 6.315065641 |
| RAB29 | -2.741189263 | 7.731220359 | -9.021340229 | 7.25E-07 | 4.69E-05 | 6.310781531 |
| PUDP | -2.562847218 | 7.418991364 | -9.00549773 | 7.39E-07 | 4.76E-05 | 6.291057597 |
| BEND2 | -2.57436162 | 5.644925586 | -9.002674856 | 7.42E-07 | 4.76E-05 | 6.287540143 |
| IFIT1 | -5.393209236 | 8.68232449 | -8.993401887 | 7.50E-07 | 4.79E-05 | 6.275979186 |
| MDM4 | 2.531505355 | 9.905877804 | 8.991254432 | 7.52E-07 | 4.79E-05 | 6.273300487 |
| TTC3 | 2.573039049 | 6.741407956 | 8.970699271 | 7.71E-07 | 4.88E-05 | 6.247633937 |
| MAT2A | 3.055325673 | 7.479590049 | 8.969135875 | 7.73E-07 | 4.88E-05 | 6.245679818 |
| GOLIM4 | 2.522269134 | 7.381375918 | 8.961380336 | 7.80E-07 | 4.91E-05 | 6.235981921 |
| MYZAP | -4.13588141 | 4.127869648 | -8.95560394 | 7.86E-07 | 4.92E-05 | 6.228754406 |
| LINGO2 | -3.034541708 | 4.162170363 | -8.952846178 | 7.89E-07 | 4.92E-05 | 6.225302518 |
| ICOS | -3.414550316 | 7.999004217 | -8.94063831 | 8.00E-07 | 4.96E-05 | 6.210011555 |
| TLR8 | -6.207749576 | 6.038397115 | -8.939762835 | 8.01E-07 | 4.96E-05 | 6.208914328 |
| STEAP4 | -2.518222074 | 4.088550041 | -8.898205685 | 8.44E-07 | 5.20E-05 | 6.156730591 |
| RIN1 | -3.561222198 | 8.733994313 | -8.892881413 | 8.49E-07 | 5.21E-05 | 6.150030605 |
| S100A4 | -2.403523118 | 15.04585803 | -8.856035181 | 8.89E-07 | 5.41E-05 | 6.1035749 |
| CLEC4D | -6.050916947 | 6.170794979 | -8.855978965 | 8.89E-07 | 5.41E-05 | 6.103503905 |
| GP9 | -3.722832554 | 10.81504633 | -8.840930726 | 9.06E-07 | 5.49E-05 | 6.084486196 |
| DEDD2 | -2.042046562 | 12.16219684 | -8.836705597 | 9.11E-07 | 5.49E-05 | 6.079141868 |
| SIRPG | -2.949152493 | 9.591579992 | -8.833359221 | 9.14E-07 | 5.49E-05 | 6.074907609 |
| IRF2 | -1.648888575 | 6.833440767 | -8.831229176 | 9.17E-07 | 5.49E-05 | 6.072211735 |
| AQP10 | -3.219268547 | 7.745147144 | -8.825615727 | 9.23E-07 | 5.51E-05 | 6.065104614 |
| DNMT3B | 4.974651197 | 7.548686555 | 8.822602974 | 9.27E-07 | 5.51E-05 | 6.061288706 |
| NRTN | -2.691285029 | 4.491885999 | -8.81631959 | 9.34E-07 | 5.53E-05 | 6.0533269 |
| CCR2 | -4.539952477 | 5.624071032 | -8.807841903 | 9.44E-07 | 5.56E-05 | 6.042577428 |
| MED13 | 1.518114206 | 9.673690823 | 8.788612333 | 9.67E-07 | 5.66E-05 | 6.018164094 |
| CTPS2 | 1.970294243 | 7.636138448 | 8.788380929 | 9.67E-07 | 5.66E-05 | 6.017870049 |
| GLT1D1 | -6.330629492 | 7.93211335 | -8.782484835 | 9.74E-07 | 5.67E-05 | 6.010375804 |
| SYT17 | -3.528106329 | 5.880786997 | -8.778128336 | 9.80E-07 | 5.67E-05 | 6.004835879 |
| CAVIN2 | -7.401363258 | 7.887633962 | -8.777731931 | 9.80E-07 | 5.67E-05 | 6.004331684 |
| OAS1 | -4.307431763 | 7.469322688 | -8.771431073 | 9.88E-07 | 5.68E-05 | 5.996315043 |
| HK3 | -4.792170539 | 7.211010618 | -8.770217551 | 9.89E-07 | 5.68E-05 | 5.994770538 |
| PEA15 | -2.738348221 | 7.52596259 | -8.754773965 | 1.01E-06 | 5.73E-05 | 5.975099868 |
| U2AF1 | 1.585771628 | 13.93916546 | 8.751610958 | 1.01E-06 | 5.73E-05 | 5.971067694 |
| SAMD3 | -4.888240137 | 7.474410241 | -8.751440251 | 1.01E-06 | 5.73E-05 | 5.970850045 |
| NAAA | -2.127814122 | 8.69769738 | -8.751353138 | 1.01E-06 | 5.73E-05 | 5.970738975 |
| MNDA | -4.583085449 | 10.45933651 | -8.743090997 | 1.02E-06 | 5.75E-05 | 5.960200734 |
| CDH23 | -2.579237321 | 3.690541593 | -8.741574529 | 1.03E-06 | 5.75E-05 | 5.958265639 |
| IL13RA1 | -2.743885089 | 8.96283617 | -8.734014704 | 1.04E-06 | 5.79E-05 | 5.9486149 |
| EHMT1 | 2.534636578 | 4.263149916 | 8.718295776 | 1.06E-06 | 5.88E-05 | 5.928527085 |
| YIPF4 | 1.875651457 | 6.28201583 | 8.715027367 | 1.06E-06 | 5.88E-05 | 5.924346644 |
| PPP1R17 | -3.288041644 | 5.820322952 | -8.700929719 | 1.08E-06 | 5.97E-05 | 5.90630083 |
| C1orf210 | 2.44388668 | 4.312444877 | 8.695830141 | 1.09E-06 | 5.97E-05 | 5.899767352 |
| CXCL16 | -1.571622092 | 8.275214547 | -8.694419779 | 1.09E-06 | 5.97E-05 | 5.897959888 |
| RGS2 | -3.045701006 | 12.58154538 | -8.692479249 | 1.09E-06 | 5.97E-05 | 5.895472601 |
| PET117 | -1.913533638 | 10.21428801 | -8.675745124 | 1.11E-06 | 6.04E-05 | 5.874005235 |
| RANGAP1 | 1.574230009 | 13.2348856 | 8.675414952 | 1.11E-06 | 6.04E-05 | 5.873581345 |
| NCKAP1 | -1.883684915 | 4.507493754 | -8.674383871 | 1.12E-06 | 6.04E-05 | 5.872257511 |
| CPED1 | -2.901092447 | 3.744585992 | -8.668366028 | 1.12E-06 | 6.05E-05 | 5.864528541 |
| CDC42EP1 | -3.033720495 | 8.669972677 | -8.666818614 | 1.13E-06 | 6.05E-05 | 5.862540446 |
| SEMA4F | -2.1151209 | 4.376014798 | -8.636116498 | 1.17E-06 | 6.27E-05 | 5.823036658 |
| CRIP3 | -1.855362194 | 6.356861659 | -8.630530349 | 1.18E-06 | 6.29E-05 | 5.815837151 |
| HFE | -2.115276686 | 5.445254813 | -8.621754621 | 1.19E-06 | 6.33E-05 | 5.804519447 |
| RBM47 | -2.477622908 | 5.303625314 | -8.620415046 | 1.19E-06 | 6.33E-05 | 5.802791051 |
| TLR10 | -1.877020273 | 5.576904302 | -8.607318342 | 1.21E-06 | 6.41E-05 | 5.785881794 |
| SMARCD3 | -4.089639835 | 10.31052239 | -8.594850157 | 1.23E-06 | 6.48E-05 | 5.769765198 |
| LY6G6F | -4.220550165 | 7.167571612 | -8.591326481 | 1.24E-06 | 6.48E-05 | 5.765207102 |
| LAYN | -2.139016848 | 5.199520311 | -8.587279269 | 1.25E-06 | 6.48E-05 | 5.759969964 |
| KCNJ2 | -4.778483032 | 4.22504085 | -8.585970638 | 1.25E-06 | 6.48E-05 | 5.758276167 |
| ZBTB38 | -3.205341533 | 4.79110142 | -8.585525428 | 1.25E-06 | 6.48E-05 | 5.757699873 |
| VNN2 | -4.917733271 | 9.312473031 | -8.564607142 | 1.28E-06 | 6.63E-05 | 5.730596103 |
| BEND3 | 1.691286257 | 7.246919612 | 8.55750711 | 1.29E-06 | 6.67E-05 | 5.721384811 |
| SPARC | -3.292024311 | 7.412604718 | -8.543422169 | 1.32E-06 | 6.74E-05 | 5.703093858 |
| BFSP1 | -4.209110832 | 6.151393333 | -8.541951305 | 1.32E-06 | 6.74E-05 | 5.701182408 |
| GIMAP7 | -5.457672298 | 10.02945055 | -8.539668529 | 1.32E-06 | 6.74E-05 | 5.698215334 |
| NPIPB12 | 3.424050208 | 14.59497024 | 8.538619283 | 1.33E-06 | 6.74E-05 | 5.696851352 |
| RCC2 | 1.617647994 | 9.59001436 | 8.519804324 | 1.36E-06 | 6.88E-05 | 5.672370326 |
| RNF13 | -2.006726156 | 9.78624941 | -8.508880388 | 1.38E-06 | 6.96E-05 | 5.658137313 |
| IPCEF1 | -2.557393795 | 5.352700009 | -8.501102437 | 1.39E-06 | 7.00E-05 | 5.647994582 |
| CLEC7A | -6.469187226 | 7.581277717 | -8.497120376 | 1.40E-06 | 7.01E-05 | 5.642799032 |
| NUPR2 | -2.665505514 | 5.600793211 | -8.485355321 | 1.42E-06 | 7.10E-05 | 5.627437626 |
| PLAU | 2.903652673 | 7.204899625 | 8.477791949 | 1.43E-06 | 7.12E-05 | 5.617553528 |
| HLTF | 1.926547952 | 7.213107642 | 8.477388904 | 1.43E-06 | 7.12E-05 | 5.617026622 |
| FCRL6 | -3.346636929 | 8.424594077 | -8.43246268 | 1.52E-06 | 7.50E-05 | 5.558171692 |
| PSAP | -2.396577774 | 12.21110207 | -8.432195255 | 1.52E-06 | 7.50E-05 | 5.55782063 |
| RABL2B | 1.596163018 | 7.514046085 | 8.421856182 | 1.54E-06 | 7.58E-05 | 5.544241388 |
| ARMC3 | -2.823721562 | 3.075562367 | -8.419182875 | 1.55E-06 | 7.58E-05 | 5.540728195 |
| ROBO2 | 1.603720036 | 2.718858643 | 8.410232748 | 1.56E-06 | 7.64E-05 | 5.528959889 |
| CXCR3 | -2.316434267 | 7.773941121 | -8.406528255 | 1.57E-06 | 7.65E-05 | 5.524086112 |
| SH2D1B | -4.750543909 | 8.04912532 | -8.402277536 | 1.58E-06 | 7.67E-05 | 5.518491657 |
| HORMAD1 | -1.462867457 | 5.55507079 | -8.393919429 | 1.60E-06 | 7.73E-05 | 5.507485018 |
| XKR8 | -1.934384499 | 11.88646335 | -8.374851228 | 1.64E-06 | 7.88E-05 | 5.48234282 |
| RNF166 | -1.846011259 | 7.678247682 | -8.370444096 | 1.65E-06 | 7.88E-05 | 5.476525572 |
| TMEM181 | 1.730187206 | 8.677114237 | 8.370294229 | 1.65E-06 | 7.88E-05 | 5.476327711 |
| MFAP3L | -5.462273524 | 5.947336707 | -8.369432615 | 1.65E-06 | 7.88E-05 | 5.475190124 |
| IARS | 1.583418487 | 9.434838415 | 8.350103129 | 1.69E-06 | 8.05E-05 | 5.449645729 |
| SAPCD1 | 2.491002264 | 8.521907881 | 8.346542291 | 1.70E-06 | 8.06E-05 | 5.444935046 |
| XRCC2 | 1.73095907 | 6.558416416 | 8.33403685 | 1.73E-06 | 8.16E-05 | 5.428379201 |
| BZW2 | 1.598618927 | 11.26782124 | 8.331228686 | 1.73E-06 | 8.16E-05 | 5.42465888 |
| ZNF483 | -2.525514546 | 5.398362367 | -8.329882761 | 1.73E-06 | 8.16E-05 | 5.422875428 |
| CDC6 | 2.214808283 | 5.22107093 | 8.319473272 | 1.76E-06 | 8.25E-05 | 5.409074613 |
| IRF2BP2 | 1.80667063 | 7.790580637 | 8.303971487 | 1.79E-06 | 8.38E-05 | 5.388497984 |
| SYCE2 | 2.564273667 | 6.596135876 | 8.303056838 | 1.80E-06 | 8.38E-05 | 5.387282989 |
| HECTD4 | 2.124213029 | 8.556969239 | 8.296551956 | 1.81E-06 | 8.42E-05 | 5.37863913 |
| ACOT8 | -1.478958356 | 7.54523857 | -8.288009485 | 1.83E-06 | 8.49E-05 | 5.367279814 |
| ATXN3 | 1.77826926 | 7.087956226 | 8.284893337 | 1.84E-06 | 8.50E-05 | 5.363133907 |
| MYLK | -3.468952455 | 5.998197211 | -8.281913 | 1.85E-06 | 8.50E-05 | 5.359167579 |
| PPIAL4G | 2.735699895 | 9.176041344 | 8.276337138 | 1.86E-06 | 8.54E-05 | 5.351744124 |
| CRB3 | -2.841021353 | 4.698216678 | -8.264030228 | 1.89E-06 | 8.65E-05 | 5.335345771 |
| SUSD3 | -2.250207202 | 9.533091897 | -8.262340919 | 1.89E-06 | 8.65E-05 | 5.333093401 |
| ARHGAP31 | -3.579299848 | 4.591338987 | -8.251832991 | 1.92E-06 | 8.74E-05 | 5.319075225 |
| RLIM | 2.19401557 | 10.09192119 | 8.247665815 | 1.93E-06 | 8.76E-05 | 5.313512221 |
| MGAT4A | -2.949660389 | 9.853575772 | -8.238572085 | 1.95E-06 | 8.84E-05 | 5.301365065 |
| FOLR2 | -1.798998038 | 7.983141648 | -8.231291437 | 1.97E-06 | 8.90E-05 | 5.291632447 |
| N4BP2 | 1.739793003 | 9.273088038 | 8.2275361 | 1.98E-06 | 8.91E-05 | 5.286609839 |
| DYRK3 | 3.900844911 | 4.605566456 | 8.219545642 | 2.00E-06 | 8.95E-05 | 5.275917158 |
| ZNF491 | 1.834752722 | 4.578221348 | 8.218304918 | 2.01E-06 | 8.95E-05 | 5.274256138 |
| LY96 | -4.101443271 | 10.44195092 | -8.216707895 | 2.01E-06 | 8.95E-05 | 5.272117844 |
| UNC45B | -1.999687186 | 2.645331065 | -8.215627042 | 2.01E-06 | 8.95E-05 | 5.270670486 |
| MAFG | 2.188019972 | 8.645479998 | 8.210925659 | 2.03E-06 | 8.98E-05 | 5.264373236 |
| GNG11 | -7.3885619 | 10.36771096 | -8.208097646 | 2.03E-06 | 8.99E-05 | 5.260583951 |
| DKC1 | 1.852807798 | 10.48038707 | 8.205793772 | 2.04E-06 | 8.99E-05 | 5.257496236 |
| SH3BGRL2 | -5.432383028 | 5.672532484 | -8.194867724 | 2.07E-06 | 9.09E-05 | 5.24284393 |
| CLIC5 | -1.427573897 | 4.497810885 | -8.191259947 | 2.08E-06 | 9.09E-05 | 5.238002506 |
| CD3D | -3.889556236 | 13.19299097 | -8.190568021 | 2.08E-06 | 9.09E-05 | 5.237073798 |
| LRRK2 | -4.186876137 | 4.314167149 | -8.188652138 | 2.09E-06 | 9.09E-05 | 5.234501978 |
| ECE1 | -3.42087141 | 6.332662206 | -8.183830752 | 2.10E-06 | 9.09E-05 | 5.228027896 |
| SPTA1 | 6.210926676 | 5.353521953 | 8.18370668 | 2.10E-06 | 9.09E-05 | 5.227861257 |
| MAGEE1 | -4.493161732 | 4.955907475 | -8.17870933 | 2.11E-06 | 9.13E-05 | 5.221147781 |
| ATP5F1E | -2.034663767 | 12.73235537 | -8.155608724 | 2.18E-06 | 9.38E-05 | 5.190074078 |
| CD8B | -4.056832053 | 9.622170304 | -8.151737193 | 2.19E-06 | 9.39E-05 | 5.184859829 |
| NPIPB13 | 2.856461602 | 12.95321953 | 8.148514495 | 2.20E-06 | 9.39E-05 | 5.180518022 |
| QSER1 | 1.975147426 | 6.763238046 | 8.148509073 | 2.20E-06 | 9.39E-05 | 5.180510716 |
| SRSF6 | 1.556533027 | 11.92683498 | 8.14597042 | 2.21E-06 | 9.39E-05 | 5.177089584 |
| C6orf226 | -1.876763573 | 10.48586438 | -8.143599181 | 2.21E-06 | 9.40E-05 | 5.173893339 |
| SMARCC1 | 1.528923923 | 9.72041129 | 8.137975324 | 2.23E-06 | 9.44E-05 | 5.166310027 |
| EIF3B | 1.701578972 | 10.79012241 | 8.134183551 | 2.24E-06 | 9.46E-05 | 5.161194913 |
| HS1BP3 | -1.46160808 | 6.009137488 | -8.130362248 | 2.25E-06 | 9.48E-05 | 5.156038154 |
| NPIPB5 | 2.90832753 | 10.63267679 | 8.127761627 | 2.26E-06 | 9.49E-05 | 5.152527637 |
| THEMIS | -5.829439997 | 5.997591834 | -8.106604352 | 2.33E-06 | 9.73E-05 | 5.12393665 |
| GPR149 | 2.027976796 | 4.923088904 | 8.097104462 | 2.35E-06 | 9.83E-05 | 5.111080779 |
| FRMD3 | -3.270223948 | 5.392669049 | -8.092562215 | 2.37E-06 | 9.86E-05 | 5.104929934 |
| PRKDC | 1.684437482 | 6.828114965 | 8.090629385 | 2.38E-06 | 9.86E-05 | 5.102311827 |
| PTGDR | -1.696974891 | 6.411515127 | -8.081677126 | 2.40E-06 | 9.95E-05 | 5.090179496 |
| UPK3A | -2.194253682 | 4.876183965 | -8.070252728 | 2.44E-06 | 0.00010075 | 5.074682308 |
| LY6G5B | 2.40610651 | 6.888820843 | 8.058512453 | 2.48E-06 | 0.000102057 | 5.058739613 |
| UBTD1 | -2.557107701 | 8.744973543 | -8.048183548 | 2.51E-06 | 0.000103189 | 5.044699206 |
| STX18 | -4.81190833 | 6.639650217 | -8.029207699 | 2.58E-06 | 0.000105266 | 5.018869845 |
| MAP7 | 3.429642998 | 7.588375564 | 8.02917903 | 2.58E-06 | 0.000105266 | 5.018830788 |
| PIWIL4 | 2.159421817 | 8.481598558 | 8.023214747 | 2.60E-06 | 0.000105519 | 5.010703019 |
| CCL4L2 | -2.055602357 | 6.350562868 | -8.021619469 | 2.60E-06 | 0.000105519 | 5.008528311 |
| JAKMIP1 | -4.413665209 | 5.868708493 | -8.021049361 | 2.61E-06 | 0.000105519 | 5.007751053 |
| CYP2W1 | 1.609937725 | 17.00110736 | 8.019338743 | 2.61E-06 | 0.000105519 | 5.005418633 |
| BCL2L2 | 2.05303642 | 8.144174118 | 8.013689609 | 2.63E-06 | 0.000105893 | 4.99771345 |
| PLBD1 | -6.239018175 | 10.43753617 | -8.012709199 | 2.63E-06 | 0.000105893 | 4.996375801 |
| MT-CYB | 1.222997109 | 17.01201948 | 8.004029155 | 2.67E-06 | 0.000106845 | 4.984527682 |
| DBNDD2 | -1.928031414 | 10.10681173 | -7.99819419 | 2.69E-06 | 0.000107398 | 4.976557714 |
| PTGDS | -3.223940246 | 10.74530504 | -7.991107904 | 2.71E-06 | 0.000108135 | 4.966872795 |
| HIC2 | 1.652008102 | 6.598846098 | 7.97286187 | 2.78E-06 | 0.00011052 | 4.941906536 |
| UBR5 | 1.81018215 | 8.080391478 | 7.962995758 | 2.82E-06 | 0.000111699 | 4.928389094 |
| LATS2 | 2.255237365 | 7.457388698 | 7.959849984 | 2.83E-06 | 0.000111878 | 4.924076518 |
| GIMAP2 | -2.788440762 | 10.18071057 | -7.955434487 | 2.84E-06 | 0.000112052 | 4.918021152 |
| FUT4 | 3.868373172 | 9.427004642 | 7.954116632 | 2.85E-06 | 0.000112052 | 4.916213381 |
| CCR3 | -2.351857687 | 5.307204418 | -7.952879359 | 2.85E-06 | 0.000112052 | 4.914515949 |
| STEAP1B | -3.441218162 | 3.78705203 | -7.947481903 | 2.88E-06 | 0.000112576 | 4.907108834 |
| CASP1 | -2.921614044 | 11.49687399 | -7.935519228 | 2.92E-06 | 0.000114106 | 4.890678881 |
| EPSTI1 | -3.697124398 | 7.039957866 | -7.930768719 | 2.94E-06 | 0.000114542 | 4.884149331 |
| RNF103-CHMP3 | -1.922228978 | 10.02666071 | -7.927983894 | 2.95E-06 | 0.000114677 | 4.88032027 |
| GZMM | -2.323003035 | 10.86056709 | -7.922994454 | 2.97E-06 | 0.000115154 | 4.873457462 |
| GNGT2 | -4.052998591 | 7.250408511 | -7.918671741 | 2.99E-06 | 0.000115531 | 4.867509158 |
| PTGIR | -3.073609061 | 8.594939069 | -7.905580069 | 3.04E-06 | 0.000117288 | 4.849479763 |
| EPHX2 | -4.373576872 | 7.51206095 | -7.892274891 | 3.10E-06 | 0.000118669 | 4.831133986 |
| STMN1 | 2.774556012 | 11.82106097 | 7.891718062 | 3.10E-06 | 0.000118669 | 4.830365714 |
| CNTROB | 1.261151263 | 8.596652944 | 7.89126586 | 3.10E-06 | 0.000118669 | 4.829741769 |
| GYG1 | -1.574469544 | 7.555602894 | -7.885150632 | 3.13E-06 | 0.000119352 | 4.821301466 |
| CHERP | 1.502108949 | 7.65685797 | 7.881628657 | 3.14E-06 | 0.00011962 | 4.816438236 |
| RASSF3 | -2.175304033 | 8.303044734 | -7.877448349 | 3.16E-06 | 0.000119996 | 4.810663907 |
| IKBKB | 2.767905342 | 7.746636915 | 7.873101484 | 3.18E-06 | 0.000120402 | 4.804657146 |
| ADSSL1 | -2.197058744 | 6.622073086 | -7.863262024 | 3.22E-06 | 0.000121711 | 4.791051474 |
| CA4 | -2.431823425 | 5.288485933 | -7.854099007 | 3.26E-06 | 0.000122632 | 4.778370038 |
| GABRA3 | -1.647249069 | 6.374950563 | -7.854014263 | 3.26E-06 | 0.000122632 | 4.778252704 |
| MME | -3.096151041 | 6.483790427 | -7.851660222 | 3.27E-06 | 0.000122719 | 4.774992998 |
| C1QTNF4 | 5.567231112 | 7.407501606 | 7.84920402 | 3.28E-06 | 0.000122824 | 4.771591072 |
| EPDR1 | 4.322081558 | 7.87813241 | 7.843930932 | 3.31E-06 | 0.000122962 | 4.76428505 |
| PGRMC1 | -2.567931571 | 10.87003276 | -7.843639874 | 3.31E-06 | 0.000122962 | 4.763881676 |
| C3orf18 | -2.241439846 | 8.765606218 | -7.842931253 | 3.31E-06 | 0.000122962 | 4.762899563 |
| USP33 | 1.93274217 | 14.97758213 | 7.839577864 | 3.33E-06 | 0.000123221 | 4.75825106 |
| C7orf50 | -2.034044634 | 9.55630559 | -7.836085287 | 3.34E-06 | 0.000123504 | 4.753408082 |
| KIR2DL1 | -2.866669564 | 6.954874537 | -7.825381037 | 3.39E-06 | 0.000125006 | 4.738555309 |
| CKAP5 | 1.521803634 | 8.128433227 | 7.820139015 | 3.42E-06 | 0.000125594 | 4.731276337 |
| VOPP1 | -2.773394443 | 9.306837517 | -7.808670279 | 3.47E-06 | 0.000127258 | 4.715338765 |
| PERP | -3.290568288 | 5.200025462 | -7.806187481 | 3.48E-06 | 0.00012738 | 4.711886312 |
| THAP12 | -1.630549008 | 8.291726792 | -7.801679101 | 3.50E-06 | 0.000127855 | 4.70561516 |
| MYL6 | -1.401166177 | 14.62781494 | -7.799636939 | 3.51E-06 | 0.000127902 | 4.702773654 |
| TCF7 | -3.836638253 | 7.347034048 | -7.788768814 | 3.56E-06 | 0.000129497 | 4.687642504 |
| ZNF587 | 2.641841591 | 9.123267604 | 7.781137797 | 3.60E-06 | 0.000130538 | 4.677009133 |
| CMKLR1 | -3.731087977 | 6.193661326 | -7.764927199 | 3.68E-06 | 0.000133114 | 4.654395722 |
| CST5 | -3.208898017 | 10.50047473 | -7.763310033 | 3.69E-06 | 0.000133114 | 4.652137953 |
| MMD | -3.58680825 | 9.912577201 | -7.755677115 | 3.73E-06 | 0.00013419 | 4.641476877 |
| NHSL2 | -2.412835601 | 5.156140142 | -7.750043449 | 3.76E-06 | 0.000134777 | 4.633603377 |
| PTBP2 | 2.692325872 | 8.523771265 | 7.749012717 | 3.76E-06 | 0.000134777 | 4.632162404 |
| ISG15 | -3.139450743 | 12.51134675 | -7.745334311 | 3.78E-06 | 0.000135137 | 4.627018833 |
| HMGA1 | 1.591286753 | 12.92529698 | 7.736784768 | 3.83E-06 | 0.000136405 | 4.615057121 |
| PAICS | 1.364620238 | 9.388270235 | 7.722849718 | 3.90E-06 | 0.000138598 | 4.595540246 |
| ZNF738 | 2.15969475 | 7.622508899 | 7.721459477 | 3.91E-06 | 0.000138598 | 4.593591752 |
| RIF1 | 1.868570975 | 7.90265955 | 7.717059136 | 3.93E-06 | 0.000138598 | 4.587422792 |
| HERC6 | -2.103615999 | 4.392190832 | -7.716723025 | 3.93E-06 | 0.000138598 | 4.586951487 |
| DYNLT3 | -2.286202548 | 7.325362911 | -7.71660045 | 3.93E-06 | 0.000138598 | 4.586779603 |
| S1PR5 | -3.988190123 | 7.522467672 | -7.71276802 | 3.95E-06 | 0.000139005 | 4.581404542 |
| KLRC1 | -5.093685796 | 8.290360909 | -7.700956529 | 4.02E-06 | 0.000140949 | 4.5648267 |
| TBC1D12 | -3.186426759 | 3.676347046 | -7.686202407 | 4.10E-06 | 0.000143372 | 4.544093332 |
| YWHAG | 2.18966207 | 11.88220235 | 7.685180978 | 4.11E-06 | 0.000143372 | 4.542656911 |
| C5orf24 | 1.796189157 | 8.543357234 | 7.682644412 | 4.12E-06 | 0.000143543 | 4.539089192 |
| PHLPP1 | 3.969924391 | 4.309127239 | 7.680392449 | 4.13E-06 | 0.000143657 | 4.53592107 |
| GZMH | -4.551092292 | 13.58007903 | -7.676140607 | 4.16E-06 | 0.000144169 | 4.529937665 |
| DMKN | -2.388574886 | 7.546588037 | -7.66008445 | 4.25E-06 | 0.000147055 | 4.507321417 |
| KIAA1147 | 1.278010788 | 8.941674321 | 7.657394003 | 4.27E-06 | 0.000147265 | 4.503528444 |
| PIGN | 2.761244206 | 12.74382685 | 7.655137508 | 4.28E-06 | 0.000147388 | 4.500346527 |
| CYP1B1 | -3.367212949 | 6.289988472 | -7.652178492 | 4.30E-06 | 0.000147496 | 4.496172968 |
| PYGM | -1.605792526 | 5.471618045 | -7.651316268 | 4.30E-06 | 0.000147496 | 4.494956625 |
| MID2 | -2.99684578 | 4.357098808 | -7.641753378 | 4.36E-06 | 0.000149117 | 4.481459714 |
| HPR | -2.622502701 | 9.482214229 | -7.638434368 | 4.38E-06 | 0.000149341 | 4.476772527 |
| ZFAND2A | -2.171608117 | 9.823712354 | -7.63740113 | 4.39E-06 | 0.000149341 | 4.475313068 |
| ETV6 | 2.314886919 | 8.705344578 | 7.626858262 | 4.45E-06 | 0.000150897 | 4.460413203 |
| SLC22A16 | 2.841559222 | 9.303665323 | 7.6252939 | 4.46E-06 | 0.000150897 | 4.458201107 |
| IRGQ | 3.609640706 | 6.78099539 | 7.625029981 | 4.46E-06 | 0.000150897 | 4.45782788 |
| BLOC1S2 | -1.448585564 | 8.951437794 | -7.617039362 | 4.51E-06 | 0.000152233 | 4.446523431 |
| CPLANE1 | 1.53256009 | 7.447782772 | 7.612380625 | 4.54E-06 | 0.000152877 | 4.439928789 |
| WNT11 | -2.905735981 | 4.192656272 | -7.609337938 | 4.56E-06 | 0.000153181 | 4.435620203 |
| TMEM184A | 2.164467543 | 8.969406001 | 7.601625361 | 4.61E-06 | 0.000154483 | 4.424693405 |
| CHI3L1 | -5.662546212 | 6.191227962 | -7.594051811 | 4.66E-06 | 0.000155767 | 4.41395599 |
| TCEA3 | -4.978494448 | 6.430612193 | -7.584175141 | 4.72E-06 | 0.000157567 | 4.399942021 |
| BEX2 | -3.492805446 | 9.88645524 | -7.582039686 | 4.74E-06 | 0.000157687 | 4.396910351 |
| APOBEC3B | -3.046584406 | 9.119684356 | -7.569884505 | 4.82E-06 | 0.000160018 | 4.379642432 |
| GPR68 | -3.21024388 | 7.145144853 | -7.566552191 | 4.84E-06 | 0.000160305 | 4.374905083 |
| SLC38A2 | 1.297616278 | 6.907556489 | 7.563728993 | 4.86E-06 | 0.000160305 | 4.370890372 |
| TMEM191B | -1.766486899 | 8.345066259 | -7.562958755 | 4.86E-06 | 0.000160305 | 4.369794879 |
| PAN3 | 2.837791363 | 7.803186276 | 7.562308509 | 4.87E-06 | 0.000160305 | 4.368869986 |
| ATAD5 | 3.03758982 | 6.232315946 | 7.555849526 | 4.91E-06 | 0.000161399 | 4.359679882 |
| IL23A | -2.84003812 | 7.758865085 | -7.549944566 | 4.95E-06 | 0.000162067 | 4.351273261 |
| FCGRT | -2.155250793 | 13.55603616 | -7.549767308 | 4.95E-06 | 0.000162067 | 4.351020836 |
| ZNF846 | 2.874266637 | 5.5031707 | 7.54427493 | 4.99E-06 | 0.000162958 | 4.343197341 |
| EFHC1 | 1.957105961 | 4.068814576 | 7.541534569 | 5.01E-06 | 0.000163229 | 4.339292411 |
| C8orf89 | -2.794925597 | 3.634536472 | -7.537997346 | 5.03E-06 | 0.000163683 | 4.334250513 |
| MPIG6B | -2.426677204 | 7.928263984 | -7.53457718 | 5.06E-06 | 0.000163755 | 4.329373897 |
| RTKN | 1.821257166 | 5.440715036 | 7.534541252 | 5.06E-06 | 0.000163755 | 4.329322662 |
| CYBB | -4.498260656 | 10.66731309 | -7.533076906 | 5.07E-06 | 0.000163755 | 4.327234259 |
| OAZ3 | -2.138944283 | 4.563197489 | -7.531524466 | 5.08E-06 | 0.00016376 | 4.32501991 |
| DMRTC1 | -4.189922901 | 4.777543767 | -7.516483635 | 5.19E-06 | 0.000166875 | 4.303549706 |
| TTF2 | 2.221069831 | 4.943782764 | 7.511553639 | 5.22E-06 | 0.000167643 | 4.296505831 |
| OSTF1 | -1.885749139 | 11.48149685 | -7.508909952 | 5.24E-06 | 0.000167643 | 4.292727264 |
| IL1RN | -3.180671465 | 8.918332319 | -7.508643874 | 5.24E-06 | 0.000167643 | 4.292346913 |
| 1-Mar | -4.160121747 | 6.061324196 | -7.497756859 | 5.33E-06 | 0.000169854 | 4.276776213 |
| CD8B2 | -2.053970311 | 6.167952944 | -7.495266792 | 5.34E-06 | 0.000170088 | 4.273212695 |
| THRB | -1.676081886 | 4.272281101 | -7.493234418 | 5.36E-06 | 0.000170214 | 4.270303572 |
| GAA | -1.763645548 | 12.50921633 | -7.484415565 | 5.43E-06 | 0.000170819 | 4.257674008 |
| ZBTB7B | -1.924691368 | 8.571535895 | -7.484304028 | 5.43E-06 | 0.000170819 | 4.257514208 |
| ANKRD36B | 2.536837268 | 9.335025867 | 7.48309572 | 5.44E-06 | 0.000170819 | 4.25578296 |
| CEACAM3 | -4.061031527 | 5.027612936 | -7.482869716 | 5.44E-06 | 0.000170819 | 4.255459122 |
| EXTL2 | 1.546353729 | 5.690422967 | 7.482477475 | 5.44E-06 | 0.000170819 | 4.25489707 |
| MAN1C1 | -2.926408044 | 6.546009391 | -7.481759026 | 5.45E-06 | 0.000170819 | 4.253867535 |
| ZNF343 | -1.216684321 | 4.879820755 | -7.479719553 | 5.46E-06 | 0.000170954 | 4.250944603 |
| DAPK2 | -2.447868034 | 6.011916392 | -7.476523763 | 5.49E-06 | 0.000171366 | 4.246363351 |
| CADM1 | -2.002912125 | 3.661234844 | -7.473805429 | 5.51E-06 | 0.000171666 | 4.242465482 |
| ABCA9 | 2.84236369 | 13.25320712 | 7.469106487 | 5.54E-06 | 0.000172445 | 4.235725272 |
| CREBL2 | -3.318694444 | 6.77336869 | -7.467452445 | 5.56E-06 | 0.00017249 | 4.233352001 |
| SLX4IP | 2.690004874 | 8.777130152 | 7.465424819 | 5.57E-06 | 0.000172628 | 4.230442205 |
| FCN1 | -6.092964425 | 12.58605662 | -7.461529776 | 5.60E-06 | 0.000173218 | 4.224850996 |
| ADGRA3 | 4.129674984 | 5.241765082 | 7.458661687 | 5.62E-06 | 0.000173305 | 4.220732659 |
| SLC49A3 | -2.634247171 | 7.417392271 | -7.456817453 | 5.64E-06 | 0.000173305 | 4.218083916 |
| BSDC1 | 2.235915973 | 9.818737424 | 7.456772473 | 5.64E-06 | 0.000173305 | 4.21801931 |
| DBNL | -1.54388259 | 9.595946782 | -7.455373573 | 5.65E-06 | 0.000173305 | 4.216009859 |
| NRBF2 | -1.823304282 | 7.532768425 | -7.449020808 | 5.70E-06 | 0.000174504 | 4.206881159 |
| GIPC3 | 1.711377852 | 4.757198473 | 7.442601632 | 5.75E-06 | 0.000175728 | 4.197651584 |
| HABP2 | 1.322302496 | 2.294863608 | 7.437561829 | 5.79E-06 | 0.000176621 | 4.190401454 |
| CCR4 | -1.94938983 | 4.653049635 | -7.429683556 | 5.86E-06 | 0.000178139 | 4.179061207 |
| ZNF70 | 2.56320122 | 5.715230828 | 7.428613721 | 5.87E-06 | 0.000178139 | 4.177520615 |
| RCN3 | -2.029728965 | 5.700152388 | -7.415101842 | 5.98E-06 | 0.000181195 | 4.158050021 |
| HIGD2A | -1.547161346 | 11.70381573 | -7.413572521 | 5.99E-06 | 0.000181223 | 4.155844741 |
| SURF2 | -1.876416288 | 9.94155117 | -7.406790615 | 6.05E-06 | 0.000182253 | 4.14606148 |
| PIKFYVE | 1.66426786 | 7.886201764 | 7.406718797 | 6.05E-06 | 0.000182253 | 4.145957847 |
| RFC3 | 1.502292881 | 6.997937342 | 7.395285483 | 6.15E-06 | 0.000184294 | 4.129450693 |
| CXCR6 | -3.649601793 | 4.950257884 | -7.394711016 | 6.15E-06 | 0.000184294 | 4.128620831 |
| TICRR | 3.270516074 | 3.503938989 | 7.394506997 | 6.16E-06 | 0.000184294 | 4.128326099 |
| PPA1 | -1.652790857 | 11.26451348 | -7.393198539 | 6.17E-06 | 0.000184294 | 4.12643573 |
| CHD8 | 1.719074868 | 10.03763987 | 7.38652504 | 6.23E-06 | 0.000185671 | 4.116790768 |
| FAM69A | -2.613810061 | 4.90153192 | -7.384745384 | 6.24E-06 | 0.000185772 | 4.114217693 |
| PHRF1 | 1.678214504 | 12.44101993 | 7.383129205 | 6.26E-06 | 0.000185831 | 4.111880612 |
| MPHOSPH6 | -2.15681908 | 7.289575581 | -7.373241056 | 6.34E-06 | 0.000188076 | 4.097574216 |
| SELENOM | -3.932487984 | 7.73924676 | -7.369602819 | 6.38E-06 | 0.000188676 | 4.092307046 |
| STX11 | -2.317876188 | 8.767204435 | -7.367881694 | 6.39E-06 | 0.000188767 | 4.089814711 |
| CHD6 | 1.507772797 | 7.141058616 | 7.365464074 | 6.41E-06 | 0.000189045 | 4.086313124 |
| TMA16 | 1.498544908 | 8.64628831 | 7.360499776 | 6.46E-06 | 0.000189982 | 4.079120578 |
| SLC19A3 | 2.656274777 | 5.466851285 | 7.359222509 | 6.47E-06 | 0.000189982 | 4.077269469 |
| MAF | -2.293194328 | 9.912675821 | -7.352658586 | 6.53E-06 | 0.000191384 | 4.067753114 |
| CST7 | -2.947767953 | 9.466976674 | -7.348775633 | 6.57E-06 | 0.000191735 | 4.062120904 |
| KDM1A | 1.567247555 | 8.44570254 | 7.348467784 | 6.57E-06 | 0.000191735 | 4.061674285 |
| IL2RB | -4.360539707 | 9.982761238 | -7.347276133 | 6.58E-06 | 0.000191735 | 4.059945345 |
| ID1 | -3.374136583 | 8.869032687 | -7.341597299 | 6.63E-06 | 0.000192913 | 4.051703439 |
| CXCL9 | -3.612697286 | 5.218675834 | -7.338178795 | 6.67E-06 | 0.00019338 | 4.046739952 |
| ATOH8 | -3.327698033 | 6.522924503 | -7.337180304 | 6.68E-06 | 0.00019338 | 4.045289901 |
| SUZ12 | 1.783716307 | 10.69776674 | 7.335836472 | 6.69E-06 | 0.00019338 | 4.043338119 |
| SLC11A2 | 1.266458693 | 8.983216457 | 7.334006262 | 6.71E-06 | 0.000193514 | 4.040679533 |
| NR2F6 | 1.680912857 | 10.09371798 | 7.332169889 | 6.72E-06 | 0.000193649 | 4.038011541 |
| GLB1L3 | -2.106631451 | 4.452897911 | -7.322253857 | 6.82E-06 | 0.00019602 | 4.023597132 |
| CRTAM | -3.789798108 | 6.271995974 | -7.317996682 | 6.86E-06 | 0.000196835 | 4.017404653 |
| DDX52 | 3.006571891 | 5.874903783 | 7.316027036 | 6.88E-06 | 0.000197013 | 4.014538786 |
| KRTAP20-4 | 1.614284345 | 4.266520575 | 7.314047341 | 6.90E-06 | 0.000197195 | 4.011657773 |
| ATP6V1E2 | -2.141128081 | 7.654306096 | -7.306387266 | 6.97E-06 | 0.00019861 | 4.000505251 |
| SMPD3 | -3.225614026 | 7.202593137 | -7.306349414 | 6.97E-06 | 0.00019861 | 4.000450122 |
| JARID2 | 1.460942974 | 11.41217088 | 7.304394576 | 6.99E-06 | 0.00019861 | 3.997602743 |
| ATP6V0E1 | -1.595568949 | 10.26410201 | -7.30372232 | 7.00E-06 | 0.00019861 | 3.99662343 |
| ZFYVE27 | 1.6510024 | 12.17264853 | 7.300126112 | 7.04E-06 | 0.000199255 | 3.9913836 |
| AKTIP | -2.242135034 | 5.242719794 | -7.296380612 | 7.07E-06 | 0.000199582 | 3.985924395 |
| TRIM24 | 3.220547794 | 7.342223374 | 7.295484633 | 7.08E-06 | 0.000199582 | 3.984618193 |
| SECTM1 | -5.984065104 | 10.02748312 | -7.295042089 | 7.09E-06 | 0.000199582 | 3.983972991 |
| TMEM211 | 1.73515585 | 7.188634294 | 7.292912925 | 7.11E-06 | 0.000199816 | 3.980868433 |
| TRAPPC5 | -1.727727546 | 12.41763545 | -7.290598531 | 7.13E-06 | 0.000200104 | 3.977493094 |
| GP6 | -3.618597938 | 6.193027891 | -7.27981667 | 7.24E-06 | 0.000202825 | 3.961759209 |
| HMOX2 | -1.491114919 | 11.16439807 | -7.275051044 | 7.29E-06 | 0.000203738 | 3.954799778 |
| CDC14C | -1.516155578 | 5.094029364 | -7.274075379 | 7.30E-06 | 0.000203738 | 3.9533746 |
| SPDYE16 | 1.891512851 | 6.707892718 | 7.270822496 | 7.34E-06 | 0.000204194 | 3.948622103 |
| GTF2E2 | -1.743676506 | 7.824203285 | -7.269925876 | 7.35E-06 | 0.000204194 | 3.947311881 |
| GPR171 | -3.2886624 | 9.31945287 | -7.2622866 | 7.43E-06 | 0.000205798 | 3.936144286 |
| WEE1 | 1.65484667 | 7.740669926 | 7.261872268 | 7.43E-06 | 0.000205798 | 3.935538363 |
| INTS14 | 1.087088594 | 8.49199899 | 7.250567394 | 7.55E-06 | 0.000208765 | 3.91899708 |
| TRIM21 | -1.323692437 | 7.829753372 | -7.247314776 | 7.59E-06 | 0.000209355 | 3.914234656 |
| SLC9B1 | 1.978689171 | 4.242630482 | 7.244580357 | 7.62E-06 | 0.000209793 | 3.910229865 |
| ME1 | -1.898141012 | 2.843043443 | -7.239325591 | 7.67E-06 | 0.000210991 | 3.902530974 |
| COL24A1 | 3.902693699 | 6.721568149 | 7.236452376 | 7.70E-06 | 0.000211476 | 3.898319777 |
| EVI2A | -2.445670697 | 11.13137905 | -7.229408324 | 7.78E-06 | 0.000213231 | 3.887990768 |
| SMARCAL1 | 1.248916218 | 10.6169645 | 7.218696094 | 7.90E-06 | 0.000216136 | 3.872270087 |
| TOMM7 | -1.381577527 | 16.06052717 | -7.21717548 | 7.92E-06 | 0.000216217 | 3.870037258 |
| CCNQ | -1.856959987 | 7.928598584 | -7.209610508 | 8.01E-06 | 0.00021793 | 3.858924407 |
| AZGP1 | 2.455865798 | 7.199377899 | 7.209049894 | 8.01E-06 | 0.00021793 | 3.858100564 |
| NREP | 2.456835434 | 8.095858192 | 7.207589022 | 8.03E-06 | 0.00021793 | 3.855953558 |
| PELI3 | -2.944039782 | 7.55990048 | -7.206658535 | 8.04E-06 | 0.00021793 | 3.854585894 |
| PI4K2A | -2.101181835 | 7.363660842 | -7.204784635 | 8.06E-06 | 0.000218126 | 3.85183121 |
| NR2C2 | 1.788366584 | 8.712752693 | 7.19916709 | 8.13E-06 | 0.000219499 | 3.843570419 |
| SERPINA1 | -1.466752315 | 9.627977523 | -7.195736446 | 8.17E-06 | 0.000219613 | 3.838523437 |
| TSPAN3 | 1.127179442 | 6.786291361 | 7.194860251 | 8.18E-06 | 0.000219613 | 3.83723417 |
| TNFAIP8L1 | 3.079321901 | 12.50463952 | 7.194221376 | 8.19E-06 | 0.000219613 | 3.836294038 |
| BDH1 | 1.435188619 | 8.505310138 | 7.192779839 | 8.20E-06 | 0.000219613 | 3.834172553 |
| RPGR | 2.519404073 | 6.348337771 | 7.192621712 | 8.20E-06 | 0.000219613 | 3.833939823 |
| GZMB | -4.187070685 | 13.64025494 | -7.189974192 | 8.24E-06 | 0.000219795 | 3.830042722 |
| CHKA | 2.47802642 | 9.24548278 | 7.18852837 | 8.25E-06 | 0.000219795 | 3.827914098 |
| YIPF5 | -1.630126367 | 6.984353832 | -7.18771078 | 8.26E-06 | 0.000219795 | 3.826710268 |
| ALG9 | 1.182560868 | 6.276850343 | 7.187142982 | 8.27E-06 | 0.000219795 | 3.825874182 |
| CAPZA2 | -2.144682871 | 7.263764672 | -7.181149294 | 8.34E-06 | 0.000221307 | 3.817045768 |
| SFPQ | 1.713007831 | 11.45032929 | 7.177179963 | 8.39E-06 | 0.000222185 | 3.811196452 |
| FLOT2 | -1.444196592 | 11.72680864 | -7.170920999 | 8.46E-06 | 0.000223802 | 3.801968724 |
| HNRNPU | 1.578026786 | 8.26881534 | 7.163926205 | 8.55E-06 | 0.000225672 | 3.791649848 |
| FUS | 1.585205941 | 7.104198916 | 7.162284204 | 8.57E-06 | 0.000225813 | 3.789226568 |
| RGS10 | -2.420778949 | 13.93116219 | -7.156237784 | 8.64E-06 | 0.000227362 | 3.780300047 |
| PRKACA | -1.47772165 | 8.580391188 | -7.155124748 | 8.66E-06 | 0.000227362 | 3.778656296 |
| HTR3A | -1.415191046 | 4.647675813 | -7.152076383 | 8.70E-06 | 0.000227968 | 3.774153552 |
| TRIO | 2.376130031 | 8.989335126 | 7.150469087 | 8.72E-06 | 0.000228102 | 3.771778904 |
| CHML | 2.216676 | 6.943694552 | 7.142550099 | 8.82E-06 | 0.000230033 | 3.760074118 |
| TSPO | -1.661319125 | 14.6995363 | -7.141199684 | 8.83E-06 | 0.000230033 | 3.758077266 |
| CYP4F22 | -2.385751033 | 3.833656953 | -7.140578697 | 8.84E-06 | 0.000230033 | 3.757158931 |
| FAM122B | 1.399060852 | 8.873499692 | 7.139845284 | 8.85E-06 | 0.000230033 | 3.756074268 |
| HPS4 | 2.233702116 | 6.897482838 | 7.136526549 | 8.89E-06 | 0.000230393 | 3.751165201 |
| ARHGEF17 | 1.731287995 | 7.917981426 | 7.136388851 | 8.90E-06 | 0.000230393 | 3.750961485 |
| LAT2 | 1.892275485 | 12.99406642 | 7.133848391 | 8.93E-06 | 0.000230844 | 3.747202579 |
| PGLYRP1 | -2.05222082 | 8.478457221 | -7.127384884 | 9.01E-06 | 0.000232612 | 3.737635104 |
| CD247 | -4.3477958 | 9.982342831 | -7.125946452 | 9.03E-06 | 0.000232699 | 3.735505121 |
| OR5D3P | 2.213551824 | 3.037155217 | 7.124442847 | 9.05E-06 | 0.000232809 | 3.733278329 |
| SGPL1 | -1.862541403 | 8.125931272 | -7.118749731 | 9.13E-06 | 0.000233866 | 3.724844212 |
| ZNF85 | 2.098116161 | 5.852566176 | 7.118273767 | 9.13E-06 | 0.000233866 | 3.72413889 |
| SMPD4 | 1.404492276 | 11.24402541 | 7.117789691 | 9.14E-06 | 0.000233866 | 3.723421517 |
| CCDC200 | 2.009425539 | 6.734240652 | 7.113952062 | 9.19E-06 | 0.000234771 | 3.717733231 |
| SLFN5 | 2.573741177 | 12.81035206 | 7.110608963 | 9.23E-06 | 0.000235511 | 3.71277632 |
| TEC | 2.808221118 | 3.65473905 | 7.108100689 | 9.27E-06 | 0.000235766 | 3.709056228 |
| TSPAN18 | -3.357714077 | 6.733643564 | -7.107538508 | 9.27E-06 | 0.000235766 | 3.708222323 |
| MYO19 | 1.223660264 | 9.377901784 | 7.103316991 | 9.33E-06 | 0.000236068 | 3.701959011 |
| CHN1 | -3.163411738 | 4.56175195 | -7.102481812 | 9.34E-06 | 0.000236068 | 3.700719596 |
| TAS2R8 | 1.449530295 | 2.862534964 | 7.102033811 | 9.35E-06 | 0.000236068 | 3.70005472 |
| GOLGA6L7 | 1.281086329 | 4.522739144 | 7.102030285 | 9.35E-06 | 0.000236068 | 3.700049487 |
| HYKK | -2.735094651 | 5.42543259 | -7.098220225 | 9.40E-06 | 0.000236978 | 3.694393879 |
| TRADD | -1.575898598 | 12.50202764 | -7.096334267 | 9.43E-06 | 0.000237232 | 3.691593652 |
| KLF17 | 3.216415407 | 12.69115314 | 7.094334738 | 9.45E-06 | 0.000237246 | 3.688624267 |
| IL18RAP | -2.662407441 | 9.835507517 | -7.094002396 | 9.46E-06 | 0.000237246 | 3.688130672 |
| FBLN2 | -2.882220874 | 4.104772754 | -7.08829561 | 9.54E-06 | 0.000238695 | 3.679652591 |
| PRR7 | -1.78290106 | 10.16131977 | -7.087372957 | 9.55E-06 | 0.000238695 | 3.678281468 |
| IFNL1 | -1.556162247 | 4.974168504 | -7.086379789 | 9.56E-06 | 0.000238695 | 3.676805422 |
| LYRM9 | -1.422862816 | 9.516462272 | -7.084027104 | 9.60E-06 | 0.000239116 | 3.67330833 |
| PLPPR2 | -1.804385742 | 7.133176836 | -7.078808644 | 9.67E-06 | 0.000240328 | 3.665548772 |
| CFP | -2.556548013 | 13.22860453 | -7.078275595 | 9.68E-06 | 0.000240328 | 3.664755948 |
| METTL22 | 1.252738403 | 9.535679935 | 7.073098174 | 9.75E-06 | 0.000241669 | 3.657053356 |
| CRYL1 | -2.090863042 | 10.56362868 | -7.072184868 | 9.76E-06 | 0.000241669 | 3.655694226 |
| FOXP3 | -1.588844935 | 4.579850761 | -7.069198588 | 9.80E-06 | 0.000242323 | 3.651249414 |
| POU2F2 | -1.523615153 | 6.131299088 | -7.063925605 | 9.88E-06 | 0.000243787 | 3.643398068 |
| ZNF439 | 2.187323944 | 6.661997855 | 7.061849084 | 9.91E-06 | 0.000244126 | 3.640305134 |
| ELOVL7 | -4.714851855 | 5.268266687 | -7.060582456 | 9.93E-06 | 0.000244141 | 3.638418228 |
| LRRC25 | -5.030848668 | 9.901996638 | -7.059571489 | 9.94E-06 | 0.000244141 | 3.636912025 |
| NAT10 | 1.399333099 | 9.199832449 | 7.052870161 | 1.00E-05 | 0.000245628 | 3.626924422 |
| ZNF431 | 2.407434463 | 3.547972851 | 7.052336445 | 1.00E-05 | 0.000245628 | 3.62612871 |
| ZNF713 | 2.456709306 | 4.243556728 | 7.048017114 | 1.01E-05 | 0.000246677 | 3.619687638 |
| GABARAPL1 | -1.391271841 | 8.612648745 | -7.045369736 | 1.01E-05 | 0.00024723 | 3.615738553 |
| BEX5 | -4.30920945 | 6.64975504 | -7.0419855 | 1.02E-05 | 0.000248052 | 3.610688898 |
| UBR4 | 2.318618746 | 4.973415884 | 7.037634129 | 1.03E-05 | 0.000249227 | 3.604193867 |
| CD163 | -3.327353087 | 9.701596297 | -7.031838205 | 1.04E-05 | 0.000250937 | 3.595538601 |
| PVRIG | -3.195572454 | 10.72954054 | -7.015391895 | 1.06E-05 | 0.000256614 | 3.570953629 |
| NR3C2 | -3.902384023 | 4.705702601 | -7.008520863 | 1.07E-05 | 0.000258786 | 3.560671382 |
| ZNF445 | 1.949332211 | 6.549916796 | 6.993986691 | 1.09E-05 | 0.000263916 | 3.538900148 |
| NAP1L3 | -1.459767813 | 3.783715071 | -6.99022264 | 1.10E-05 | 0.00026495 | 3.533257111 |
| NR1D1 | -1.145295296 | 7.324599095 | -6.973357637 | 1.13E-05 | 0.000270371 | 3.507949291 |
| COQ10B | -1.072160446 | 8.270049631 | -6.973331464 | 1.13E-05 | 0.000270371 | 3.507909986 |
| CNEP1R1 | -1.773928892 | 5.982367913 | -6.973125477 | 1.13E-05 | 0.000270371 | 3.507600637 |
| GEN1 | 3.081538923 | 5.153370532 | 6.970780487 | 1.13E-05 | 0.000270872 | 3.504078541 |
| MYLK4 | 2.13816902 | 3.90201261 | 6.969438602 | 1.13E-05 | 0.000270977 | 3.502062734 |
| LAMP3 | -3.114721158 | 5.357541051 | -6.966367644 | 1.14E-05 | 0.00027177 | 3.497448547 |
| CNN1 | -4.220777194 | 4.342611797 | -6.962051015 | 1.15E-05 | 0.000273064 | 3.490960511 |
| IQCA1L | 1.745834205 | 4.859337806 | 6.954224449 | 1.16E-05 | 0.00027543 | 3.479190375 |
| ARMCX4 | 1.4757446 | 4.66924397 | 6.954023467 | 1.16E-05 | 0.00027543 | 3.478888013 |
| FAM241B | -3.153592533 | 6.116566912 | -6.952318978 | 1.16E-05 | 0.000275688 | 3.476323516 |
| UAP1 | -1.140752341 | 7.196095288 | -6.943493676 | 1.18E-05 | 0.000278844 | 3.463038958 |
| CD5 | -4.115965372 | 9.615341995 | -6.941532401 | 1.18E-05 | 0.000278891 | 3.460085229 |
| VIL1 | -3.951785935 | 5.409243546 | -6.941253444 | 1.18E-05 | 0.000278891 | 3.459665071 |
| AGO2 | 1.668462899 | 6.17684283 | 6.939294938 | 1.19E-05 | 0.000279002 | 3.456714905 |
| HEATR1 | 1.469998286 | 7.211017232 | 6.938863461 | 1.19E-05 | 0.000279002 | 3.456064886 |
| TNNT1 | -2.97612555 | 8.4818776 | -6.932372814 | 1.20E-05 | 0.000281238 | 3.446283633 |
| RCOR3 | 1.472602221 | 7.927734952 | 6.925941563 | 1.21E-05 | 0.00028347 | 3.436586149 |
| WFDC8 | 1.834947499 | 2.625326132 | 6.922608355 | 1.21E-05 | 0.000284422 | 3.431557861 |
| MIS18BP1 | 1.982746491 | 10.38723938 | 6.918482826 | 1.22E-05 | 0.000285712 | 3.425332197 |
| AC068775.1 | -5.232917389 | 7.517032794 | -6.916199919 | 1.23E-05 | 0.000286231 | 3.421886145 |
| VEPH1 | -3.613606044 | 4.983408757 | -6.914078175 | 1.23E-05 | 0.000286685 | 3.418682724 |
| HKDC1 | -2.024350261 | 5.86944026 | -6.912884 | 1.23E-05 | 0.000286748 | 3.416879477 |
| RARRES3 | -1.684955459 | 13.88282932 | -6.910539817 | 1.24E-05 | 0.000287298 | 3.413339105 |
| CDO1 | -2.027429499 | 3.879532773 | -6.904789312 | 1.25E-05 | 0.000289297 | 3.404651018 |
| CTLA4 | -4.865200429 | 6.500855652 | -6.901394422 | 1.25E-05 | 0.000290023 | 3.399519737 |
| PLEKHF1 | -2.226016381 | 7.112125307 | -6.901012844 | 1.25E-05 | 0.000290023 | 3.398942892 |
| SUGP2 | 2.787983166 | 8.05858212 | 6.893008138 | 1.27E-05 | 0.000293017 | 3.386837269 |
| GNG10 | -2.009844682 | 10.49241696 | -6.890469837 | 1.27E-05 | 0.000293669 | 3.38299671 |
| CD52 | -4.363981389 | 15.12188579 | -6.883000774 | 1.29E-05 | 0.000296445 | 3.371690523 |
| KIR3DL1 | -1.777703551 | 7.013174329 | -6.882031969 | 1.29E-05 | 0.000296445 | 3.370223443 |
| SH3D21 | 1.481424963 | 5.97162187 | 6.878002933 | 1.30E-05 | 0.000297728 | 3.364120798 |
| TRAF3IP2 | 2.333840962 | 3.42969002 | 6.876975768 | 1.30E-05 | 0.000297728 | 3.362564626 |
| OSBPL3 | 2.094410626 | 8.575404964 | 6.876028952 | 1.30E-05 | 0.000297728 | 3.361130054 |
| LRFN4 | 1.318791386 | 9.806311293 | 6.872952427 | 1.31E-05 | 0.000298633 | 3.35646779 |
| ITPR2 | 2.777561723 | 8.954552343 | 6.85658862 | 1.34E-05 | 0.000305484 | 3.331647503 |
| CLEC1A | -2.590035613 | 4.767723051 | -6.853298399 | 1.35E-05 | 0.000306215 | 3.326652479 |
| CCDC189 | 2.220518567 | 6.611523314 | 6.852938402 | 1.35E-05 | 0.000306215 | 3.326105862 |
| CUX1 | 1.594354229 | 7.508792327 | 6.847755744 | 1.36E-05 | 0.000308111 | 3.318234543 |
| XCL1 | -2.500678932 | 5.41488292 | -6.843933643 | 1.36E-05 | 0.000308914 | 3.312427223 |
| SPTLC1 | -1.520948582 | 6.213537658 | -6.84344474 | 1.37E-05 | 0.000308914 | 3.311684236 |
| GGCX | 1.876546053 | 10.46785668 | 6.842155646 | 1.37E-05 | 0.000308914 | 3.309725035 |
| OXNAD1 | -2.421623675 | 8.271490042 | -6.841959415 | 1.37E-05 | 0.000308914 | 3.309426776 |
| ANXA6 | -3.035391119 | 10.07246006 | -6.839754753 | 1.37E-05 | 0.000309463 | 3.306075475 |
| ACPP | -3.016970538 | 6.299728446 | -6.838646439 | 1.37E-05 | 0.000309511 | 3.304390475 |
| CCDC163 | -3.026278729 | 4.107721658 | -6.831167346 | 1.39E-05 | 0.000312497 | 3.293015343 |
| DCTN4 | 1.768330987 | 10.2294825 | 6.823953037 | 1.41E-05 | 0.00031539 | 3.282035565 |
| SBK1 | -3.596780731 | 7.22477349 | -6.821560692 | 1.41E-05 | 0.000316015 | 3.278392951 |
| NFAM1 | -4.727712575 | 8.42245779 | -6.820626666 | 1.41E-05 | 0.000316015 | 3.276970575 |
| NPIPB15 | 1.904928768 | 10.98043292 | 6.817104094 | 1.42E-05 | 0.00031697 | 3.271605158 |
| MPV17L | 2.294425245 | 6.735251049 | 6.816605381 | 1.42E-05 | 0.00031697 | 3.270845403 |
| DSC1 | -3.742758322 | 4.044726995 | -6.813675961 | 1.43E-05 | 0.00031772 | 3.26638193 |
| KCNT1 | 2.390859186 | 9.23597787 | 6.813032855 | 1.43E-05 | 0.00031772 | 3.265401889 |
| ZNF883 | -1.752036292 | 2.680743764 | -6.806706538 | 1.44E-05 | 0.000320249 | 3.255758024 |
| GLTP | -2.175700963 | 7.882276622 | -6.799025558 | 1.46E-05 | 0.000323452 | 3.244041622 |
| OR10H2 | -2.613801915 | 12.14381096 | -6.79657836 | 1.46E-05 | 0.00032347 | 3.240306993 |
| APOL3 | -2.227564874 | 7.27868375 | -6.795619898 | 1.47E-05 | 0.00032347 | 3.238844074 |
| TNPO2 | 1.170529897 | 11.20407989 | 6.795539317 | 1.47E-05 | 0.00032347 | 3.238721075 |
| MANSC1 | -3.149822461 | 3.725506867 | -6.795060828 | 1.47E-05 | 0.00032347 | 3.237990695 |
| SHLD3 | -1.96001279 | 8.615329783 | -6.79403945 | 1.47E-05 | 0.000323491 | 3.236431524 |
| CD14 | -5.354907061 | 12.0508969 | -6.788602888 | 1.48E-05 | 0.000325645 | 3.228129972 |
| UQCRB | -2.093755154 | 5.192669109 | -6.787409848 | 1.48E-05 | 0.000325751 | 3.226307667 |
| SH2D3A | -2.638402248 | 9.037310385 | -6.784129495 | 1.49E-05 | 0.000326767 | 3.221296078 |
| NPC2 | -1.441772391 | 14.37109997 | -6.783373444 | 1.49E-05 | 0.000326767 | 3.220140801 |
| DPYD | -1.376363604 | 6.952510503 | -6.778629429 | 1.50E-05 | 0.000327892 | 3.21288994 |
| NKG7 | -2.984998191 | 13.90549067 | -6.777747069 | 1.51E-05 | 0.000327892 | 3.211540974 |
| GRAMD1B | -2.293702363 | 4.883152805 | -6.777708394 | 1.51E-05 | 0.000327892 | 3.211481845 |
| CEP170 | 1.934546205 | 6.803822171 | 6.777195305 | 1.51E-05 | 0.000327892 | 3.210697375 |
| CENPP | 1.391772505 | 7.494639694 | 6.775585505 | 1.51E-05 | 0.000328208 | 3.208235885 |
| NBAS | 1.523876564 | 8.499212717 | 6.772379521 | 1.52E-05 | 0.000328891 | 3.20333265 |
| ZBED4 | 1.479191689 | 6.328411575 | 6.771286624 | 1.52E-05 | 0.000328891 | 3.201660845 |
| GART | 1.286467335 | 7.607800975 | 6.769680152 | 1.52E-05 | 0.000328891 | 3.199203121 |
| CCDC169 | 2.536759534 | 3.093234552 | 6.768602832 | 1.53E-05 | 0.000328891 | 3.197554739 |
| ATAT1 | 1.497321091 | 7.2671663 | 6.768498396 | 1.53E-05 | 0.000328891 | 3.197394936 |
| CCDC14 | 2.261320611 | 8.405270074 | 6.76843962 | 1.53E-05 | 0.000328891 | 3.197304999 |
| EZH2 | 1.87232431 | 9.356426717 | 6.755465269 | 1.56E-05 | 0.000334126 | 3.17744025 |
| FAM49B | -1.274036428 | 7.954944773 | -6.755164239 | 1.56E-05 | 0.000334126 | 3.176979072 |
| LLPH | -1.326035757 | 9.988396635 | -6.754998675 | 1.56E-05 | 0.000334126 | 3.176725421 |
| COA1 | -1.142210739 | 9.883401266 | -6.752125847 | 1.56E-05 | 0.000335088 | 3.172323534 |
| CDK13 | 1.262908736 | 7.721514681 | 6.745332031 | 1.58E-05 | 0.000336703 | 3.161909136 |
| RTL10 | 1.352458602 | 9.924314704 | 6.744955187 | 1.58E-05 | 0.000336703 | 3.161331275 |
| AL139011.2 | 2.669588236 | 8.123596662 | 6.744283546 | 1.58E-05 | 0.000336703 | 3.160301315 |
| NUP205 | 1.480505784 | 6.514500705 | 6.744208801 | 1.58E-05 | 0.000336703 | 3.16018669 |
| SLC12A2 | 1.670422478 | 5.882465806 | 6.742978755 | 1.59E-05 | 0.000336703 | 3.158300242 |
| RAD1 | 1.415581382 | 5.490720447 | 6.742419589 | 1.59E-05 | 0.000336703 | 3.157442614 |
| IL12RB2 | -1.417912796 | 5.523966388 | -6.742310682 | 1.59E-05 | 0.000336703 | 3.157275571 |
| PBX1 | -3.783107746 | 4.578299126 | -6.724325442 | 1.63E-05 | 0.000345399 | 3.129666881 |
| AKAP17A | 2.538836702 | 6.135367327 | 6.722710057 | 1.63E-05 | 0.000345751 | 3.127184932 |
| ETS1 | -2.378570658 | 8.857950583 | -6.718745426 | 1.64E-05 | 0.000347325 | 3.121091953 |
| GZMA | -4.632519488 | 12.45284256 | -6.71407548 | 1.66E-05 | 0.000349276 | 3.113912204 |
| KCTD6 | -2.303625472 | 7.381092754 | -6.707725697 | 1.67E-05 | 0.000352124 | 3.104144919 |
| THOP1 | 1.231559792 | 12.3514349 | 6.703511349 | 1.68E-05 | 0.000353595 | 3.097659262 |
| NEMP1 | 1.672117807 | 7.299676959 | 6.703094004 | 1.68E-05 | 0.000353595 | 3.097016857 |
| BCL9L | -1.905829878 | 7.293652681 | -6.701373279 | 1.69E-05 | 0.000354017 | 3.094367938 |
| MRPS7 | -1.029700132 | 9.531154606 | -6.699674546 | 1.69E-05 | 0.000354429 | 3.091752469 |
| GABPB1 | 1.472133116 | 8.158709467 | 6.697884418 | 1.70E-05 | 0.000354892 | 3.088995845 |
| ASB3 | 1.907282427 | 11.11832011 | 6.696186905 | 1.70E-05 | 0.000355306 | 3.086381426 |
| MS4A6A | -4.072716865 | 10.62858697 | -6.694031856 | 1.71E-05 | 0.000355966 | 3.083061753 |
| MYF6 | 1.561926807 | 3.293862765 | 6.689915867 | 1.72E-05 | 0.000357679 | 3.076719608 |
| ZNF326 | 1.900021239 | 8.639307968 | 6.688853 | 1.72E-05 | 0.000357758 | 3.075081499 |
| OCLM | 2.513684531 | 4.690206366 | 6.681982244 | 1.74E-05 | 0.000360974 | 3.064488352 |
| SGCE | -4.14978486 | 4.513523555 | -6.680786652 | 1.74E-05 | 0.000361127 | 3.062644344 |
| HDAC6 | 1.495944647 | 9.867078799 | 6.676783004 | 1.75E-05 | 0.00036248 | 3.056467908 |
| HELZ2 | -1.69589928 | 10.46729395 | -6.675685633 | 1.75E-05 | 0.00036248 | 3.054774601 |
| CATSPER2 | 1.94695382 | 5.720722354 | 6.675570104 | 1.75E-05 | 0.00036248 | 3.054596323 |
| LTB | -4.018281468 | 10.97969413 | -6.66831122 | 1.77E-05 | 0.000365512 | 3.043391069 |
| SCGN | 1.309107311 | 4.553024501 | 6.668217861 | 1.77E-05 | 0.000365512 | 3.043246908 |
| PLPP4 | 2.568895413 | 3.405915139 | 6.658890952 | 1.80E-05 | 0.000370175 | 3.028838365 |
| ZNF286A | -1.891795676 | 3.025258361 | -6.657422201 | 1.80E-05 | 0.000370491 | 3.026568275 |
| MBTD1 | 1.505917017 | 7.914139945 | 6.65215474 | 1.82E-05 | 0.000372936 | 3.018424441 |
| POLR3E | 1.684451372 | 4.285913032 | 6.650112891 | 1.82E-05 | 0.000373085 | 3.015266564 |
| USP28 | -2.141266483 | 4.300935928 | -6.650093454 | 1.82E-05 | 0.000373085 | 3.015236501 |
| E2F3 | 1.672060234 | 8.815158364 | 6.64697729 | 1.83E-05 | 0.000374335 | 3.010415978 |
| FBXL20 | 1.938637509 | 8.366759194 | 6.644411415 | 1.84E-05 | 0.00037511 | 3.006445697 |
| GBP1 | -4.516515043 | 6.662251437 | -6.643817229 | 1.84E-05 | 0.00037511 | 3.005526158 |
| PLCD1 | -1.230371607 | 8.280823939 | -6.642329849 | 1.84E-05 | 0.000375355 | 3.003224129 |
| CFAP161 | -2.275827629 | 6.366812692 | -6.641600996 | 1.85E-05 | 0.000375355 | 3.002095964 |
| MDC1 | 1.638388835 | 9.015166861 | 6.638062447 | 1.86E-05 | 0.000376362 | 2.996617717 |
| OR52I2 | 3.24909176 | 4.971480107 | 6.638047116 | 1.86E-05 | 0.000376362 | 2.996593977 |
| GNLY | -4.187748378 | 12.45062038 | -6.633666015 | 1.87E-05 | 0.000377996 | 2.98980888 |
| ZBTB12 | 2.125139305 | 3.522965331 | 6.632456174 | 1.87E-05 | 0.000377996 | 2.9879347 |
| SNN | -2.661887632 | 9.930772351 | -6.631715614 | 1.87E-05 | 0.000377996 | 2.986787388 |
| TMCO3 | 1.160656419 | 7.788169783 | 6.630862423 | 1.88E-05 | 0.000377996 | 2.985465487 |
| DPP4 | -4.925414994 | 6.509501811 | -6.630753478 | 1.88E-05 | 0.000377996 | 2.985296686 |
| MT-ATP6 | 1.763593247 | 16.50464266 | 6.627706229 | 1.88E-05 | 0.000379234 | 2.980574516 |
| OR4D1 | -1.333847898 | 4.076567335 | -6.624890397 | 1.89E-05 | 0.000379734 | 2.976209803 |
| ZNF595 | -2.343720004 | 5.863932069 | -6.624317768 | 1.89E-05 | 0.000379734 | 2.975322057 |
| GSC | -2.881831752 | 4.887271151 | -6.624200542 | 1.89E-05 | 0.000379734 | 2.975140315 |
| USP34 | 2.080889558 | 7.617588034 | 6.622690597 | 1.90E-05 | 0.000380098 | 2.972799203 |
| ZNF532 | -1.752880244 | 4.778294378 | -6.620543513 | 1.91E-05 | 0.00038083 | 2.96946968 |
| PTK7 | 1.676663834 | 4.500218326 | 6.615216322 | 1.92E-05 | 0.000382611 | 2.96120591 |
| IL4 | -3.77570329 | 4.180148436 | -6.614930828 | 1.92E-05 | 0.000382611 | 2.960762926 |
| CDK19 | 1.512987397 | 11.03471507 | 6.614841862 | 1.92E-05 | 0.000382611 | 2.960624882 |
| ABHD11 | -1.294300249 | 8.478705415 | -6.611679211 | 1.93E-05 | 0.00038394 | 2.955716788 |
| NUP160 | 1.38530715 | 8.808753415 | 6.609909647 | 1.94E-05 | 0.000384249 | 2.952970001 |
| LRRC24 | 1.41894971 | 9.081692329 | 6.608884825 | 1.94E-05 | 0.000384249 | 2.951379033 |
| UNKL | 2.415267358 | 7.086823311 | 6.6085472 | 1.94E-05 | 0.000384249 | 2.950854859 |
| NAA35 | 1.801816396 | 7.50231401 | 6.605473581 | 1.95E-05 | 0.000385536 | 2.946082235 |
| CLEC1B | -5.396114979 | 7.452323973 | -6.604201191 | 1.95E-05 | 0.000385775 | 2.944106118 |
| KCTD17 | -1.974991163 | 6.498128026 | -6.594910275 | 1.98E-05 | 0.000390728 | 2.929669726 |
| IRS1 | -1.437373722 | 4.016260619 | -6.580898618 | 2.02E-05 | 0.000398592 | 2.907875214 |
| IFI6 | -1.879175319 | 11.44431088 | -6.579231982 | 2.03E-05 | 0.000398746 | 2.905280999 |
| INPP5E | 1.379140466 | 10.04545958 | 6.578731977 | 2.03E-05 | 0.000398746 | 2.904502635 |
| FAM206A | -1.866151176 | 6.77936972 | -6.578076905 | 2.03E-05 | 0.000398746 | 2.903482824 |
| RFWD3 | 1.782891752 | 8.349689514 | 6.57306836 | 2.05E-05 | 0.000400799 | 2.895683568 |
| SIAH1 | 1.546500848 | 7.856825769 | 6.570528203 | 2.06E-05 | 0.000400799 | 2.891726711 |
| TMEM120A | -2.253536075 | 9.451997673 | -6.570495097 | 2.06E-05 | 0.000400799 | 2.891675135 |
| SELP | -1.829146896 | 6.543630585 | -6.569181091 | 2.06E-05 | 0.000400799 | 2.889627918 |
| C16orf87 | -1.103522367 | 9.421561717 | -6.568686496 | 2.06E-05 | 0.000400799 | 2.888857277 |
| PKD1L3 | 1.70122282 | 3.608383635 | 6.568662309 | 2.06E-05 | 0.000400799 | 2.88881959 |
| LRFN3 | -3.50504124 | 6.855600532 | -6.568358054 | 2.06E-05 | 0.000400799 | 2.888345506 |
| SHANK3 | 3.34579903 | 5.458288098 | 6.567896018 | 2.06E-05 | 0.000400799 | 2.887625543 |
| DTX3 | -1.118796466 | 4.59821321 | -6.565923254 | 2.07E-05 | 0.000401486 | 2.884551169 |
| C9orf16 | -1.595827254 | 9.922639249 | -6.56446469 | 2.07E-05 | 0.000401861 | 2.882277778 |
| FKRP | 1.49400978 | 7.746205511 | 6.557620438 | 2.10E-05 | 0.000404727 | 2.871605983 |
| NOL8 | 2.057082786 | 11.2449474 | 6.556579863 | 2.10E-05 | 0.000404727 | 2.869982904 |
| ALDH5A1 | 1.729315111 | 6.079899695 | 6.555496605 | 2.10E-05 | 0.000404727 | 2.868293087 |
| SATB1 | 1.674939243 | 11.55848617 | 6.554786994 | 2.10E-05 | 0.000404727 | 2.867186047 |
| TGFBR3 | -4.079912793 | 6.817322876 | -6.554584204 | 2.11E-05 | 0.000404727 | 2.86686967 |
| SOCS3 | -2.386272814 | 8.870388373 | -6.554165305 | 2.11E-05 | 0.000404727 | 2.866216114 |
| MRFAP1L1 | -1.579295279 | 9.498031819 | -6.553918875 | 2.11E-05 | 0.000404727 | 2.865831629 |
| TTC1 | -1.309513337 | 9.412141873 | -6.551548211 | 2.12E-05 | 0.000405674 | 2.862132434 |
| CPVL | -5.308626138 | 8.827924546 | -6.550405238 | 2.12E-05 | 0.000405865 | 2.860348651 |
| ZNF629 | 1.090339345 | 8.020880224 | 6.547565376 | 2.13E-05 | 0.000406953 | 2.855915818 |
| TREM1 | -3.577361267 | 6.740412253 | -6.546331645 | 2.13E-05 | 0.000406953 | 2.853989694 |
| CFAP221 | 1.12102996 | 9.309247957 | 6.5461557 | 2.13E-05 | 0.000406953 | 2.853714987 |
| SH3BP5 | -3.311774497 | 7.247056945 | -6.544404469 | 2.14E-05 | 0.000407525 | 2.850980521 |
| MDFI | 4.202867687 | 6.722629108 | 6.540251705 | 2.15E-05 | 0.000409592 | 2.844494443 |
| AMIGO2 | -3.399844169 | 6.5434493 | -6.538497651 | 2.16E-05 | 0.000410171 | 2.841754108 |
| BCOR | 1.365187441 | 5.843885495 | 6.535932486 | 2.17E-05 | 0.00041126 | 2.837745807 |
| PPT2 | 2.105625243 | 9.208882737 | 6.53343295 | 2.17E-05 | 0.000411465 | 2.833839164 |
| PKD2 | 2.020376028 | 7.614659203 | 6.533139429 | 2.18E-05 | 0.000411465 | 2.833380348 |
| GCNT4 | -2.26891654 | 5.065404397 | -6.53228646 | 2.18E-05 | 0.000411486 | 2.832046967 |
| BMPR2 | -2.664320584 | 3.392500692 | -6.529120303 | 2.19E-05 | 0.000412754 | 2.827096655 |
| MYO1E | -2.022814293 | 5.94430238 | -6.528630927 | 2.19E-05 | 0.000412754 | 2.826331386 |
| AC018755.2 | -2.233108007 | 7.110365116 | -6.527634649 | 2.19E-05 | 0.000412867 | 2.824773337 |
| ASH1L | 1.388554977 | 7.112910063 | 6.526727976 | 2.20E-05 | 0.000412925 | 2.823355295 |
| B4GALT6 | 3.060826004 | 3.462801092 | 6.524646821 | 2.20E-05 | 0.000413723 | 2.820099922 |
| LY6E | -2.718234239 | 10.38118727 | -6.519622017 | 2.22E-05 | 0.000416387 | 2.812237534 |
| GABRR2 | 3.014597526 | 3.293173981 | 6.513628385 | 2.24E-05 | 0.00041969 | 2.802854549 |
| SDHB | -1.567478922 | 10.89691266 | -6.512278544 | 2.25E-05 | 0.000420035 | 2.800740684 |
| UCN | 2.016776449 | 9.423809361 | 6.511438363 | 2.25E-05 | 0.000420055 | 2.799424823 |
| BMP8B | 1.304530175 | 6.705575269 | 6.505091441 | 2.27E-05 | 0.000423621 | 2.789481283 |
| LTF | -5.006390842 | 8.718454452 | -6.504157097 | 2.27E-05 | 0.000423703 | 2.788016993 |
| NLRP12 | -4.894945682 | 7.213438647 | -6.502911787 | 2.28E-05 | 0.000423968 | 2.786065171 |
| NADK2 | 2.942136715 | 7.364898505 | 6.50213816 | 2.28E-05 | 0.000423968 | 2.784852527 |
| DEFB105A | 1.600676817 | 3.067363165 | 6.501019911 | 2.28E-05 | 0.000424172 | 2.783099544 |
| NEURL3 | -3.366136791 | 4.142895629 | -6.500123247 | 2.29E-05 | 0.000424233 | 2.781693792 |
| POU2F1 | 1.597696987 | 5.568899316 | 6.498061996 | 2.29E-05 | 0.000425051 | 2.778461823 |
| PI16 | -2.906582766 | 7.707870887 | -6.496907914 | 2.30E-05 | 0.000425077 | 2.776652002 |
| TAS2R16 | 2.448033006 | 3.29150394 | 6.495179092 | 2.30E-05 | 0.000425077 | 2.773940526 |
| ZFHX2 | 1.478648951 | 3.70426986 | 6.494894817 | 2.31E-05 | 0.000425077 | 2.773494629 |
| ILF2 | 2.947657889 | 4.507050955 | 6.494827199 | 2.31E-05 | 0.000425077 | 2.773388567 |
| LARP1B | 1.407364141 | 6.791237524 | 6.493940395 | 2.31E-05 | 0.000425136 | 2.7719975 |
| SREBF1 | 1.334934189 | 11.83846249 | 6.492170569 | 2.32E-05 | 0.00042577 | 2.769220968 |
| TREX1 | -2.21134829 | 10.72505357 | -6.487518499 | 2.33E-05 | 0.000428288 | 2.761920614 |
| LUZP1 | 2.087449418 | 6.217577908 | 6.479414222 | 2.36E-05 | 0.000433104 | 2.749195521 |
| SNAPC5 | -1.676939781 | 6.771761188 | -6.47039389 | 2.39E-05 | 0.000438593 | 2.735021162 |
| MTNR1A | -2.163388213 | 3.073296378 | -6.468084447 | 2.40E-05 | 0.000439615 | 2.731390306 |
| TMEM120B | 1.52671968 | 5.594649248 | 6.465529721 | 2.41E-05 | 0.000440806 | 2.727372942 |
| KMT2A | 1.475020367 | 6.991187336 | 6.464585269 | 2.42E-05 | 0.000440912 | 2.725887537 |
| ZNF320 | 1.566581992 | 6.179421341 | 6.46197223 | 2.43E-05 | 0.000441595 | 2.721777173 |
| 6-Mar | 1.203167081 | 6.577888665 | 6.461920587 | 2.43E-05 | 0.000441595 | 2.721695928 |
| NCR1 | -2.028643564 | 6.919917038 | -6.461219735 | 2.43E-05 | 0.000441595 | 2.720593305 |
| ZMAT1 | 1.55373553 | 7.695143797 | 6.45985398 | 2.43E-05 | 0.000441989 | 2.718444415 |
| C1orf167 | 1.637266967 | 2.641927286 | 6.457753199 | 2.44E-05 | 0.000442451 | 2.715138515 |
| DNAJB1 | -1.945359283 | 9.831892938 | -6.457609769 | 2.44E-05 | 0.000442451 | 2.714912783 |
| H2AFV | 1.115974189 | 10.14192397 | 6.452165652 | 2.46E-05 | 0.000445626 | 2.706342624 |
| CDT1 | 4.042673791 | 9.146424091 | 6.439994689 | 2.51E-05 | 0.000453311 | 2.687167876 |
| IFIT3 | -3.820726479 | 8.359899863 | -6.439465492 | 2.51E-05 | 0.000453311 | 2.686333678 |
| IFITM1 | -3.945380084 | 13.63055476 | -6.435737972 | 2.52E-05 | 0.00045537 | 2.680456689 |
| GNPDA1 | -2.559594015 | 6.073681036 | -6.431449739 | 2.54E-05 | 0.000457834 | 2.673693224 |
| TNRC6C | -2.079666886 | 9.219312491 | -6.429294984 | 2.55E-05 | 0.000458807 | 2.67029373 |
| MTAP | 2.06664026 | 5.710972632 | 6.426743836 | 2.56E-05 | 0.000460063 | 2.66626801 |
| ATAD3A | 1.299080298 | 12.3522505 | 6.408038452 | 2.63E-05 | 0.00047293 | 2.636722742 |
| SELPLG | -2.573401881 | 9.362627789 | -6.406555326 | 2.64E-05 | 0.00047315 | 2.634378019 |
| CXCL5 | -1.825345063 | 8.181079592 | -6.40484142 | 2.65E-05 | 0.00047315 | 2.631668059 |
| MLEC | 1.380859033 | 6.807463383 | 6.404822044 | 2.65E-05 | 0.00047315 | 2.63163742 |
| TOM1 | -1.017655188 | 10.36931179 | -6.404665212 | 2.65E-05 | 0.00047315 | 2.631389422 |
| AFAP1 | -2.504638086 | 3.264820499 | -6.399462001 | 2.67E-05 | 0.000476396 | 2.623159638 |
| ZNF492 | 2.020109294 | 4.536635774 | 6.397533212 | 2.68E-05 | 0.000476902 | 2.620107949 |
| C15orf38-AP3S2 | -1.317259531 | 9.504167378 | -6.396624786 | 2.68E-05 | 0.000476902 | 2.618670473 |
| CHMP2B | -1.154273591 | 7.863408022 | -6.3964839 | 2.68E-05 | 0.000476902 | 2.618447528 |
| KLRB1 | -5.133492556 | 11.57701015 | -6.394147712 | 2.69E-05 | 0.000478061 | 2.61475021 |
| COL14A1 | 1.685007095 | 5.344373906 | 6.387493041 | 2.72E-05 | 0.000482205 | 2.6042141 |
| ERI2 | 1.798153101 | 4.84107985 | 6.387030505 | 2.72E-05 | 0.000482205 | 2.60348155 |
| DIAPH3 | 2.93857478 | 5.743322748 | 6.38583245 | 2.73E-05 | 0.000482532 | 2.601583967 |
| TOP1 | 1.821286637 | 9.821738386 | 6.383581544 | 2.74E-05 | 0.000483181 | 2.598018239 |
| SYNE1 | -2.10528133 | 6.682322653 | -6.383193851 | 2.74E-05 | 0.000483181 | 2.597404009 |
| TSHZ2 | -4.34455048 | 4.807555496 | -6.38269364 | 2.74E-05 | 0.000483181 | 2.596611485 |
| GIPC1 | -2.30395176 | 10.81974093 | -6.381731316 | 2.74E-05 | 0.000483336 | 2.5950867 |
| AP1S2 | -1.735612993 | 6.896457696 | -6.379583359 | 2.75E-05 | 0.000484013 | 2.591682824 |
| CPSF6 | 1.146704577 | 10.21659167 | 6.379318791 | 2.75E-05 | 0.000484013 | 2.591263516 |
| ANKRD36 | 3.159688102 | 9.546675583 | 6.37758221 | 2.76E-05 | 0.000484323 | 2.588511008 |
| PNOC | -3.654262792 | 8.524473773 | -6.376924629 | 2.76E-05 | 0.000484323 | 2.58746862 |
| VNN3 | -5.463666325 | 7.498923735 | -6.376653259 | 2.77E-05 | 0.000484323 | 2.58703843 |
| NEGR1 | 1.287412412 | 6.21599332 | 6.374224381 | 2.78E-05 | 0.000485581 | 2.583187584 |
| BMPR1A | -3.034227759 | 4.743069566 | -6.368317702 | 2.80E-05 | 0.000489466 | 2.573819399 |
| PADI4 | -3.032896871 | 4.852895157 | -6.366591012 | 2.81E-05 | 0.000490208 | 2.571079879 |
| VAMP5 | -2.526461953 | 10.75707356 | -6.363886558 | 2.82E-05 | 0.000491694 | 2.566788217 |
| CLIP2 | 2.146426662 | 8.412176488 | 6.362872403 | 2.82E-05 | 0.0004919 | 2.565178601 |
| VGLL4 | 1.004072475 | 8.50109183 | 6.360358773 | 2.84E-05 | 0.000493248 | 2.561188465 |
| GCLM | 2.694820913 | 12.27352521 | 6.358789243 | 2.84E-05 | 0.000493427 | 2.55869654 |
| SLC35A1 | -1.639763656 | 7.862233047 | -6.35864005 | 2.84E-05 | 0.000493427 | 2.558459649 |
| PRF1 | -3.087478495 | 8.77924188 | -6.354683871 | 2.86E-05 | 0.000495887 | 2.552176836 |
| KCNQ1 | -1.982051585 | 5.84146372 | -6.352321805 | 2.87E-05 | 0.000497075 | 2.548424579 |
| LPAR1 | -2.637559099 | 3.964663906 | -6.351659672 | 2.87E-05 | 0.000497075 | 2.547372607 |
| HACD1 | 3.441297336 | 6.893910661 | 6.3441523 | 2.91E-05 | 0.000501829 | 2.535440828 |
| KCNJ15 | -2.850445385 | 4.973192817 | -6.343365769 | 2.91E-05 | 0.000501829 | 2.534190299 |
| WDR97 | 1.279422829 | 4.474656961 | 6.343299736 | 2.91E-05 | 0.000501829 | 2.534085307 |
| CIC | 1.285384526 | 9.673031885 | 6.341107569 | 2.92E-05 | 0.000502677 | 2.530599435 |
| CAB39L | -1.397737427 | 6.589556722 | -6.340741662 | 2.92E-05 | 0.000502677 | 2.530017521 |
| RWDD2A | -3.705271062 | 5.575153263 | -6.338837953 | 2.93E-05 | 0.000503216 | 2.526989687 |
| CC2D2A | -2.04318791 | 3.893880964 | -6.338586361 | 2.93E-05 | 0.000503216 | 2.526589494 |
| KLRG1 | -3.141416204 | 7.892807424 | -6.337477341 | 2.94E-05 | 0.000503511 | 2.524825331 |
| CLEC9A | -3.318970188 | 4.259365947 | -6.332812179 | 2.96E-05 | 0.00050659 | 2.51740236 |
| BRWD1 | 1.671355148 | 7.94659292 | 6.330938429 | 2.97E-05 | 0.000506937 | 2.514420076 |
| SV2A | 1.869917831 | 6.143190804 | 6.3306624 | 2.97E-05 | 0.000506937 | 2.513980703 |
| CLCN6 | 1.42615272 | 7.884734354 | 6.330187334 | 2.97E-05 | 0.000506937 | 2.513224484 |
| ZMYND8 | 1.664103066 | 11.75988134 | 6.328167562 | 2.98E-05 | 0.000507461 | 2.510009016 |
| TACSTD2 | -3.432489712 | 4.131422454 | -6.327795159 | 2.98E-05 | 0.000507461 | 2.509416089 |
| PCED1B | -1.513601076 | 9.11248687 | -6.327345566 | 2.98E-05 | 0.000507461 | 2.508700237 |
| BCR | 1.481463199 | 10.26718224 | 6.324196885 | 3.00E-05 | 0.000509373 | 2.503686031 |
| ARPC2 | -1.466755214 | 11.77490624 | -6.321793655 | 3.01E-05 | 0.000509819 | 2.499857996 |
| GPRIN3 | -3.147324058 | 4.266273552 | -6.321707637 | 3.01E-05 | 0.000509819 | 2.499720965 |
| SLC26A11 | -2.687129945 | 9.109969013 | -6.321467048 | 3.01E-05 | 0.000509819 | 2.499337689 |
| FGR | -3.640823249 | 13.54039887 | -6.316685278 | 3.03E-05 | 0.000513043 | 2.491718289 |
| PTPN18 | -2.064847673 | 10.24394438 | -6.314326552 | 3.05E-05 | 0.000513908 | 2.487958638 |
| FIG4 | -1.521502914 | 6.794799288 | -6.313539076 | 3.05E-05 | 0.000513908 | 2.486703278 |
| XPO1 | 1.277604804 | 9.25823111 | 6.312904958 | 3.05E-05 | 0.000513908 | 2.485692332 |
| MYBL1 | -3.500780106 | 6.233942666 | -6.312730081 | 3.05E-05 | 0.000513908 | 2.485413523 |
| BLOC1S1 | -2.196362564 | 11.10018182 | -6.311851585 | 3.06E-05 | 0.000514038 | 2.484012859 |
| MAVS | 1.355146352 | 9.497288462 | 6.307596644 | 3.08E-05 | 0.000516872 | 2.47722728 |
| C19orf48 | 1.645735986 | 11.59465764 | 6.297525113 | 3.13E-05 | 0.000524443 | 2.461155454 |
| GRHL1 | -1.649160103 | 5.875378998 | -6.295145698 | 3.14E-05 | 0.000525804 | 2.457356358 |
| ZBTB8A | 4.297137683 | 6.072398414 | 6.292452999 | 3.15E-05 | 0.000527427 | 2.453056091 |
| ZNF10 | 1.713544892 | 6.786021932 | 6.287518876 | 3.17E-05 | 0.000530904 | 2.445173577 |
| SFTPD | -1.514885548 | 5.916029545 | -6.284409045 | 3.19E-05 | 0.000532891 | 2.440203689 |
| ZDBF2 | -3.430796357 | 3.93292452 | -6.283033008 | 3.20E-05 | 0.000533446 | 2.438004178 |
| DCAF1 | 1.315300937 | 6.241610273 | 6.280161988 | 3.21E-05 | 0.000535246 | 2.43341416 |
| LUC7L | 2.469212859 | 9.938795609 | 6.274726635 | 3.24E-05 | 0.000539202 | 2.424721239 |
| ZNF573 | 2.490576851 | 8.603970923 | 6.269381271 | 3.27E-05 | 0.000542107 | 2.416168153 |
| THOC1 | 2.3493244 | 7.429532707 | 6.269042758 | 3.27E-05 | 0.000542107 | 2.415626363 |
| IP6K2 | 1.178777972 | 6.861431255 | 6.268502654 | 3.27E-05 | 0.000542107 | 2.414761894 |
| PRR20E | 3.140080507 | 6.457054635 | 6.268463793 | 3.27E-05 | 0.000542107 | 2.414699693 |
| SUV39H2 | 1.703788938 | 5.966590371 | 6.262662103 | 3.30E-05 | 0.000546223 | 2.405411101 |
| HNRNPD | 1.07738128 | 11.19105854 | 6.261717294 | 3.30E-05 | 0.000546223 | 2.403897996 |
| MRC2 | 3.648686548 | 10.03991087 | 6.261509237 | 3.31E-05 | 0.000546223 | 2.403564776 |
| MAP4K5 | 1.737842778 | 8.867267796 | 6.260560504 | 3.31E-05 | 0.000546413 | 2.402045232 |
| MCCC1 | 1.223542798 | 10.70223852 | 6.25988993 | 3.31E-05 | 0.000546413 | 2.400971124 |
| TSR3 | -1.78667561 | 9.845211972 | -6.258999089 | 3.32E-05 | 0.000546578 | 2.399544101 |
| HNRNPAB | 1.706225336 | 10.29701212 | 6.256477386 | 3.33E-05 | 0.000548136 | 2.395504018 |
| FCGR3A | -5.003447885 | 11.43054102 | -6.255028217 | 3.34E-05 | 0.000548781 | 2.39318186 |
| MORN3 | -2.172399494 | 5.74670506 | -6.251586834 | 3.36E-05 | 0.000550542 | 2.387666171 |
| GRN | -1.819378365 | 15.4734193 | -6.249324748 | 3.37E-05 | 0.000551793 | 2.384039689 |
| PAFAH1B2 | 1.27708264 | 10.05026233 | 6.248748042 | 3.37E-05 | 0.000551793 | 2.383115021 |
| SPON2 | -2.719461342 | 13.41094707 | -6.247688248 | 3.38E-05 | 0.000552111 | 2.381415669 |
| MICALL2 | 4.309549433 | 9.571597985 | 6.2447992 | 3.39E-05 | 0.000553714 | 2.376782341 |
| GOLGA7B | -1.757512706 | 4.333640531 | -6.244450877 | 3.40E-05 | 0.000553714 | 2.376223635 |
| AIDA | -1.555350421 | 6.898076013 | -6.242825654 | 3.40E-05 | 0.000554525 | 2.373616573 |
| AKAP11 | 1.091899015 | 8.14770655 | 6.241328948 | 3.41E-05 | 0.000555226 | 2.371215338 |
| ZDHHC21 | 1.581928399 | 5.415202025 | 6.238584955 | 3.43E-05 | 0.000556837 | 2.366812195 |
| SLC39A8 | 2.402530784 | 7.257723844 | 6.237340864 | 3.43E-05 | 0.000556837 | 2.364815516 |
| TAS2R39 | 1.911253284 | 4.749795167 | 6.236589544 | 3.44E-05 | 0.000556837 | 2.363609592 |
| BTLA | -3.978433376 | 4.819298486 | -6.236358706 | 3.44E-05 | 0.000556837 | 2.363239065 |
| SMIM10L2B | -1.476671991 | 4.869207255 | -6.235534127 | 3.44E-05 | 0.000556837 | 2.361915436 |
| UBXN2B | -2.388065786 | 7.272907561 | -6.235363248 | 3.44E-05 | 0.000556837 | 2.361641126 |
| TRABD2A | -3.328127148 | 8.523796278 | -6.231746558 | 3.46E-05 | 0.000559398 | 2.355834327 |
| GLB1L | -1.765391954 | 6.161925442 | -6.22617235 | 3.49E-05 | 0.000563695 | 2.346880988 |
| PPBP | -6.456448719 | 11.74004308 | -6.222031227 | 3.52E-05 | 0.000566755 | 2.340226626 |
| HAO1 | 1.147616422 | 4.165035603 | 6.217903426 | 3.54E-05 | 0.000569628 | 2.33359125 |
| CYP21A2 | 1.457175796 | 7.961703856 | 6.217443409 | 3.54E-05 | 0.000569628 | 2.332851629 |
| MAP9 | -1.839465146 | 3.252188243 | -6.216016954 | 3.55E-05 | 0.000570296 | 2.33055797 |
| MBOAT7 | 2.427293419 | 7.938336094 | 6.214729274 | 3.56E-05 | 0.000570842 | 2.328487205 |
| PHKA1 | 2.079274812 | 4.737918219 | 6.21057678 | 3.58E-05 | 0.000573958 | 2.321807823 |
| FGL2 | -5.025421345 | 7.490200308 | -6.208790186 | 3.59E-05 | 0.000574792 | 2.318933294 |
| RIMS1 | 1.348408911 | 5.685847622 | 6.208301421 | 3.59E-05 | 0.000574792 | 2.31814682 |
| ACIN1 | 1.835836922 | 10.34448026 | 6.206788031 | 3.60E-05 | 0.000575548 | 2.3157114 |
| ADH4 | 1.805439151 | 2.606174999 | 6.204671222 | 3.61E-05 | 0.000576852 | 2.312304386 |
| BCL2A1 | -3.106795589 | 9.052276883 | -6.202462838 | 3.63E-05 | 0.000577718 | 2.308749301 |
| GOLGA7 | -1.378233373 | 10.33764219 | -6.201515756 | 3.63E-05 | 0.000577718 | 2.307224465 |
| IER3 | -2.556026844 | 9.265753319 | -6.200557242 | 3.64E-05 | 0.000577718 | 2.305681094 |
| C19orf70 | -1.303133693 | 11.68990482 | -6.200134246 | 3.64E-05 | 0.000577718 | 2.304999956 |
| CLASP2 | 1.543347951 | 7.872078557 | 6.200013786 | 3.64E-05 | 0.000577718 | 2.304805978 |
| TBXAS1 | -1.305836306 | 8.283014447 | -6.199255783 | 3.64E-05 | 0.000577718 | 2.303585313 |
| HOPX | -3.624346936 | 9.962626845 | -6.199026652 | 3.65E-05 | 0.000577718 | 2.303216311 |
| C11orf95 | 1.132458556 | 7.266042612 | 6.196887491 | 3.66E-05 | 0.000579054 | 2.299770961 |
| PRDX5 | -1.005946688 | 13.30677836 | -6.189025194 | 3.70E-05 | 0.000585169 | 2.287102298 |
| PADI3 | 2.5052451 | 3.330345274 | 6.188861589 | 3.70E-05 | 0.000585169 | 2.286838585 |
| AC022414.1 | 1.520321744 | 3.766514642 | 6.186417878 | 3.72E-05 | 0.000586652 | 2.282899145 |
| RFX7 | 2.26288172 | 8.366576478 | 6.183014766 | 3.74E-05 | 0.000588112 | 2.277411669 |
| NDUFB10 | -1.322919508 | 12.63149942 | -6.174708856 | 3.79E-05 | 0.000595217 | 2.264011587 |
| ILF3 | 1.462691767 | 9.125710591 | 6.173655948 | 3.79E-05 | 0.000595219 | 2.26231221 |
| HTATIP2 | -2.349352853 | 7.639514238 | -6.173387695 | 3.80E-05 | 0.000595219 | 2.26187923 |
| STXBP2 | -1.380680336 | 8.376350434 | -6.171995928 | 3.80E-05 | 0.000595906 | 2.259632647 |
| SLC27A4 | -1.275110926 | 5.908026688 | -6.169971334 | 3.82E-05 | 0.000597188 | 2.256364067 |
| PHF14 | 1.953594874 | 5.996671908 | 6.165396055 | 3.84E-05 | 0.000600879 | 2.248975422 |
| TMTC4 | 1.325935083 | 7.858844917 | 6.16423752 | 3.85E-05 | 0.000601354 | 2.247104026 |
| CEBPB | -2.476863488 | 10.79397577 | -6.162434264 | 3.86E-05 | 0.000602441 | 2.244190825 |
| BPI | -4.534658321 | 6.195060339 | -6.159038968 | 3.88E-05 | 0.000605045 | 2.238704393 |
| MGA | 1.522303902 | 6.997526782 | 6.153792001 | 3.91E-05 | 0.000609436 | 2.230222643 |
| PLEKHG1 | -2.5709962 | 3.511548789 | -6.151441967 | 3.93E-05 | 0.000610928 | 2.226422533 |
| CLSPN | 3.160933025 | 5.118690871 | 6.150934997 | 3.93E-05 | 0.000610928 | 2.225602638 |
| IFIT2 | -2.380885783 | 6.988918806 | -6.14757485 | 3.95E-05 | 0.00061354 | 2.220167527 |
| OAS2 | -2.160481798 | 8.431683408 | -6.144025318 | 3.97E-05 | 0.000616349 | 2.214424341 |
| SLC25A29 | 2.508810027 | 3.16858841 | 6.143081036 | 3.98E-05 | 0.000616636 | 2.212896179 |
| SCAF8 | 1.350490322 | 8.732224629 | 6.141569004 | 3.99E-05 | 0.000617475 | 2.210448947 |
| BET1 | -1.209681011 | 6.862624924 | -6.136302291 | 4.02E-05 | 0.000621988 | 2.201922209 |
| TMEM201 | 1.373956708 | 9.107355646 | 6.134921505 | 4.03E-05 | 0.000622708 | 2.199686084 |
| PPM1N | -1.606437004 | 7.562388272 | -6.132330596 | 4.05E-05 | 0.000624621 | 2.195489483 |
| C1orf74 | -1.084363399 | 10.07428417 | -6.127007626 | 4.08E-05 | 0.000629248 | 2.186864665 |
| PCDHGB3 | 2.831713545 | 2.999704803 | 6.125753591 | 4.09E-05 | 0.000629854 | 2.184832162 |
| IFITM3 | -2.470755994 | 15.10125139 | -6.124121257 | 4.10E-05 | 0.000630838 | 2.182186193 |
| OTUD7A | -1.441688737 | 3.7513582 | -6.118411801 | 4.14E-05 | 0.00063526 | 2.172928338 |
| TLR7 | -3.331891 | 5.773806339 | -6.115239288 | 4.16E-05 | 0.000637532 | 2.167782126 |
| IRF4 | -2.550036614 | 7.108819461 | -6.114785414 | 4.16E-05 | 0.000637532 | 2.167045768 |
| MEI1 | -2.600380172 | 10.48064436 | -6.114230585 | 4.17E-05 | 0.000637532 | 2.166145583 |
| PLXDC1 | -1.713733042 | 6.416991319 | -6.111868545 | 4.18E-05 | 0.00063927 | 2.162312793 |
| 7-Sep | -1.491011965 | 8.120776877 | -6.105344646 | 4.22E-05 | 0.000645245 | 2.1517226 |
| RUBCNL | -2.315900029 | 6.764175539 | -6.098940126 | 4.27E-05 | 0.000650533 | 2.141320318 |
| NARS2 | 1.278972037 | 8.805177687 | 6.09890985 | 4.27E-05 | 0.000650533 | 2.141271129 |
| CHEK1 | 2.099386394 | 5.392290048 | 6.096851952 | 4.28E-05 | 0.00065191 | 2.137927428 |
| ASPM | 3.407960042 | 5.9573288 | 6.096302631 | 4.29E-05 | 0.00065191 | 2.137034782 |
| ENOSF1 | 3.657945112 | 11.18090191 | 6.093531188 | 4.30E-05 | 0.000653648 | 2.132530535 |
| ZNF493 | 1.221974231 | 6.365981823 | 6.093353288 | 4.31E-05 | 0.000653648 | 2.132241368 |
| TMEM203 | -1.515953262 | 8.946245911 | -6.089878156 | 4.33E-05 | 0.000655917 | 2.126591834 |
| GSKIP | -1.148309468 | 6.67823974 | -6.089220962 | 4.33E-05 | 0.000655917 | 2.125523238 |
| SGK2 | 2.28058163 | 3.063327206 | 6.088919936 | 4.34E-05 | 0.000655917 | 2.12503375 |
| HSPG2 | 4.046759169 | 7.094186545 | 6.0886369 | 4.34E-05 | 0.000655917 | 2.124573504 |
| BMP6 | -1.409992187 | 6.210851381 | -6.087440169 | 4.35E-05 | 0.000656506 | 2.122627367 |
| WT1 | 2.751907381 | 4.899849668 | 6.084732753 | 4.36E-05 | 0.00065867 | 2.118223787 |
| NKRF | 1.069777857 | 8.545614206 | 6.081295611 | 4.39E-05 | 0.0006615 | 2.112631817 |
| B3GAT1 | -4.52995704 | 6.369631848 | -6.080771054 | 4.39E-05 | 0.0006615 | 2.111778255 |
| E2F7 | 3.257305368 | 4.028776562 | 6.076571808 | 4.42E-05 | 0.000665255 | 2.104943812 |
| SCAP | 1.007876417 | 10.77894635 | 6.075606389 | 4.43E-05 | 0.000665613 | 2.103372199 |
| HMGN4 | -1.449590128 | 9.695502891 | -6.074883242 | 4.43E-05 | 0.000665716 | 2.102194896 |
| RPRD1B | 1.395377836 | 8.61024902 | 6.073465666 | 4.44E-05 | 0.000666553 | 2.099886829 |
| SLC5A3 | 1.409332166 | 8.644212623 | 6.070536028 | 4.46E-05 | 0.000668995 | 2.095115951 |
| RPP38 | -1.154294856 | 7.713977623 | -6.067859781 | 4.48E-05 | 0.000670599 | 2.090756651 |
| SLC9A9 | -3.272541687 | 7.800489205 | -6.066980461 | 4.49E-05 | 0.000670599 | 2.089324118 |
| HPCAL4 | -2.04037894 | 3.401607442 | -6.066593101 | 4.49E-05 | 0.000670599 | 2.088693022 |
| ORC2 | 1.495736296 | 8.056807191 | 6.066536422 | 4.49E-05 | 0.000670599 | 2.088600676 |
| SMN1 | 1.303344829 | 9.260450097 | 6.062134456 | 4.52E-05 | 0.000674596 | 2.081427343 |
| MRAS | -3.231626041 | 5.726625943 | -6.060967935 | 4.53E-05 | 0.000674596 | 2.079525949 |
| TTC38 | -1.784489361 | 8.47405718 | -6.060931599 | 4.53E-05 | 0.000674596 | 2.079466719 |
| RIPPLY3 | 1.501768987 | 5.284433871 | 6.059110036 | 4.55E-05 | 0.000675885 | 2.07649723 |
| CAP1 | -1.004274542 | 9.59861303 | -6.055404529 | 4.57E-05 | 0.000679207 | 2.070455107 |
| GIMAP8 | -4.325693747 | 5.888707255 | -6.053767747 | 4.58E-05 | 0.000680308 | 2.067785584 |
| ST20 | -2.155247465 | 11.61748598 | -6.051010097 | 4.60E-05 | 0.000682626 | 2.063287112 |
| SNAPC1 | 1.056403791 | 6.456132035 | 6.048311408 | 4.62E-05 | 0.000684889 | 2.058883777 |
| LGALS2 | -6.128823354 | 11.521376 | -6.04630665 | 4.64E-05 | 0.000686403 | 2.05561203 |
| RUNX1 | 2.251380053 | 9.443385587 | 6.044125268 | 4.66E-05 | 0.000688115 | 2.052051387 |
| CYTL1 | 4.324331562 | 7.904773887 | 6.042271601 | 4.67E-05 | 0.000689473 | 2.049025139 |
| ZNF726 | 1.371560905 | 5.676496932 | 6.040644688 | 4.68E-05 | 0.000690586 | 2.046368682 |
| SEC14L4 | 2.16571117 | 7.318310261 | 6.032720883 | 4.74E-05 | 0.000698664 | 2.03342516 |
| GOLGA6D | 1.149132515 | 4.543729416 | 6.030011358 | 4.76E-05 | 0.000701001 | 2.028997113 |
| ZNF451 | 1.460304813 | 6.463607481 | 6.027672744 | 4.78E-05 | 0.000702933 | 2.02517439 |
| CNOT2 | 1.275028684 | 8.566150898 | 6.022723757 | 4.82E-05 | 0.000707806 | 2.017082168 |
| SPIN1 | 1.428220512 | 9.101944789 | 6.022097655 | 4.82E-05 | 0.000707826 | 2.016058163 |
| GPSM3 | -1.021509397 | 11.88070666 | -6.019070343 | 4.84E-05 | 0.000710559 | 2.011106142 |
| CDC26 | -1.207705055 | 10.72916606 | -6.016800336 | 4.86E-05 | 0.000712443 | 2.007392054 |
| TAF1 | 1.938211219 | 9.344853254 | 6.015762565 | 4.87E-05 | 0.000712615 | 2.005693854 |
| MEX3B | 2.190878608 | 5.792956426 | 6.01543688 | 4.87E-05 | 0.000712615 | 2.005160874 |
| KNTC1 | 1.145631795 | 4.844828369 | 6.012418399 | 4.90E-05 | 0.000714635 | 2.00022045 |
| GZMK | -4.413881141 | 10.05617665 | -6.010903545 | 4.91E-05 | 0.000714635 | 1.997740563 |
| GJD4 | 2.383155504 | 11.70385218 | 6.010896823 | 4.91E-05 | 0.000714635 | 1.997729558 |
| CD300E | -1.763968236 | 8.607678947 | -6.010640176 | 4.91E-05 | 0.000714635 | 1.997309382 |
| LSMEM1 | -2.39302675 | 6.975155532 | -6.00921003 | 4.92E-05 | 0.000715579 | 1.994967815 |
| FTHL17 | -1.187278979 | 13.29969592 | -6.006208495 | 4.94E-05 | 0.000717674 | 1.99005248 |
| ECE2 | 2.429454521 | 4.038211325 | 6.005962625 | 4.95E-05 | 0.000717674 | 1.989649784 |
| AP1M2 | -1.384472916 | 4.881502458 | -6.005573597 | 4.95E-05 | 0.000717674 | 1.989012603 |
| ABLIM2 | -1.63907018 | 4.074106736 | -6.002606498 | 4.97E-05 | 0.000720389 | 1.984152136 |
| NCOR1 | 1.046136008 | 6.948274671 | 6.000044172 | 4.99E-05 | 0.00072265 | 1.979953734 |
| PSMB11 | 1.260381224 | 4.196116975 | 5.99917949 | 5.00E-05 | 0.000722957 | 1.978536732 |
| MX1 | -3.211473562 | 10.85936279 | -5.997995331 | 5.01E-05 | 0.000723634 | 1.976596013 |
| LILRA6 | -2.862939337 | 9.681687169 | -5.996599337 | 5.02E-05 | 0.000723882 | 1.974307863 |
| PYDC1 | -2.195437632 | 8.702579596 | -5.996587114 | 5.02E-05 | 0.000723882 | 1.974287827 |
| S100A6 | -2.39944158 | 14.90895142 | -5.995628606 | 5.03E-05 | 0.000724301 | 1.972716591 |
| DENND2D | -1.567165897 | 10.76947412 | -5.993499315 | 5.05E-05 | 0.000725393 | 1.969225681 |
| ISCU | -1.116806634 | 12.37710194 | -5.993495714 | 5.05E-05 | 0.000725393 | 1.969219777 |
| COTL1 | -2.555980009 | 10.57760114 | -5.991912899 | 5.06E-05 | 0.000726539 | 1.966624381 |
| LENG8 | 1.843955558 | 10.36215632 | 5.989395084 | 5.08E-05 | 0.000728177 | 1.962495101 |
| ST20-MTHFS | -1.534505517 | 11.47324507 | -5.989316703 | 5.08E-05 | 0.000728177 | 1.96236654 |
| MPEG1 | -3.775856349 | 8.825769132 | -5.988686877 | 5.09E-05 | 0.000728221 | 1.961333462 |
| RNASE6 | -3.029671888 | 10.06167061 | -5.985245542 | 5.11E-05 | 0.000731544 | 1.955687787 |
| ZNF551 | 1.478553484 | 6.766141531 | 5.979638318 | 5.16E-05 | 0.0007373 | 1.946485273 |
| TLK2 | 2.52158434 | 9.811821333 | 5.978646364 | 5.17E-05 | 0.0007373 | 1.944856826 |
| SSR2 | -1.236829501 | 12.13070904 | -5.978571341 | 5.17E-05 | 0.0007373 | 1.944733658 |
| RNF157 | -2.492110415 | 4.632121713 | -5.975011483 | 5.20E-05 | 0.000740812 | 1.938888408 |
| KRT33B | 1.507202993 | 4.865537395 | 5.972296556 | 5.22E-05 | 0.000743336 | 1.93442932 |
| LILRB3 | -4.089962169 | 11.7521414 | -5.968765582 | 5.25E-05 | 0.000746845 | 1.928628364 |
| MACF1 | 2.769696372 | 8.893940525 | 5.967872802 | 5.26E-05 | 0.000747157 | 1.927161358 |
| ZNF302 | 1.839709914 | 8.212816638 | 5.967332227 | 5.26E-05 | 0.000747157 | 1.926273035 |
| ZNF195 | 1.381052545 | 9.650644634 | 5.963059354 | 5.30E-05 | 0.00075158 | 1.919250002 |
| ANAPC15 | -1.736301482 | 9.399966546 | -5.962112686 | 5.31E-05 | 0.000751658 | 1.917693677 |
| DLD | 1.758717964 | 6.684278382 | 5.961284472 | 5.31E-05 | 0.000751658 | 1.916331988 |
| CALM1 | -2.123063094 | 8.093950425 | -5.961242022 | 5.31E-05 | 0.000751658 | 1.916262193 |
| IL1R2 | -4.388065333 | 5.580121697 | -5.95816139 | 5.34E-05 | 0.000754162 | 1.911196368 |
| SPRYD3 | -1.856553934 | 8.29218252 | -5.958000586 | 5.34E-05 | 0.000754162 | 1.910931903 |
| SERPINC1 | -2.112674543 | 3.04293732 | -5.95495731 | 5.37E-05 | 0.000756871 | 1.905926118 |
| EARS2 | 1.017724809 | 8.942480144 | 5.954599044 | 5.37E-05 | 0.000756871 | 1.905336732 |
| CA11 | -1.314488451 | 7.573677519 | -5.942049355 | 5.48E-05 | 0.00077119 | 1.884679666 |
| KCNQ5 | 1.748286869 | 5.022575025 | 5.94136779 | 5.49E-05 | 0.00077119 | 1.883557158 |
| PLCL2 | -1.978111826 | 8.001315255 | -5.940941869 | 5.49E-05 | 0.00077119 | 1.882855649 |
| C16orf45 | -3.112134926 | 6.053210395 | -5.93960712 | 5.50E-05 | 0.00077119 | 1.880657101 |
| THAP5 | -1.407715546 | 4.942847365 | -5.939142131 | 5.50E-05 | 0.00077119 | 1.87989113 |
| WASHC3 | -1.248467921 | 10.2851099 | -5.938466515 | 5.51E-05 | 0.00077119 | 1.87877814 |
| COX14 | -1.196944836 | 14.27657658 | -5.93780729 | 5.52E-05 | 0.00077119 | 1.87769209 |
| DNAJC5B | -1.635885233 | 4.127355285 | -5.937467333 | 5.52E-05 | 0.00077119 | 1.877131999 |
| CAPRIN1 | 1.987236929 | 7.170599589 | 5.937271795 | 5.52E-05 | 0.00077119 | 1.876809836 |
| RAB31 | -4.60497217 | 9.676198165 | -5.936375785 | 5.53E-05 | 0.00077119 | 1.875333525 |
| SMARCC2 | 1.383750486 | 12.72639639 | 5.9362763 | 5.53E-05 | 0.00077119 | 1.875169602 |
| FBXO2 | -3.719252477 | 7.069776635 | -5.935986364 | 5.53E-05 | 0.00077119 | 1.87469186 |
| GATA3 | -4.205082281 | 5.16158987 | -5.934621746 | 5.54E-05 | 0.000771813 | 1.872443153 |
| RCC1 | 1.561462802 | 7.814385318 | 5.934337444 | 5.55E-05 | 0.000771813 | 1.871974628 |
| CRHR1 | 1.522378419 | 7.355284452 | 5.932943874 | 5.56E-05 | 0.000772831 | 1.869677885 |
| ACKR2 | 1.560499434 | 3.846807088 | 5.927321419 | 5.61E-05 | 0.000779124 | 1.860408718 |
| FTSJ3 | 1.093558905 | 10.31699988 | 5.925731851 | 5.62E-05 | 0.0007804 | 1.85778735 |
| GALNT7 | 1.538508385 | 8.706207149 | 5.921823611 | 5.66E-05 | 0.000784405 | 1.85134072 |
| AP1S1 | -1.992971394 | 6.583602013 | -5.92140658 | 5.66E-05 | 0.000784405 | 1.850652701 |
| MRGPRE | 1.476008245 | 8.310187156 | 5.914872777 | 5.72E-05 | 0.000791961 | 1.83987 |
| MEGF9 | -2.64394527 | 7.568599927 | -5.913513112 | 5.74E-05 | 0.00079297 | 1.837625393 |
| CLRN2 | -2.132277259 | 3.574985252 | -5.908292374 | 5.78E-05 | 0.000798926 | 1.829004291 |
| KLRC4 | -3.474663651 | 9.306299253 | -5.906409054 | 5.80E-05 | 0.00080062 | 1.825893384 |
| PLAGL1 | 2.236291264 | 6.334399811 | 5.902132113 | 5.84E-05 | 0.000805043 | 1.81882678 |
| TDRP | -1.61935657 | 3.062098071 | -5.90185468 | 5.84E-05 | 0.000805043 | 1.818368301 |
| LRCOL1 | 1.950035046 | 4.445581792 | 5.900621 | 5.86E-05 | 0.00080591 | 1.816329415 |
| DYRK1A | 1.07834207 | 9.671568156 | 5.899680962 | 5.87E-05 | 0.000806398 | 1.814775685 |
| TMEM229A | 2.786039623 | 3.290781958 | 5.897440915 | 5.89E-05 | 0.000808578 | 1.811072742 |
| MS4A1 | -2.583511861 | 8.09214172 | -5.895868637 | 5.90E-05 | 0.000809389 | 1.808473243 |
| TMEM272 | -1.552667525 | 3.773211291 | -5.895692987 | 5.90E-05 | 0.000809389 | 1.808182813 |
| CEP85L | -2.045126225 | 4.796440391 | -5.891498357 | 5.94E-05 | 0.000814139 | 1.801245877 |
| PF4 | -6.735306064 | 11.86723039 | -5.890598071 | 5.95E-05 | 0.000814584 | 1.79975669 |
| DDAH2 | 1.80782348 | 11.32957064 | 5.889544191 | 5.96E-05 | 0.000815231 | 1.798013293 |
| SVIP | -2.449009743 | 6.306574558 | -5.886244089 | 5.99E-05 | 0.000818839 | 1.792553038 |
| CDCA5 | 1.308156842 | 5.854419434 | 5.881732718 | 6.04E-05 | 0.000824072 | 1.785086159 |
| OR51I1 | 1.939370271 | 2.805342562 | 5.879827092 | 6.06E-05 | 0.000825864 | 1.781931247 |
| TNRC18 | 2.93212928 | 5.744747352 | 5.878546043 | 6.07E-05 | 0.000826799 | 1.779810082 |
| MAL | -4.529116914 | 10.71069302 | -5.878009908 | 6.07E-05 | 0.000826799 | 1.77892228 |
| DEPDC7 | 3.223157604 | 5.244892612 | 5.876975421 | 6.08E-05 | 0.000827061 | 1.777209126 |
| SPRY2 | 3.573986129 | 7.17323703 | 5.876700319 | 6.09E-05 | 0.000827061 | 1.77675352 |
| WDR6 | 1.458922772 | 9.702877774 | 5.87484311 | 6.10E-05 | 0.000828255 | 1.773677453 |
| TPRG1L | -1.7468419 | 8.224628827 | -5.874695328 | 6.11E-05 | 0.000828255 | 1.773432662 |
| RNF212 | 2.631215892 | 6.604300427 | 5.866287199 | 6.19E-05 | 0.000838819 | 1.759500129 |
| CYP27B1 | 1.246953744 | 5.304560154 | 5.860572706 | 6.25E-05 | 0.000845791 | 1.750025336 |
| ARID1B | 1.557583768 | 6.892565963 | 5.86005445 | 6.25E-05 | 0.000845791 | 1.749165824 |
| CD6 | -3.48765244 | 11.7767608 | -5.859471518 | 6.26E-05 | 0.000845832 | 1.748199005 |
| ZMIZ1 | 1.113185469 | 8.384768924 | 5.85843676 | 6.27E-05 | 0.000846492 | 1.746482693 |
| ATP7A | 1.479302176 | 7.172492302 | 5.853102619 | 6.32E-05 | 0.000851401 | 1.737632765 |
| GABBR1 | -1.579677568 | 5.562923325 | -5.852856353 | 6.33E-05 | 0.000851401 | 1.737224085 |
| TMEM176B | -3.737699023 | 9.505347812 | -5.85265598 | 6.33E-05 | 0.000851401 | 1.736891558 |
| OLR1 | -2.012564683 | 2.609328622 | -5.851587086 | 6.34E-05 | 0.000852116 | 1.735117595 |
| ZNF511 | 2.549051594 | 7.85982049 | 5.850474285 | 6.35E-05 | 0.000852894 | 1.733270591 |
| ZKSCAN8 | 2.073232202 | 5.9990396 | 5.849380255 | 6.36E-05 | 0.000853647 | 1.731454573 |
| MLF1 | -1.842836053 | 4.141933759 | -5.845577643 | 6.40E-05 | 0.000858152 | 1.725141169 |
| ARL5B | 2.036625025 | 8.144246905 | 5.843723596 | 6.42E-05 | 0.00085951 | 1.722062191 |
| HELLS | 2.160351493 | 6.955887527 | 5.843506828 | 6.42E-05 | 0.00085951 | 1.721702177 |
| ATL2 | 1.87154524 | 7.77750587 | 5.842786024 | 6.43E-05 | 0.000859753 | 1.720504999 |
| USP54 | 1.25588965 | 7.476161518 | 5.84151627 | 6.44E-05 | 0.000860342 | 1.718395897 |
| HHIP | 1.88509565 | 2.94721015 | 5.841272357 | 6.45E-05 | 0.000860342 | 1.717990723 |
| BOK | -2.082194977 | 6.447048989 | -5.840655937 | 6.45E-05 | 0.000860442 | 1.716966726 |
| EAPP | -1.240144104 | 11.12396771 | -5.837605181 | 6.48E-05 | 0.00086394 | 1.71189802 |
| JUP | 1.918337649 | 10.6111861 | 5.836866537 | 6.49E-05 | 0.000864213 | 1.710670597 |
| CCDC88A | 1.563987716 | 8.007945364 | 5.833708991 | 6.52E-05 | 0.000867879 | 1.705422752 |
| CCNA1 | 5.382734527 | 5.793907164 | 5.832729073 | 6.53E-05 | 0.000868495 | 1.703793842 |
| CNNM4 | 1.322158646 | 9.727682473 | 5.829696994 | 6.57E-05 | 0.000872004 | 1.698752781 |
| CD2 | -2.175553846 | 6.815410654 | -5.82747956 | 6.59E-05 | 0.000874375 | 1.695065309 |
| USP42 | 2.412807151 | 7.499663097 | 5.818951253 | 6.68E-05 | 0.000885767 | 1.680876735 |
| ATP6AP2 | -1.215735617 | 11.45780872 | -5.810343872 | 6.78E-05 | 0.000897431 | 1.66654621 |
| TNFSF13 | -3.095361325 | 8.995596084 | -5.808417843 | 6.80E-05 | 0.000899454 | 1.663338113 |
| AKAP9 | 2.320349059 | 6.181106181 | 5.806538432 | 6.82E-05 | 0.000901413 | 1.660207161 |
| SYNGAP1 | 1.407245841 | 6.398230198 | 5.800402224 | 6.89E-05 | 0.000909643 | 1.649981255 |
| ERLIN1 | 2.657944081 | 7.658113798 | 5.799389894 | 6.90E-05 | 0.000910345 | 1.648293712 |
| GIMAP6 | -3.392514957 | 8.593797934 | -5.795700608 | 6.94E-05 | 0.000915 | 1.642142488 |
| LIG1 | 1.591621905 | 10.63768564 | 5.795177936 | 6.95E-05 | 0.000915 | 1.641270871 |
| UFSP2 | -1.541880912 | 7.903974243 | -5.789266078 | 7.01E-05 | 0.000923028 | 1.631409475 |
| ANKRD36C | 2.004340082 | 8.399629467 | 5.788078971 | 7.03E-05 | 0.000924008 | 1.629428705 |
| ZNF609 | 1.455817656 | 6.139948404 | 5.787025977 | 7.04E-05 | 0.000924788 | 1.627671545 |
| JAM3 | -2.332566772 | 5.759376783 | -5.786351089 | 7.05E-05 | 0.000925001 | 1.626545258 |
| L2HGDH | 1.980192298 | 7.450898301 | 5.78181239 | 7.10E-05 | 0.000931045 | 1.618969188 |
| PRSS38 | 1.900402 | 5.195766246 | 5.774715293 | 7.18E-05 | 0.000941041 | 1.607116789 |
| CHST7 | -2.443864706 | 8.239226566 | -5.772610221 | 7.20E-05 | 0.000943454 | 1.603599883 |
| NPIPB9 | 1.645271811 | 13.48090257 | 5.767713429 | 7.26E-05 | 0.000950183 | 1.595416489 |
| FBXO17 | 1.890177503 | 2.58323475 | 5.760617208 | 7.35E-05 | 0.000960399 | 1.583551481 |
| NELL2 | -4.753526492 | 7.953018004 | -5.753188216 | 7.44E-05 | 0.000971259 | 1.571122492 |
| TMX2 | 1.140720568 | 12.53461152 | 5.751533452 | 7.46E-05 | 0.000972402 | 1.568352951 |
| ALG10B | 1.484225531 | 7.062103793 | 5.751066288 | 7.46E-05 | 0.000972402 | 1.567571 |
| COL8A2 | -1.804052522 | 5.469358414 | -5.750882499 | 7.46E-05 | 0.000972402 | 1.567263361 |
| CCDC125 | 1.602178731 | 9.876421447 | 5.749455947 | 7.48E-05 | 0.000973306 | 1.564875336 |
| AC008758.1 | 1.618571006 | 6.013344397 | 5.74446226 | 7.54E-05 | 0.00098011 | 1.556513727 |
| TTC39C | -1.594588482 | 9.794328938 | -5.74284341 | 7.56E-05 | 0.000981845 | 1.553802315 |
| STXBP4 | 1.409086902 | 3.600634139 | 5.742327614 | 7.57E-05 | 0.000981845 | 1.552938332 |
| ZBTB20 | 1.765664755 | 7.588079934 | 5.739608979 | 7.60E-05 | 0.000984884 | 1.548383865 |
| CPQ | -1.811283227 | 8.7458664 | -5.739385285 | 7.61E-05 | 0.000984884 | 1.548009069 |
| SCCPDH | 1.870723046 | 12.01797197 | 5.737555285 | 7.63E-05 | 0.000986987 | 1.54494267 |
| XBP1 | 1.052954666 | 13.954138 | 5.736014878 | 7.65E-05 | 0.000988628 | 1.542361159 |
| UFC1 | -1.319082513 | 8.081848042 | -5.735476777 | 7.65E-05 | 0.000988653 | 1.541459296 |
| SRP54 | 1.618646389 | 8.02558261 | 5.734580102 | 7.67E-05 | 0.000989258 | 1.539956368 |
| LRRC37A3 | 1.873376738 | 6.676044536 | 5.732309529 | 7.69E-05 | 0.000992088 | 1.536150132 |
| IDH3G | -1.169537287 | 10.64000955 | -5.731499257 | 7.70E-05 | 0.000992557 | 1.534791671 |
| RP9 | -1.2451822 | 9.037622487 | -5.726815108 | 7.76E-05 | 0.000999331 | 1.52693666 |
| CCDC92 | -1.965206482 | 9.019007159 | -5.721339444 | 7.83E-05 | 0.001006388 | 1.517750426 |
| BAZ2B | 1.920312944 | 6.986762519 | 5.720890323 | 7.84E-05 | 0.001006388 | 1.516996772 |
| PPIP5K2 | 1.547415895 | 9.35227793 | 5.720660979 | 7.84E-05 | 0.001006388 | 1.516611908 |
| CSRP1 | -2.198606876 | 8.863834887 | -5.719930137 | 7.85E-05 | 0.001006388 | 1.515385425 |
| ZNF566 | 1.418885268 | 4.739222913 | 5.719916084 | 7.85E-05 | 0.001006388 | 1.515361841 |
| EBLN2 | 2.647088418 | 9.493251247 | 5.717739987 | 7.88E-05 | 0.001008474 | 1.511709515 |
| IQCN | 1.21927661 | 7.027951857 | 5.717615165 | 7.88E-05 | 0.001008474 | 1.511499996 |
| CSTA | -3.929344613 | 11.09787476 | -5.716885198 | 7.89E-05 | 0.001008827 | 1.51027467 |
| GTF3C3 | 1.190355565 | 7.591083331 | 5.71631858 | 7.90E-05 | 0.001008911 | 1.50932349 |
| ALDH4A1 | 1.188919864 | 7.479457093 | 5.715013727 | 7.91E-05 | 0.001010214 | 1.507132868 |
| G6PD | -2.01277906 | 8.222180708 | -5.711480224 | 7.96E-05 | 0.001015215 | 1.501199529 |
| SPRY4 | 1.387541746 | 7.049157532 | 5.707249692 | 8.02E-05 | 0.001020735 | 1.494093462 |
| MRPS25 | 1.461682292 | 9.01828438 | 5.707138481 | 8.02E-05 | 0.001020735 | 1.493906626 |
| MINDY1 | -1.676532883 | 10.36481862 | -5.705026932 | 8.04E-05 | 0.001022905 | 1.490358864 |
| B3GLCT | -1.969598815 | 5.629179698 | -5.70481505 | 8.05E-05 | 0.001022905 | 1.490002832 |
| AC108488.2 | -2.751267963 | 5.733255069 | -5.701728041 | 8.09E-05 | 0.001027226 | 1.484814916 |
| PLCXD1 | 1.385552523 | 9.398340795 | 5.699489205 | 8.12E-05 | 0.001029535 | 1.481051574 |
| ZNF32 | -2.02683132 | 8.084355442 | -5.699334261 | 8.12E-05 | 0.001029535 | 1.480791098 |
| RB1CC1 | 1.966111886 | 9.630499198 | 5.698523597 | 8.13E-05 | 0.00103004 | 1.479428231 |
| TFRC | 2.789047834 | 11.10618776 | 5.696484386 | 8.16E-05 | 0.001031889 | 1.47599956 |
| SNAP47 | -1.120099953 | 8.993794293 | -5.696408644 | 8.16E-05 | 0.001031889 | 1.475872199 |
| ADM | -2.211833815 | 10.27639717 | -5.695543737 | 8.17E-05 | 0.001032491 | 1.474417783 |
| ZC2HC1A | -2.39406621 | 4.336937539 | -5.694838992 | 8.18E-05 | 0.001032822 | 1.473232617 |
| ADAMTS14 | 3.383070433 | 5.133634004 | 5.691886586 | 8.22E-05 | 0.001036582 | 1.468266812 |
| MED11 | -1.11174025 | 9.220043727 | -5.691605113 | 8.22E-05 | 0.001036582 | 1.467793325 |
| WARS | -2.080200624 | 10.52821578 | -5.690765399 | 8.24E-05 | 0.001037147 | 1.466380711 |
| AMZ1 | 2.336550192 | 9.691996527 | 5.689417125 | 8.25E-05 | 0.001038337 | 1.464112362 |
| AL512428.1 | -1.279526275 | 5.494810058 | -5.689052332 | 8.26E-05 | 0.001038337 | 1.463498586 |
| DPY19L4 | 1.628388432 | 4.023422841 | 5.686972678 | 8.29E-05 | 0.001041019 | 1.459999151 |
| LST1 | -3.91266634 | 11.86802274 | -5.685094151 | 8.31E-05 | 0.001043365 | 1.456837633 |
| KRT5 | -1.741921661 | 3.740987076 | -5.682018919 | 8.35E-05 | 0.001047772 | 1.451661022 |
| NANOG | 3.618020465 | 3.478557353 | 5.677521391 | 8.42E-05 | 0.001054658 | 1.444087843 |
| DDX17 | 1.536189816 | 10.64237605 | 5.67681396 | 8.43E-05 | 0.00105501 | 1.442896375 |
| IGLL1 | 4.927294772 | 8.435162633 | 5.675055709 | 8.45E-05 | 0.001056393 | 1.439934795 |
| MTFP1 | -2.307643548 | 9.281081168 | -5.67478154 | 8.45E-05 | 0.001056393 | 1.439472947 |
| NFKBIE | -1.407051128 | 11.30192686 | -5.674506936 | 8.46E-05 | 0.001056393 | 1.439010357 |
| PCOLCE2 | -1.702399603 | 3.50185016 | -5.673932028 | 8.47E-05 | 0.001056519 | 1.438041851 |
| CACNA1F | 1.129441561 | 4.901045911 | 5.672311489 | 8.49E-05 | 0.001058461 | 1.435311593 |
| MBP | -1.124719202 | 5.764549068 | -5.668451302 | 8.54E-05 | 0.001064315 | 1.428806536 |
| AGMAT | -1.856764894 | 7.254059718 | -5.665199607 | 8.59E-05 | 0.001069134 | 1.423325272 |
| SCOC | -1.369213204 | 6.545011861 | -5.664541803 | 8.60E-05 | 0.001069411 | 1.422216257 |
| FAM126A | -1.107529739 | 6.368798903 | -5.663226999 | 8.62E-05 | 0.001069967 | 1.4199994 |
| CIB1 | -1.254663723 | 11.91650363 | -5.660173266 | 8.66E-05 | 0.001074468 | 1.414849647 |
| TNFAIP8 | -1.764591063 | 10.06887039 | -5.658673024 | 8.68E-05 | 0.001075791 | 1.412319192 |
| DPEP2 | -1.925214697 | 10.14486054 | -5.658428159 | 8.68E-05 | 0.001075791 | 1.411906149 |
| RHOU | -3.412883094 | 5.167638209 | -5.656996191 | 8.70E-05 | 0.001077445 | 1.409490511 |
| MLXIP | 1.480258501 | 10.8253814 | 5.654069423 | 8.75E-05 | 0.001081758 | 1.404552347 |
| COMMD10 | -1.441833929 | 9.019492343 | -5.653015461 | 8.76E-05 | 0.00108275 | 1.402773767 |
| OSBPL10 | -2.358541148 | 6.099224993 | -5.650609566 | 8.80E-05 | 0.001085743 | 1.398713193 |
| ZBTB43 | 2.478985092 | 10.55938803 | 5.650345431 | 8.80E-05 | 0.001085743 | 1.398267347 |
| ZNF479 | -1.7591286 | 3.39714942 | -5.649380516 | 8.81E-05 | 0.001086583 | 1.396638541 |
| GTF2I | 1.727399356 | 12.17642495 | 5.645533602 | 8.87E-05 | 0.001092592 | 1.390143532 |
| ING5 | 1.176929712 | 7.092638679 | 5.642809423 | 8.91E-05 | 0.001096609 | 1.385542867 |
| MCM3 | 1.732741459 | 11.28995072 | 5.640603016 | 8.94E-05 | 0.001099705 | 1.38181587 |
| RBM28 | 1.041327318 | 8.288737899 | 5.639578882 | 8.96E-05 | 0.001100667 | 1.380085703 |
| METTL18 | -1.596066602 | 7.515298107 | -5.63862067 | 8.97E-05 | 0.001101511 | 1.378466771 |
| GADD45B | -1.53536475 | 9.175291712 | -5.637664105 | 8.99E-05 | 0.001102354 | 1.376850493 |
| FCGR2A | -3.497481546 | 8.881927485 | -5.634007946 | 9.04E-05 | 0.001108113 | 1.370671625 |
| EOMES | -3.723225848 | 5.818404505 | -5.632583022 | 9.06E-05 | 0.001109819 | 1.368263014 |
| ALDH1A1 | -2.653340559 | 7.502057138 | -5.628310767 | 9.13E-05 | 0.001116754 | 1.36103974 |
| AFF1 | 1.94815196 | 8.645971363 | 5.627345278 | 9.14E-05 | 0.00111763 | 1.359406996 |
| RANBP2 | 1.965215594 | 6.983155239 | 5.625727481 | 9.16E-05 | 0.001119418 | 1.35667084 |
| MS4A7 | -3.012073934 | 9.993433417 | -5.625396406 | 9.17E-05 | 0.001119418 | 1.356110853 |
| GCN1 | 1.218172765 | 9.97459359 | 5.623874586 | 9.19E-05 | 0.001120721 | 1.353536619 |
| AL645922.1 | -2.369306655 | 5.45954874 | -5.623713602 | 9.19E-05 | 0.001120721 | 1.353264288 |
| ACTL10 | -1.021866974 | 6.924051119 | -5.622899743 | 9.21E-05 | 0.001121323 | 1.351887453 |
| PAXBP1 | 1.992188328 | 7.863176227 | 5.614957074 | 9.33E-05 | 0.001134283 | 1.33844572 |
| CLP1 | -1.394544419 | 7.797557082 | -5.611907274 | 9.38E-05 | 0.001138555 | 1.333282072 |
| ZFYVE28 | -1.671147765 | 5.031862853 | -5.611706273 | 9.38E-05 | 0.001138555 | 1.33294171 |
| NFATC2IP | 1.893025702 | 8.537211383 | 5.609291348 | 9.42E-05 | 0.001142186 | 1.328851991 |
| LARGE1 | -1.507283853 | 7.849389722 | -5.606125388 | 9.47E-05 | 0.001147251 | 1.32348915 |
| CDK4 | 1.481114977 | 11.13487683 | 5.604603066 | 9.49E-05 | 0.001149219 | 1.320909984 |
| PAK1 | -2.10202092 | 9.879180184 | -5.603756421 | 9.50E-05 | 0.001149907 | 1.31947543 |
| FAM180B | 1.427094689 | 7.925476686 | 5.603186324 | 9.51E-05 | 0.001150072 | 1.318509404 |
| PPFIBP1 | 2.399507822 | 5.019054244 | 5.602448742 | 9.52E-05 | 0.001150555 | 1.317259506 |
| DEFB131B | 1.377853311 | 5.549698061 | 5.599853532 | 9.56E-05 | 0.001153725 | 1.312861097 |
| LIG4 | -1.43766913 | 2.677180324 | -5.599444611 | 9.57E-05 | 0.001153725 | 1.312167966 |
| DNM3 | -3.136443478 | 7.347450337 | -5.599335899 | 9.57E-05 | 0.001153725 | 1.311983691 |
| KRTDAP | 1.088509125 | 2.049161736 | 5.598587662 | 9.58E-05 | 0.001154234 | 1.310715337 |
| TMEM35B | -1.01760917 | 9.27711257 | -5.595373532 | 9.63E-05 | 0.001159457 | 1.305266108 |
| AP000781.2 | 3.645675157 | 6.348227407 | 5.594008823 | 9.66E-05 | 0.001161152 | 1.302951949 |
| TIMD4 | -2.338596782 | 5.744779687 | -5.593101267 | 9.67E-05 | 0.001161973 | 1.301412849 |
| HRK | -2.218261756 | 3.804059388 | -5.590700145 | 9.71E-05 | 0.001165669 | 1.297340295 |
| ATAD2 | 2.124229392 | 8.593069301 | 5.589422309 | 9.73E-05 | 0.001167209 | 1.295172626 |
| PGF | 4.083249402 | 4.494553328 | 5.588515041 | 9.74E-05 | 0.001167669 | 1.293633435 |
| SLC6A20 | 1.4915439 | 2.823965863 | 5.587913601 | 9.75E-05 | 0.001167669 | 1.292613023 |
| CCR1 | -3.557524827 | 8.411987976 | -5.587587917 | 9.76E-05 | 0.001167669 | 1.292060441 |
| ST14 | -1.283401154 | 8.712056724 | -5.587271192 | 9.76E-05 | 0.001167669 | 1.291523046 |
| MAP4K3 | 2.785592288 | 4.373757376 | 5.586198379 | 9.78E-05 | 0.00116882 | 1.289702674 |
| OGA | 2.098410113 | 11.66504723 | 5.584968928 | 9.80E-05 | 0.001170276 | 1.287616319 |
| SORBS1 | 2.524564981 | 3.864505506 | 5.582668517 | 9.84E-05 | 0.001173812 | 1.283712 |
| ZDHHC9 | 1.845816662 | 7.714066239 | 5.581373206 | 9.86E-05 | 0.001174536 | 1.281513241 |
| ZNF426 | 1.302025815 | 6.282193022 | 5.581344451 | 9.86E-05 | 0.001174536 | 1.281464427 |
| CENPJ | 1.699090567 | 4.90925047 | 5.579392534 | 9.89E-05 | 0.001177409 | 1.278150647 |
| TMEM154 | -3.066051715 | 6.873431086 | -5.578621357 | 9.91E-05 | 0.001177987 | 1.276841271 |
| MED27 | -1.057999501 | 6.925514885 | -5.576825979 | 9.93E-05 | 0.00117964 | 1.273792588 |
| OLFM4 | -5.709211014 | 5.669428706 | -5.575052031 | 9.96E-05 | 0.001182182 | 1.270779857 |
| KATNAL1 | 1.405381537 | 7.858199575 | 5.57449508 | 9.97E-05 | 0.001182347 | 1.269833887 |
| MTR | 1.584307049 | 6.137681821 | 5.567386937 | 0.000100913 | 0.001195192 | 1.257757058 |
| FAM160B1 | 1.765199506 | 8.107882842 | 5.566631522 | 0.000101039 | 0.001195192 | 1.256473185 |
| RASGRF2 | -1.572199021 | 4.438728612 | -5.566553115 | 0.000101052 | 0.001195192 | 1.256339924 |
| APMAP | -1.795577836 | 8.374541443 | -5.564336686 | 0.000101424 | 0.001198652 | 1.252572492 |
| RAP1GAP | 2.608778003 | 4.8553204 | 5.56363914 | 0.000101541 | 0.001199103 | 1.25138668 |
| NDUFAF2 | -1.325072471 | 9.609534682 | -5.561818112 | 0.000101848 | 0.001201789 | 1.248290656 |
| PIGM | 2.136826234 | 5.549566568 | 5.560902527 | 0.000102003 | 0.001202677 | 1.24673385 |
| MATK | -1.712433729 | 9.573251457 | -5.558569819 | 0.000102398 | 0.001206367 | 1.242766925 |
| TRPM6 | -1.113675744 | 3.130232736 | -5.557771587 | 0.000102533 | 0.001206367 | 1.241409306 |
| HSPD1 | 1.642851996 | 12.43345728 | 5.557647418 | 0.000102554 | 0.001206367 | 1.241198113 |
| AIM2 | -4.86461563 | 6.9974102 | -5.5473892 | 0.000104314 | 0.001226112 | 1.223743081 |
| C17orf97 | 3.920950054 | 5.334866819 | 5.546732323 | 0.000104428 | 0.001226497 | 1.222624864 |
| PTPRK | -1.420310589 | 6.240274552 | -5.545901127 | 0.000104572 | 0.001227239 | 1.221209817 |
| ZNF280C | 1.230130151 | 6.533742313 | 5.544014452 | 0.0001049 | 0.001230134 | 1.217997541 |
| ZNF763 | 2.844954069 | 10.87532349 | 5.541578611 | 0.000105324 | 0.001234162 | 1.21384952 |
| EMC3 | -1.387373667 | 10.34423042 | -5.539405255 | 0.000105705 | 0.001237665 | 1.210147796 |
| RASSF6 | 1.988791684 | 6.685078315 | 5.538237754 | 0.00010591 | 0.001239109 | 1.208159003 |
| IL2RA | -3.158499934 | 5.453741852 | -5.536096855 | 0.000106287 | 0.001242144 | 1.204511577 |
| MST1R | 1.14094641 | 5.021209087 | 5.535835026 | 0.000106334 | 0.001242144 | 1.204065458 |
| LINGO3 | -1.171274109 | 6.328427867 | -5.533915802 | 0.000106673 | 0.001245149 | 1.200795089 |
| TMEM132E | -1.842102015 | 2.958587431 | -5.532094888 | 0.000106996 | 0.00124796 | 1.197691769 |
| CENPF | 3.92875513 | 8.377238524 | 5.529047463 | 0.000107539 | 0.001252366 | 1.192497126 |
| LRG1 | -1.893064371 | 7.92680541 | -5.526713455 | 0.000107957 | 0.001256267 | 1.188517705 |
| CD300C | -2.354368057 | 8.32508859 | -5.525181118 | 0.000108232 | 0.001257845 | 1.185904705 |
| TIAL1 | 1.034214875 | 6.689709276 | 5.525034357 | 0.000108259 | 0.001257845 | 1.185654423 |
| BRK1 | -1.182670724 | 10.84264158 | -5.519574546 | 0.000109246 | 0.001266811 | 1.176341393 |
| AC092718.8 | 1.461992201 | 5.967520122 | 5.519357284 | 0.000109286 | 0.001266811 | 1.175970714 |
| NACC2 | -1.709590756 | 7.602224199 | -5.518987876 | 0.000109353 | 0.001266811 | 1.17534044 |
| EIF3K | -1.221995257 | 13.32969706 | -5.51891998 | 0.000109365 | 0.001266811 | 1.175224596 |
| TMEM98 | 1.335992947 | 5.800755916 | 5.518013716 | 0.00010953 | 0.001267341 | 1.173678262 |
| ALDH18A1 | 1.10706213 | 8.904985974 | 5.5177499 | 0.000109578 | 0.001267341 | 1.1732281 |
| ERRFI1 | -2.24834898 | 4.633985946 | -5.516096415 | 0.00010988 | 0.001269862 | 1.170406452 |
| WDFY2 | 2.013047518 | 8.516078529 | 5.512343847 | 0.000110568 | 0.00127684 | 1.164001352 |
| DEFA3 | -5.559347768 | 14.16647246 | -5.511643463 | 0.000110697 | 0.001277337 | 1.16280568 |
| AVPI1 | -1.194215389 | 8.241397231 | -5.511194646 | 0.00011078 | 0.001277337 | 1.16203944 |
| USP43 | 3.85747229 | 3.69591478 | 5.508965162 | 0.000111191 | 0.001280961 | 1.158232754 |
| C14orf180 | 1.318732301 | 5.562900704 | 5.508578429 | 0.000111263 | 0.001280961 | 1.157572365 |
| LILRA2 | -2.870932033 | 8.469104288 | -5.507970123 | 0.000111376 | 0.001281285 | 1.156533575 |
| BICC1 | 2.507634961 | 5.285709558 | 5.507459242 | 0.00011147 | 0.001281402 | 1.155661116 |
| ANKRD11 | 1.594420132 | 9.361167399 | 5.50574111 | 0.00011179 | 0.001284098 | 1.152726702 |
| EGFL7 | 3.748253174 | 11.0475337 | 5.504638719 | 0.000111995 | 0.001285483 | 1.150843705 |
| SPOCK1 | -2.459340888 | 2.811562349 | -5.503268809 | 0.000112251 | 0.001287443 | 1.148503526 |
| SCAI | 1.273850339 | 7.897002323 | 5.498993121 | 0.000113053 | 0.00129541 | 1.14119782 |
| SLC30A4 | -1.627349498 | 4.34839831 | -5.498656225 | 0.000113116 | 0.00129541 | 1.140622071 |
| EFCAB2 | 1.322493558 | 7.028785831 | 5.497845657 | 0.000113269 | 0.001296181 | 1.139236763 |
| C10orf143 | -2.530382717 | 9.117839432 | -5.495474169 | 0.000113718 | 0.00130033 | 1.13518323 |
| PTAFR | -2.346048791 | 10.02114319 | -5.492860847 | 0.000114214 | 0.001304037 | 1.130715441 |
| MFSD5 | -1.56679705 | 9.047161586 | -5.483396717 | 0.000116031 | 0.001323171 | 1.114527506 |
| NCF2 | -4.924766476 | 10.11721834 | -5.483221163 | 0.000116065 | 0.001323171 | 1.114227112 |
| AKAP14 | 1.265730135 | 5.486312474 | 5.480902293 | 0.000116514 | 0.001327301 | 1.110258863 |
| CAVIN1 | 1.585773152 | 11.84583729 | 5.479604515 | 0.000116767 | 0.001329178 | 1.108037672 |
| SDR42E1 | -2.666298913 | 3.942902378 | -5.476755686 | 0.000117323 | 0.00133285 | 1.103160991 |
| PUM1 | 1.26605262 | 10.87237942 | 5.476091759 | 0.000117453 | 0.00133285 | 1.102024308 |
| NUP155 | 1.190466653 | 7.218009038 | 5.475895751 | 0.000117492 | 0.00133285 | 1.101688718 |
| MCM3AP | 1.217283923 | 9.860378887 | 5.475724505 | 0.000117525 | 0.00133285 | 1.10139552 |
| KCTD3 | 2.692280742 | 5.725647211 | 5.475704751 | 0.000117529 | 0.00133285 | 1.101361699 |
| SASH1 | -2.272398701 | 2.744339913 | -5.472822244 | 0.000118096 | 0.001338276 | 1.096425817 |
| HUS1B | -1.181176505 | 6.355962805 | -5.472057208 | 0.000118247 | 0.001338985 | 1.095115611 |
| IKZF3 | -1.490368785 | 5.242286126 | -5.471463525 | 0.000118364 | 0.001339312 | 1.094098808 |
| GREM2 | -1.351246833 | 4.149925654 | -5.471009171 | 0.000118454 | 0.001339328 | 1.093320603 |
| LEFTY2 | -1.433437059 | 2.797265239 | -5.466971761 | 0.000119256 | 0.001347384 | 1.086404191 |
| PTPRJ | -2.248043442 | 5.920528039 | -5.463716611 | 0.000119906 | 0.001353721 | 1.080826227 |
| MAD1L1 | -1.39410527 | 9.18688473 | -5.462319481 | 0.000120186 | 0.001355874 | 1.078431684 |
| ITGB7 | -1.297731177 | 12.52204607 | -5.461147606 | 0.000120422 | 0.001357521 | 1.076423001 |
| DNAJC17 | -2.598308716 | 9.648825018 | -5.456746333 | 0.000121311 | 0.001365512 | 1.068877199 |
| NKX6-3 | 1.221420664 | 6.063029395 | 5.455238361 | 0.000121617 | 0.001367942 | 1.066291232 |
| LCE2C | 1.362121334 | 2.600598481 | 5.452766232 | 0.000122121 | 0.001372589 | 1.062051192 |
| USP36 | 1.877313206 | 10.18063584 | 5.45156488 | 0.000122366 | 0.001373413 | 1.059990408 |
| LIPA | -1.817335816 | 11.96464628 | -5.451520786 | 0.000122375 | 0.001373413 | 1.059914765 |
| SLC38A7 | 1.593766896 | 9.316625818 | 5.450320636 | 0.000122621 | 0.001374553 | 1.057855837 |
| C1QA | -3.700038171 | 6.489836756 | -5.450003497 | 0.000122686 | 0.001374553 | 1.057311733 |
| MYO1C | 2.342759102 | 14.91128133 | 5.449697941 | 0.000122749 | 0.001374553 | 1.05678749 |
| APOL1 | -1.032223789 | 9.228356858 | -5.444996886 | 0.000123718 | 0.001384383 | 1.048720252 |
| MS4A14 | -2.952808171 | 5.802705524 | -5.443814032 | 0.000123963 | 0.001386102 | 1.046689942 |
| ZNF512B | 1.412437018 | 6.211942836 | 5.441189654 | 0.000124509 | 0.001391177 | 1.042184643 |
| FCRL3 | -4.006167451 | 8.206725137 | -5.439702692 | 0.000124819 | 0.001393616 | 1.039631538 |
| ZGRF1 | 2.682395733 | 2.84130604 | 5.438638442 | 0.000125042 | 0.001395073 | 1.037804044 |
| GALM | -2.606610896 | 7.134789876 | -5.436746557 | 0.000125439 | 0.001398469 | 1.034554979 |
| KIF2C | 2.582615612 | 8.896059274 | 5.435592383 | 0.000125681 | 0.001400145 | 1.032572595 |
| CALCA | 1.804421382 | 4.437198809 | 5.434759115 | 0.000125857 | 0.00140107 | 1.03114128 |
| SBNO1 | 1.296461793 | 8.465727734 | 5.434288901 | 0.000125956 | 0.001401144 | 1.030333545 |
| ZNF337 | 1.104291893 | 7.927661675 | 5.430717219 | 0.000126712 | 0.001408518 | 1.024197114 |
| STARD13 | 2.205209262 | 3.796138888 | 5.429777919 | 0.000126911 | 0.001409702 | 1.022583033 |
| KLF4 | -2.232365576 | 7.196897235 | -5.428197376 | 0.000127248 | 0.001412404 | 1.019866776 |
| TMEM40 | -1.078812203 | 8.90200964 | -5.426518021 | 0.000127606 | 0.001415348 | 1.016980331 |
| AMER3 | 3.83916469 | 4.564642494 | 5.423127407 | 0.000128334 | 0.001422373 | 1.011151432 |
| ABCD3 | -1.183267005 | 7.566835711 | -5.421832169 | 0.000128613 | 0.001423985 | 1.008924337 |
| CNGA4 | 1.565732672 | 3.986036542 | 5.421196318 | 0.00012875 | 0.001423985 | 1.00783094 |
| CLTB | -1.699996567 | 6.806531005 | -5.420836179 | 0.000128828 | 0.001423985 | 1.00721163 |
| MVP | -1.411079857 | 13.58101974 | -5.420709214 | 0.000128855 | 0.001423985 | 1.006993289 |
| CELA1 | -3.719165106 | 7.04965572 | -5.418073332 | 0.000129426 | 0.00142925 | 1.002459917 |
| RCOR1 | 1.538832073 | 9.969228963 | 5.417277479 | 0.000129599 | 0.001430117 | 1.001090967 |
| DDX39A | 1.215922875 | 12.96766876 | 5.415660689 | 0.000129951 | 0.001432723 | 0.998309655 |
| SOX11 | 1.293521217 | 4.080202768 | 5.415324075 | 0.000130024 | 0.001432723 | 0.997730543 |
| KRTAP22-1 | 1.394441743 | 4.277379905 | 5.411228412 | 0.000130921 | 0.001441554 | 0.990683123 |
| LCOR | 1.072795519 | 7.817532802 | 5.409321954 | 0.00013134 | 0.001445123 | 0.987401894 |
| DPY19L3 | 1.996625948 | 6.732077302 | 5.408293883 | 0.000131567 | 0.001446568 | 0.985632262 |
| RNF20 | -1.404649622 | 7.060788536 | -5.407056001 | 0.000131841 | 0.00144772 | 0.983501289 |
| TRAF3 | -1.809797303 | 5.85185909 | -5.406955675 | 0.000131863 | 0.00144772 | 0.983328572 |
| CCDC115 | -1.148686154 | 7.774444074 | -5.405789042 | 0.000132121 | 0.001448624 | 0.981320045 |
| GFRA3 | -2.194786498 | 8.134771942 | -5.405721119 | 0.000132136 | 0.001448624 | 0.981203101 |
| SCMH1 | 1.153951509 | 8.144501575 | 5.404927375 | 0.000132313 | 0.001449507 | 0.979836442 |
| LRIT2 | 1.8863955 | 3.163711292 | 5.404131664 | 0.000132489 | 0.001450396 | 0.978466311 |
| ZAP70 | -3.330789413 | 9.156123572 | -5.403151683 | 0.000132708 | 0.001451735 | 0.976778768 |
| DOCK7 | 1.493406771 | 6.458268177 | 5.402088025 | 0.000132945 | 0.001453281 | 0.974946982 |
| GNA15 | 2.585215193 | 10.47603955 | 5.401436596 | 0.00013309 | 0.001453823 | 0.973825045 |
| ARRB2 | -1.152878724 | 13.62351076 | -5.400735437 | 0.000133247 | 0.001454062 | 0.972617393 |
| PPARD | -1.270450277 | 6.611747966 | -5.400480887 | 0.000133304 | 0.001454062 | 0.97217895 |
| KDF1 | -1.884325999 | 3.862675732 | -5.399178681 | 0.000133596 | 0.001456197 | 0.969935853 |
| PRM1 | 1.142224759 | 5.891694783 | 5.39284317 | 0.000135026 | 0.001469666 | 0.95901942 |
| CRIP2 | -3.227485654 | 10.0736905 | -5.391594808 | 0.000135309 | 0.001471166 | 0.956867778 |
| IRGM | 2.382949912 | 6.175228221 | 5.391381929 | 0.000135358 | 0.001471166 | 0.956500847 |
| MRPL54 | -1.695826699 | 11.79588956 | -5.389934112 | 0.000135688 | 0.001473693 | 0.954005123 |
| ENOX2 | -1.161547321 | 7.551068077 | -5.383970298 | 0.000137055 | 0.001487479 | 0.943721798 |
| GOLGA8M | 3.246249623 | 3.654812354 | 5.381445862 | 0.000137638 | 0.001491687 | 0.939367492 |
| FOCAD | 1.614006003 | 9.838979098 | 5.381439608 | 0.00013764 | 0.001491687 | 0.939356704 |
| PCSK5 | -2.099381968 | 4.30292216 | -5.380412298 | 0.000137878 | 0.001493199 | 0.937584485 |
| KIF11 | 3.41216078 | 6.14491736 | 5.375962383 | 0.000138914 | 0.00150324 | 0.92990626 |
| SLC30A8 | 2.089237685 | 3.463386632 | 5.375580426 | 0.000139003 | 0.00150324 | 0.929247076 |
| EXOSC6 | 2.151504473 | 10.27747468 | 5.373884089 | 0.000139401 | 0.001506463 | 0.926319292 |
| RUNX2 | 2.28450325 | 8.211189866 | 5.373263126 | 0.000139547 | 0.001506963 | 0.925247445 |
| ZNF821 | -1.6679582 | 4.442402187 | -5.371996331 | 0.000139844 | 0.001509105 | 0.923060664 |
| RANBP10 | 1.418421738 | 10.05730932 | 5.370278228 | 0.00014025 | 0.001512399 | 0.920094474 |
| ADAM19 | -2.159708273 | 6.953038068 | -5.369446055 | 0.000140446 | 0.001513442 | 0.918657638 |
| TMEM19 | -1.463135551 | 8.424690939 | -5.366603834 | 0.00014112 | 0.001519266 | 0.913749533 |
| ZEB2 | 1.290159089 | 8.827245435 | 5.366321282 | 0.000141187 | 0.001519266 | 0.913261547 |
| ZNF526 | 2.356689186 | 14.40024124 | 5.36242985 | 0.000142116 | 0.001526962 | 0.906539683 |
| CXorf57 | -2.203158597 | 4.834252039 | -5.362023289 | 0.000142213 | 0.001526962 | 0.905837291 |
| HSPA1L | -2.341562141 | 5.713376136 | -5.361319699 | 0.000142382 | 0.001526962 | 0.904621687 |
| C2orf88 | -4.143911812 | 7.922142226 | -5.360852151 | 0.000142494 | 0.001526962 | 0.90381386 |
| MT-CO2 | 1.719081015 | 15.94982364 | 5.36079806 | 0.000142507 | 0.001526962 | 0.9037204 |
| RBM15 | 1.18937043 | 6.724641313 | 5.35805696 | 0.000143167 | 0.001531865 | 0.898983725 |
| ALG13 | 1.19811144 | 8.724210017 | 5.357581403 | 0.000143282 | 0.001532012 | 0.898161849 |
| MMP25 | -1.233541844 | 10.30596472 | -5.356538812 | 0.000143534 | 0.001533624 | 0.896359897 |
| RORA | -3.442884789 | 8.728514617 | -5.347869968 | 0.000145647 | 0.00155511 | 0.88137149 |
| SEC14L5 | -2.992145603 | 3.33120574 | -5.346816517 | 0.000145906 | 0.001556779 | 0.879549383 |
| RAP1A | -1.225200736 | 10.426625 | -5.340568927 | 0.000147453 | 0.001571683 | 0.868740129 |
| GFPT2 | -2.026512497 | 3.483024319 | -5.340335798 | 0.000147511 | 0.001571683 | 0.868336681 |
| POLR2J | -1.102389408 | 12.2603483 | -5.337901763 | 0.000148118 | 0.001576596 | 0.864123936 |
| ATP13A3 | 1.595781963 | 11.51739864 | 5.33765456 | 0.00014818 | 0.001576596 | 0.863696041 |
| SETBP1 | -2.240359356 | 6.297787631 | -5.335857182 | 0.00014863 | 0.001580278 | 0.860584628 |
| SLMAP | 1.400353778 | 6.422419987 | 5.335051242 | 0.000148833 | 0.00158132 | 0.859189338 |
| RNF24 | 1.784869311 | 8.91778229 | 5.334202971 | 0.000149046 | 0.001582477 | 0.857720664 |
| AATK | -1.837639552 | 5.453295018 | -5.33306224 | 0.000149333 | 0.001584334 | 0.855745483 |
| MAN2A2 | 1.763954209 | 8.376529059 | 5.33254492 | 0.000149464 | 0.001584334 | 0.854849682 |
| BLOC1S4 | -1.420037522 | 8.183030961 | -5.3322652 | 0.000149534 | 0.001584334 | 0.854365299 |
| SRSF2 | 1.87193901 | 8.117548483 | 5.328199346 | 0.000150565 | 0.001594138 | 0.847323386 |
| CLEC3B | -2.74353899 | 9.108090627 | -5.32390128 | 0.000151663 | 0.001604637 | 0.839876868 |
| PTCD1 | 1.154078606 | 9.842455406 | 5.323064678 | 0.000151877 | 0.001604997 | 0.838427142 |
| COMTD1 | -1.510740368 | 9.828256506 | -5.320093771 | 0.000152642 | 0.001610023 | 0.833278174 |
| NHS | -1.428454676 | 2.608679721 | -5.316517531 | 0.000153568 | 0.001617622 | 0.827078509 |
| FNDC3A | 1.619900221 | 8.039665503 | 5.316047049 | 0.00015369 | 0.001617622 | 0.826262769 |
| ZNF831 | -4.054584812 | 5.687222972 | -5.315839425 | 0.000153744 | 0.001617622 | 0.825902771 |
| PURB | 1.634559911 | 9.553152091 | 5.314471004 | 0.0001541 | 0.001620245 | 0.823529937 |
| REM2 | -2.719885222 | 7.605849866 | -5.31368723 | 0.000154305 | 0.00162127 | 0.822170764 |
| GLMP | -1.343709894 | 9.059665562 | -5.311290705 | 0.000154932 | 0.001626728 | 0.818014343 |
| ACACA | 1.547676539 | 8.427721567 | 5.309309759 | 0.000155452 | 0.001630257 | 0.8145781 |
| ALDH1A3 | 1.667449498 | 4.617812662 | 5.309191484 | 0.000155483 | 0.001630257 | 0.814372919 |
| TWF2 | -1.909576107 | 10.2774532 | -5.308641892 | 0.000155628 | 0.001630645 | 0.813419467 |
| TMEM158 | -4.231102789 | 5.342670427 | -5.307638763 | 0.000155892 | 0.001632287 | 0.811679103 |
| ARL4C | -3.067531181 | 11.11029207 | -5.307146696 | 0.000156022 | 0.001632519 | 0.810825347 |
| MTHFD1L | 1.22617274 | 6.619899343 | 5.303823594 | 0.000156902 | 0.001639757 | 0.805058788 |
| CHCHD7 | -1.149690367 | 9.716359505 | -5.303717953 | 0.00015693 | 0.001639757 | 0.804875444 |
| MTPN | -1.139836283 | 10.30954903 | -5.299712115 | 0.000157998 | 0.001649779 | 0.797922111 |
| SEC61A2 | 2.097208305 | 5.692370848 | 5.298475882 | 0.000158329 | 0.001652098 | 0.795775821 |
| PNN | 1.775786892 | 9.119049925 | 5.296643987 | 0.000158821 | 0.001655619 | 0.792594997 |
| ANKEF1 | -1.951548128 | 4.43910038 | -5.296406415 | 0.000158885 | 0.001655619 | 0.792182453 |
| SMIM11A | -1.426839911 | 8.724882513 | -5.295415362 | 0.000159152 | 0.001657261 | 0.790461411 |
| CALML4 | -1.248968162 | 6.762442998 | -5.290250574 | 0.000160551 | 0.00167068 | 0.781490218 |
| NT5E | -2.104920841 | 4.834877984 | -5.289370721 | 0.000160791 | 0.001671782 | 0.779961563 |
| PATL1 | -1.322801672 | 7.828349888 | -5.289051821 | 0.000160878 | 0.001671782 | 0.779407482 |
| LARP1 | 1.352034941 | 9.763549497 | 5.284028948 | 0.000162253 | 0.001681534 | 0.770678558 |
| AMIGO1 | -1.809549893 | 6.929926133 | -5.283932711 | 0.00016228 | 0.001681534 | 0.770511281 |
| TOP2B | 1.796306139 | 11.35855908 | 5.283252654 | 0.000162467 | 0.001681534 | 0.769329186 |
| OR2T27 | 1.778025285 | 6.320151219 | 5.283211567 | 0.000162479 | 0.001681534 | 0.769257764 |
| BASP1 | -4.206424852 | 8.720140594 | -5.282561478 | 0.000162658 | 0.001681534 | 0.768127699 |
| SPR | -2.126677844 | 5.18288474 | -5.282509832 | 0.000162672 | 0.001681534 | 0.76803792 |
| WBP1L | -1.662743604 | 8.537630463 | -5.282306174 | 0.000162728 | 0.001681534 | 0.767683883 |
| BAG2 | -2.207750143 | 7.227264217 | -5.281991816 | 0.000162815 | 0.001681534 | 0.767137396 |
| HKR1 | 1.412767412 | 6.693913626 | 5.279515253 | 0.0001635 | 0.001687462 | 0.76283162 |
| SPOCK2 | -1.954551772 | 10.93089683 | -5.279047995 | 0.00016363 | 0.00168765 | 0.762019149 |
| LRRC2 | 2.575693379 | 4.887695249 | 5.278289357 | 0.000163841 | 0.001688674 | 0.760699962 |
| ZNF641 | -1.390816281 | 6.495147444 | -5.277459012 | 0.000164072 | 0.001689763 | 0.759255997 |
| S1PR4 | -1.106331225 | 14.13772887 | -5.276198439 | 0.000164423 | 0.001691224 | 0.757063692 |
| TBC1D10B | 1.166136281 | 7.30216764 | 5.274502794 | 0.000164897 | 0.001694946 | 0.754114403 |
| EPM2AIP1 | 2.734482203 | 5.251541061 | 5.269696875 | 0.000166247 | 0.001707671 | 0.745753226 |
| USP14 | 1.526191008 | 6.357153466 | 5.265974466 | 0.000167301 | 0.001715455 | 0.739274984 |
| VN1R1 | 2.864299433 | 3.630069972 | 5.265851732 | 0.000167336 | 0.001715455 | 0.739061354 |
| HSDL2 | -1.283136947 | 7.160334993 | -5.265821553 | 0.000167345 | 0.001715455 | 0.739008825 |
| SLC8A1 | -1.136076646 | 4.918020999 | -5.264885478 | 0.000167611 | 0.001717023 | 0.737379429 |
| BEST1 | -2.067558972 | 5.766968168 | -5.263313882 | 0.000168059 | 0.001720449 | 0.73464354 |
| C2orf74 | -1.920971296 | 7.166811762 | -5.262019269 | 0.000168429 | 0.001723072 | 0.732389587 |
| HJURP | 3.631200375 | 8.359288678 | 5.259473949 | 0.000169159 | 0.001729372 | 0.727957468 |
| MT-CO1 | 1.680735973 | 16.40585785 | 5.256723668 | 0.000169951 | 0.001736126 | 0.723167484 |
| CD226 | -4.991030916 | 5.515894642 | -5.256386499 | 0.000170049 | 0.001736126 | 0.722580188 |
| ANAPC1 | 1.537869807 | 7.679240672 | 5.255435767 | 0.000170324 | 0.001737762 | 0.720924084 |
| BCL11B | -2.781198501 | 7.486691323 | -5.253724788 | 0.00017082 | 0.001741279 | 0.717943381 |
| PLA2G2C | 1.091764106 | 6.017702563 | 5.253454784 | 0.000170898 | 0.001741279 | 0.71747297 |
| FNIP1 | 2.055454289 | 7.625678684 | 5.251521385 | 0.000171461 | 0.0017441 | 0.71410425 |
| FBP1 | -1.658675796 | 9.40262285 | -5.250863437 | 0.000171653 | 0.0017441 | 0.712957741 |
| TMEM185A | -1.410144993 | 8.564727977 | -5.250615219 | 0.000171725 | 0.0017441 | 0.712525191 |
| INIP | -1.146016575 | 8.588395398 | -5.250343125 | 0.000171805 | 0.0017441 | 0.712051027 |
| CRAMP1 | 2.451625468 | 5.236878295 | 5.248950077 | 0.000172212 | 0.001746445 | 0.709623278 |
| SNAPC4 | 1.275046066 | 11.14424593 | 5.247411587 | 0.000172663 | 0.001749849 | 0.706941758 |
| TAF1L | 1.434359156 | 6.921628988 | 5.246980909 | 0.00017279 | 0.001749961 | 0.706191049 |
| FCRL2 | -2.410867444 | 6.304458612 | -5.246336672 | 0.000172979 | 0.001750709 | 0.705068043 |
| GPM6A | -1.09651184 | 4.579433718 | -5.245940075 | 0.000173096 | 0.001750721 | 0.704376685 |
| SNX10 | -3.098024933 | 8.965926773 | -5.244895818 | 0.000173404 | 0.001752206 | 0.702556206 |
| TMEM38A | -2.255046184 | 6.048619387 | -5.244657315 | 0.000173474 | 0.001752206 | 0.7021404 |
| SLC35C1 | -1.166999408 | 8.370847123 | -5.243733137 | 0.000173747 | 0.001753794 | 0.700529108 |
| CEP19 | -2.078298197 | 5.011672508 | -5.239155455 | 0.000175106 | 0.001765156 | 0.692546311 |
| MDN1 | 1.379558852 | 6.412373684 | 5.237543462 | 0.000175587 | 0.001768321 | 0.689734572 |
| CSNK1A1 | 1.846191715 | 10.6691781 | 5.237321779 | 0.000175653 | 0.001768321 | 0.689347871 |
| CHMP2A | -1.02481495 | 12.50725565 | -5.235132827 | 0.000176309 | 0.001772567 | 0.685529147 |
| LY86 | -1.791719083 | 12.70030495 | -5.234181225 | 0.000176595 | 0.001774263 | 0.683868838 |
| IGBP1 | -1.321300116 | 11.06439916 | -5.232338636 | 0.00017715 | 0.001777481 | 0.680653633 |
| ANKS1A | 1.453935782 | 9.049336819 | 5.22955949 | 0.00017799 | 0.001783552 | 0.675803343 |
| C4orf48 | -1.45271558 | 11.6193847 | -5.228506907 | 0.00017831 | 0.001785571 | 0.67396606 |
| PER2 | 1.341621859 | 7.527697418 | 5.227439373 | 0.000178634 | 0.00178764 | 0.672102531 |
| PRPF40A | 1.562659874 | 10.2805955 | 5.226487318 | 0.000178924 | 0.001788975 | 0.670440458 |
| COL16A1 | -1.212508656 | 7.998174027 | -5.22622606 | 0.000179004 | 0.001788975 | 0.66998434 |
| L1TD1 | -2.955117766 | 3.470433712 | -5.225756531 | 0.000179147 | 0.001789227 | 0.669164588 |
| PPP1R12B | 1.301115809 | 9.646328707 | 5.222546145 | 0.00018013 | 0.001797856 | 0.663558784 |
| TPP2 | 1.205857399 | 5.509290379 | 5.219027408 | 0.000181213 | 0.001807481 | 0.657412986 |
| MAML2 | -2.49804839 | 5.559535312 | -5.218512663 | 0.000181372 | 0.001807877 | 0.656513801 |
| STK16 | -1.555795856 | 8.398404367 | -5.218042424 | 0.000181518 | 0.001808095 | 0.655692328 |
| APH1A | -1.564794989 | 8.079229062 | -5.217537529 | 0.000181674 | 0.001808095 | 0.654810282 |
| SSU72P7 | 1.061467265 | 3.221728666 | 5.217124249 | 0.000181802 | 0.001808095 | 0.654088262 |
| KRTAP6-1 | 1.066231929 | 6.488703905 | 5.216900345 | 0.000181872 | 0.001808095 | 0.653697082 |
| LFNG | -1.687543886 | 9.29002385 | -5.216416542 | 0.000182022 | 0.001808401 | 0.652851811 |
| PHLDA2 | -3.614695915 | 6.42210958 | -5.210502587 | 0.000183867 | 0.001825535 | 0.64251681 |
| TLR4 | -3.46412521 | 6.903606143 | -5.210107366 | 0.000183991 | 0.00182557 | 0.641825973 |
| ARHGEF3 | -1.7992277 | 8.475829524 | -5.209414611 | 0.000184208 | 0.001826185 | 0.640615001 |
| ANKDD1B | 1.019152971 | 4.106931702 | 5.207873422 | 0.000184693 | 0.001827189 | 0.637920699 |
| ZSCAN22 | 1.668368367 | 5.616449938 | 5.207786071 | 0.000184721 | 0.001827189 | 0.637767983 |
| AK4 | 1.814009051 | 9.047211752 | 5.207119271 | 0.000184931 | 0.001827189 | 0.636602178 |
| DEXI | 1.504837657 | 6.003902109 | 5.206907236 | 0.000184998 | 0.001827189 | 0.636231454 |
| NOTCH4 | -1.973913302 | 7.45907321 | -5.205907829 | 0.000185314 | 0.001828829 | 0.634483997 |
| ILDR1 | 2.860716528 | 13.30534213 | 5.205617748 | 0.000185406 | 0.001828829 | 0.633976767 |
| CBFA2T2 | 1.201298365 | 7.048861427 | 5.203053109 | 0.000186219 | 0.001833271 | 0.62949181 |
| TONSL | 1.333709576 | 7.386548267 | 5.201967803 | 0.000186564 | 0.001833673 | 0.627593603 |
| PHF5A | -1.406256169 | 9.482950058 | -5.201783854 | 0.000186623 | 0.001833673 | 0.627271859 |
| TRIP12 | 1.239122512 | 8.677728073 | 5.199214665 | 0.000187443 | 0.00184054 | 0.622777644 |
| SCAF11 | 1.233333991 | 9.63131067 | 5.196426735 | 0.000188338 | 0.001847153 | 0.617899806 |
| TMBIM4 | -1.0372845 | 11.37296965 | -5.196355857 | 0.000188361 | 0.001847153 | 0.617775784 |
| TIE1 | 4.453096583 | 4.503369487 | 5.195881599 | 0.000188513 | 0.001847453 | 0.616945903 |
| ASPHD2 | -1.315530813 | 7.054325612 | -5.195404851 | 0.000188667 | 0.001847763 | 0.616111635 |
| THADA | 1.24983838 | 7.265649025 | 5.192824215 | 0.0001895 | 0.001854727 | 0.611595224 |
| CREBZF | 1.298701173 | 7.505265069 | 5.191348539 | 0.000189979 | 0.001858209 | 0.609012226 |
| CEACAM8 | -4.088952323 | 7.008815558 | -5.190550636 | 0.000190238 | 0.001859133 | 0.607615472 |
| ZSCAN31 | 1.996766719 | 4.68401465 | 5.190302278 | 0.000190318 | 0.001859133 | 0.607180697 |
| ZMYM4 | 1.19958497 | 8.815805005 | 5.185841574 | 0.000191775 | 0.001870947 | 0.5993704 |
| AWAT2 | 1.776157449 | 3.180579425 | 5.182659695 | 0.000192821 | 0.001879941 | 0.593797612 |
| TCL1A | -2.363279084 | 9.190470426 | -5.178294404 | 0.000194265 | 0.001890291 | 0.586150016 |
| SYNM | -1.723835654 | 3.045554558 | -5.178157612 | 0.000194311 | 0.001890291 | 0.585910328 |
| MTMR4 | 1.169753478 | 9.99806525 | 5.177945526 | 0.000194381 | 0.001890291 | 0.585538705 |
| UNK | 1.257837154 | 9.243740761 | 5.17711345 | 0.000194658 | 0.001891298 | 0.584080661 |
| STX10 | -1.078235048 | 10.46597057 | -5.176884075 | 0.000194735 | 0.001891298 | 0.583678711 |
| UBE2D2 | 1.972773498 | 11.7656457 | 5.175592418 | 0.000195165 | 0.001893475 | 0.581415126 |
| CFAP97 | -1.19038075 | 8.257239127 | -5.175462677 | 0.000195209 | 0.001893475 | 0.581187746 |
| CD68 | -1.911557448 | 8.268880845 | -5.174938384 | 0.000195384 | 0.001893961 | 0.580268868 |
| CPNE5 | -2.63552149 | 11.01970443 | -5.17167726 | 0.000196477 | 0.001903341 | 0.574552602 |
| APPL1 | 1.060495988 | 10.77892341 | 5.170655817 | 0.000196821 | 0.001905452 | 0.572761876 |
| NEU2 | 1.508797825 | 2.641319758 | 5.169928496 | 0.000197066 | 0.001906607 | 0.571486703 |
| MCM6 | 1.604139472 | 10.05482148 | 5.168349307 | 0.000197599 | 0.001910547 | 0.568717756 |
| C17orf98 | -1.404693476 | 3.693020671 | -5.166938925 | 0.000198077 | 0.001913944 | 0.566244518 |
| POGZ | 1.642249125 | 9.30382977 | 5.166208353 | 0.000198325 | 0.001915118 | 0.564963288 |
| SLC35E2A | 1.537730225 | 8.716214433 | 5.165558632 | 0.000198546 | 0.001916028 | 0.56382379 |
| ZBTB25 | -1.294665055 | 8.13531271 | -5.163567654 | 0.000199224 | 0.001920125 | 0.560331619 |
| FAM49A | -2.438854499 | 9.264074894 | -5.161922477 | 0.000199786 | 0.001923888 | 0.557445589 |
| LSM11 | 2.519322726 | 3.591402738 | 5.161681705 | 0.000199868 | 0.001923888 | 0.557023188 |
| MPPE1 | -1.235259612 | 8.282008541 | -5.161036032 | 0.000200089 | 0.001924794 | 0.555890407 |
| PRUNE1 | -1.911703119 | 5.227543266 | -5.156319675 | 0.000201712 | 0.001938898 | 0.547614289 |
| PABPC1 | 1.077517833 | 15.55922409 | 5.156033161 | 0.000201811 | 0.001938898 | 0.547111431 |
| EVI2B | -1.201545527 | 10.91107491 | -5.154444841 | 0.000202361 | 0.00194295 | 0.544323582 |
| PLEKHA1 | -2.813205323 | 8.244738812 | -5.153847314 | 0.000202569 | 0.001943708 | 0.543274705 |
| UGGT1 | 1.477432786 | 7.722151057 | 5.153302589 | 0.000202758 | 0.001944292 | 0.542318475 |
| ACTN3 | -1.672243961 | 4.292407903 | -5.151842967 | 0.000203266 | 0.001947929 | 0.53975601 |
| MYCBP2 | 1.309290297 | 11.7402912 | 5.151277952 | 0.000203462 | 0.001948584 | 0.538764012 |
| SMAD4 | 1.077715 | 10.05344169 | 5.150460418 | 0.000203748 | 0.001949981 | 0.537328595 |
| ROR2 | 1.661307042 | 11.48881665 | 5.150015188 | 0.000203903 | 0.001949981 | 0.536546828 |
| SLC2A9 | -1.777421458 | 7.019379456 | -5.149377021 | 0.000204127 | 0.001950013 | 0.535426243 |
| ASIC2 | 1.106903753 | 6.325888708 | 5.143013698 | 0.000206366 | 0.001970164 | 0.524249698 |
| KIAA0040 | -1.979836184 | 6.429014284 | -5.142072434 | 0.000206699 | 0.001970984 | 0.522596013 |
| SLC10A1 | 1.702251242 | 6.729180635 | 5.141941823 | 0.000206746 | 0.001970984 | 0.522366536 |
| GID8 | 1.054217006 | 10.85470768 | 5.141670072 | 0.000206842 | 0.001970984 | 0.521889078 |
| SGPP2 | -1.257559411 | 2.920833863 | -5.141270605 | 0.000206984 | 0.001971095 | 0.521187206 |
| SGO2 | 1.511466717 | 4.072028276 | 5.138638909 | 0.00020792 | 0.001978771 | 0.516562751 |
| KCNN4 | 1.441950178 | 10.05455148 | 5.137264702 | 0.000208411 | 0.001981539 | 0.514147617 |
| C18orf54 | 2.997779147 | 3.67981496 | 5.13709249 | 0.000208473 | 0.001981539 | 0.513844941 |
| GPR155 | -2.799814141 | 6.405386332 | -5.136167276 | 0.000208804 | 0.001981837 | 0.512218741 |
| DYRK2 | -1.298549587 | 8.306949145 | -5.135911243 | 0.000208896 | 0.001981837 | 0.511768704 |
| NCALD | -2.116432006 | 7.45834574 | -5.135909081 | 0.000208897 | 0.001981837 | 0.511764904 |
| ANXA2 | -3.566663689 | 12.30290476 | -5.135539689 | 0.000209029 | 0.001981853 | 0.511115599 |
| COX4I1 | -1.041392244 | 14.43903152 | -5.134665784 | 0.000209343 | 0.001982916 | 0.509579408 |
| ATP5F1D | -1.06893248 | 11.33855453 | -5.133004461 | 0.000209941 | 0.001986765 | 0.506658782 |
| GPR75-ASB3 | 1.273371238 | 8.838843632 | 5.132470448 | 0.000210133 | 0.001987346 | 0.505719905 |
| SLC44A2 | -1.779596535 | 9.679602863 | -5.131171545 | 0.000210602 | 0.001990396 | 0.503436077 |
| CLCF1 | -1.638311836 | 6.777812504 | -5.13056176 | 0.000210823 | 0.001990396 | 0.502363832 |
| SPPL2A | -1.459006591 | 9.238711647 | -5.130487423 | 0.00021085 | 0.001990396 | 0.502233116 |
| ZNF581 | -1.510149134 | 8.946466926 | -5.129123715 | 0.000211344 | 0.001993819 | 0.499834984 |
| LRRC41 | 3.328871793 | 7.172687453 | 5.128301683 | 0.000211643 | 0.001995393 | 0.498389294 |
| MAPK8IP3 | 1.60693981 | 12.87141541 | 5.122803312 | 0.000213651 | 0.002013073 | 0.488717169 |
| CYTIP | -1.686633125 | 12.22556422 | -5.121410846 | 0.000214162 | 0.002016116 | 0.486267073 |
| TBC1D1 | -1.009116345 | 9.448508072 | -5.121195701 | 0.000214242 | 0.002016116 | 0.485888495 |
| ZNF524 | -1.252031469 | 10.62930544 | -5.120528846 | 0.000214487 | 0.002016116 | 0.48471503 |
| KCTD11 | -1.302779503 | 5.724863212 | -5.117601922 | 0.000215569 | 0.002023594 | 0.479563842 |
| PLEKHO2 | -1.622795791 | 10.25992096 | -5.114419045 | 0.000216751 | 0.002033435 | 0.473960933 |
| TBX21 | -3.610909971 | 8.513041688 | -5.113654402 | 0.000217036 | 0.00203485 | 0.472614715 |
| C9orf66 | -1.669705606 | 3.715279261 | -5.113285181 | 0.000217174 | 0.002034883 | 0.471964643 |
| SNAI3 | -1.976432784 | 8.016918157 | -5.112257413 | 0.000217558 | 0.002037222 | 0.470155002 |
| INHBA | 2.078299092 | 4.743692747 | 5.109131278 | 0.000218731 | 0.002046645 | 0.464649823 |
| ZNF516 | 1.636846467 | 7.432309554 | 5.108855172 | 0.000218835 | 0.002046645 | 0.464163533 |
| IQSEC2 | -1.947480312 | 9.223846879 | -5.10695844 | 0.00021955 | 0.002052066 | 0.460822671 |
| KIF18A | 2.996124599 | 4.23259798 | 5.10644721 | 0.000219743 | 0.002052605 | 0.459922122 |
| FBXO22 | 1.440903984 | 5.6937027 | 5.103822586 | 0.000220737 | 0.002060624 | 0.455298223 |
| CIAO2A | -1.571856755 | 11.67872424 | -5.103128638 | 0.000221001 | 0.002061815 | 0.454075519 |
| SLC10A2 | 2.492569925 | 4.903405283 | 5.101331193 | 0.000221686 | 0.002066929 | 0.450908218 |
| TMEM71 | -1.363087168 | 9.147758626 | -5.099593835 | 0.000222349 | 0.002071543 | 0.447846398 |
| PXDC1 | -2.033706199 | 3.815402779 | -5.099320751 | 0.000222454 | 0.002071543 | 0.447365096 |
| VWA2 | -1.125854914 | 5.564936083 | -5.093947619 | 0.000224521 | 0.002089383 | 0.437893141 |
| BIN2 | -2.649154198 | 9.894250881 | -5.093519237 | 0.000224687 | 0.002089383 | 0.437137815 |
| BPIFB3 | 1.533029074 | 3.038267011 | 5.093269612 | 0.000224783 | 0.002089383 | 0.436697662 |
| ISY1-RAB43 | -2.011606596 | 7.41597303 | -5.090277439 | 0.000225945 | 0.002098889 | 0.431421073 |
| ZADH2 | 1.889604095 | 6.956768142 | 5.089473763 | 0.000226258 | 0.002100509 | 0.430003622 |
| ANKRD17 | 1.228678071 | 7.139241975 | 5.088962785 | 0.000226457 | 0.002101071 | 0.429102362 |
| USP24 | 1.515934675 | 7.775389692 | 5.087512725 | 0.000227023 | 0.002105037 | 0.426544572 |
| KIAA0895L | 1.723087016 | 7.17284277 | 5.083519689 | 0.000228591 | 0.002116978 | 0.419499775 |
| BTBD16 | 1.511308078 | 4.826119812 | 5.081892741 | 0.000229232 | 0.002121625 | 0.416628809 |
| ULK1 | 1.041229663 | 10.03361902 | 5.08058434 | 0.00022975 | 0.002125117 | 0.414319715 |
| FOXQ1 | 1.602526068 | 2.664647565 | 5.078974294 | 0.000230388 | 0.002129722 | 0.411477968 |
| LCP1 | -1.57554514 | 14.36094393 | -5.077306832 | 0.000231052 | 0.002134551 | 0.40853453 |
| CCNE2 | 1.74037957 | 6.174784777 | 5.076869262 | 0.000231226 | 0.00213486 | 0.407762063 |
| MAPK13 | -1.393677036 | 7.056881463 | -5.072134004 | 0.000233122 | 0.002149745 | 0.399401074 |
| NPL | -3.119647235 | 6.620719719 | -5.070580174 | 0.000233748 | 0.002154203 | 0.396656867 |
| MYL12B | -1.074021553 | 14.21344926 | -5.069634308 | 0.000234129 | 0.002155922 | 0.394986229 |
| SERINC5 | 1.184043948 | 9.067079717 | 5.069412934 | 0.000234219 | 0.002155922 | 0.394595211 |
| CERS1 | 2.180052992 | 4.610215943 | 5.068447699 | 0.000234609 | 0.002158204 | 0.392890214 |
| URB1 | 1.023129866 | 4.834708543 | 5.067828167 | 0.00023486 | 0.002158781 | 0.391795808 |
| LGALSL | -3.106859133 | 7.871864397 | -5.067589416 | 0.000234957 | 0.002158781 | 0.39137404 |
| KRCC1 | -1.828948717 | 8.830439044 | -5.062920503 | 0.000236858 | 0.002174599 | 0.383124652 |
| C6orf62 | 1.312824266 | 12.57098287 | 5.06265679 | 0.000236965 | 0.002174599 | 0.382658622 |
| TBC1D9 | -1.670193735 | 6.912309125 | -5.062055794 | 0.000237211 | 0.002175538 | 0.381596513 |
| IFNL3 | -2.121686546 | 4.006411015 | -5.06157682 | 0.000237408 | 0.00217602 | 0.380750015 |
| CBX5 | 1.692969904 | 9.393771028 | 5.060790455 | 0.00023773 | 0.002177659 | 0.379360196 |
| TAGLN2 | -1.427099851 | 13.26305732 | -5.059900946 | 0.000238095 | 0.002179688 | 0.377787985 |
| ITPKB | -1.561899127 | 9.169430035 | -5.057676654 | 0.000239012 | 0.002186753 | 0.373856095 |
| ZNF114 | 2.083551184 | 2.536601092 | 5.056567056 | 0.00023947 | 0.00218945 | 0.371894418 |
| B3GNT5 | 1.236514141 | 7.897264186 | 5.056097343 | 0.000239664 | 0.00218945 | 0.371063956 |
| BICDL1 | -1.536013373 | 4.346196629 | -5.055914923 | 0.00023974 | 0.00218945 | 0.370741427 |
| OR2AG1 | 1.231065241 | 3.828441614 | 5.052831156 | 0.00024102 | 0.002199817 | 0.365288497 |
| SENP5 | 1.187932819 | 9.919124981 | 5.051245067 | 0.000241681 | 0.002204525 | 0.362483391 |
| SGO1 | 2.205418024 | 5.191266942 | 5.04893994 | 0.000242646 | 0.00221199 | 0.358406044 |
| CAPNS2 | -1.483077073 | 7.742638916 | -5.048153615 | 0.000242976 | 0.002212401 | 0.357015025 |
| GNPTAB | 1.74640607 | 9.882593601 | 5.047477823 | 0.00024326 | 0.002213591 | 0.355819476 |
| USP49 | 1.101701629 | 5.00036736 | 5.045197437 | 0.00024422 | 0.002220998 | 0.3517848 |
| GLIPR1 | -1.308908486 | 8.921912576 | -5.044399557 | 0.000244557 | 0.002222729 | 0.350372956 |
| SLC43A1 | 1.355941147 | 8.472608213 | 5.043611827 | 0.000244891 | 0.002224423 | 0.348978995 |
| GAL3ST4 | 1.211661285 | 5.989708945 | 5.042546443 | 0.000245342 | 0.002227189 | 0.347093572 |
| FUCA1 | -1.362728194 | 8.498151995 | -5.037553235 | 0.00024747 | 0.002245157 | 0.338255104 |
| FZD5 | 2.786290521 | 4.575723008 | 5.03607949 | 0.000248101 | 0.002248448 | 0.335645824 |
| CD1B | -1.960359723 | 4.956741848 | -5.036014301 | 0.000248129 | 0.002248448 | 0.3355304 |
| DHRS12 | -1.489881167 | 7.695824662 | -5.034851286 | 0.000248629 | 0.002251629 | 0.333471065 |
| TANGO6 | -1.488787673 | 8.170694224 | -5.033232936 | 0.000249326 | 0.002255778 | 0.330605189 |
| TTC37 | 1.144568731 | 9.843900506 | 5.033096374 | 0.000249385 | 0.002255778 | 0.330363342 |
| OXR1 | -1.088795342 | 5.618496273 | -5.022040259 | 0.000254203 | 0.002296619 | 0.310775374 |
| SYDE2 | -1.167761536 | 2.526507394 | -5.021431054 | 0.000254471 | 0.002297455 | 0.3096956 |
| IL7R | -2.848545216 | 11.23387661 | -5.021141862 | 0.000254599 | 0.002297455 | 0.309183013 |
| PCNX1 | 1.40546395 | 10.67400268 | 5.016214627 | 0.000256781 | 0.002315767 | 0.300447937 |
| PABPC3 | 1.054664775 | 12.72523037 | 5.015456441 | 0.000257118 | 0.002317071 | 0.29910354 |
| KIAA0513 | -1.610060186 | 10.15698659 | -5.014767485 | 0.000257426 | 0.002317071 | 0.297881838 |
| DDX60 | -1.111005109 | 7.448349574 | -5.01451706 | 0.000257537 | 0.002317071 | 0.297437752 |
| ECHDC1 | -1.611507587 | 9.385884314 | -5.010930256 | 0.000259143 | 0.002330133 | 0.291076306 |
| TDP2 | -1.263735009 | 9.957809838 | -5.009741082 | 0.000259678 | 0.002333556 | 0.288966864 |
| ZNF620 | 1.415420571 | 6.604826329 | 5.009228938 | 0.000259908 | 0.002334244 | 0.28805833 |
| MCM10 | 2.269819514 | 4.662125597 | 5.007595559 | 0.000260645 | 0.002339475 | 0.285160528 |
| RBM12B | 1.571902877 | 6.536059467 | 5.006456025 | 0.000261161 | 0.002342714 | 0.283138664 |
| BTNL8 | -5.111561684 | 4.404737867 | -5.004743438 | 0.000261937 | 0.002347843 | 0.280099729 |
| CREB3L2 | 1.174763133 | 9.457423577 | 5.004266708 | 0.000262154 | 0.002347843 | 0.27925372 |
| NDUFA6 | -1.334669942 | 12.26456807 | -5.002745977 | 0.000262846 | 0.002352262 | 0.276554825 |
| TMEM91 | -1.310969869 | 10.02047876 | -4.999935117 | 0.000264131 | 0.002362362 | 0.271565525 |
| ZNF579 | 1.049738566 | 8.067751973 | 4.998276214 | 0.000264892 | 0.002367772 | 0.268620491 |
| C4orf33 | -1.33431607 | 8.583223971 | -4.996546886 | 0.000265688 | 0.002373487 | 0.265550063 |
| NUP43 | 2.07669184 | 9.213644077 | 4.995691364 | 0.000266082 | 0.002375612 | 0.264030939 |
| GOLM1 | -2.593528385 | 6.436510966 | -4.994498094 | 0.000266634 | 0.002379134 | 0.261911934 |
| PDCD10 | -1.300498085 | 10.08889521 | -4.993643496 | 0.00026703 | 0.002379927 | 0.260394233 |
| PROM1 | 4.796334275 | 4.445158439 | 4.993627683 | 0.000267037 | 0.002379927 | 0.260366149 |
| STX8 | -1.348083484 | 10.84670026 | -4.992467817 | 0.000267575 | 0.00238324 | 0.258306164 |
| IFIT5 | -1.432162609 | 9.035493391 | -4.992148758 | 0.000267723 | 0.00238324 | 0.257739469 |
| RAD18 | 1.096190913 | 6.289103317 | 4.991259204 | 0.000268137 | 0.002385521 | 0.256159421 |
| RAB3A | -2.23911401 | 5.967817797 | -4.988604815 | 0.000269375 | 0.002395132 | 0.251444042 |
| PSENEN | -1.02979717 | 10.98239093 | -4.988018052 | 0.00026965 | 0.002396167 | 0.25040157 |
| ECHDC3 | -1.213319926 | 7.096124268 | -4.985846359 | 0.000270668 | 0.002403809 | 0.246542854 |
| CD28 | -2.169485694 | 7.719765325 | -4.984114364 | 0.000271484 | 0.002409638 | 0.243464979 |
| ARHGAP18 | -1.770992033 | 7.140713118 | -4.981378733 | 0.000272777 | 0.002419697 | 0.238602806 |
| NKX6-1 | -1.634132554 | 2.940996079 | -4.97559707 | 0.000275531 | 0.002442696 | 0.228323677 |
| UHRF1 | 3.003966554 | 7.922815054 | 4.974654661 | 0.000275982 | 0.002445069 | 0.226647785 |
| WDR31 | 1.511712715 | 6.898770336 | 4.974365362 | 0.000276121 | 0.002445069 | 0.2261333 |
| DNAJB5 | -2.029191741 | 5.49919658 | -4.972876888 | 0.000276837 | 0.002449865 | 0.223486058 |
| KDM4A | 1.300287215 | 7.70248942 | 4.972565751 | 0.000276986 | 0.002449865 | 0.222932667 |
| NAP1L2 | -2.710300353 | 4.00726176 | -4.967797559 | 0.000279292 | 0.00246598 | 0.21445041 |
| TIAF1 | 1.631440919 | 9.163855501 | 4.967788329 | 0.000279297 | 0.00246598 | 0.214433987 |
| MRE11 | 1.964372847 | 8.68503419 | 4.96547041 | 0.000280425 | 0.002474197 | 0.210309552 |
| ZNF260 | 1.105304692 | 4.575632449 | 4.965134425 | 0.000280589 | 0.002474197 | 0.209711654 |
| STX7 | -1.148059244 | 8.288346818 | -4.964871016 | 0.000280717 | 0.002474197 | 0.209242899 |
| PLTP | 1.967412733 | 8.510502628 | 4.964507882 | 0.000280894 | 0.002474321 | 0.20859666 |
| NMUR1 | -2.528697951 | 8.176520071 | -4.962316958 | 0.000281967 | 0.002482326 | 0.204697316 |
| GOLGA8K | 1.851829996 | 5.901438609 | 4.958934558 | 0.000283631 | 0.00249125 | 0.198676238 |
| KLHDC1 | 1.717542917 | 5.550511807 | 4.958495315 | 0.000283848 | 0.00249125 | 0.197894227 |
| REV3L | 1.517296321 | 8.656881344 | 4.958312375 | 0.000283939 | 0.00249125 | 0.197568523 |
| GRAMD4 | 2.485334953 | 9.970807217 | 4.958254066 | 0.000283967 | 0.00249125 | 0.197464709 |
| TRPM5 | 1.614459343 | 7.822825146 | 4.95745585 | 0.000284362 | 0.002493269 | 0.196043511 |
| HMGB3 | 1.954159675 | 6.860165109 | 4.956927913 | 0.000284623 | 0.002494118 | 0.195103493 |
| FOXM1 | 2.311718575 | 6.874830488 | 4.952784205 | 0.000286684 | 0.00251072 | 0.187724214 |
| TSSK2 | 1.740468994 | 2.821185697 | 4.951552591 | 0.000287299 | 0.002514656 | 0.185530494 |
| VEGFC | -2.283122784 | 3.984846445 | -4.949993078 | 0.00028808 | 0.002519998 | 0.182752458 |
| SRMS | -1.749355016 | 8.223918181 | -4.949670703 | 0.000288242 | 0.002519998 | 0.182178158 |
| LYAR | -2.461261247 | 10.01140547 | -4.942119432 | 0.000292058 | 0.00255189 | 0.168722129 |
| NUP133 | 1.233468444 | 8.271340475 | 4.940345102 | 0.000292963 | 0.002558316 | 0.165559325 |
| E2F8 | 3.477286242 | 3.645575357 | 4.937995088 | 0.000294165 | 0.002566074 | 0.161369742 |
| SAMD12 | -2.160262247 | 3.502597663 | -4.937868466 | 0.00029423 | 0.002566074 | 0.161143981 |
| CDCA7L | 1.037436268 | 8.444398851 | 4.937616382 | 0.000294359 | 0.002566074 | 0.160694524 |
| POLR1E | -1.234060574 | 7.375334945 | -4.936606152 | 0.000294878 | 0.002569119 | 0.158893237 |
| CD34 | 4.813899033 | 6.091221552 | 4.935983431 | 0.000295198 | 0.002570431 | 0.157782833 |
| NEFL | -1.630212292 | 4.33499929 | -4.93277562 | 0.000296854 | 0.002583361 | 0.152062071 |
| TAS2R30 | 2.468008966 | 7.572590519 | 4.930728178 | 0.000297916 | 0.002589236 | 0.148410028 |
| PRPF18 | -1.215750537 | 9.73331352 | -4.930485575 | 0.000298042 | 0.002589236 | 0.147977261 |
| AC037459.1 | -1.591179602 | 8.590668263 | -4.929287778 | 0.000298665 | 0.002591679 | 0.145840461 |
| MIF4GD | -1.472119209 | 8.546233484 | -4.928523039 | 0.000299063 | 0.002593652 | 0.144476119 |
| SS18L1 | 1.646932636 | 5.912533309 | 4.925935765 | 0.000300416 | 0.002603893 | 0.139859724 |
| PGPEP1L | 1.105761675 | 5.053723799 | 4.925502492 | 0.000300643 | 0.002604372 | 0.139086567 |
| VAT1 | 1.272497546 | 7.414874843 | 4.924524972 | 0.000301156 | 0.002606163 | 0.137342139 |
| PQLC3 | -1.805411564 | 10.35564471 | -4.920681192 | 0.000303183 | 0.002621864 | 0.130481601 |
| OMP | -1.110437712 | 6.262055907 | -4.918608537 | 0.000304282 | 0.002628378 | 0.126781482 |
| LRCH4 | 1.495173721 | 4.425770658 | 4.917648762 | 0.000304792 | 0.002630425 | 0.125067906 |
| RPL7L1 | 1.28323807 | 6.513970777 | 4.917509804 | 0.000304866 | 0.002630425 | 0.124819802 |
| C15orf53 | -1.580837945 | 2.369429454 | -4.916308013 | 0.000305506 | 0.002634449 | 0.12267395 |
| PCYT1B | -2.333511928 | 4.149584578 | -4.915053194 | 0.000306176 | 0.002637225 | 0.120433225 |
| DCP1A | 1.163207836 | 9.3581199 | 4.912184588 | 0.000307714 | 0.002646481 | 0.11531004 |
| TRAPPC3L | 1.746283667 | 7.004483863 | 4.911826799 | 0.000307906 | 0.002646481 | 0.114670975 |
| GOLGA3 | 1.064744445 | 7.150433483 | 4.911570585 | 0.000308044 | 0.002646481 | 0.11421333 |
| RASSF4 | -1.672886723 | 8.188007216 | -4.911207637 | 0.000308239 | 0.002646481 | 0.113565022 |
| PROS1 | -4.102141882 | 5.195194967 | -4.911096716 | 0.000308299 | 0.002646481 | 0.113366889 |
| GAREM2 | -2.105345622 | 4.071757214 | -4.908104279 | 0.000309915 | 0.002656122 | 0.108021068 |
| TRIM50 | 1.502034013 | 4.015880828 | 4.906992016 | 0.000310517 | 0.002659497 | 0.106033792 |
| PGA5 | -1.137987165 | 5.412301049 | -4.905853613 | 0.000311136 | 0.002661964 | 0.103999653 |
| ACOT4 | -1.325939137 | 6.200265428 | -4.905814488 | 0.000311157 | 0.002661964 | 0.10392974 |
| ZFYVE1 | 1.026192591 | 8.601355413 | 4.905196518 | 0.000311493 | 0.002663336 | 0.10282546 |
| TMEM8A | -1.227804485 | 9.97214782 | -4.904259541 | 0.000312003 | 0.002666196 | 0.101151041 |
| ZNF670 | 1.251957972 | 8.414825879 | 4.89969147 | 0.000314504 | 0.002686039 | 0.092986157 |
| KRTAP10-5 | 1.22103441 | 6.588509697 | 4.89908813 | 0.000314836 | 0.002686039 | 0.091907566 |
| CBLB | -1.055551563 | 5.270973604 | -4.898891202 | 0.000314945 | 0.002686039 | 0.091555509 |
| C8orf59 | -1.069015345 | 11.60020416 | -4.898727659 | 0.000315035 | 0.002686039 | 0.091263132 |
| TMPRSS9 | 1.949900367 | 5.880935616 | 4.898059378 | 0.000315403 | 0.002687666 | 0.090068366 |
| SPAG11A | 1.639318882 | 2.325924246 | 4.89714343 | 0.000315908 | 0.00269046 | 0.088430725 |
| SCP2 | -1.31423432 | 10.03545017 | -4.895789994 | 0.000316657 | 0.002695319 | 0.086010705 |
| ERC1 | 1.230888287 | 6.552698678 | 4.894644353 | 0.000317292 | 0.002699053 | 0.083962059 |
| ZNF165 | 1.668222147 | 7.619171327 | 4.894356209 | 0.000317452 | 0.002699053 | 0.083446771 |
| UBR2 | 1.891829185 | 6.516112907 | 4.891150422 | 0.000319237 | 0.002712705 | 0.077713185 |
| SLC9B2 | 1.177924324 | 5.218613455 | 4.890154086 | 0.000319793 | 0.002715058 | 0.07593097 |
| PACSIN3 | 1.478462277 | 4.459932252 | 4.890013928 | 0.000319872 | 0.002715058 | 0.07568025 |
| OTULINL | -3.413085019 | 8.200088205 | -4.886874072 | 0.000321634 | 0.002726378 | 0.070062926 |
| GRB10 | 3.258557759 | 7.429925874 | 4.886491001 | 0.000321849 | 0.002726378 | 0.069377515 |
| KCNH7 | 1.263696635 | 2.284010747 | 4.886145 | 0.000322044 | 0.002726378 | 0.068758416 |
| PRKCA | -3.084247441 | 7.600322198 | -4.886037015 | 0.000322105 | 0.002726378 | 0.068565195 |
| MYH14 | 1.394882224 | 8.702967495 | 4.885339861 | 0.000322498 | 0.002728182 | 0.067317727 |
| PGM1 | -2.073079385 | 9.563234307 | -4.882807069 | 0.000323931 | 0.002738772 | 0.062785112 |
| GPR182 | 1.582184447 | 4.344834977 | 4.881911526 | 0.000324439 | 0.002740562 | 0.061182285 |
| SEH1L | 1.218987114 | 7.264194651 | 4.881796567 | 0.000324505 | 0.002740562 | 0.060976526 |
| FYB1 | -1.942100485 | 8.544422725 | -4.879793335 | 0.000325644 | 0.002748657 | 0.057390797 |
| OR5T2 | 1.873644569 | 2.777705125 | 4.87778814 | 0.00032679 | 0.002756788 | 0.053801064 |
| ARHGAP26 | -1.346228439 | 7.317159745 | -4.873929588 | 0.000329005 | 0.002772391 | 0.046892043 |
| DRGX | 1.124709161 | 6.917542933 | 4.872878017 | 0.000329612 | 0.002775958 | 0.045008813 |
| CST3 | -1.43599005 | 10.58850458 | -4.871994356 | 0.000330122 | 0.002777871 | 0.043426184 |
| GINS1 | 2.93068125 | 7.134233219 | 4.871850373 | 0.000330206 | 0.002777871 | 0.043168303 |
| NDUFA5 | -1.225309535 | 11.44385581 | -4.870318529 | 0.000331093 | 0.00278379 | 0.040424535 |
| NUP153 | 1.356373513 | 9.509824586 | 4.869340131 | 0.000331661 | 0.002785631 | 0.038671924 |
| CARD11 | -2.249895957 | 7.790369121 | -4.869308028 | 0.00033168 | 0.002785631 | 0.038614414 |
| MSS51 | 1.880187555 | 2.813410607 | 4.867858438 | 0.000332523 | 0.002791168 | 0.036017535 |
| PABPC1L2B | 1.015252502 | 5.839424648 | 4.867203528 | 0.000332905 | 0.002792825 | 0.034844207 |
| BNC2 | -1.481047344 | 4.260582586 | -4.864649053 | 0.000334399 | 0.002802253 | 0.030267149 |
| MEGF8 | 1.481499502 | 8.410808661 | 4.860901918 | 0.000336602 | 0.002819116 | 0.023551672 |
| ATXN1 | -2.148553478 | 4.977685706 | -4.860595682 | 0.000336783 | 0.002819116 | 0.023002772 |
| MITD1 | -1.262678663 | 10.5423966 | -4.859388613 | 0.000337496 | 0.002823528 | 0.0208391 |
| ODF3B | -1.698193367 | 8.228808829 | -4.858773742 | 0.000337861 | 0.002825014 | 0.019736875 |
| AK7 | 1.565115685 | 2.448790347 | 4.8583967 | 0.000338084 | 0.002825323 | 0.019060965 |
| ERP27 | -1.982104123 | 7.687089705 | -4.857348854 | 0.000338706 | 0.002828959 | 0.017182431 |
| ANKRD27 | 1.677263437 | 8.025421336 | 4.856733606 | 0.000339071 | 0.002830452 | 0.016079379 |
| LONRF2 | 1.396808664 | 2.504754509 | 4.856066828 | 0.000339468 | 0.002832203 | 0.014883889 |
| GPR160 | -1.781160071 | 8.524350127 | -4.855315389 | 0.000339916 | 0.002834378 | 0.013536543 |
| NAA15 | 1.742067569 | 6.03648099 | 4.854774599 | 0.000340238 | 0.002835507 | 0.012566854 |
| CHRNE | 1.158374022 | 7.173647989 | 4.853809061 | 0.000340815 | 0.002838752 | 0.010835461 |
| SNRNP27 | -1.048777071 | 8.001070492 | -4.853438645 | 0.000341037 | 0.002839036 | 0.010171205 |
| ZBTB46 | 2.36296689 | 5.382896662 | 4.851157367 | 0.000342404 | 0.002848855 | 0.006079891 |
| OR7A17 | 1.009929429 | 4.078699164 | 4.850071241 | 0.000343057 | 0.002852723 | 0.004131778 |
| MAML3 | 1.086669755 | 7.732530733 | 4.845689494 | 0.000345705 | 0.002873166 | -0.003728919 |
| AUNIP | 2.115718152 | 7.134394528 | 4.84412869 | 0.000346654 | 0.002877891 | -0.006529505 |
| P3H4 | -1.635246395 | 7.601466725 | -4.8418801 | 0.000348025 | 0.00288611 | -0.010564716 |
| PKIA | -3.13919269 | 8.150980404 | -4.841448086 | 0.000348289 | 0.00288672 | -0.011340057 |
| TMEM229B | -1.732889006 | 9.298821872 | -4.839596031 | 0.000349423 | 0.002894539 | -0.014664218 |
| ORC1 | 2.240515885 | 5.351756891 | 4.83717987 | 0.000350909 | 0.002903672 | -0.019001487 |
| ZNF765 | 1.030668621 | 5.204762357 | 4.836157835 | 0.00035154 | 0.002905715 | -0.02083636 |
| KIF19 | -1.423118788 | 7.197127046 | -4.835456664 | 0.000351973 | 0.002907709 | -0.022095256 |
| TTYH2 | -1.95155394 | 6.160621009 | -4.834992881 | 0.00035226 | 0.002908493 | -0.022927971 |
| VSTM2L | 1.639957668 | 5.45515426 | 4.833780682 | 0.00035301 | 0.002913106 | -0.025104581 |
| PDE6D | -1.700904574 | 8.769746406 | -4.832573538 | 0.00035376 | 0.002917701 | -0.02727229 |
| SMG5 | 1.472542544 | 8.382035772 | 4.82997657 | 0.000355378 | 0.00292945 | -0.031936344 |
| RFLNA | 1.011370302 | 5.320300541 | 4.82933454 | 0.000355779 | 0.002931162 | -0.033089531 |
| PRMT2 | -1.196777076 | 9.576801552 | -4.827659583 | 0.000356828 | 0.002938205 | -0.036098247 |
| STIL | 2.097221245 | 3.22662324 | 4.826855143 | 0.000357333 | 0.002940764 | -0.037543376 |
| NRIP3 | 1.157411329 | 5.581896823 | 4.825404383 | 0.000358245 | 0.002945073 | -0.040149781 |
| TNFSF14 | -1.203939999 | 3.945830209 | -4.821742407 | 0.000360559 | 0.002962004 | -0.046729926 |
| PPP3CA | 1.09133092 | 8.783698223 | 4.821526809 | 0.000360696 | 0.002962004 | -0.047117381 |
| INTS13 | 1.165245538 | 7.863542953 | 4.820566422 | 0.000361305 | 0.002965403 | -0.048843376 |
| ECT2 | 1.254434899 | 6.255041207 | 4.819454339 | 0.000362012 | 0.002967993 | -0.050842133 |
| HOOK1 | -1.729546476 | 3.029902421 | -4.818736724 | 0.00036247 | 0.002970134 | -0.052131988 |
| TAS2R43 | 2.071474978 | 6.4633992 | 4.817040494 | 0.000363553 | 0.002976223 | -0.05518107 |
| ABCA3 | -1.06574683 | 6.492691427 | -4.816957544 | 0.000363606 | 0.002976223 | -0.055330186 |
| DKK3 | -2.058101919 | 2.730215395 | -4.815051947 | 0.000364827 | 0.002982936 | -0.058756047 |
| SUCO | 1.42902403 | 10.20267097 | 4.814602778 | 0.000365115 | 0.002982936 | -0.05956362 |
| PGAM1 | -1.215294298 | 12.01578031 | -4.814351316 | 0.000365276 | 0.002982936 | -0.060015741 |
| FYN | -2.989427467 | 11.17270706 | -4.814143242 | 0.00036541 | 0.002982936 | -0.060389857 |
| IMPA2 | -1.410799372 | 8.489699089 | -4.813525167 | 0.000365808 | 0.002984574 | -0.061501187 |
| ZNF443 | 2.120850736 | 4.882759796 | 4.811620255 | 0.000367036 | 0.002992982 | -0.064926594 |
| CD8A | -2.292954181 | 10.96948903 | -4.810368027 | 0.000367846 | 0.002996708 | -0.067178582 |
| AC1267552 | 1.396478877 | 10.95858827 | 4.810301888 | 0.000367888 | 0.002996708 | -0.067297532 |
| FKBP15 | -1.668595292 | 8.572501967 | -4.807641416 | 0.000369615 | 0.003007539 | -0.072082727 |
| RPA4 | 4.840865053 | 7.962636332 | 4.806944437 | 0.000370069 | 0.003009614 | -0.07333647 |
| GLUL | 1.740755512 | 11.25025477 | 4.806383316 | 0.000370434 | 0.003010416 | -0.074345872 |
| ATRAID | -1.292287278 | 10.25088711 | -4.806183637 | 0.000370565 | 0.003010416 | -0.074705083 |
| GPC2 | 1.641906969 | 8.727527688 | 4.805457865 | 0.000371038 | 0.003012649 | -0.076010748 |
| KCNA3 | -1.048135395 | 7.60459179 | -4.802069342 | 0.000373259 | 0.003027431 | -0.082107533 |
| PCDHGA9 | 2.239165985 | 4.128568548 | 4.800631927 | 0.000374205 | 0.00303348 | -0.084694207 |
| TM2D2 | -1.070226577 | 8.977053348 | -4.79911657 | 0.000375205 | 0.003038336 | -0.087421407 |
| NCBP2L | -1.461267867 | 2.571143379 | -4.796928548 | 0.000376653 | 0.003045099 | -0.091359686 |
| GOLGA6L6 | 1.591174311 | 2.629536581 | 4.796814846 | 0.000376729 | 0.003045099 | -0.091564357 |
| VSIG8 | -1.435010009 | 5.080353274 | -4.796769082 | 0.000376759 | 0.003045099 | -0.091646737 |
| POLE2 | 1.981040055 | 6.4653102 | 4.796641823 | 0.000376844 | 0.003045099 | -0.091875812 |
| CCSER2 | -1.824938329 | 8.498841142 | -4.794000847 | 0.000378601 | 0.00305604 | -0.096630223 |
| ZNF527 | 1.032628345 | 5.69529468 | 4.793681514 | 0.000378814 | 0.003056132 | -0.097205156 |
| SELENOF | -1.024853446 | 10.91685981 | -4.793067479 | 0.000379224 | 0.003057812 | -0.098310712 |
| STARD10 | -1.465201856 | 7.68979945 | -4.792524419 | 0.000379587 | 0.003059111 | -0.099288517 |
| MED15 | 1.39565966 | 8.923912126 | 4.791636523 | 0.000380182 | 0.003062273 | -0.100887289 |
| MRVI1 | -2.904223708 | 7.313537718 | -4.790511499 | 0.000380936 | 0.00306672 | -0.102913176 |
| KIF22 | 1.358071057 | 9.96061105 | 4.788637973 | 0.000382196 | 0.003074348 | -0.106287259 |
| C10orf62 | 1.343987986 | 2.660974281 | 4.788499248 | 0.00038229 | 0.003074348 | -0.10653711 |
| AMBRA1 | 1.22346182 | 8.376317782 | 4.785094877 | 0.000384591 | 0.003089014 | -0.11266925 |
| CES2 | 1.026173655 | 8.587740139 | 4.7848972 | 0.000384725 | 0.003089014 | -0.113025359 |
| RBM6 | 1.739655522 | 9.52927224 | 4.783728394 | 0.000385519 | 0.003092109 | -0.115131019 |
| FBXO30 | 1.598428388 | 7.721917428 | 4.781137871 | 0.000387284 | 0.003104623 | -0.119798545 |
| COMMD8 | -1.650906835 | 9.772023139 | -4.780782094 | 0.000387527 | 0.003104929 | -0.120439635 |
| TTBK2 | 1.104850119 | 5.510904408 | 4.777075763 | 0.000390069 | 0.003121992 | -0.12711912 |
| INPP5J | 1.545967986 | 4.620362056 | 4.775608081 | 0.00039108 | 0.003126783 | -0.129764598 |
| TTI1 | 1.062626583 | 9.063170433 | 4.774946749 | 0.000391537 | 0.003128783 | -0.130956723 |
| VHL | 1.343098245 | 9.485586244 | 4.77455207 | 0.000391809 | 0.00312902 | -0.131668201 |
| FBXL16 | -3.488945349 | 7.113754615 | -4.774306316 | 0.000391979 | 0.00312902 | -0.132111223 |
| TRIM56 | 1.397691702 | 8.152029946 | 4.773650467 | 0.000392433 | 0.003130993 | -0.133293565 |
| DOCK9 | -1.201454975 | 5.639874732 | -4.772330525 | 0.000393348 | 0.003133739 | -0.135673264 |
| FCHSD1 | -1.402378477 | 9.82470624 | -4.772259121 | 0.000393398 | 0.003133739 | -0.135802003 |
| KMT2E | 1.308726308 | 11.50687158 | 4.771370267 | 0.000394016 | 0.003136481 | -0.137404623 |
| CDC42 | -1.01052091 | 11.51544847 | -4.771168202 | 0.000394156 | 0.003136481 | -0.137768962 |
| OR2T12 | 1.799826703 | 3.174778525 | 4.766830529 | 0.000397186 | 0.003158929 | -0.145591303 |
| CCDC149 | -3.577174363 | 6.711258703 | -4.763714734 | 0.000399377 | 0.003174689 | -0.151211531 |
| PIAS2 | 1.419357867 | 6.485834458 | 4.761510735 | 0.000400934 | 0.003185399 | -0.155187759 |
| SYVN1 | 1.082509321 | 9.408492403 | 4.761024572 | 0.000401279 | 0.003185455 | -0.156064921 |
| CNTNAP3B | -1.301763668 | 3.45707409 | -4.760907265 | 0.000401362 | 0.003185455 | -0.156276577 |
| HPDL | 1.345003904 | 7.80822381 | 4.759815662 | 0.000402136 | 0.003189863 | -0.158246223 |
| IL17RC | 1.219897469 | 5.490276867 | 4.759531604 | 0.000402338 | 0.003189863 | -0.158758789 |
| RREB1 | 1.51787654 | 6.758218935 | 4.758081188 | 0.00040337 | 0.003196374 | -0.161376126 |
| PRDM1 | -2.609812058 | 8.026381462 | -4.757299734 | 0.000403928 | 0.003199116 | -0.162786395 |
| 5-Mar | 1.026460326 | 5.672224938 | 4.756063745 | 0.000404811 | 0.003201319 | -0.165017096 |
| PUS3 | -1.155267149 | 8.944354685 | -4.756023266 | 0.00040484 | 0.003201319 | -0.165090155 |
| RECQL4 | 1.743401597 | 7.837021942 | 4.754465352 | 0.000405956 | 0.003208469 | -0.167902125 |
| EMC1 | 1.00873073 | 6.960154175 | 4.754056178 | 0.000406249 | 0.003209116 | -0.168640713 |
| SMARCAD1 | 1.089502065 | 8.023681663 | 4.753426443 | 0.000406702 | 0.003210873 | -0.16977747 |
| DSCC1 | 3.329687894 | 4.932148733 | 4.753070248 | 0.000406958 | 0.003210873 | -0.17042047 |
| SAMD9 | -1.843829695 | 5.830095126 | -4.75260922 | 0.000407289 | 0.003210873 | -0.171252734 |
| CDC23 | 1.278142719 | 6.587395231 | 4.752567595 | 0.000407319 | 0.003210873 | -0.17132788 |
| DCLRE1C | 1.108669654 | 7.75639399 | 4.751355454 | 0.000408193 | 0.003216085 | -0.173516207 |
| RSPH9 | -1.082446394 | 8.946164347 | -4.750980798 | 0.000408463 | 0.003216543 | -0.174192623 |
| LTN1 | 1.04078769 | 5.374584127 | 4.746268778 | 0.00041188 | 0.0032384 | -0.182701257 |
| KMT5A | 1.174490111 | 10.61905222 | 4.745706247 | 0.00041229 | 0.003239896 | -0.183717209 |
| SSBP2 | 2.14198575 | 5.156627401 | 4.745111693 | 0.000412724 | 0.003239896 | -0.184791035 |
| RERG | 1.065773234 | 7.944801137 | 4.745005908 | 0.000412801 | 0.003239896 | -0.184982098 |
| ABLIM1 | -2.026642859 | 8.405380183 | -4.744779384 | 0.000412966 | 0.003239896 | -0.185391237 |
| ZNF234 | 1.037574628 | 6.868048415 | 4.744542008 | 0.00041314 | 0.003239896 | -0.185819984 |
| APLP1 | 1.113069283 | 6.738120679 | 4.743670775 | 0.000413777 | 0.003240262 | -0.187393652 |
| KIAA2013 | -1.057798443 | 11.19308532 | -4.743617103 | 0.000413816 | 0.003240262 | -0.187490599 |
| KIF14 | 3.126959533 | 5.945740864 | 4.737602368 | 0.000418242 | 0.00326807 | -0.198357186 |
| VDR | -1.179057592 | 7.613818736 | -4.736533297 | 0.000419034 | 0.003272569 | -0.200289072 |
| PRR23D2 | 2.404358011 | 3.596019407 | 4.735337358 | 0.000419922 | 0.003277812 | -0.202450376 |
| S1PR3 | -2.766499915 | 5.307553082 | -4.732716546 | 0.000421874 | 0.003291355 | -0.207187293 |
| KIF7 | 1.975749305 | 3.156492532 | 4.73186773 | 0.000422508 | 0.003294607 | -0.208721634 |
| TCF25 | 1.448778324 | 12.8311166 | 4.731502343 | 0.000422781 | 0.003295042 | -0.209382141 |
| CREB3L4 | 1.566027654 | 9.610223095 | 4.729165558 | 0.000424534 | 0.003306695 | -0.213606699 |
| MCUB | -3.964672099 | 10.03908087 | -4.72892729 | 0.000424713 | 0.003306695 | -0.214037487 |
| LHX6 | 2.684552987 | 3.80739047 | 4.728318435 | 0.000425171 | 0.003307814 | -0.215138326 |
| HMGXB3 | 1.346983614 | 10.16831909 | 4.728155861 | 0.000425294 | 0.003307814 | -0.215432274 |
| F11R | 1.840581319 | 11.99032586 | 4.727715966 | 0.000425625 | 0.003308693 | -0.21622766 |
| PARP9 | -1.623741273 | 7.808157669 | -4.726154402 | 0.000426803 | 0.003314661 | -0.219051348 |
| ARHGAP30 | -1.471582728 | 10.22855089 | -4.725884343 | 0.000427008 | 0.003314661 | -0.219539708 |
| GLI2 | 1.142097456 | 4.187164468 | 4.725829663 | 0.000427049 | 0.003314661 | -0.219638589 |
| AP000350.4 | -1.464340175 | 7.238601911 | -4.724118268 | 0.000428345 | 0.003323019 | -0.222733593 |
| UBE2V2 | -1.443986296 | 7.019132187 | -4.721723011 | 0.000430166 | 0.003335439 | -0.227065908 |
| PSME4 | 1.48197762 | 6.809818518 | 4.71854795 | 0.000432592 | 0.003351354 | -0.232809676 |
| UHRF1BP1 | 1.713609508 | 9.017617344 | 4.718458376 | 0.000432661 | 0.003351354 | -0.232971734 |
| STRBP | 1.491760332 | 7.718129503 | 4.717847723 | 0.000433129 | 0.003353125 | -0.234076559 |
| PABPC4 | 1.212328367 | 8.628073836 | 4.717583438 | 0.000433332 | 0.003353125 | -0.234554731 |
| PDK3 | -1.192660845 | 7.442146814 | -4.717192929 | 0.000433632 | 0.003353733 | -0.235261296 |
| KLHDC7B | -1.961320262 | 8.471076468 | -4.716227706 | 0.000434375 | 0.00335776 | -0.23700779 |
| OAS3 | -1.640918736 | 6.746128139 | -4.71535077 | 0.00043505 | 0.003359743 | -0.238594627 |
| MAP2K6 | -1.015359642 | 5.572786966 | -4.715318981 | 0.000435075 | 0.003359743 | -0.238652152 |
| ING1 | -1.257348994 | 8.942230948 | -4.713928701 | 0.000436148 | 0.003366315 | -0.241168085 |
| PARP1 | 1.512637588 | 8.02122078 | 4.712006604 | 0.000437636 | 0.003376083 | -0.244646792 |
| LIMD1 | 1.027777282 | 6.833126179 | 4.710942646 | 0.000438462 | 0.003379613 | -0.246572577 |
| ZNF12 | 1.528096203 | 7.140646807 | 4.710480654 | 0.000438822 | 0.003380065 | -0.247408833 |
| FZD1 | -1.837944866 | 3.993923064 | -4.710121293 | 0.000439101 | 0.0033805 | -0.248059332 |
| CBX7 | -1.934377136 | 10.04455005 | -4.708085675 | 0.000440689 | 0.003391 | -0.251744396 |
| WWP1 | -1.408933276 | 7.850990257 | -4.707763258 | 0.000440941 | 0.003391216 | -0.252328107 |
| CCDC9 | 1.427298666 | 14.8696177 | 4.707085336 | 0.000441471 | 0.003393573 | -0.253555474 |
| RPS6KA4 | -1.166334123 | 9.871280667 | -4.705336314 | 0.000442843 | 0.003400663 | -0.256722294 |
| OR5D13 | 1.267490097 | 2.128404048 | 4.701616489 | 0.000445774 | 0.00342144 | -0.263458659 |
| CFAP97D2 | -1.382459003 | 4.161892368 | -4.700203173 | 0.000446893 | 0.003428292 | -0.266018498 |
| WDR33 | 1.155999395 | 7.88426242 | 4.696675805 | 0.000449699 | 0.003446326 | -0.272408363 |
| GALNT12 | -2.421686729 | 4.036936362 | -4.695847895 | 0.00045036 | 0.003449648 | -0.273908334 |
| CXCL1 | -2.100192318 | 7.332098095 | -4.695141014 | 0.000450925 | 0.003452233 | -0.275189095 |
| FANCA | 1.59176311 | 7.047745483 | 4.692276777 | 0.000453223 | 0.003468075 | -0.280379233 |
| CCER2 | 1.466470636 | 7.401812906 | 4.689323489 | 0.000455606 | 0.003483829 | -0.285731714 |
| TMSB10 | -1.486416475 | 15.00937339 | -4.689155058 | 0.000455742 | 0.003483829 | -0.286037004 |
| NUDT18 | -1.076494882 | 8.836797124 | -4.688068055 | 0.000456623 | 0.003488799 | -0.288007337 |
| TADA2B | -2.538878273 | 5.839871346 | -4.685093784 | 0.000459041 | 0.003505507 | -0.293399272 |
| IGFBP3 | -1.395066621 | 4.274863624 | -4.683368562 | 0.000460449 | 0.003514494 | -0.296527315 |
| TM7SF3 | 1.782003863 | 11.04223702 | 4.68280292 | 0.000460912 | 0.003516255 | -0.297552969 |
| PHF8 | 1.677546985 | 8.107050372 | 4.682017563 | 0.000461556 | 0.003518241 | -0.298977081 |
| TMC3 | 1.257158018 | 5.419898952 | 4.681855573 | 0.000461689 | 0.003518241 | -0.299270832 |
| ICAM1 | -2.309004487 | 9.067910881 | -4.681635299 | 0.000461869 | 0.003518241 | -0.299670279 |
| MESP2 | -1.174649281 | 7.779064143 | -4.68109707 | 0.000462311 | 0.003519837 | -0.300646329 |
| SLC35E4 | 1.220123972 | 10.81098621 | 4.68075615 | 0.000462591 | 0.003520201 | -0.301264588 |
| PROC | -2.164704084 | 4.855304147 | -4.680258349 | 0.000463001 | 0.003521547 | -0.302167374 |
| AL121758.1 | -1.661355923 | 9.022795398 | -4.679060389 | 0.000463987 | 0.003527279 | -0.304340044 |
| ENHO | -1.96456469 | 7.181813014 | -4.67561431 | 0.000466837 | 0.003545284 | -0.310590906 |
| TRAPPC10 | 1.83718139 | 9.025247761 | 4.675348672 | 0.000467057 | 0.003545284 | -0.311072805 |
| LNPK | -1.167172356 | 5.230079397 | -4.673014696 | 0.000468999 | 0.003556459 | -0.315307248 |
| NTN4 | -2.133489054 | 2.714326107 | -4.671120429 | 0.000470581 | 0.00356667 | -0.318744394 |
| RFX3 | 1.283973821 | 5.922867579 | 4.668491845 | 0.000472785 | 0.003581586 | -0.323514629 |
| CDH11 | 1.158965614 | 2.236325723 | 4.667047903 | 0.000474001 | 0.003588999 | -0.326135361 |
| CAMK2N1 | -1.614582395 | 5.911447087 | -4.665668144 | 0.000475165 | 0.003595648 | -0.328639821 |
| STK26 | 1.153556281 | 11.94939974 | 4.665445341 | 0.000475354 | 0.003595648 | -0.32904426 |
| MDFIC | -2.131710292 | 8.279134834 | -4.664076109 | 0.000476513 | 0.00360082 | -0.331529858 |
| NUDCD1 | 2.02100367 | 2.803130199 | 4.662539158 | 0.000477817 | 0.003608471 | -0.334320169 |
| TRIM45 | 1.379501669 | 7.120459183 | 4.662125731 | 0.000478169 | 0.003608471 | -0.335070785 |
| ID2 | -2.445692149 | 12.00307176 | -4.662042324 | 0.00047824 | 0.003608471 | -0.335222221 |
| SSTR4 | 1.07714913 | 5.555276857 | 4.661050995 | 0.000479084 | 0.003612081 | -0.337022161 |
| SART1 | 1.303488731 | 8.767001042 | 4.660704956 | 0.000479379 | 0.003612081 | -0.337650484 |
| ACOD1 | 1.286166862 | 8.748896447 | 4.660641125 | 0.000479433 | 0.003612081 | -0.337766387 |
| SOD2 | -2.45799947 | 12.3022726 | -4.659272569 | 0.000480602 | 0.003616947 | -0.340251494 |
| GUCY1A1 | 2.716406907 | 8.56774913 | 4.658880535 | 0.000480938 | 0.003616947 | -0.340963412 |
| XPC | 1.056298456 | 10.35863728 | 4.658767696 | 0.000481034 | 0.003616947 | -0.341168326 |
| KIF23 | 1.685689757 | 8.412860574 | 4.656717169 | 0.000482793 | 0.003626569 | -0.344892308 |
| ARHGAP24 | -2.459725089 | 4.538066496 | -4.654145868 | 0.000485007 | 0.003641398 | -0.349562737 |
| CCNI2 | -2.140682462 | 3.227138026 | -4.653309187 | 0.00048573 | 0.003645019 | -0.351082617 |
| CENPO | 1.30561241 | 6.689411676 | 4.65096276 | 0.000487763 | 0.003654846 | -0.355345457 |
| ZIM2 | 1.600279939 | 5.312496199 | 4.650381786 | 0.000488268 | 0.003656764 | -0.356401028 |
| ADGRG4 | 1.755639956 | 5.205692211 | 4.649000844 | 0.00048947 | 0.003662201 | -0.358910212 |
| CEACAM6 | -2.387581713 | 5.205785337 | -4.648244581 | 0.000490129 | 0.003664174 | -0.36028444 |
| FBXL5 | -1.140320992 | 10.58373196 | -4.648144037 | 0.000490217 | 0.003664174 | -0.360467145 |
| GPR158 | 1.658117193 | 6.660053352 | 4.645855615 | 0.000492219 | 0.003677323 | -0.364625916 |
| DUSP7 | -1.065436167 | 5.81121397 | -4.645193313 | 0.0004928 | 0.003679848 | -0.365829632 |
| LRCH1 | 1.546566204 | 6.013715374 | 4.643706053 | 0.000494107 | 0.003685976 | -0.368532866 |
| GOLGA6L10 | 2.138813103 | 9.893855465 | 4.641299091 | 0.00049623 | 0.003698535 | -0.372908265 |
| CDK5 | -1.029600261 | 6.763975729 | -4.64124421 | 0.000496279 | 0.003698535 | -0.373008037 |
| SHANK1 | 1.12824962 | 11.03428171 | 4.638597908 | 0.000498624 | 0.003712362 | -0.377819269 |
| HMBOX1 | 3.728495338 | 9.880012918 | 4.633832056 | 0.000502878 | 0.003738517 | -0.386486011 |
| FAM234A | 1.429156175 | 10.89226995 | 4.632889814 | 0.000503723 | 0.003742965 | -0.388199783 |
| 6-Sep | 1.312199031 | 10.58402051 | 4.630667329 | 0.000505723 | 0.003754142 | -0.392242483 |
| NSMCE1 | -1.204626388 | 9.902339413 | -4.630024389 | 0.000506303 | 0.003756608 | -0.393412092 |
| SLC31A1 | -1.813576116 | 6.293702553 | -4.627114256 | 0.000508937 | 0.003774304 | -0.398706654 |
| PSD4 | -1.012736476 | 6.53667064 | -4.626822186 | 0.000509202 | 0.003774423 | -0.399238084 |
| SOHLH2 | 2.197998505 | 2.964289628 | 4.626188947 | 0.000509777 | 0.00377585 | -0.400390316 |
| SPDYE5 | 1.21471236 | 6.337142837 | 4.625651691 | 0.000510266 | 0.00377585 | -0.401367932 |
| DCTN5 | 1.363875311 | 5.794707028 | 4.625514011 | 0.000510391 | 0.00377585 | -0.401618465 |
| GLDN | 1.325621493 | 4.839266963 | 4.624629826 | 0.000511197 | 0.003779964 | -0.403227452 |
| SHISAL2A | -1.809534415 | 8.617733977 | -4.623233497 | 0.000512472 | 0.003787542 | -0.405768584 |
| TMEM132C | 1.659432612 | 2.974830387 | 4.621868003 | 0.000513722 | 0.003794928 | -0.408253809 |
| NPLOC4 | 1.13052822 | 7.443147094 | 4.61973677 | 0.000515679 | 0.003807529 | -0.412133103 |
| PALLD | 1.744327219 | 6.103079593 | 4.618228703 | 0.000517068 | 0.003815929 | -0.414878403 |
| ZNF407 | 1.472379019 | 6.994170673 | 4.617457651 | 0.00051778 | 0.003819275 | -0.416282132 |
| SLAMF6 | -1.440539461 | 7.781123951 | -4.616919231 | 0.000518278 | 0.003819275 | -0.417262384 |
| ZNF730 | 1.780133636 | 2.896231274 | 4.616135269 | 0.000519004 | 0.003822763 | -0.418689727 |
| B3GALNT1 | 1.561522776 | 7.904318067 | 4.615069531 | 0.000519992 | 0.003828039 | -0.420630206 |
| CYR61 | 1.669154308 | 2.363015591 | 4.614189595 | 0.000520809 | 0.003830475 | -0.422232471 |
| TGFB1 | -1.477203255 | 10.57657272 | -4.612777419 | 0.000522124 | 0.00383828 | -0.424804064 |
| POM121C | 1.208860302 | 10.2824741 | 4.610572667 | 0.000524183 | 0.003849478 | -0.428819387 |
| CLC | -5.602522432 | 9.841046778 | -4.610333586 | 0.000524407 | 0.003849478 | -0.429254836 |
| CABP5 | -1.081005077 | 9.642110567 | -4.610330942 | 0.000524409 | 0.003849478 | -0.429259652 |
| ASMT | -1.893247877 | 4.645592678 | -4.609375048 | 0.000525305 | 0.003852328 | -0.43100073 |
| SQOR | -1.335537955 | 12.81758152 | -4.609374103 | 0.000525306 | 0.003852328 | -0.431002452 |
| LRRTM1 | 1.303962375 | 9.289855058 | 4.608665408 | 0.000525971 | 0.003855339 | -0.432293343 |
| ADCK5 | -1.168726499 | 7.960355163 | -4.607231376 | 0.00052732 | 0.003861489 | -0.434905605 |
| PRKRIP1 | 1.174485598 | 4.50466884 | 4.606402276 | 0.000528101 | 0.003861901 | -0.436416012 |
| PI3 | -2.116497399 | 8.239304685 | -4.60634601 | 0.000528154 | 0.003861901 | -0.436518518 |
| TYROBP | -2.425129761 | 16.0883875 | -4.606090178 | 0.000528395 | 0.003861901 | -0.436984595 |
| PWWP2B | 1.144621361 | 11.64873417 | 4.605161009 | 0.000529273 | 0.00386645 | -0.438677425 |
| KDM6A | 1.347137627 | 6.34939733 | 4.602893661 | 0.000531421 | 0.003878401 | -0.442808646 |
| GPSM1 | 1.904134022 | 9.545362832 | 4.601948837 | 0.000532319 | 0.003883082 | -0.444530326 |
| RPL37A | -1.414879934 | 6.709918552 | -4.601233755 | 0.000532999 | 0.003886175 | -0.445833432 |
| SIL1 | 1.358407253 | 8.716038895 | 4.60066647 | 0.00053354 | 0.003888244 | -0.446867244 |
| RHEX | 2.707847979 | 11.10189524 | 4.600393197 | 0.0005338 | 0.003888273 | -0.447365265 |
| RAB39B | -1.98496191 | 5.46951339 | -4.599720025 | 0.000534443 | 0.00388844 | -0.448592108 |
| EDN2 | -1.843917546 | 3.720177468 | -4.599562465 | 0.000534593 | 0.00388844 | -0.448879267 |
| GIGYF1 | 1.098289413 | 9.722364805 | 4.598607693 | 0.000535506 | 0.003893211 | -0.450619422 |
| PALM | 3.000024959 | 9.786472921 | 4.597451135 | 0.000536614 | 0.003899395 | -0.452727485 |
| BORCS6 | -1.234213266 | 9.063489075 | -4.595648147 | 0.000538346 | 0.003910104 | -0.456014087 |
| S100A2 | 1.078912445 | 7.135693558 | 4.59151377 | 0.000542339 | 0.003937221 | -0.463551829 |
| ZNF780B | 1.685582708 | 4.789645413 | 4.590426064 | 0.000543395 | 0.003942996 | -0.465535228 |
| ARHGAP10 | 1.316712841 | 6.018206209 | 4.590045353 | 0.000543765 | 0.003943792 | -0.466229475 |
| DISC1 | 1.38596691 | 5.688530275 | 4.587247295 | 0.000546492 | 0.003961677 | -0.471332354 |
| CD24 | -1.70281231 | 9.306250234 | -4.58640663 | 0.000547315 | 0.003965741 | -0.472865659 |
| ZBTB21 | 1.06928823 | 8.528014455 | 4.58498318 | 0.00054871 | 0.003973541 | -0.47546209 |
| CACNG6 | -3.931199726 | 6.125453685 | -4.58477338 | 0.000548916 | 0.003973541 | -0.475844794 |
| HCAR3 | -1.842500766 | 6.889342226 | -4.583967302 | 0.000549708 | 0.003977374 | -0.477315229 |
| FXR1 | 1.137034263 | 6.760718057 | 4.583635947 | 0.000550034 | 0.003977627 | -0.477919701 |
| AC092587.1 | -1.692320264 | 3.763665977 | -4.583304271 | 0.00055036 | 0.003977627 | -0.478524772 |
| ARHGAP23 | 2.975722396 | 5.27049564 | 4.58292966 | 0.000550729 | 0.003977627 | -0.479208181 |
| PDK4 | -1.215670528 | 7.218828122 | -4.582864933 | 0.000550793 | 0.003977627 | -0.479326266 |
| CIDEC | -1.098495091 | 8.237889271 | -4.582153279 | 0.000551494 | 0.003980797 | -0.480624598 |
| CD274 | -2.009982426 | 4.58946859 | -4.581371211 | 0.000552267 | 0.003983479 | -0.482051455 |
| ZKSCAN2 | 1.515405059 | 6.660251116 | 4.581244452 | 0.000552392 | 0.003983479 | -0.482282728 |
| ZNF507 | -1.332697428 | 4.493784354 | -4.580827244 | 0.000552804 | 0.003983902 | -0.483043944 |
| SRD5A1 | -1.900013519 | 6.901836938 | -4.58054417 | 0.000553084 | 0.003983902 | -0.483560435 |
| SERPINB4 | 1.083445193 | 4.235924446 | 4.58035191 | 0.000553275 | 0.003983902 | -0.483911234 |
| NR2C1 | 1.284146764 | 7.208464655 | 4.580122059 | 0.000553502 | 0.003983902 | -0.484330628 |
| PREX2 | 1.344931119 | 7.845124509 | 4.579448805 | 0.000554169 | 0.003985738 | -0.485559101 |
| RNF144A | 1.555578064 | 5.672526973 | 4.579333756 | 0.000554284 | 0.003985738 | -0.485769034 |
| KCNH6 | 1.698845798 | 3.910752124 | 4.578952697 | 0.000554662 | 0.003986564 | -0.486464371 |
| ARPC5 | -1.283991456 | 7.283780013 | -4.577743021 | 0.000555864 | 0.003991416 | -0.488671829 |
| TCF3 | 1.402202241 | 11.30968877 | 4.576635196 | 0.000556967 | 0.00399555 | -0.490693564 |
| BEND4 | -1.703218711 | 3.610956294 | -4.575413607 | 0.000558186 | 0.004000727 | -0.492923068 |
| NF1 | 1.060726921 | 5.921564495 | 4.575382848 | 0.000558216 | 0.004000727 | -0.492979208 |
| PARM1 | -1.279523307 | 3.458210765 | -4.573804833 | 0.000559795 | 0.004008545 | -0.495859461 |
| TMPRSS5 | 1.473514575 | 3.167049536 | 4.573607563 | 0.000559993 | 0.004008545 | -0.496219546 |
| PSMD5 | 1.024818393 | 5.884188858 | 4.573415754 | 0.000560185 | 0.004008545 | -0.496569666 |
| GRIN3A | -1.586667524 | 7.378486966 | -4.573236096 | 0.000560366 | 0.004008545 | -0.496897608 |
| C3orf38 | -1.061868467 | 8.217554966 | -4.571493002 | 0.000562117 | 0.004016262 | -0.500079588 |
| GCH1 | -1.164025777 | 8.073707071 | -4.571371057 | 0.00056224 | 0.004016262 | -0.50030221 |
| NHLRC2 | 1.144068906 | 7.021545951 | 4.570315896 | 0.000563303 | 0.004021625 | -0.502228555 |
| MAP3K6 | -1.333110233 | 7.281309015 | -4.570099355 | 0.000563521 | 0.004021625 | -0.502623895 |
| APOC2 | 4.555402389 | 4.625815204 | 4.569660402 | 0.000563964 | 0.004021816 | -0.503425312 |
| DNAJC4 | -1.243592075 | 8.017969925 | -4.569546888 | 0.000564079 | 0.004021816 | -0.503632563 |
| MMACHC | 1.166194045 | 6.434805873 | 4.567954096 | 0.00056569 | 0.004031406 | -0.506540788 |
| RFX4 | 1.435404539 | 8.557020161 | 4.565610498 | 0.000568069 | 0.004046459 | -0.510820379 |
| SMARCA2 | 1.454392932 | 8.456986588 | 4.56492206 | 0.00056877 | 0.004049547 | -0.512077631 |
| KRTAP12-2 | 1.562067525 | 9.141017097 | 4.564396559 | 0.000569306 | 0.004051457 | -0.513037357 |
| PSG11 | 1.236870673 | 4.868798482 | 4.563319943 | 0.000570405 | 0.004056865 | -0.515003677 |
| DPYSL2 | -2.997549156 | 5.65949361 | -4.563127642 | 0.000570601 | 0.004056865 | -0.515354907 |
| C3orf70 | 1.436097147 | 3.823794816 | 4.561859233 | 0.000571899 | 0.004064187 | -0.517671702 |
| RBM14 | 1.404707347 | 10.01286468 | 4.557216657 | 0.000576676 | 0.004096214 | -0.526152998 |
| NUP58 | 1.272130144 | 7.388232996 | 4.556217002 | 0.000577711 | 0.004101636 | -0.52797952 |
| GGACT | -2.430932709 | 7.973865448 | -4.555527929 | 0.000578424 | 0.004104781 | -0.529238623 |
| THAP7 | -1.365467577 | 7.311794276 | -4.554555001 | 0.000579434 | 0.004108097 | -0.531016485 |
| RMI2 | 1.814256026 | 8.765673252 | 4.553637824 | 0.000580387 | 0.004112932 | -0.532692562 |
| AP4M1 | 1.302291663 | 6.049936605 | 4.552630052 | 0.000581437 | 0.004118442 | -0.534534299 |
| KANTR | 1.006512356 | 4.860990017 | 4.55161487 | 0.000582496 | 0.004124016 | -0.536389688 |
| GANAB | 1.197187519 | 10.22463606 | 4.551055091 | 0.000583081 | 0.004126229 | -0.537412811 |
| OR52I1 | 1.800673806 | 3.764804809 | 4.550348781 | 0.00058382 | 0.004129529 | -0.538703798 |
| BAD | -1.463055313 | 9.77152464 | -4.549351664 | 0.000584864 | 0.004134988 | -0.540526409 |
| KANK2 | 2.388987677 | 9.333905333 | 4.547543014 | 0.000586764 | 0.004144552 | -0.543832679 |
| NDUFS6 | -1.078243941 | 12.00392631 | -4.54585025 | 0.000588548 | 0.004155216 | -0.546927418 |
| LIPN | -1.424571627 | 7.589678868 | -4.545582775 | 0.000588831 | 0.004155273 | -0.547416448 |
| GTF3C2 | 1.254460484 | 10.46197828 | 4.5446269 | 0.000589841 | 0.004158279 | -0.549164156 |
| CTDSPL | -2.574064577 | 6.596909222 | -4.544401106 | 0.00059008 | 0.004158279 | -0.549577009 |
| PRELID2 | 2.588675124 | 6.238186784 | 4.543733853 | 0.000590786 | 0.004161323 | -0.550797077 |
| HIST4H4 | -2.133886415 | 6.387445826 | -4.541398642 | 0.000593267 | 0.004173196 | -0.555067371 |
| DUSP22 | -1.441958467 | 7.221769923 | -4.541368654 | 0.000593298 | 0.004173196 | -0.555122211 |
| CHRNA9 | -1.05735895 | 2.051057239 | -4.540104123 | 0.000594646 | 0.004180734 | -0.557434847 |
| KLHL24 | 1.244724188 | 8.501038405 | 4.537715918 | 0.0005972 | 0.00419653 | -0.561802968 |
| SERTAD1 | -1.5730943 | 11.40675471 | -4.537485348 | 0.000597447 | 0.00419653 | -0.562224722 |
| CPA4 | 1.724580562 | 9.717820089 | 4.536528521 | 0.000598474 | 0.004201794 | -0.563974988 |
| SHTN1 | -1.867982623 | 3.21300608 | -4.535796072 | 0.000599261 | 0.004205373 | -0.56531488 |
| ARL15 | -1.488638155 | 5.593898706 | -4.535083071 | 0.000600028 | 0.004208808 | -0.566619248 |
| CNTRL | 1.208320281 | 7.594527591 | 4.534510166 | 0.000600645 | 0.004211188 | -0.567667362 |
| KIAA1841 | 1.472334591 | 3.153537091 | 4.534182605 | 0.000600999 | 0.004211716 | -0.568266642 |
| FDXR | 1.466519744 | 11.10941798 | 4.533374102 | 0.000601871 | 0.004211988 | -0.569745864 |
| ZNF26 | 1.290476267 | 8.450569809 | 4.532642853 | 0.000602662 | 0.00421261 | -0.571083802 |
| KPNB1 | 1.17816199 | 11.92744814 | 4.53257819 | 0.000602732 | 0.00421261 | -0.571202116 |
| TIMP4 | 1.734537364 | 2.379501292 | 4.532520419 | 0.000602794 | 0.00421261 | -0.57130782 |
| SAMD9L | -1.382697286 | 8.18827387 | -4.532170427 | 0.000603173 | 0.004213314 | -0.571948213 |
| YJEFN3 | 1.018269768 | 7.286684647 | 4.531842681 | 0.000603528 | 0.004213851 | -0.57254791 |
| PFN1 | -1.251841681 | 12.78850541 | -4.525768307 | 0.000610147 | 0.00425614 | -0.58366464 |
| HSP90AA1 | 1.118542943 | 14.44239593 | 4.524917531 | 0.00061108 | 0.004258645 | -0.585221957 |
| ZNF785 | 1.10969463 | 6.095743293 | 4.521869535 | 0.000614435 | 0.004278171 | -0.590801832 |
| DHX38 | 1.130448408 | 9.742581633 | 4.520961676 | 0.000615438 | 0.004281359 | -0.59246401 |
| DUS3L | 1.023876792 | 11.49625548 | 4.517650034 | 0.00061911 | 0.004304793 | -0.598527948 |
| KYAT3 | -1.141395225 | 7.428315766 | -4.516236494 | 0.000620685 | 0.004311784 | -0.601116626 |
| PCBP4 | -2.232510457 | 5.891903188 | -4.515845353 | 0.000621122 | 0.004312838 | -0.601832976 |
| IZUMO1R | 1.857545621 | 4.773532042 | 4.515564211 | 0.000621435 | 0.004313041 | -0.602347878 |
| SENP7 | 1.234462912 | 8.747116836 | 4.513837565 | 0.000623367 | 0.004324466 | -0.605510364 |
| CYGB | 1.371648079 | 14.52956815 | 4.51190012 | 0.000625542 | 0.004334351 | -0.609059312 |
| RAB3IL1 | 1.387093385 | 5.2436882 | 4.511382439 | 0.000626124 | 0.004334351 | -0.61000765 |
| DENND3 | 1.469127383 | 10.19905283 | 4.511367588 | 0.000626141 | 0.004334351 | -0.610034856 |
| NOD2 | -1.196537928 | 4.632212927 | -4.511295235 | 0.000626222 | 0.004334351 | -0.6101674 |
| BRCA1 | 2.095261851 | 5.767744556 | 4.510958602 | 0.000626601 | 0.004334665 | -0.610784096 |
| SAP18 | -2.195500835 | 6.051625717 | -4.510746926 | 0.00062684 | 0.004334665 | -0.611171881 |
| RBM11 | -2.447549362 | 2.89469804 | -4.510233559 | 0.000627419 | 0.004336688 | -0.612112377 |
| TRPM7 | 1.005544492 | 7.375139394 | 4.508934733 | 0.000628885 | 0.004344844 | -0.614491966 |
| KRTAP4-11 | 1.235885009 | 10.55934076 | 4.508449758 | 0.000629434 | 0.004346653 | -0.61538054 |
| CEP83 | 1.173547655 | 7.131313826 | 4.507793849 | 0.000630177 | 0.0043498 | -0.616582334 |
| JAK1 | -1.63860745 | 8.315257061 | -4.504759589 | 0.000633625 | 0.004371608 | -0.622142469 |
| MT-ND4 | 1.798903465 | 14.91189432 | 4.503262405 | 0.000635333 | 0.0043814 | -0.624886338 |
| FEM1C | 1.074725106 | 8.571724245 | 4.501605819 | 0.000637229 | 0.004392223 | -0.627922609 |
| CEP152 | 1.594627074 | 6.192479909 | 4.501385009 | 0.000637483 | 0.004392223 | -0.628327342 |
| SPTBN1 | 1.99868653 | 7.203721363 | 4.500105568 | 0.000638952 | 0.004398517 | -0.630672588 |
| ZBTB14 | -1.014512829 | 6.869892487 | -4.500083759 | 0.000638977 | 0.004398517 | -0.630712566 |
| SMIM4 | -1.200167012 | 11.4068506 | -4.499142683 | 0.000640059 | 0.004403969 | -0.63243769 |
| RSC1A1 | 1.738376056 | 7.966161599 | 4.498068242 | 0.000641298 | 0.004410488 | -0.634407402 |
| CDC25A | 1.498134316 | 6.850835219 | 4.497680368 | 0.000641746 | 0.004411565 | -0.635118499 |
| CKS2 | 2.343630989 | 10.50590911 | 4.497106462 | 0.000642409 | 0.00441412 | -0.63617068 |
| UCP2 | -1.219529357 | 11.5249659 | -4.495173272 | 0.000644648 | 0.004427495 | -0.639715181 |
| GFM2 | 1.238835577 | 6.797590538 | 4.493958324 | 0.000646059 | 0.004433184 | -0.641942982 |
| ZDHHC12 | -1.340673962 | 8.72831903 | -4.493956314 | 0.000646061 | 0.004433184 | -0.641946669 |
| RIPOR2 | -2.078239034 | 9.198701757 | -4.492972245 | 0.000647206 | 0.004439032 | -0.643751228 |
| DCTD | -1.216011585 | 8.297527056 | -4.492376019 | 0.000647901 | 0.004441788 | -0.64484462 |
| CCDC171 | 2.826697429 | 3.834336999 | 4.491658607 | 0.000648738 | 0.004445516 | -0.646160297 |
| CLEC18B | -1.476333238 | 6.954000277 | -4.491015029 | 0.00064949 | 0.004447582 | -0.647340614 |
| SLC25A23 | -1.722261532 | 6.28154094 | -4.490898222 | 0.000649627 | 0.004447582 | -0.647554842 |
| SH3BP5L | 1.920399552 | 3.493211702 | 4.490246153 | 0.00065039 | 0.004449239 | -0.648750782 |
| ZNF66 | 2.010413466 | 7.492094574 | 4.489938827 | 0.00065075 | 0.004449239 | -0.649314454 |
| APBB1 | -1.73699222 | 9.600241086 | -4.489384143 | 0.0006514 | 0.004451675 | -0.650331836 |
| CD86 | 4.957986659 | 10.41308272 | 4.48866148 | 0.000652248 | 0.004455461 | -0.651657366 |
| PNPLA1 | -1.207844287 | 2.968396591 | -4.486796295 | 0.000654442 | 0.004468435 | -0.65507879 |
| TOP2A | 3.438923518 | 6.528546042 | 4.486228145 | 0.000655112 | 0.004470994 | -0.656121054 |
| RABGAP1L | -1.330120497 | 8.792391756 | -4.484966537 | 0.000656602 | 0.004473712 | -0.658435574 |
| SLC35G5 | 1.171562111 | 4.400688845 | 4.484871651 | 0.000656715 | 0.004473712 | -0.658609658 |
| TMEM179B | -1.093010351 | 9.396049017 | -4.484391973 | 0.000657282 | 0.004473712 | -0.659489712 |
| TTC22 | -1.152441362 | 6.373802645 | -4.481022528 | 0.000661283 | 0.004498924 | -0.665672225 |
| RRN3 | 1.12294409 | 7.69571046 | 4.480030605 | 0.000662466 | 0.004502926 | -0.6674925 |
| ERCC6 | -1.435128403 | 6.262349073 | -4.47948055 | 0.000663123 | 0.004505369 | -0.668501948 |
| CREB5 | -2.094462095 | 6.974247871 | -4.479184136 | 0.000663477 | 0.004505755 | -0.669045932 |
| REEP5 | -1.484278397 | 10.78362711 | -4.477898825 | 0.000665015 | 0.004514178 | -0.671404864 |
| DGKQ | -1.011372294 | 10.66756681 | -4.476812292 | 0.000666318 | 0.004520999 | -0.67339911 |
| MT-ND4L | 1.70293477 | 15.59723784 | 4.476550799 | 0.000666632 | 0.004521105 | -0.673879078 |
| VAX2 | 1.358710335 | 8.362109082 | 4.476165878 | 0.000667095 | 0.004522218 | -0.674585609 |
| TRNP1 | -1.269710258 | 3.991953296 | -4.474774749 | 0.000668769 | 0.004531541 | -0.677139183 |
| CBLL2 | 1.001162845 | 3.711395283 | 4.472784416 | 0.000671173 | 0.004545792 | -0.680793005 |
| KIAA1211L | -1.099418057 | 4.007901646 | -4.471833196 | 0.000672324 | 0.004551558 | -0.682539383 |
| AIF1 | -2.028859087 | 15.3186195 | -4.469847033 | 0.000674736 | 0.004564517 | -0.686186143 |
| DHX30 | 1.163817044 | 9.39278758 | 4.468142945 | 0.000676812 | 0.004573763 | -0.689315308 |
| KIT | 5.057869458 | 5.490646052 | 4.466304753 | 0.000679059 | 0.004586901 | -0.692691054 |
| ZFP3 | -1.202505439 | 6.883895773 | -4.46459438 | 0.000681156 | 0.00459902 | -0.695832373 |
| POU5F2 | 1.405556532 | 5.211166189 | 4.461665825 | 0.000684764 | 0.004619258 | -0.701211727 |
| MED26 | 1.196841291 | 8.556372643 | 4.460647177 | 0.000686023 | 0.004624827 | -0.703083046 |
| ZNF236 | 1.183089012 | 6.037468209 | 4.46041401 | 0.000686312 | 0.004624827 | -0.703511404 |
| SHCBP1 | 2.455500906 | 5.189037857 | 4.460257827 | 0.000686505 | 0.004624827 | -0.703798334 |
| LDHA | 1.617414596 | 7.203468617 | 4.459799149 | 0.000687073 | 0.004626598 | -0.704641006 |
| MICB | -1.309592567 | 10.25874603 | -4.457914552 | 0.000689413 | 0.004640292 | -0.708103564 |
| ACSS1 | 1.073320531 | 9.689205273 | 4.457515411 | 0.00068991 | 0.004640388 | -0.708836949 |
| NAP1L4 | 1.944361856 | 7.53023939 | 4.457164999 | 0.000690346 | 0.004640388 | -0.709480811 |
| PANK3 | 1.113800335 | 8.63089725 | 4.454935365 | 0.000693129 | 0.004657028 | -0.713577931 |
| JTB | 1.524608567 | 9.123837352 | 4.453731081 | 0.000694637 | 0.004665091 | -0.715791102 |
| ZNF440 | 2.630418631 | 4.539533129 | 4.453197503 | 0.000695306 | 0.004665449 | -0.716771731 |
| ABL2 | 1.184329198 | 4.799825589 | 4.450465991 | 0.000698743 | 0.00468598 | -0.721792245 |
| SPRR2F | 1.277228387 | 8.060469531 | 4.449690681 | 0.000699721 | 0.004688843 | -0.7232174 |
| KMT2C | 1.183571466 | 8.648409347 | 4.445665506 | 0.000704825 | 0.004718867 | -0.730617338 |
| WASF1 | 1.825141779 | 8.138951563 | 4.445410746 | 0.000705149 | 0.004718953 | -0.731085746 |
| OAF | -1.977541247 | 6.869241662 | -4.444629139 | 0.000706145 | 0.004723366 | -0.73252287 |
| MEMO1 | 1.733041062 | 4.665459903 | 4.444404088 | 0.000706432 | 0.004723366 | -0.73293668 |
| CLEC20A | 1.569187063 | 11.93865252 | 4.443298456 | 0.000707844 | 0.004730718 | -0.734969711 |
| RAB20 | -2.194538233 | 7.092104684 | -4.44166471 | 0.000709935 | 0.004742605 | -0.737974059 |
| TRIM54 | -1.542451651 | 8.027078748 | -4.441305337 | 0.000710396 | 0.004743593 | -0.738634957 |
| FCRL1 | -1.295242619 | 7.084246939 | -4.44069594 | 0.000711179 | 0.004746037 | -0.739755686 |
| BICD1 | 1.546405375 | 3.959042579 | 4.440532369 | 0.000711389 | 0.004746037 | -0.740056513 |
| ALOX5AP | -3.055559441 | 10.46635551 | -4.439097563 | 0.000713235 | 0.004754166 | -0.742695404 |
| TNF | -2.811283212 | 7.503849355 | -4.438073246 | 0.000714556 | 0.004760877 | -0.744579452 |
| PKN2 | 1.349829652 | 7.077591749 | 4.437133126 | 0.00071577 | 0.004764259 | -0.746308724 |
| CCDC158 | 1.066222318 | 4.359204617 | 4.436137868 | 0.000717059 | 0.004769164 | -0.748139513 |
| CETN2 | -1.407762359 | 9.189337754 | -4.435185696 | 0.000718293 | 0.004771558 | -0.749891139 |
| METTL8 | 1.259330748 | 5.861813675 | 4.435131656 | 0.000718363 | 0.004771558 | -0.749990554 |
| CABLES1 | 1.239418647 | 7.672900767 | 4.433319821 | 0.000720719 | 0.004783012 | -0.75332388 |
| HLA-E | -1.710596057 | 12.14954281 | -4.432745811 | 0.000721467 | 0.00478588 | -0.754379986 |
| OR10G3 | 1.153787053 | 5.896443598 | 4.429504224 | 0.000725706 | 0.004809789 | -0.760344696 |
| SLC4A3 | -1.550340986 | 4.402437153 | -4.428270745 | 0.000727326 | 0.004818418 | -0.762614642 |
| LEPROTL1 | 1.304246215 | 9.961129596 | 4.427607242 | 0.000728199 | 0.004822092 | -0.763835735 |
| ITM2B | -1.238869055 | 13.25933799 | -4.42539511 | 0.000731117 | 0.004835076 | -0.767907197 |
| FAS | -1.633433869 | 8.390101668 | -4.424555375 | 0.000732228 | 0.00484031 | -0.769452869 |
| TMEM37 | 1.245570237 | 6.360839499 | 4.422558733 | 0.000734877 | 0.004855699 | -0.773128296 |
| CDK16 | 1.309124923 | 12.04709954 | 4.420104661 | 0.000738145 | 0.004875171 | -0.7776463 |
| NET1 | 1.731741059 | 7.695937216 | 4.417272526 | 0.000741937 | 0.004895941 | -0.782861062 |
| PDGFD | -2.034262137 | 4.177428113 | -4.414283044 | 0.00074596 | 0.00491709 | -0.788366394 |
| C9orf24 | -1.382480869 | 6.874686412 | -4.414168252 | 0.000746115 | 0.00491709 | -0.78857781 |
| ABCG8 | 1.433733164 | 3.532776821 | 4.41310192 | 0.000747556 | 0.004922305 | -0.790541755 |
| PPM1H | 2.862440309 | 3.303253124 | 4.412534584 | 0.000748324 | 0.00492522 | -0.791586708 |
| ZNF277 | -1.171338828 | 9.284212288 | -4.410617868 | 0.000750924 | 0.004940187 | -0.795117256 |
| ITGB1BP1 | -1.706914034 | 5.932429595 | -4.408398336 | 0.000753947 | 0.00495792 | -0.799206032 |
| BORCS7 | -1.409182179 | 10.26016809 | -4.407504277 | 0.000755168 | 0.004962444 | -0.800853185 |
| MTFR1L | 1.024200847 | 5.365933662 | 4.406400787 | 0.000756678 | 0.004967641 | -0.802886286 |
| CYP2U1 | -2.725888807 | 4.647850766 | -4.406358013 | 0.000756736 | 0.004967641 | -0.802965096 |
| WNK1 | 1.111970287 | 6.818870863 | 4.405863329 | 0.000757414 | 0.004969938 | -0.803876557 |
| SPATA24 | -2.015442885 | 5.420718803 | -4.400598524 | 0.000764669 | 0.005011433 | -0.813578484 |
| GFOD2 | 1.285728417 | 6.020471888 | 4.400529739 | 0.000764764 | 0.005011433 | -0.813705257 |
| CLTC | 1.64188911 | 4.975711256 | 4.400315405 | 0.000765061 | 0.005011433 | -0.814100289 |
| HAUS6 | 1.038618731 | 6.073384001 | 4.399899129 | 0.000765638 | 0.005012216 | -0.814867524 |
| DNAJB9 | -1.185599368 | 10.4366097 | -4.399751872 | 0.000765843 | 0.005012216 | -0.815138936 |
| ATMIN | -1.391918587 | 7.473579544 | -4.399366488 | 0.000766377 | 0.00501355 | -0.815849255 |
| NSUN6 | 1.215437103 | 7.20835225 | 4.398376874 | 0.000767752 | 0.005020377 | -0.817673322 |
| ANTXR2 | 1.80904917 | 7.159234923 | 4.397827231 | 0.000768517 | 0.005023209 | -0.818686472 |
| KIAA1958 | 1.174784068 | 8.031247631 | 4.396587821 | 0.000770244 | 0.005032328 | -0.820971167 |
| LILRB4 | -2.167589002 | 8.010349118 | -4.395145096 | 0.00077226 | 0.005043323 | -0.823630833 |
| ZNF804A | 1.413842989 | 2.375224807 | 4.394128706 | 0.000773683 | 0.005050441 | -0.82550467 |
| TAS2R46 | 2.659275691 | 4.107157617 | 4.391974187 | 0.000776709 | 0.005065829 | -0.829477114 |
| CDC14B | -1.04136782 | 5.074144459 | -4.391718853 | 0.000777069 | 0.005065993 | -0.829947921 |
| MON2 | 1.412646168 | 6.440797198 | 4.391070933 | 0.000777981 | 0.005069072 | -0.831142642 |
| HIST3H2A | -1.982800827 | 4.921118307 | -4.390908756 | 0.00077821 | 0.005069072 | -0.831441692 |
| ZFP36L2 | 1.27747255 | 12.74866576 | 4.390632893 | 0.000778599 | 0.005069427 | -0.831950381 |
| NPIPB8 | 1.354450447 | 10.13325893 | 4.388122656 | 0.000782149 | 0.005090354 | -0.836579575 |
| KIR3DL3 | 1.029481647 | 5.339601564 | 4.387317821 | 0.000783291 | 0.005095597 | -0.83806392 |
| KIFC1 | 4.085948144 | 5.718552117 | 4.386782201 | 0.000784052 | 0.005097193 | -0.839051791 |
| TRPM3 | 1.012466198 | 3.926072945 | 4.386287416 | 0.000784755 | 0.005097193 | -0.839964371 |
| FLOT1 | -1.800150582 | 10.74238097 | -4.386141107 | 0.000784964 | 0.005097193 | -0.840234229 |
| TUBA4B | -1.761136557 | 9.045828209 | -4.385981275 | 0.000785191 | 0.005097193 | -0.840529029 |
| ZMAT5 | -1.709813574 | 8.577456308 | -4.385961863 | 0.000785219 | 0.005097193 | -0.840564835 |
| TCEAL8 | -1.492520284 | 9.619967655 | -4.385065183 | 0.000786496 | 0.005103298 | -0.842218757 |
| TRAM1 | -1.374714265 | 9.229405963 | -4.384524301 | 0.000787268 | 0.005106118 | -0.84321645 |
| ATG2B | 1.342976776 | 6.116748149 | 4.383606421 | 0.000788579 | 0.005112433 | -0.844909605 |
| DOCK4 | 1.988768735 | 3.470284342 | 4.381631567 | 0.000791407 | 0.005128577 | -0.848552764 |
| HNRNPA0 | 1.15885066 | 5.579386276 | 4.380626406 | 0.000792851 | 0.005135736 | -0.850407201 |
| CRISPLD1 | 1.678319173 | 3.490145124 | 4.377545743 | 0.000797293 | 0.005162301 | -0.856091364 |
| UNC93B1 | -1.083113278 | 9.005097814 | -4.377180395 | 0.000797821 | 0.005163516 | -0.856765532 |
| NUPL2 | 1.508612142 | 9.900158841 | 4.376598837 | 0.000798663 | 0.00516619 | -0.857838693 |
| MBD6 | 1.165737744 | 8.804020945 | 4.376424101 | 0.000798916 | 0.00516619 | -0.858161144 |
| FBLN5 | -4.010663893 | 6.693625005 | -4.37072189 | 0.000807224 | 0.005213234 | -0.868685344 |
| FAM198B | -4.406320558 | 7.388447391 | -4.369814875 | 0.000808553 | 0.005219596 | -0.870359647 |
| EVPL | 1.033601403 | 11.92383234 | 4.369345599 | 0.000809242 | 0.005221818 | -0.871225938 |
| PCLAF | 3.113140308 | 11.11141919 | 4.368066242 | 0.000811123 | 0.005229501 | -0.873587754 |
| YLPM1 | 1.786856645 | 4.527874967 | 4.365636897 | 0.000814707 | 0.005248142 | -0.878072984 |
| ZBTB40 | 1.091334431 | 8.198875678 | 4.363651218 | 0.000817649 | 0.005264854 | -0.881739501 |
| GOLGA8R | 3.93392918 | 5.237399237 | 4.362929602 | 0.000818721 | 0.005269515 | -0.883072042 |
| PRR34 | -1.837706287 | 3.299395525 | -4.360326829 | 0.000822599 | 0.005292226 | -0.887878749 |
| PTPN12 | -1.51947354 | 10.8363528 | -4.358442768 | 0.000825418 | 0.005308108 | -0.891358563 |
| NCAM1 | -2.814885414 | 4.095345802 | -4.357334818 | 0.00082708 | 0.005314287 | -0.893405075 |
| HLA-DRA | -1.614090429 | 13.8400615 | -4.356973787 | 0.000827622 | 0.005315518 | -0.894071966 |
| ARHGEF37 | 1.283376684 | 7.188192902 | 4.353071046 | 0.00083351 | 0.005351062 | -0.901281827 |
| ANKRD34B | -2.221170213 | 2.687810197 | -4.352279319 | 0.000834709 | 0.005356494 | -0.902744625 |
| ARHGEF33 | 1.323137546 | 3.128646469 | 4.351009694 | 0.000836637 | 0.005364319 | -0.905090511 |
| JHY | -1.549765219 | 3.640869357 | -4.347725277 | 0.000841644 | 0.005389584 | -0.911159825 |
| ZKSCAN3 | 1.053112576 | 6.086688985 | 4.346825796 | 0.000843021 | 0.00539612 | -0.912822163 |
| COA6 | -1.064069819 | 10.60425406 | -4.346359935 | 0.000843735 | 0.00539617 | -0.913683152 |
| THBS3 | 1.365845421 | 10.64364611 | 4.346349116 | 0.000843752 | 0.00539617 | -0.913703148 |
| CRISPLD2 | -2.216983266 | 5.258098784 | -4.346123624 | 0.000844098 | 0.00539617 | -0.914119903 |
| CISD1 | -2.039673122 | 3.320359469 | -4.344556764 | 0.000846505 | 0.005406304 | -0.917015911 |
| DNASE1L1 | -1.084567484 | 7.941765398 | -4.34439521 | 0.000846753 | 0.005406304 | -0.917314522 |
| C5AR1 | -4.530873067 | 8.038747173 | -4.343847663 | 0.000847596 | 0.005409407 | -0.918326608 |
| RFX8 | 3.28636297 | 6.431598839 | 4.342352662 | 0.000849903 | 0.005421843 | -0.92109011 |
| GPR18 | -2.596907124 | 7.736620394 | -4.339105236 | 0.000854935 | 0.005444631 | -0.927093673 |
| ADAD1 | 1.108234126 | 5.162745906 | 4.338873094 | 0.000855296 | 0.005444631 | -0.927522875 |
| PKIG | 1.104415178 | 9.041981352 | 4.338657584 | 0.000855631 | 0.005444631 | -0.927921332 |
| SLC29A2 | 1.057821463 | 5.630524399 | 4.338354005 | 0.000856104 | 0.005445351 | -0.928482624 |
| CD40LG | -2.679814099 | 8.623577331 | -4.337753009 | 0.00085704 | 0.005449017 | -0.929593844 |
| DAND5 | 1.993693023 | 3.893573106 | 4.335398125 | 0.000860717 | 0.005467811 | -0.933948256 |
| SRSF3 | 1.125400959 | 8.592891422 | 4.334301229 | 0.000862436 | 0.005476433 | -0.9359767 |
| USB1 | -1.054617274 | 7.518162369 | -4.333552096 | 0.000863612 | 0.005480875 | -0.937362103 |
| ZNF385D | 2.875544301 | 5.052946954 | 4.333394723 | 0.000863859 | 0.005480875 | -0.937653148 |
| RLN1 | -1.783045221 | 4.773116097 | -4.33128473 | 0.000867181 | 0.005498 | -0.941555555 |
| EHBP1 | 1.528858989 | 7.752687339 | 4.33121926 | 0.000867284 | 0.005498 | -0.941676648 |
| NDFIP1 | -2.030411753 | 9.049199804 | -4.330105508 | 0.000869043 | 0.005504544 | -0.943736693 |
| CEP68 | 1.128027653 | 6.743358472 | 4.329449646 | 0.00087008 | 0.005508813 | -0.944949856 |
| DLG1 | 1.46934206 | 7.708067413 | 4.328624143 | 0.000871388 | 0.005512654 | -0.946476865 |
| NAP1L5 | -1.482889715 | 7.14026692 | -4.328607264 | 0.000871415 | 0.005512654 | -0.946508088 |
| TBC1D8B | 1.184687871 | 6.150762279 | 4.327794478 | 0.000872704 | 0.005518508 | -0.948011636 |
| MACO1 | 1.102067141 | 7.717297049 | 4.326096832 | 0.000875404 | 0.005530963 | -0.951152254 |
| RBM5 | 1.410562151 | 12.69257897 | 4.325566503 | 0.000876249 | 0.005533464 | -0.952133408 |
| ZNF598 | 1.359792028 | 8.991167804 | 4.324762631 | 0.000877532 | 0.00553748 | -0.95362069 |
| C1orf131 | -1.446793418 | 7.865268285 | -4.323327695 | 0.000879827 | 0.005549647 | -0.956275679 |
| TEX30 | 1.25169875 | 9.5088658 | 4.322480057 | 0.000881185 | 0.005555902 | -0.957844109 |
| TMEM173 | 1.024399698 | 12.84097157 | 4.321922455 | 0.00088208 | 0.00555923 | -0.958875907 |
| STARD5 | -1.608943348 | 7.485944375 | -4.320815748 | 0.000883858 | 0.005565809 | -0.96092386 |
| TPGS1 | -1.518556288 | 7.411108907 | -4.317731435 | 0.000888835 | 0.005592498 | -0.96663195 |
| EIF4A3 | 1.111500381 | 11.93336189 | 4.31434004 | 0.00089434 | 0.005617942 | -0.972909351 |
| IHH | 1.411407192 | 4.870732792 | 4.314258213 | 0.000894473 | 0.005617942 | -0.973060823 |
| CDK12 | 1.160088149 | 6.148776919 | 4.314099058 | 0.000894733 | 0.005617942 | -0.973355443 |
| RUFY2 | 1.131856818 | 7.223606629 | 4.312224644 | 0.000897792 | 0.005633705 | -0.976825433 |
| RECQL5 | 1.07556834 | 5.774225278 | 4.312105475 | 0.000897987 | 0.005633705 | -0.977046054 |
| CEP350 | 1.177776772 | 8.371690664 | 4.310980002 | 0.000899829 | 0.005640593 | -0.979129738 |
| LYPD6B | -1.585699138 | 2.798399654 | -4.309516797 | 0.000902231 | 0.005653307 | -0.981838867 |
| PPP1R14A | -1.767445757 | 9.964841008 | -4.309156009 | 0.000902824 | 0.005654483 | -0.982506895 |
| AP1G1 | 1.357111512 | 9.410266212 | 4.308852176 | 0.000903324 | 0.005654483 | -0.983069478 |
| DBX1 | -1.545679231 | 3.310458305 | -4.308721897 | 0.000903538 | 0.005654483 | -0.983310707 |
| ZNF518A | 1.348774134 | 6.316119288 | 4.30716208 | 0.000906109 | 0.005665892 | -0.986199038 |
| GNAQ | 2.032613125 | 7.83586358 | 4.30485855 | 0.00090992 | 0.005685028 | -0.99046491 |
| PASK | -1.877532807 | 7.36171578 | -4.304484552 | 0.00091054 | 0.005686558 | -0.991157557 |
| NDUFB7 | -1.315909409 | 11.64317022 | -4.303134421 | 0.000912783 | 0.005693524 | -0.993658112 |
| TRPC1 | -2.207566751 | 3.447557798 | -4.302266089 | 0.000914228 | 0.00569409 | -0.995266422 |
| TMEM50A | -1.249770713 | 10.09697086 | -4.302179906 | 0.000914372 | 0.00569409 | -0.995426052 |
| OR1L4 | 1.739418867 | 2.911915127 | 4.302016685 | 0.000914644 | 0.00569409 | -0.995728376 |
| SPDYE1 | 1.510621891 | 2.544537311 | 4.301951412 | 0.000914753 | 0.00569409 | -0.995849277 |
| RNF113B | -1.526717145 | 5.504758918 | -4.300449192 | 0.00091726 | 0.005707355 | -0.998631865 |
| SYTL5 | 1.160913043 | 4.780237289 | 4.299318743 | 0.000919152 | 0.005716164 | -1.000725949 |
| LIPT2 | -1.267063527 | 7.800818516 | -4.299152574 | 0.00091943 | 0.005716164 | -1.001033777 |
| RAB39A | -1.490326977 | 2.272995204 | -4.298585576 | 0.000920381 | 0.005719727 | -1.002084155 |
| CEP57L1 | 1.224153043 | 6.579637168 | 4.297943262 | 0.000921459 | 0.00572408 | -1.003274093 |
| SMC2 | 1.957561683 | 6.991027487 | 4.29334316 | 0.000929219 | 0.005766225 | -1.011797229 |
| KRTAP19-5 | 1.548002627 | 6.456283982 | 4.293076634 | 0.000929671 | 0.005766225 | -1.01229111 |
| PLIN4 | -1.556296811 | 4.71105967 | -4.293020441 | 0.000929766 | 0.005766225 | -1.012395237 |
| TMEM59 | -1.014557741 | 9.220659433 | -4.289236604 | 0.000936204 | 0.0058014 | -1.019407507 |
| ERMP1 | 1.752259315 | 7.060971516 | 4.288774806 | 0.000936993 | 0.005801816 | -1.020263407 |
| RASGRP1 | -2.71994012 | 8.652339278 | -4.288749036 | 0.000937037 | 0.005801816 | -1.02031117 |
| CAV3 | 1.566461058 | 3.476398198 | 4.28741993 | 0.000939311 | 0.005813521 | -1.02277465 |
| CSAD | 1.593387095 | 7.490803404 | 4.287085407 | 0.000939884 | 0.005814694 | -1.023394708 |
| PLOD1 | -1.02156232 | 5.208983222 | -4.286789291 | 0.000940392 | 0.005815461 | -1.023943586 |
| BACH1 | -1.091553159 | 5.938536221 | -4.285604267 | 0.000942427 | 0.005823689 | -1.02614021 |
| EP400 | 1.184291154 | 7.01881975 | 4.285566831 | 0.000942491 | 0.005823689 | -1.026209606 |
| DPPA5 | 1.579044761 | 3.784216488 | 4.284849223 | 0.000943726 | 0.005828941 | -1.027539866 |
| SYNGR2 | -1.099013852 | 8.919462684 | -4.284131473 | 0.000944962 | 0.0058342 | -1.028870434 |
| UPF2 | 1.063534658 | 7.999985262 | 4.283603183 | 0.000945874 | 0.005837447 | -1.029849811 |
| OR5AS1 | 1.268692475 | 2.93769453 | 4.28276324 | 0.000947324 | 0.005844019 | -1.031406998 |
| DDX11 | 1.411797611 | 7.949991659 | 4.281763608 | 0.000949054 | 0.005852304 | -1.033260318 |
| TPPP3 | -3.334548573 | 6.971504611 | -4.279965129 | 0.000952173 | 0.005869152 | -1.036594922 |
| PJVK | 2.610520397 | 4.787766999 | 4.279417452 | 0.000953125 | 0.005872632 | -1.037610441 |
| IRAK4 | -1.128701743 | 7.121069033 | -4.279147659 | 0.000953595 | 0.005873135 | -1.038110709 |
| PPRC1 | 1.136542 | 9.790148713 | 4.277718739 | 0.000956085 | 0.005886078 | -1.040760411 |
| RIDA | -1.291194024 | 8.39591102 | -4.277285079 | 0.000956842 | 0.005888346 | -1.041564598 |
| ETV3 | 1.036460661 | 6.323345816 | 4.277008675 | 0.000957324 | 0.005888925 | -1.042077176 |
| GOLGA6L4 | 1.540115697 | 4.854242786 | 4.275175984 | 0.000960532 | 0.005903465 | -1.045475985 |
| TLR1 | -1.714004521 | 9.784087265 | -4.274990705 | 0.000960857 | 0.005903465 | -1.045819609 |
| GNAL | 1.329665705 | 6.634942121 | 4.272272587 | 0.000965637 | 0.005928104 | -1.050861067 |
| HSPA6 | -2.495684288 | 8.769927723 | -4.272158503 | 0.000965838 | 0.005928104 | -1.05107268 |
| HLF | -1.884470923 | 2.618431635 | -4.27204317 | 0.000966041 | 0.005928104 | -1.051286609 |
| THSD1 | 1.341108012 | 5.784854879 | 4.271547377 | 0.000966916 | 0.005931071 | -1.052206266 |
| SGMS2 | -2.692644754 | 4.65697413 | -4.270500521 | 0.000968766 | 0.005940013 | -1.054148173 |
| CHRNB3 | -1.517667369 | 5.65992256 | -4.269822557 | 0.000969966 | 0.005944965 | -1.055405839 |
| RYR1 | -2.866785481 | 5.142944343 | -4.269363179 | 0.00097078 | 0.00594709 | -1.056258038 |
| KLK1 | -3.096617473 | 5.741750115 | -4.26918391 | 0.000971098 | 0.00594709 | -1.056590607 |
| SCN4A | 1.011299899 | 5.25035179 | 4.268772385 | 0.000971828 | 0.005949156 | -1.057354055 |
| S100PBP | 1.429540246 | 9.566390173 | 4.268360235 | 0.000972559 | 0.00595123 | -1.058118676 |
| ZNF254 | 1.144960379 | 8.309430991 | 4.268038629 | 0.000973131 | 0.005952323 | -1.058715331 |
| CXCL12 | 2.153213267 | 5.385134252 | 4.265869754 | 0.000976993 | 0.005973534 | -1.06273934 |
| BIRC3 | -3.051762884 | 9.513747061 | -4.265500411 | 0.000977652 | 0.005975154 | -1.063424639 |
| MTREX | 1.176403898 | 10.60482732 | 4.262069385 | 0.000983798 | 0.006007871 | -1.069791314 |
| SHKBP1 | -1.216767163 | 9.549479845 | -4.261388999 | 0.000985022 | 0.00601292 | -1.071053972 |
| C2orf16 | 1.032910299 | 3.248116305 | 4.260580611 | 0.000986478 | 0.006017574 | -1.072554229 |
| TMPRSS3 | -1.383730317 | 3.937773859 | -4.260417348 | 0.000986772 | 0.006017574 | -1.072857229 |
| AIP | -1.061503418 | 11.62692014 | -4.260304287 | 0.000986976 | 0.006017574 | -1.073067061 |
| P2RY11 | 1.29562591 | 6.551611491 | 4.259478987 | 0.000988465 | 0.00602423 | -1.074598782 |
| CTSO | -1.375361599 | 9.805731662 | -4.257788408 | 0.000991523 | 0.006035584 | -1.077736606 |
| KCTD21 | -1.487935566 | 5.804073772 | -4.257560434 | 0.000991936 | 0.006035674 | -1.078159761 |
| ADAM29 | 1.451219946 | 3.589132025 | 4.256998559 | 0.000992955 | 0.006039449 | -1.079202702 |
| KLRG2 | 1.209910292 | 6.985763166 | 4.256667167 | 0.000993557 | 0.006040682 | -1.079817838 |
| KRT72 | -1.743769694 | 6.976132982 | -4.25609029 | 0.000994605 | 0.00604409 | -1.080888672 |
| MADD | 1.077030417 | 10.73349296 | 4.255919534 | 0.000994915 | 0.00604409 | -1.081205645 |
| GPR34 | -1.486994491 | 5.908313051 | -4.253433236 | 0.000999446 | 0.00606422 | -1.085821224 |
| C17orf100 | -1.160695307 | 4.162398285 | -4.253223406 | 0.00099983 | 0.00606422 | -1.086210777 |
| PAQR3 | 1.065206468 | 7.763361484 | 4.252521132 | 0.001001114 | 0.006069579 | -1.087514592 |
| ADAM21 | 1.054788787 | 4.02561752 | 4.248862676 | 0.001007832 | 0.006100543 | -1.094307425 |
| SCML2 | 1.193109116 | 9.438784075 | 4.248399262 | 0.001008686 | 0.006103276 | -1.095167951 |
| CTSE | 2.622254252 | 5.921476798 | 4.245001107 | 0.001014973 | 0.006133967 | -1.101478631 |
| RHOF | -1.142638651 | 7.485330908 | -4.244173372 | 0.001016511 | 0.006138693 | -1.103015958 |
| MLC1 | 2.135541402 | 10.76188063 | 4.242359931 | 0.001019887 | 0.006156301 | -1.106384212 |
| SRGAP3 | 2.544316558 | 2.783651691 | 4.241798453 | 0.001020935 | 0.006158243 | -1.10742715 |
| NDP | 1.344062307 | 6.451986785 | 4.240802199 | 0.001022797 | 0.006164909 | -1.109277741 |
| APCDD1 | -2.951711338 | 4.237789431 | -4.240725388 | 0.001022941 | 0.006164909 | -1.109420425 |
| NIPSNAP3B | 2.98514551 | 4.473921424 | 4.240244126 | 0.001023842 | 0.006167885 | -1.110314428 |
| HTR2B | 1.014850205 | 6.08265283 | 4.238966284 | 0.001026238 | 0.006179862 | -1.112688267 |
| GTF2B | -1.023091941 | 9.725668486 | -4.237160759 | 0.001029633 | 0.006195384 | -1.116042616 |
| HMOX1 | -3.277669701 | 10.33209049 | -4.236246064 | 0.001031358 | 0.006203297 | -1.117742066 |
| CD7 | -2.330890575 | 9.120770314 | -4.23498976 | 0.001033731 | 0.006215106 | -1.120076319 |
| GPR50 | 1.046738405 | 5.299021642 | 4.232253319 | 0.001038921 | 0.006239966 | -1.125161176 |
| PROSER1 | 1.39529458 | 9.588200961 | 4.232158083 | 0.001039102 | 0.006239966 | -1.125338155 |
| P2RY6 | -1.752105427 | 5.663467071 | -4.23188526 | 0.001039621 | 0.006240609 | -1.125845152 |
| ANGPTL4 | 3.509392402 | 3.707563302 | 4.230878868 | 0.001041538 | 0.006249638 | -1.12771542 |
| MVB12A | -1.19283612 | 8.265039803 | -4.229977158 | 0.001043258 | 0.006256187 | -1.12939122 |
| TTC39B | -2.741182426 | 5.567006672 | -4.229466617 | 0.001044233 | 0.006256187 | -1.130340075 |
| EDA2R | 2.001119211 | 4.142232366 | 4.229441794 | 0.001044281 | 0.006256187 | -1.13038621 |
| TPBG | 2.283230662 | 3.301419552 | 4.226637813 | 0.001049655 | 0.00628093 | -1.135597889 |
| MAML1 | 1.06741101 | 8.438905119 | 4.22370342 | 0.001055309 | 0.006312272 | -1.141052663 |
| ZNF622 | -1.257633547 | 10.10114466 | -4.223305684 | 0.001056078 | 0.006313502 | -1.141792074 |
| ABHD14B | -1.302693704 | 8.684971849 | -4.223165948 | 0.001056348 | 0.006313502 | -1.142051855 |
| ATP2A2 | 1.09685762 | 8.316831523 | 4.222254179 | 0.001058113 | 0.006321558 | -1.143746938 |
| FPR3 | -1.517105953 | 6.693185656 | -4.220121389 | 0.001062254 | 0.006343364 | -1.147712315 |
| GOLGA8S | 3.134473712 | 8.074891076 | 4.219795155 | 0.001062889 | 0.006343364 | -1.148318898 |
| CPPED1 | -2.432614441 | 9.762591747 | -4.219725969 | 0.001063023 | 0.006343364 | -1.148447539 |
| IFI27L2 | -2.145280093 | 12.89803521 | -4.219513238 | 0.001063438 | 0.006343364 | -1.148843087 |
| DIDO1 | 1.027286002 | 7.948985378 | 4.21905607 | 0.001064328 | 0.006346179 | -1.149693146 |
| IQCF2 | 1.200889121 | 4.793865174 | 4.218700331 | 0.001065022 | 0.00634683 | -1.15035462 |
| MYO1G | -1.258564883 | 11.18601441 | -4.218570451 | 0.001065276 | 0.00634683 | -1.150596126 |
| PDE4D | -1.527681157 | 6.784414944 | -4.217728386 | 0.00106692 | 0.006353843 | -1.152161942 |
| ST6GAL2 | -1.32668392 | 2.357059242 | -4.21724426 | 0.001067866 | 0.00635477 | -1.153062201 |
| QARS | 1.118344413 | 13.52760793 | 4.216578756 | 0.001069169 | 0.006358673 | -1.154299772 |
| COQ4 | 1.161339022 | 5.328840141 | 4.215063485 | 0.001072141 | 0.006370198 | -1.157117705 |
| CCDC18 | 1.134631586 | 7.722161859 | 4.214434854 | 0.001073376 | 0.006375039 | -1.158286821 |
| ATRX | 1.266741976 | 9.180474625 | 4.211355175 | 0.00107945 | 0.0064086 | -1.164014816 |
| INVS | 1.226510286 | 7.136240169 | 4.21111159 | 0.001079932 | 0.006408949 | -1.1644679 |
| PLLP | -1.096747328 | 7.856178111 | -4.210619836 | 0.001080905 | 0.006412215 | -1.165382612 |
| PRXL2C | -1.184759987 | 8.413332731 | -4.20887563 | 0.001084366 | 0.006430226 | -1.16862717 |
| GOLGA5 | -1.320989054 | 7.810732467 | -4.208452894 | 0.001085206 | 0.0064318 | -1.16941358 |
| ERC2 | -1.628042267 | 2.427483261 | -4.207197262 | 0.001087706 | 0.006439953 | -1.171749495 |
| ARHGEF7 | 1.64165234 | 7.166064754 | 4.201966015 | 0.001098187 | 0.006496839 | -1.181482842 |
| CNKSR3 | 2.547619476 | 3.44300343 | 4.199749711 | 0.001102659 | 0.006515509 | -1.185607209 |
| ITGB3 | -4.942602792 | 7.910279618 | -4.199268204 | 0.001103633 | 0.006516421 | -1.18650331 |
| GRIPAP1 | 1.054834437 | 9.45262735 | 4.198069972 | 0.00110606 | 0.006525669 | -1.18873334 |
| SYTL2 | -1.856147596 | 5.336095792 | -4.197512022 | 0.001107192 | 0.006529807 | -1.189771782 |
| HIPK1 | 1.287867557 | 9.309591747 | 4.197015129 | 0.001108202 | 0.006533216 | -1.190696606 |
| GCSAM | -1.688270232 | 6.169546062 | -4.195725125 | 0.001110827 | 0.006543599 | -1.193097675 |
| VSIR | -2.052309627 | 12.44333419 | -4.19466842 | 0.001112982 | 0.006551808 | -1.195064608 |
| ST18 | 1.30716359 | 3.865992161 | 4.194467065 | 0.001113393 | 0.006551808 | -1.195439416 |
| ITGAL | -1.561636728 | 10.76782364 | -4.194405973 | 0.001113518 | 0.006551808 | -1.195553135 |
| MKS1 | 1.020146777 | 7.280921736 | 4.193914393 | 0.001114522 | 0.006555172 | -1.196468196 |
| PHTF1 | 1.58573305 | 7.106975865 | 4.192997799 | 0.001116398 | 0.006563655 | -1.198174457 |
| SCARB1 | 1.483240068 | 11.68568239 | 4.191645463 | 0.00111917 | 0.006573348 | -1.200691988 |
| VWCE | 1.642697278 | 8.403111905 | 4.191631039 | 0.0011192 | 0.006573348 | -1.200718842 |
| HM13 | 1.55119589 | 7.437827743 | 4.191404258 | 0.001119666 | 0.006573348 | -1.201141035 |
| AKT3 | -2.134918832 | 6.366377542 | -4.191347864 | 0.001119782 | 0.006573348 | -1.201246023 |
| PDZD8 | 2.41768043 | 9.110117491 | 4.191093748 | 0.001120304 | 0.006573866 | -1.201719112 |
| ZNF397 | 1.221777667 | 6.058064181 | 4.190482284 | 0.001121561 | 0.006578697 | -1.2028575 |
| 2-Mar | -1.211225168 | 8.116822571 | -4.190091892 | 0.001122365 | 0.006580862 | -1.203584324 |
| MTX3 | -1.619816075 | 4.506717854 | -4.188107503 | 0.001126459 | 0.006602311 | -1.207279011 |
| ARL6IP5 | -1.302242423 | 11.4717414 | -4.186823108 | 0.001129116 | 0.00661533 | -1.209670567 |
| RLN2 | -3.134854755 | 5.07743283 | -4.185437904 | 0.00113199 | 0.006629604 | -1.212249976 |
| TMEM273 | -1.160769436 | 5.069047807 | -4.184828883 | 0.001133256 | 0.006633137 | -1.213384094 |
| MPP7 | -2.053615707 | 5.406504705 | -4.184726501 | 0.001133469 | 0.006633137 | -1.213574751 |
| CHAMP1 | 1.226601985 | 7.002449172 | 4.180549067 | 0.001142193 | 0.006676455 | -1.22135479 |
| SRRM2 | 1.875872897 | 11.03118021 | 4.179210804 | 0.001145003 | 0.006687717 | -1.223847462 |
| ZBED6 | 1.120168033 | 9.303864723 | 4.178149016 | 0.001147237 | 0.006695604 | -1.225825268 |
| FAM69B | 1.987293196 | 9.819470372 | 4.177733345 | 0.001148113 | 0.006695716 | -1.226599569 |
| CYSTM1 | -2.874034199 | 9.585878685 | -4.177720419 | 0.00114814 | 0.006695716 | -1.226623647 |
| NRIP2 | 1.670717667 | 5.247561796 | 4.177353441 | 0.001148914 | 0.006697651 | -1.227307255 |
| ADAT2 | 1.526821435 | 9.857206352 | 4.176901741 | 0.001149867 | 0.006700629 | -1.228148698 |
| LPAR4 | 2.341573064 | 3.178138658 | 4.175371499 | 0.001153102 | 0.006716899 | -1.23099941 |
| LCP2 | -1.387613756 | 9.922621151 | -4.172037802 | 0.001160183 | 0.00675036 | -1.237210452 |
| HTR6 | 1.089682334 | 7.170389424 | 4.170843307 | 0.001162731 | 0.006762589 | -1.23943614 |
| NR3C1 | 1.230700303 | 9.89916298 | 4.169482893 | 0.00116564 | 0.00677171 | -1.241971123 |
| RALY | 1.043751188 | 5.733965278 | 4.167610302 | 0.001169657 | 0.006789836 | -1.245460728 |
| CENPE | 2.345819984 | 5.261342456 | 4.167159405 | 0.001170626 | 0.006790558 | -1.246301024 |
| CASP3 | 2.271976334 | 9.656172469 | 4.166276081 | 0.001172527 | 0.006796084 | -1.247947242 |
| SLC35D2 | -1.051954665 | 8.576031476 | -4.163795639 | 0.001177883 | 0.006821755 | -1.252570275 |
| TUBA3D | 1.082756929 | 12.95802755 | 4.16369608 | 0.001178099 | 0.006821755 | -1.252755843 |
| TRIM40 | -1.383712147 | 2.952905741 | -4.163599739 | 0.001178307 | 0.006821755 | -1.252935413 |
| METRN | -1.615663078 | 7.297936652 | -4.162302423 | 0.00118112 | 0.006835425 | -1.255353556 |
| HIBCH | 1.012412705 | 7.441274403 | 4.158957783 | 0.001188402 | 0.006864277 | -1.261588431 |
| TSPAN9 | -1.211685999 | 3.074788213 | -4.158764862 | 0.001188823 | 0.006864277 | -1.26194809 |
| ITGA7 | 1.024584273 | 7.747474306 | 4.158439634 | 0.001189534 | 0.006865765 | -1.262554413 |
| BMP5 | 1.649778957 | 4.172593287 | 4.157306772 | 0.001192014 | 0.006877456 | -1.264666469 |
| GPR65 | -3.411281541 | 9.455866989 | -4.157026282 | 0.001192628 | 0.006878384 | -1.265189417 |
| SLC9A3R2 | 1.756029125 | 8.65484545 | 4.156615016 | 0.00119353 | 0.006880966 | -1.265956198 |
| PATJ | -1.877739582 | 3.021827726 | -4.155875496 | 0.001195154 | 0.006884467 | -1.26733502 |
| TRIP13 | 1.591189434 | 6.272412422 | 4.154039252 | 0.001199195 | 0.006903119 | -1.270758849 |
| TFDP2 | 1.889971338 | 5.604037025 | 4.150120688 | 0.001207867 | 0.006946334 | -1.27806621 |
| ZNF718 | 1.730677561 | 4.621571436 | 4.150025549 | 0.001208078 | 0.006946334 | -1.278243639 |
| MMS22L | 1.292458798 | 5.567883842 | 4.149665283 | 0.001208879 | 0.006948301 | -1.278915528 |
| LYZ | -2.659135687 | 9.44085558 | -4.147562977 | 0.001213561 | 0.006972571 | -1.282836484 |
| CCDC124 | -1.194556 | 9.765213686 | -4.147042137 | 0.001214725 | 0.006976608 | -1.283807941 |
| CTDP1 | 1.216072872 | 5.892210599 | 4.143369886 | 0.001222957 | 0.007013258 | -1.290657927 |
| GPATCH1 | -1.067572902 | 7.405665291 | -4.142688126 | 0.001224492 | 0.007018414 | -1.291929753 |
| BCORL1 | 1.760846386 | 6.954702089 | 4.142559038 | 0.001224783 | 0.007018414 | -1.29217057 |
| VPS18 | 3.913754879 | 11.66112261 | 4.137334707 | 0.001236613 | 0.007078368 | -1.301917814 |
| ZNF704 | 3.842394915 | 3.610396305 | 4.137319687 | 0.001236647 | 0.007078368 | -1.30194584 |
| WDR35 | 1.148691309 | 6.100028036 | 4.136088492 | 0.001239452 | 0.007088777 | -1.304243235 |
| ZNF25 | -1.630148139 | 5.901915277 | -4.13590568 | 0.001239869 | 0.007088777 | -1.304584369 |
| PRAMEF4 | -1.304711097 | 7.080911102 | -4.135494436 | 0.001240808 | 0.007091469 | -1.305351776 |
| VPS13C | 1.04809996 | 8.750972857 | 4.133990618 | 0.001244247 | 0.007108442 | -1.308158107 |
| TMEM52 | 1.096580135 | 6.226380988 | 4.131985711 | 0.001248847 | 0.007129346 | -1.311899803 |
| BRS3 | 1.749201333 | 3.76857064 | 4.131766243 | 0.001249352 | 0.007129541 | -1.312309408 |
| RECK | -1.953020613 | 6.056251411 | -4.128635854 | 0.001256573 | 0.007156775 | -1.318152219 |
| C20orf85 | 1.240288245 | 5.53925108 | 4.128240068 | 0.001257489 | 0.007157104 | -1.318891 |
| FAM167B | -1.297487083 | 6.413666753 | -4.1274493 | 0.001259321 | 0.00716484 | -1.320367094 |
| ARL11 | 2.206519319 | 4.289040834 | 4.126923066 | 0.001260542 | 0.007166685 | -1.321349417 |
| UBL3 | -1.045404272 | 8.767326241 | -4.126901735 | 0.001260591 | 0.007166685 | -1.321389237 |
| HS6ST1 | 1.440427018 | 9.909425346 | 4.126346255 | 0.001261881 | 0.007170075 | -1.32242618 |
| TAS2R19 | 1.824115131 | 6.046722873 | 4.125674215 | 0.001263444 | 0.007172138 | -1.323680742 |
| RSU1 | -1.726554736 | 5.25659125 | -4.125040979 | 0.001264918 | 0.007177816 | -1.324862897 |
| CALHM2 | -2.500662756 | 9.089032122 | -4.122845511 | 0.001270042 | 0.007201905 | -1.328961729 |
| DHTKD1 | 1.255849803 | 8.159248139 | 4.122481471 | 0.001270894 | 0.007201905 | -1.329641409 |
| FZD10 | 1.121069872 | 2.974850461 | 4.121658788 | 0.001272821 | 0.007209165 | -1.331177435 |
| RAB7A | -1.083629957 | 9.173063195 | -4.120255675 | 0.001276115 | 0.007222421 | -1.333797293 |
| TMEM44 | 1.672508268 | 6.911225034 | 4.119157084 | 0.0012787 | 0.007226313 | -1.335848658 |
| LRRC49 | 1.092832209 | 4.445555813 | 4.117668546 | 0.001282211 | 0.007240716 | -1.338628303 |
| CELF1 | 1.904435887 | 9.955050626 | 4.117666976 | 0.001282215 | 0.007240716 | -1.338631234 |
| OR8U1 | 1.244717856 | 6.135182136 | 4.117238625 | 0.001283227 | 0.007241477 | -1.339431153 |
| MOB4 | -1.053819111 | 9.712258662 | -4.117205427 | 0.001283306 | 0.007241477 | -1.339493149 |
| RIMKLB | 2.16691214 | 6.27925099 | 4.116500789 | 0.001284973 | 0.007245487 | -1.340809047 |
| ARID2 | 1.287227766 | 8.002742371 | 4.114766288 | 0.001289086 | 0.007265658 | -1.344048353 |
| TUBB | 1.467018504 | 11.09097693 | 4.114446323 | 0.001289846 | 0.007265658 | -1.344645935 |
| CDH7 | 1.753656557 | 9.714622145 | 4.114386298 | 0.001289989 | 0.007265658 | -1.344758042 |
| TFPI | 3.723261136 | 5.642615847 | 4.113444224 | 0.00129223 | 0.007275577 | -1.346517553 |
| MEGF11 | 1.551621505 | 2.731344615 | 4.11205699 | 0.001295538 | 0.007288631 | -1.349108608 |
| BAALC | 2.77331784 | 6.102361169 | 4.112053192 | 0.001295547 | 0.007288631 | -1.349115703 |
| TOB1 | -2.041392956 | 9.249245804 | -4.111866814 | 0.001295992 | 0.007288631 | -1.349463828 |
| CTSL | 2.09218676 | 8.243274359 | 4.109845221 | 0.001300829 | 0.007313122 | -1.353240014 |
| ABCC5 | 1.382970889 | 9.340625729 | 4.109463867 | 0.001301744 | 0.007315549 | -1.35395239 |
| NCAPD2 | 1.602209214 | 10.21946773 | 4.109092096 | 0.001302636 | 0.007317849 | -1.354646874 |
| NUAK1 | -1.118992118 | 5.341693554 | -4.108263292 | 0.001304628 | 0.00732632 | -1.35619515 |
| ZFP14 | 1.055164163 | 5.275644748 | 4.107485471 | 0.001306499 | 0.007334114 | -1.357648233 |
| SOAT2 | -1.11822198 | 4.879200036 | -4.105502529 | 0.001311284 | 0.007355521 | -1.36135286 |
| WDR62 | 1.965874407 | 3.682555048 | 4.101351401 | 0.001321358 | 0.007403809 | -1.369109126 |
| AL845331.2 | 1.186411016 | 2.759466527 | 4.100595963 | 0.0013232 | 0.007408979 | -1.370520775 |
| RWDD4 | -1.412313562 | 5.418702842 | -4.10021609 | 0.001324127 | 0.007411062 | -1.371230641 |
| PTPRH | 2.164669894 | 4.328549771 | 4.099979611 | 0.001324705 | 0.007411062 | -1.371672554 |
| FER | 1.038200679 | 7.270382019 | 4.098374495 | 0.001328632 | 0.007425342 | -1.374672165 |
| WNT5B | -1.142846341 | 3.750189629 | -4.098129984 | 0.001329231 | 0.007425952 | -1.375129119 |
| CDC25B | -1.613668515 | 9.550236565 | -4.097192694 | 0.001331531 | 0.007427863 | -1.376880812 |
| ELOF1 | -1.603028584 | 9.655107084 | -4.097097875 | 0.001331764 | 0.007427863 | -1.377058022 |
| RHAG | 5.546734072 | 4.642161437 | 4.096975547 | 0.001332064 | 0.007427863 | -1.377286647 |
| ZBED8 | 1.926593493 | 3.769530673 | 4.096923661 | 0.001332192 | 0.007427863 | -1.377383618 |
| TAS2R4 | 1.343733759 | 6.755143423 | 4.096792261 | 0.001332515 | 0.007427863 | -1.377629199 |
| LRRC43 | -1.837717495 | 3.413261824 | -4.096542078 | 0.00133313 | 0.007428558 | -1.378096781 |
| CHST15 | -1.921603011 | 6.395933514 | -4.095692016 | 0.001335222 | 0.007435621 | -1.379685551 |
| KIAA1024 | 1.445906894 | 9.341590475 | 4.095628214 | 0.001335379 | 0.007435621 | -1.379804799 |
| ZNF487 | -1.385933977 | 3.42808443 | -4.095173518 | 0.001336499 | 0.007438743 | -1.380654652 |
| CYP4A22 | 1.739558535 | 3.660302593 | 4.094385138 | 0.001338444 | 0.00743902 | -1.382128214 |
| OR10A6 | 1.448730844 | 2.336212379 | 4.094129 | 0.001339077 | 0.007439376 | -1.382606972 |
| NSUN7 | -2.221854159 | 3.27253263 | -4.091302299 | 0.001346078 | 0.00746517 | -1.387890782 |
| ZNF471 | 1.46720197 | 4.445918672 | 4.091291186 | 0.001346106 | 0.00746517 | -1.387911557 |
| AGA | -1.410530143 | 8.671878678 | -4.090979438 | 0.00134688 | 0.007466286 | -1.388494326 |
| ITGB2 | -1.60349776 | 11.28849717 | -4.090085633 | 0.001349103 | 0.007473586 | -1.390165211 |
| FAM107B | -2.050127729 | 10.87062211 | -4.089277186 | 0.001351117 | 0.007482007 | -1.391676578 |
| GPR179 | 4.922846536 | 6.879553133 | 4.088417645 | 0.001353261 | 0.007488409 | -1.393283516 |
| NLRC4 | -2.570978806 | 4.842335785 | -4.086870074 | 0.001357132 | 0.007507083 | -1.396176877 |
| SLC16A9 | 1.370229472 | 6.577267648 | 4.086400326 | 0.001358309 | 0.007510851 | -1.397055158 |
| KIF4A | 2.19821784 | 5.451354841 | 4.085624509 | 0.001360255 | 0.007518869 | -1.398505729 |
| CDKL5 | -2.784577052 | 5.344308934 | -4.084105781 | 0.001364073 | 0.007534476 | -1.401345467 |
| CUL7 | 1.067199991 | 6.564823161 | 4.081878004 | 0.001369693 | 0.007562764 | -1.405511291 |
| RBPJ | 1.306524751 | 10.13832565 | 4.081139762 | 0.001371561 | 0.007570318 | -1.406891844 |
| TTC21B | 1.222933474 | 8.338699612 | 4.079845705 | 0.001374842 | 0.00758014 | -1.409311891 |
| GP1BB | -3.750627838 | 12.25117256 | -4.079254102 | 0.001376344 | 0.007585104 | -1.410418302 |
| TIMP1 | -1.692703183 | 10.77986721 | -4.078900118 | 0.001377244 | 0.007585104 | -1.411080333 |
| GJC3 | 2.027789463 | 4.53303423 | 4.07812865 | 0.001379207 | 0.007590081 | -1.412523183 |
| TMEM254 | -1.250252559 | 5.771991466 | -4.077881369 | 0.001379837 | 0.007590081 | -1.412985672 |
| CCDC85B | -1.250134803 | 10.85388603 | -4.077629231 | 0.001380479 | 0.007590081 | -1.413457252 |
| MAZ | 1.21875264 | 8.852330992 | 4.077561286 | 0.001380652 | 0.007590081 | -1.413584332 |
| ZNF20 | 1.244206149 | 3.092655086 | 4.076191368 | 0.001384149 | 0.007606544 | -1.4161466 |
| SKA3 | 2.880421562 | 5.293190727 | 4.07425798 | 0.001389099 | 0.007628215 | -1.419762998 |
| DHX36 | 1.312465514 | 10.08614116 | 4.073016289 | 0.001392288 | 0.007642957 | -1.422085716 |
| ACTL7A | 1.408529513 | 7.54824986 | 4.072380056 | 0.001393925 | 0.007648521 | -1.423275902 |
| LRRC40 | 1.015671246 | 8.601824916 | 4.072229973 | 0.001394312 | 0.007648521 | -1.423556664 |
| SFTA2 | 1.023518882 | 4.22376931 | 4.07145735 | 0.001396303 | 0.007656671 | -1.425002036 |
| THOC2 | 1.094863792 | 8.670787467 | 4.070178276 | 0.001399605 | 0.00766923 | -1.427394939 |
| MRTFB | 1.120999983 | 5.256538488 | 4.069348273 | 0.001401753 | 0.007675445 | -1.428947775 |
| GCNT1 | 1.110446632 | 5.963595501 | 4.065685436 | 0.00141127 | 0.007721975 | -1.435801085 |
| ADAMTS1 | 1.405694146 | 8.977460008 | 4.065159409 | 0.001412642 | 0.007726693 | -1.436785377 |
| MORN4 | -2.206717452 | 4.020307504 | -4.063454924 | 0.001417098 | 0.007741773 | -1.439974915 |
| NDUFA12 | -1.417790753 | 11.7613023 | -4.063323152 | 0.001417443 | 0.007741773 | -1.440221504 |
| TRIM32 | -1.965594741 | 4.277649667 | -4.062907951 | 0.001418531 | 0.007744923 | -1.440998489 |
| CRISP3 | -1.400686614 | 2.41311923 | -4.06246561 | 0.001419691 | 0.007746403 | -1.441826274 |
| ELMSAN1 | 1.251737767 | 8.899600858 | 4.06241463 | 0.001419825 | 0.007746403 | -1.441921677 |
| VPS26C | -1.190821049 | 7.552515973 | -4.059965647 | 0.001426265 | 0.007777124 | -1.446504896 |
| TMEM245 | 1.441418569 | 9.000520582 | 4.059883269 | 0.001426482 | 0.007777124 | -1.446659072 |
| OGT | 1.646983559 | 12.21254229 | 4.059398706 | 0.00142776 | 0.007780061 | -1.447565973 |
| LYRM1 | -1.019172159 | 8.920726696 | -4.059289681 | 0.001428048 | 0.007780061 | -1.447770025 |
| SPIB | -1.57985864 | 9.23374937 | -4.058256752 | 0.001430776 | 0.007791235 | -1.449703301 |
| ROCK2 | 1.259830548 | 6.271888191 | 4.058124182 | 0.001431127 | 0.007791235 | -1.44995143 |
| DNM1L | 1.050472196 | 9.178940284 | 4.057441007 | 0.001432935 | 0.007798277 | -1.451230138 |
| SYNCRIP | 1.079816709 | 10.91290444 | 4.056465844 | 0.001435521 | 0.007809541 | -1.453055418 |
| KCNMB3 | 1.289927137 | 5.83364112 | 4.054957158 | 0.00143953 | 0.00782854 | -1.455879462 |
| LY75-CD302 | -2.028579498 | 9.655000181 | -4.054117126 | 0.001441767 | 0.007837893 | -1.457451947 |
| PARPBP | 1.171821651 | 5.08125351 | 4.052597625 | 0.001445823 | 0.007850345 | -1.460296478 |
| PCSK6 | -1.106676412 | 2.730224382 | -4.051391808 | 0.00144905 | 0.007854937 | -1.462553902 |
| PHF6 | 1.150706913 | 7.713268122 | 4.050728186 | 0.001450829 | 0.007861768 | -1.463796318 |
| PCDHGA5 | 1.974510885 | 3.266746559 | 4.047684382 | 0.001459018 | 0.007895804 | -1.469495231 |
| ATP4B | 1.052830593 | 2.101768878 | 4.045895746 | 0.001463852 | 0.007918179 | -1.47284439 |
| ZNF610 | 1.724546631 | 3.020103 | 4.044927205 | 0.001466476 | 0.007926716 | -1.474658037 |
| TMEM251 | -1.405255156 | 6.976359326 | -4.043640577 | 0.001469971 | 0.007939937 | -1.477067421 |
| IFNA17 | 1.25141462 | 7.280656089 | 4.042360662 | 0.001473455 | 0.007955921 | -1.479464344 |
| LRRC6 | -1.48633821 | 6.388991536 | -4.039490729 | 0.001481299 | 0.007995424 | -1.484839327 |
| PPP1CA | -1.058470953 | 11.7466384 | -4.036300679 | 0.001490068 | 0.008025602 | -1.490814491 |
| KRT3 | 1.033701639 | 9.65614191 | 4.035574835 | 0.001492071 | 0.008033533 | -1.492174139 |
| LIMK1 | -1.646698952 | 8.294948039 | -4.034475313 | 0.00149511 | 0.008044178 | -1.494233824 |
| OGFRL1 | -1.915903746 | 7.335742043 | -4.031364589 | 0.001503742 | 0.008079147 | -1.500061438 |
| ANKRD34A | -1.532609012 | 2.480596256 | -4.030348343 | 0.001506573 | 0.008091489 | -1.501965406 |
| TM4SF19 | -1.402129607 | 3.41554297 | -4.030087504 | 0.001507301 | 0.008092528 | -1.502454109 |
| FBXW7 | 1.044715131 | 7.274611607 | 4.029662143 | 0.001508488 | 0.008096034 | -1.503251064 |
| TIGD5 | 1.116167755 | 9.710356286 | 4.028880119 | 0.001510673 | 0.008104891 | -1.504716292 |
| CFAP36 | 1.589994041 | 3.748394234 | 4.026927362 | 0.001516144 | 0.008131364 | -1.508375225 |
| CEP128 | 1.301489162 | 6.782878916 | 4.024811079 | 0.001522097 | 0.008160398 | -1.512340846 |
| FAM209B | -1.476881956 | 5.958478678 | -4.023796577 | 0.001524958 | 0.008167071 | -1.514241985 |
| GPAT2 | -1.260205853 | 8.597702261 | -4.023029783 | 0.001527125 | 0.008175786 | -1.515678974 |
| SEC61G | -1.313011302 | 11.33333311 | -4.022533942 | 0.001528528 | 0.008177998 | -1.516608211 |
| DPRX | 1.213682704 | 6.518176398 | 4.022502155 | 0.001528618 | 0.008177998 | -1.516667783 |
| KLHL34 | -1.363428204 | 4.492123869 | -4.01808765 | 0.001541167 | 0.008236407 | -1.524941561 |
| ARHGEF15 | 1.43276792 | 9.972155828 | 4.017789456 | 0.001542018 | 0.008236914 | -1.52550049 |
| TLCD2 | 1.501751527 | 2.957738329 | 4.017673693 | 0.001542349 | 0.008236914 | -1.525717476 |
| MIB1 | 1.565445999 | 6.531058313 | 4.015177041 | 0.001549498 | 0.008269529 | -1.530397392 |
| MCCC2 | 1.120522898 | 7.165615206 | 4.015159854 | 0.001549547 | 0.008269529 | -1.530429611 |
| RHBDF1 | 2.827815062 | 3.315794852 | 4.013265169 | 0.001554996 | 0.008289846 | -1.533981426 |
| NAMPT | -1.479831579 | 9.81566247 | -4.012725445 | 0.001556552 | 0.008292879 | -1.534993246 |
| TIMP3 | 1.368122671 | 5.096091393 | 4.012688014 | 0.00155666 | 0.008292879 | -1.535063418 |
| CCND3 | -1.63516103 | 11.20210472 | -4.01235196 | 0.001557629 | 0.008295127 | -1.535693428 |
| TK1 | 2.803718219 | 12.23610496 | 4.01173405 | 0.001559413 | 0.008301711 | -1.536851861 |
| RTP4 | -1.623469543 | 9.138671026 | -4.009459714 | 0.001565999 | 0.008330915 | -1.541115907 |
| TM4SF1 | -1.692392844 | 4.301488949 | -4.009199733 | 0.001566753 | 0.008332004 | -1.541603354 |
| GON7 | -1.594466006 | 5.908997837 | -4.008134676 | 0.001569849 | 0.008345536 | -1.543600311 |
| SULT1C2 | 1.443806249 | 9.032366402 | 4.00626956 | 0.001575284 | 0.008369886 | -1.547097536 |
| CCM2 | -1.293907829 | 8.759660307 | -4.004428082 | 0.00158067 | 0.008388347 | -1.550550653 |
| NPR3 | 5.480130134 | 5.532586838 | 4.002772988 | 0.001585527 | 0.008411174 | -1.553654451 |
| KRIT1 | 1.85046881 | 3.053466036 | 4.001537168 | 0.001589163 | 0.008424566 | -1.555972097 |
| CRISP2 | -1.141598307 | 2.943649328 | -4.001229929 | 0.001590068 | 0.008425795 | -1.556548306 |
| CYB5D2 | -1.261285597 | 9.480035079 | -4.000876674 | 0.00159111 | 0.008425795 | -1.557210822 |
| HPSE | -2.871284515 | 6.023327659 | -4.000468394 | 0.001592315 | 0.008425795 | -1.557976546 |
| PANX2 | 1.969421607 | 7.570928584 | 3.999919543 | 0.001593936 | 0.008427972 | -1.559005924 |
| APOLD1 | 1.102118521 | 7.777447652 | 3.9956072 | 0.001606732 | 0.00847323 | -1.567094449 |
| NT5DC1 | -1.415909282 | 8.2063766 | -3.99448616 | 0.001610075 | 0.008487909 | -1.569197341 |
| CYP4F2 | 3.159089464 | 3.083350461 | 3.993951621 | 0.001611672 | 0.008493372 | -1.570200077 |
| WDR34 | 1.584667969 | 9.289423066 | 3.993189965 | 0.00161395 | 0.00850242 | -1.571628892 |
| ISPD | 2.575779605 | 5.212499115 | 3.992944016 | 0.001614687 | 0.008503342 | -1.572090283 |
| UBN2 | 1.06429108 | 7.794881516 | 3.992216563 | 0.001616866 | 0.008511864 | -1.57345498 |
| NT5C3A | -1.364177364 | 11.20262586 | -3.991291093 | 0.001619644 | 0.008522958 | -1.575191203 |
| BCL11A | 2.217121534 | 8.012564099 | 3.991139708 | 0.001620099 | 0.008522958 | -1.575475213 |
| RALGAPA2 | 1.232833266 | 7.663182843 | 3.990670476 | 0.00162151 | 0.008527419 | -1.576355537 |
| BRAT1 | 1.085403867 | 11.65475511 | 3.989148394 | 0.001626094 | 0.008547627 | -1.579211207 |
| NFKBIA | -1.456779141 | 14.52153593 | -3.989020273 | 0.001626481 | 0.008547627 | -1.579451588 |
| TMEM174 | 1.48005662 | 7.286622834 | 3.988326109 | 0.001628576 | 0.008555673 | -1.580754005 |
| RAI1 | 1.374096397 | 6.79002289 | 3.986903645 | 0.00163288 | 0.008575306 | -1.583422976 |
| NCAPD3 | 1.327060609 | 8.295553706 | 3.985985929 | 0.001635662 | 0.008583968 | -1.585144954 |
| PKP2 | -1.943320294 | 3.682476338 | -3.985259647 | 0.001637867 | 0.008592566 | -1.586507768 |
| CDK2 | 1.289520706 | 6.322820146 | 3.985002001 | 0.001638651 | 0.008593143 | -1.586991229 |
| INSR | 1.911467748 | 7.545091481 | 3.984830159 | 0.001639173 | 0.008593143 | -1.587313685 |
| PDP1 | -1.129571697 | 9.320573078 | -3.984461444 | 0.001640295 | 0.008593143 | -1.588005574 |
| SEMG1 | -1.056781383 | 2.988202782 | -3.984373215 | 0.001640563 | 0.008593143 | -1.588171134 |
| CD1A | -1.975211748 | 5.674346617 | -3.984291161 | 0.001640813 | 0.008593143 | -1.58832511 |
| MYCN | 6.319402053 | 6.077380905 | 3.982411164 | 0.001646547 | 0.00861668 | -1.591853046 |
| ERICH2 | -1.446688626 | 2.33155108 | -3.98225839 | 0.001647014 | 0.00861668 | -1.592139744 |
| CCR10 | -1.780650006 | 7.81642638 | -3.98178606 | 0.001648458 | 0.008621259 | -1.593026142 |
| UQCR10 | -1.280524907 | 11.25352169 | -3.979696117 | 0.001654864 | 0.008651774 | -1.596948387 |
| MELK | 2.775981439 | 5.948325235 | 3.977899818 | 0.00166039 | 0.008674465 | -1.600319759 |
| HIF3A | 1.348973725 | 10.88433589 | 3.977054733 | 0.001662996 | 0.008682309 | -1.60190592 |
| PRDM10 | 1.22437894 | 6.474772429 | 3.975277133 | 0.001668493 | 0.008707908 | -1.605242481 |
| TUT4 | 1.602135742 | 5.519824066 | 3.975097489 | 0.001669049 | 0.008707908 | -1.605579683 |
| FAM184B | 1.447637923 | 4.74332685 | 3.973262272 | 0.001674745 | 0.008731611 | -1.609024612 |
| LBR | 1.193257427 | 12.12767418 | 3.970466208 | 0.001683461 | 0.008768004 | -1.61427356 |
| AL096814.1 | 1.213001346 | 5.980874513 | 3.966192048 | 0.001696876 | 0.008828766 | -1.622298189 |
| MYOG | 1.18642333 | 7.276437534 | 3.963498572 | 0.001705386 | 0.008869996 | -1.627355686 |
| NAALADL2 | 2.054238593 | 4.850867176 | 3.963081344 | 0.001706708 | 0.008873826 | -1.628139146 |
| SH2B2 | -1.773922875 | 10.4778753 | -3.962801606 | 0.001707595 | 0.008875392 | -1.628664436 |
| FOXO3 | 1.573960428 | 10.15362923 | 3.96219646 | 0.001709515 | 0.008882327 | -1.629800793 |
| COMMD6 | -1.997150369 | 6.076407941 | -3.9616355 | 0.001711297 | 0.008888539 | -1.630854195 |
| GPAT3 | -3.403352985 | 7.813373744 | -3.961019616 | 0.001713256 | 0.008895664 | -1.63201076 |
| KLHL9 | 1.118266522 | 9.376352092 | 3.96010613 | 0.001716166 | 0.008907719 | -1.633726229 |
| MYL5 | -1.211400319 | 8.743823504 | -3.959006339 | 0.001719676 | 0.008920436 | -1.635791632 |
| TAS2R13 | 1.349510119 | 4.899231533 | 3.958883498 | 0.001720069 | 0.008920436 | -1.636022331 |
| RCAN2 | -1.536373335 | 4.968945488 | -3.95855979 | 0.001721103 | 0.008920436 | -1.636630271 |
| LRP1 | -1.055510188 | 7.394214286 | -3.958454086 | 0.001721441 | 0.008920436 | -1.636828789 |
| XPOT | 1.001935474 | 8.160675364 | 3.958416953 | 0.00172156 | 0.008920436 | -1.636898526 |
| KLHL13 | 2.681106739 | 3.331423253 | 3.958004204 | 0.001722881 | 0.008924227 | -1.637673701 |
| UGT2B15 | 1.596033966 | 4.647941721 | 3.953061989 | 0.001738775 | 0.00899846 | -1.64695633 |
| SOX12 | 1.067286194 | 7.159075289 | 3.952994392 | 0.001738993 | 0.00899846 | -1.647083302 |
| SLC27A2 | 3.876405794 | 5.855464384 | 3.951762383 | 0.00174298 | 0.009016006 | -1.649397528 |
| MYMX | -1.279362658 | 3.037921113 | -3.950987521 | 0.001745491 | 0.009021715 | -1.650853087 |
| SPRYD4 | 1.104183313 | 9.344110405 | 3.950417113 | 0.001747343 | 0.00902625 | -1.651924607 |
| RTN4RL2 | 1.181748103 | 9.623110887 | 3.947391849 | 0.001757196 | 0.009074054 | -1.657607924 |
| KIAA0100 | 1.124914571 | 10.84236805 | 3.946051745 | 0.001761579 | 0.009090491 | -1.660125636 |
| GRK1 | 1.363555656 | 4.380467501 | 3.945695262 | 0.001762747 | 0.00909342 | -1.660795393 |
| TMEM191C | -1.058339405 | 8.839567056 | -3.943266034 | 0.001770726 | 0.009131471 | -1.665359604 |
| POLB | -1.072877468 | 7.065657725 | -3.9427034 | 0.001772579 | 0.009136321 | -1.66641677 |
| PDHA1 | 2.283205874 | 4.904294599 | 3.940188441 | 0.001780887 | 0.009174507 | -1.671142494 |
| PCDHB1 | 1.127559873 | 4.605031522 | 3.938318267 | 0.001787092 | 0.00920334 | -1.674656871 |
| CD300A | -1.956801843 | 9.706560717 | -3.933634846 | 0.001802726 | 0.00927106 | -1.683458689 |
| CHMP3 | -1.047056945 | 6.363841792 | -3.933573652 | 0.001802932 | 0.00927106 | -1.683573701 |
| ATF3 | 1.063477864 | 9.748523018 | 3.932342141 | 0.001807067 | 0.009281483 | -1.685888358 |
| CASP14 | -1.237687004 | 5.123118804 | -3.932312488 | 0.001807166 | 0.009281483 | -1.685944094 |
| CYSLTR1 | -1.098682201 | 8.689606983 | -3.931964169 | 0.001808338 | 0.009284352 | -1.686598783 |
| ZNF658 | -1.383521516 | 4.577670187 | -3.930926335 | 0.001811832 | 0.009299143 | -1.688549509 |
| POLR1B | 1.149285402 | 5.765844687 | 3.927947877 | 0.0018219 | 0.00934765 | -1.694148184 |
| TLN1 | -1.103942941 | 9.491729325 | -3.926565166 | 0.001826594 | 0.009365387 | -1.696747466 |
| SDS | 1.286798032 | 9.004889607 | 3.923592476 | 0.001836726 | 0.009407787 | -1.702336018 |
| NR4A3 | 2.722049972 | 6.235455486 | 3.922604124 | 0.001840107 | 0.009421922 | -1.704194192 |
| ARL17A | 1.585996718 | 8.696497312 | 3.921807362 | 0.001842838 | 0.009432717 | -1.705692203 |
| DENND4B | 1.027899253 | 12.92489637 | 3.920229962 | 0.001848257 | 0.009454063 | -1.708658012 |
| FANCD2 | 1.576671683 | 2.420403544 | 3.918270405 | 0.00185501 | 0.009479009 | -1.712342537 |
| FAM228B | -1.090575429 | 8.989728989 | -3.917895313 | 0.001856306 | 0.009482432 | -1.71304784 |
| HSD17B7 | 1.617528507 | 4.927518571 | 3.917665689 | 0.0018571 | 0.009483289 | -1.713479618 |
| NR2F1 | 1.753730607 | 3.149682447 | 3.916141846 | 0.001862375 | 0.009503823 | -1.716345071 |
| TMED8 | 1.448653218 | 5.840279267 | 3.915196768 | 0.001865655 | 0.009514906 | -1.718122271 |
| KIF1C | -1.850165115 | 7.835896315 | -3.915154136 | 0.001865803 | 0.009514906 | -1.71820244 |
| TCF12 | 1.146694772 | 7.924306943 | 3.91441802 | 0.001868362 | 0.009521545 | -1.719586727 |
| GCAT | 1.701965843 | 4.825467563 | 3.912361952 | 0.001875528 | 0.009548431 | -1.723453373 |
| TRIM71 | 2.345864258 | 5.524053794 | 3.911374139 | 0.001878981 | 0.009562797 | -1.725311138 |
| CCNT1 | 1.045499444 | 9.57322643 | 3.911123862 | 0.001879857 | 0.009564042 | -1.725781838 |
| PDE6B | -1.166481208 | 7.216664181 | -3.910092081 | 0.001883473 | 0.009579219 | -1.727722363 |
| BNIP1 | -1.242353463 | 9.351485473 | -3.909370996 | 0.001886003 | 0.009588872 | -1.729078579 |
| ATAD2B | 1.120713489 | 7.510440645 | 3.908742894 | 0.001888211 | 0.009596874 | -1.730259936 |
| CHSY1 | 1.477498869 | 10.67788803 | 3.905966989 | 0.001897998 | 0.00963692 | -1.735481208 |
| SPATS2 | 1.218043067 | 7.869134311 | 3.905603907 | 0.001899282 | 0.009640209 | -1.736164168 |
| KRTAP5-2 | 1.169081419 | 6.030242087 | 3.905095632 | 0.001901081 | 0.009646109 | -1.737120252 |
| NUDT5 | 1.380385322 | 6.087922939 | 3.904080142 | 0.001904681 | 0.009657441 | -1.739030462 |
| FAHD2A | 1.029925393 | 6.026196552 | 3.903926192 | 0.001905227 | 0.009657441 | -1.739320057 |
| DLC1 | 2.835969582 | 3.50075678 | 3.901993327 | 0.001912099 | 0.009682559 | -1.742956086 |
| GNPDA2 | -1.453552536 | 7.848447481 | -3.90044368 | 0.001917627 | 0.009700826 | -1.74587136 |
| XYLT1 | 1.576958434 | 6.559282564 | 3.897745769 | 0.001927291 | 0.009743206 | -1.750947109 |
| NLRP7 | -1.366396368 | 4.24903495 | -3.891754321 | 0.001948931 | 0.009842751 | -1.762220551 |
| RTP5 | 1.055621995 | 6.077964212 | 3.890269107 | 0.001954334 | 0.009865085 | -1.765015399 |
| SLC52A3 | 1.495623784 | 3.37110046 | 3.890002057 | 0.001955307 | 0.009865085 | -1.765517941 |
| WDR92 | 1.20696512 | 6.455874861 | 3.888898396 | 0.001959333 | 0.009882111 | -1.767594879 |
| FSCN1 | 1.204224962 | 5.911035819 | 3.888160636 | 0.00196203 | 0.009889127 | -1.768983277 |
| TNFAIP8L2 | -1.637762235 | 9.161960898 | -3.887958724 | 0.001962769 | 0.00988956 | -1.769363261 |
| SNRPB2 | -1.238461295 | 10.05874529 | -3.886333515 | 0.001968724 | 0.00991627 | -1.772421873 |
| CAD | 1.019619931 | 9.144513928 | 3.884072395 | 0.001977041 | 0.009954853 | -1.776677479 |
| ZNF2 | -1.233425032 | 3.581550506 | -3.883447995 | 0.001979344 | 0.009959575 | -1.777852694 |
| SRPK2 | -1.000557253 | 7.680478439 | -3.883357677 | 0.001979678 | 0.009959575 | -1.778022688 |
| MT-ATP8 | 1.46819747 | 14.84000703 | 3.882589692 | 0.001982515 | 0.009965852 | -1.779468183 |
| GNG2 | -2.300803215 | 8.763629311 | -3.882048291 | 0.001984517 | 0.009971557 | -1.780487221 |
| PSPC1 | 1.135878407 | 8.112881173 | 3.881927051 | 0.001984966 | 0.009971557 | -1.780715425 |
| ASAH1 | -1.806501848 | 12.89339085 | -3.881003556 | 0.001988387 | 0.009982124 | -1.782453689 |
| E2F1 | 1.932904199 | 8.370853917 | 3.877862312 | 0.002000069 | 0.010031022 | -1.788366673 |
| ZYX | -1.077741393 | 11.03402796 | -3.877850515 | 0.002000113 | 0.010031022 | -1.78838888 |
| EXD3 | 1.102609188 | 6.335958533 | 3.877226327 | 0.002002443 | 0.010039383 | -1.789563893 |
| RUNX3 | -1.688933804 | 12.21714079 | -3.876923478 | 0.002003575 | 0.010041732 | -1.790134002 |
| DEFB132 | 1.1748692 | 5.109008059 | 3.875681945 | 0.00200822 | 0.010048393 | -1.792471221 |
| SPRR2G | 1.029924264 | 8.243224867 | 3.873561633 | 0.002016178 | 0.010080101 | -1.796462941 |
| KRTAP26-1 | 1.804878431 | 3.151109176 | 3.872482582 | 0.00202024 | 0.010091109 | -1.79849446 |
| LMTK3 | -2.414012312 | 6.481168606 | -3.872169029 | 0.002021422 | 0.010091109 | -1.799084793 |
| PKIB | -1.57676932 | 2.80783732 | -3.872154461 | 0.002021477 | 0.010091109 | -1.799112221 |
| GPR21 | 1.29885731 | 4.710697353 | 3.871993446 | 0.002022085 | 0.010091109 | -1.799415369 |
| AMN1 | -1.092412918 | 6.523471737 | -3.870692862 | 0.002026997 | 0.010108964 | -1.801864072 |
| C1GALT1C1 | -1.226817724 | 9.094742988 | -3.869198853 | 0.002032655 | 0.010133549 | -1.804677052 |
| SLCO4A1 | 3.205308343 | 4.456461484 | 3.868510778 | 0.002035266 | 0.010137653 | -1.805972625 |
| RFC5 | 1.409393708 | 9.757190467 | 3.867461128 | 0.002039256 | 0.010153392 | -1.80794905 |
| RXFP4 | 1.046442508 | 5.792029128 | 3.866811957 | 0.002041728 | 0.010162359 | -1.809171425 |
| PBX4 | -2.254399915 | 8.575437806 | -3.866405872 | 0.002043276 | 0.010165272 | -1.809936083 |
| CNPY3 | -1.10175053 | 10.41332693 | -3.866306399 | 0.002043655 | 0.010165272 | -1.810123394 |
| HSD17B13 | 1.73833765 | 2.396091252 | 3.865151133 | 0.002048066 | 0.010178777 | -1.812298811 |
| KIAA1522 | 2.556399056 | 7.631307588 | 3.865067369 | 0.002048386 | 0.010178777 | -1.812456547 |
| ERVW-1 | 1.37791193 | 4.129778166 | 3.86342618 | 0.002054669 | 0.010206653 | -1.815547099 |
| CR2 | -1.551021582 | 3.60065831 | -3.862514349 | 0.002058169 | 0.010220686 | -1.817264241 |
| NEURL1B | 1.070602247 | 7.732171008 | 3.861021713 | 0.002063911 | 0.010245842 | -1.82007523 |
| HMX3 | 1.241812188 | 9.187116295 | 3.860760249 | 0.002064918 | 0.010247486 | -1.82056764 |
| BRCA2 | 1.206204639 | 4.965210987 | 3.86001278 | 0.002067801 | 0.010258433 | -1.821975351 |
| ZNF621 | 1.850179732 | 8.278334169 | 3.859462938 | 0.002069924 | 0.010265605 | -1.823010888 |
| C1orf226 | 1.380024744 | 3.647468206 | 3.857985188 | 0.002075642 | 0.010290593 | -1.825794058 |
| ZNF619 | 1.341098654 | 6.495858674 | 3.855760503 | 0.00208428 | 0.010323286 | -1.829984189 |
| PDCD1 | -1.125993687 | 8.780407029 | -3.852163623 | 0.002098325 | 0.010388352 | -1.836759307 |
| ITGA4 | 1.401268909 | 9.868060381 | 3.851289306 | 0.002101753 | 0.01039963 | -1.838406271 |
| TMEM30B | -2.180918481 | 3.92310017 | -3.849965061 | 0.002106957 | 0.010421975 | -1.840900839 |
| FMOD | 1.785524014 | 3.262464381 | 3.849636891 | 0.002108249 | 0.01042496 | -1.841519049 |
| GAL | 1.726201147 | 6.108343897 | 3.846735474 | 0.002119704 | 0.010467937 | -1.846984971 |
| HCK | -2.721353186 | 9.761438139 | -3.842222762 | 0.002137647 | 0.010535942 | -1.855487154 |
| ALG10 | 1.144808928 | 5.069931677 | 3.84026074 | 0.002145496 | 0.010571192 | -1.859183997 |
| KRTAP5-9 | 1.697543675 | 3.722452375 | 3.839300831 | 0.002149347 | 0.010586724 | -1.860992722 |
| MT1M | -3.298332742 | 6.793194837 | -3.838006868 | 0.00215455 | 0.010605454 | -1.863430961 |
| CAVIN3 | -1.104876337 | 8.101646057 | -3.835921809 | 0.00216296 | 0.010643395 | -1.867360035 |
| EP300 | 1.145676036 | 7.742628023 | 3.831482828 | 0.002180978 | 0.010723358 | -1.87572548 |
| CDK5R1 | -1.428509602 | 7.655069833 | -3.831395526 | 0.002181334 | 0.010723358 | -1.875890014 |
| FUT1 | 2.776673367 | 4.767814709 | 3.830220583 | 0.002186129 | 0.010736481 | -1.878104395 |
| Z82190.2 | 1.09974856 | 6.506503374 | 3.828862558 | 0.002191685 | 0.010753312 | -1.8806639 |
| DPT | 1.046059398 | 9.457426269 | 3.827140277 | 0.002198753 | 0.010781004 | -1.883910047 |
| P2RY14 | -2.90624417 | 6.359889689 | -3.826413072 | 0.002201744 | 0.010790692 | -1.885280719 |
| SF1 | 1.036226191 | 13.34395059 | 3.826133682 | 0.002202894 | 0.010790692 | -1.885807333 |
| IL17C | -1.101456184 | 5.122504641 | -3.826050255 | 0.002203238 | 0.010790692 | -1.885964583 |
| OXCT1 | -1.825768778 | 7.73841829 | -3.825923205 | 0.002203761 | 0.010790692 | -1.886204059 |
| TBX10 | 1.045027077 | 7.088802067 | 3.825794907 | 0.00220429 | 0.010790692 | -1.886445887 |
| PROK1 | 1.172249817 | 2.425207718 | 3.825479616 | 0.002205589 | 0.010793567 | -1.887040178 |
| TBCC | -1.291746041 | 9.631985975 | -3.824476031 | 0.002209731 | 0.010810345 | -1.888931863 |
| SPG7 | 1.026212391 | 7.849755056 | 3.824140468 | 0.002211118 | 0.010813637 | -1.889564386 |
| TXK | -1.52056632 | 7.44937785 | -3.823410382 | 0.002214138 | 0.010824913 | -1.890940583 |
| PNLIPRP2 | 1.005890199 | 2.165394799 | 3.82003624 | 0.002228151 | 0.01087938 | -1.897301077 |
| FASTKD3 | -1.313200292 | 8.182029535 | -3.819413983 | 0.002230745 | 0.010888537 | -1.898474128 |
| IFIH1 | -1.054506775 | 8.375446931 | -3.816891811 | 0.002241292 | 0.010936493 | -1.903228988 |
| NDRG1 | 1.187451884 | 11.7607171 | 3.813625893 | 0.002255024 | 0.010992049 | -1.90938638 |
| NPNT | 1.011062483 | 9.411140791 | 3.813494256 | 0.002255579 | 0.010992049 | -1.909634572 |
| SACS | 1.208484244 | 8.365191097 | 3.812869618 | 0.002258216 | 0.011001361 | -1.910812286 |
| CRYZ | -1.382232379 | 5.95913736 | -3.810834537 | 0.002266829 | 0.011032678 | -1.914649417 |
| SOX15 | 1.144166291 | 5.955722143 | 3.809265201 | 0.002273494 | 0.011057714 | -1.917608506 |
| PCDHGA8 | 1.415335937 | 3.741315662 | 3.809107965 | 0.002274163 | 0.011057714 | -1.917904991 |
| SOCS4 | 1.184190404 | 8.831274969 | 3.808700406 | 0.002275897 | 0.011061413 | -1.91867349 |
| PIP4P2 | -1.01687158 | 9.687425717 | -3.807172993 | 0.00228241 | 0.011076489 | -1.921553666 |
| KLHDC10 | 1.041092761 | 7.598841721 | 3.805761943 | 0.002288444 | 0.011102214 | -1.924214509 |
| PSMD9 | -1.321663218 | 6.082791258 | -3.804906688 | 0.002292109 | 0.011116435 | -1.925827318 |
| TNFRSF17 | -3.056922254 | 6.204117779 | -3.803712102 | 0.002297238 | 0.011137746 | -1.928080077 |
| BATF | -1.719255569 | 11.44442679 | -3.803487846 | 0.002298203 | 0.011138857 | -1.928502987 |
| LIN7A | -4.053580141 | 5.528688162 | -3.802936131 | 0.002300576 | 0.011143233 | -1.92954344 |
| RASL11A | -1.023928718 | 6.85598024 | -3.802526546 | 0.00230234 | 0.011148213 | -1.930315865 |
| SCGB3A2 | -1.170812728 | 9.352650808 | -3.79965255 | 0.002314757 | 0.011201176 | -1.935736047 |
| NUDT19 | -1.135583838 | 9.579512016 | -3.798952679 | 0.002317791 | 0.011212277 | -1.937056012 |
| SLC38A8 | 1.341299453 | 4.063629283 | 3.795691225 | 0.002331984 | 0.011271259 | -1.943207417 |
| TNFRSF13C | -1.928299723 | 8.110735553 | -3.795638069 | 0.002332216 | 0.011271259 | -1.943307677 |
| MRPL50 | -1.123102487 | 9.645366826 | -3.794432816 | 0.002337483 | 0.011293116 | -1.945581007 |
| RNPC3 | 1.369047477 | 9.154098945 | 3.792838359 | 0.002344471 | 0.011319655 | -1.948588536 |
| ARMH4 | 1.529546383 | 2.541737058 | 3.791375291 | 0.002350901 | 0.011339863 | -1.951348323 |
| PELP1 | 1.229798674 | 11.15387319 | 3.79116879 | 0.002351811 | 0.011340637 | -1.951737854 |
| ALDH1A2 | -1.233857643 | 2.289612915 | -3.789886075 | 0.002357465 | 0.011361888 | -1.954157521 |
| SWI5 | -1.107025408 | 8.587684963 | -3.789715677 | 0.002358218 | 0.011361888 | -1.954478959 |
| CD48 | -1.79339165 | 6.898357686 | -3.789649107 | 0.002358512 | 0.011361888 | -1.954604537 |
| FOXP1 | 1.474896862 | 11.0304283 | 3.789319614 | 0.002359967 | 0.011361888 | -1.955226094 |
| MSI2 | 1.930091769 | 10.63098848 | 3.788351223 | 0.00236425 | 0.01137528 | -1.957052896 |
| POTED | 4.274971651 | 3.757984027 | 3.787373791 | 0.002368581 | 0.0113925 | -1.958896791 |
| TRMT10B | -1.015678768 | 7.231587624 | -3.786622399 | 0.002371916 | 0.011401302 | -1.960314294 |
| STK11IP | 1.00130405 | 11.71774584 | 3.782519927 | 0.00239021 | 0.011474674 | -1.96805401 |
| WDR89 | -1.103802188 | 5.262394097 | -3.781073137 | 0.002396696 | 0.011500411 | -1.970783675 |
| NBPF19 | 1.13837103 | 12.56797021 | 3.779634476 | 0.002403163 | 0.011518608 | -1.973498084 |
| CHPF | -1.596468573 | 8.568904484 | -3.778226079 | 0.002409511 | 0.011536414 | -1.976155467 |
| ANGPT1 | 2.51534667 | 8.555991226 | 3.776648399 | 0.002416643 | 0.011564928 | -1.979132348 |
| UGDH | -1.102369915 | 6.050769612 | -3.776216262 | 0.0024186 | 0.011570639 | -1.97994775 |
| OR10G7 | 1.356962682 | 2.513531331 | 3.77553798 | 0.002421676 | 0.011581695 | -1.981227622 |
| INTS7 | 1.030591301 | 7.091364856 | 3.774654748 | 0.002425686 | 0.011597215 | -1.982894246 |
| ALKAL2 | -2.227150843 | 3.484926962 | -3.773300869 | 0.002431847 | 0.011623002 | -1.985449019 |
| TUBA4A | -1.669289517 | 8.995723439 | -3.77205266 | 0.002437541 | 0.011644518 | -1.987804452 |
| ZNF395 | 2.384395882 | 8.274584766 | 3.77197721 | 0.002437886 | 0.011644518 | -1.987946832 |
| SGTB | -1.831140569 | 7.947703658 | -3.771452823 | 0.002440283 | 0.011652291 | -1.988936396 |
| F7 | 1.141140713 | 4.032642872 | 3.767921514 | 0.002456484 | 0.011718575 | -1.995600548 |
| STARD9 | 1.091240576 | 3.88535173 | 3.767320222 | 0.002459254 | 0.011728095 | -1.996735329 |
| IPO7 | 1.370592103 | 8.170723245 | 3.76625757 | 0.002464157 | 0.011740594 | -1.998740838 |
| TXNL4A | -1.123908653 | 9.967720052 | -3.766250077 | 0.002464191 | 0.011740594 | -1.99875498 |
| RNASE8 | 1.056233436 | 2.029700482 | 3.766248282 | 0.0024642 | 0.011740594 | -1.998758367 |
| HLA-DQA2 | -2.360503318 | 11.35413014 | -3.765183426 | 0.002469123 | 0.011756655 | -2.00076808 |
| ABCA4 | -1.012565531 | 2.338346978 | -3.763369032 | 0.002477534 | 0.0117854 | -2.004192495 |
| ITPRID2 | 1.395854359 | 9.828507395 | 3.763042918 | 0.002479049 | 0.0117854 | -2.004808001 |
| PLK5 | 1.481670417 | 4.215127526 | 3.76218331 | 0.002483047 | 0.011795043 | -2.006430443 |
| AC012476.1 | 2.552276621 | 6.081446276 | 3.762160779 | 0.002483151 | 0.011795043 | -2.00647297 |
| BRINP3 | 1.478149063 | 3.280161408 | 3.762104617 | 0.002483413 | 0.011795043 | -2.006578971 |
| ZNF681 | 1.067555547 | 4.746799835 | 3.76131422 | 0.002487095 | 0.011805347 | -2.008070808 |
| EDAR | -1.999318587 | 2.837330666 | -3.761304442 | 0.002487141 | 0.011805347 | -2.008089264 |
| PROCR | -2.147724491 | 6.76812567 | -3.760531815 | 0.002490746 | 0.011818756 | -2.009547583 |
| KIAA1109 | 1.04305417 | 7.470429201 | 3.758169642 | 0.0025018 | 0.011863779 | -2.014006273 |
| HACD3 | 1.171706626 | 10.27696368 | 3.757539955 | 0.002504755 | 0.011871261 | -2.015194865 |
| ANOS1 | 2.305142227 | 3.473130669 | 3.75641511 | 0.002510043 | 0.011887986 | -2.017318146 |
| FAM170A | 1.968133817 | 4.746994629 | 3.754112802 | 0.002520902 | 0.01192823 | -2.021664172 |
| SYTL3 | -2.547045615 | 7.798292822 | -3.75366855 | 0.002523003 | 0.011934444 | -2.0225028 |
| MTRNR2L10 | 1.052748362 | 9.278148247 | 3.753043294 | 0.002525963 | 0.011944716 | -2.023683126 |
| TRIM65 | 1.075754775 | 9.366762867 | 3.752102122 | 0.002530425 | 0.011962082 | -2.025459848 |
| TCN1 | -3.324096305 | 7.548325577 | -3.749117047 | 0.002544631 | 0.012017987 | -2.031095204 |
| SLC2A5 | 1.748355576 | 7.551173039 | 3.748827256 | 0.002546014 | 0.012020773 | -2.031642302 |
| RAB6D | 2.004953037 | 11.37926771 | 3.748227187 | 0.002548881 | 0.012030561 | -2.032775181 |
| NANOS2 | 1.024293962 | 3.381871585 | 3.747172317 | 0.002553929 | 0.012050633 | -2.034766717 |
| ABCB4 | -3.112029782 | 5.235475488 | -3.746733517 | 0.002556032 | 0.012054912 | -2.035595159 |
| EPHX4 | -1.723291985 | 4.043684135 | -3.74665105 | 0.002556428 | 0.012054912 | -2.035750854 |
| AARD | 1.010012817 | 5.474097199 | 3.746420887 | 0.002557531 | 0.012056365 | -2.036185398 |
| ASCL2 | -2.336687895 | 9.867124267 | -3.746136515 | 0.002558896 | 0.012059046 | -2.036722292 |
| WSB1 | 1.239478084 | 8.13309668 | 3.74558421 | 0.002561548 | 0.012064041 | -2.037765047 |
| SDHAF1 | -1.122143266 | 10.48378767 | -3.745380637 | 0.002562527 | 0.012064898 | -2.038149396 |
| PCM1 | 1.33805093 | 7.826590446 | 3.74513423 | 0.002563711 | 0.012066726 | -2.038614621 |
| ADAMTSL4 | -1.040295927 | 7.586850157 | -3.744920823 | 0.002564738 | 0.012067808 | -2.03901754 |
| OR2AG2 | 1.251873859 | 8.356008081 | 3.744162973 | 0.002568387 | 0.012078223 | -2.0404484 |
| HID1 | -1.792840896 | 6.472906152 | -3.743561686 | 0.002571285 | 0.012087354 | -2.041583674 |
| KRTAP10-3 | 1.025952032 | 10.12392815 | 3.741134979 | 0.002583018 | 0.012134977 | -2.046165601 |
| MAGEA2 | 1.433531816 | 2.270818186 | 3.740890442 | 0.002584203 | 0.012136782 | -2.04662733 |
| KRT35 | 1.528161867 | 5.129909131 | 3.739616074 | 0.002590389 | 0.012150769 | -2.049033585 |
| CDC20 | 1.669508694 | 8.258030188 | 3.737742246 | 0.002599513 | 0.012186019 | -2.052571836 |
| TCTEX1D1 | 2.846340505 | 3.897157828 | 3.736791175 | 0.002604156 | 0.01220401 | -2.054367738 |
| FXYD6 | -1.219528417 | 5.830203508 | -3.731580356 | 0.002629746 | 0.012289723 | -2.064207818 |
| NAA20 | -1.060528397 | 10.62742859 | -3.730914021 | 0.002633037 | 0.012298704 | -2.065466185 |
| ETV2 | -1.685636315 | 7.567111583 | -3.730652422 | 0.00263433 | 0.012298704 | -2.065960215 |
| KRT8 | 1.305138818 | 4.880579 | 3.730534175 | 0.002634915 | 0.012298704 | -2.066183527 |
| TEKT5 | -1.403163299 | 3.254171239 | -3.730160544 | 0.002636763 | 0.012303541 | -2.066889136 |
| AURKA | 2.075760868 | 6.517519705 | 3.72958226 | 0.002639627 | 0.012305611 | -2.067981243 |
| PPT1 | -1.188118858 | 13.29589799 | -3.729271359 | 0.002641167 | 0.012308929 | -2.068568395 |
| ABCB10 | 1.454296467 | 7.05991893 | 3.727565274 | 0.002649639 | 0.012340818 | -2.071790473 |
| DDX21 | 1.141648147 | 11.65539442 | 3.727157992 | 0.002651666 | 0.01234624 | -2.072559671 |
| RAB30 | -1.142884056 | 6.777344896 | -3.727003834 | 0.002652433 | 0.01234624 | -2.072850816 |
| CTNND1 | 1.316870952 | 7.213604688 | 3.726112091 | 0.002656877 | 0.012355537 | -2.074534997 |
| CDK20 | -2.33490954 | 5.959594211 | -3.725741785 | 0.002658724 | 0.012360336 | -2.075234377 |
| TRH | 6.893954293 | 6.475352759 | 3.724502208 | 0.002664919 | 0.012379584 | -2.077575546 |
| DONSON | 1.179688266 | 4.409884317 | 3.72442277 | 0.002665316 | 0.012379584 | -2.077725581 |
| PIP5K1C | -1.162158387 | 7.32730812 | -3.724106914 | 0.002666897 | 0.012383131 | -2.07832214 |
| CATSPERB | -2.172564032 | 3.34467946 | -3.72300345 | 0.002672428 | 0.012405009 | -2.080406289 |
| LDB1 | 1.430176395 | 9.548124894 | 3.721431316 | 0.002680327 | 0.012437869 | -2.083375693 |
| PCDHAC2 | 1.102420233 | 3.183629637 | 3.720873973 | 0.002683134 | 0.012447079 | -2.084428407 |
| UGP2 | -1.267807919 | 10.21640178 | -3.719174896 | 0.002691707 | 0.012463955 | -2.087637692 |
| PKNOX1 | 1.300652765 | 8.456903536 | 3.717675601 | 0.002699296 | 0.012491459 | -2.090469696 |
| ETV7 | -2.159734807 | 5.404023803 | -3.715383218 | 0.002710942 | 0.012541519 | -2.094799884 |
| DEPTOR | 2.583809562 | 7.649985109 | 3.714808666 | 0.002713868 | 0.012551227 | -2.095885208 |
| CIB2 | -1.774262903 | 7.983765161 | -3.714631937 | 0.002714769 | 0.012551562 | -2.096219048 |
| URAD | 1.714031133 | 4.372850097 | 3.714180145 | 0.002717074 | 0.012558384 | -2.097072488 |
| PLPPR3 | 2.874207755 | 5.896012596 | 3.713227364 | 0.00272194 | 0.012576873 | -2.098872321 |
| SPINT3 | 1.132235094 | 4.662750909 | 3.713072104 | 0.002722734 | 0.012576873 | -2.099165615 |
| FAM189A1 | 1.226723781 | 7.798530612 | 3.712199387 | 0.002727201 | 0.012593665 | -2.100814233 |
| CXCL11 | -1.588105528 | 2.540097983 | -3.711368157 | 0.002731462 | 0.0126095 | -2.102384499 |
| TXNL4B | -1.256312737 | 5.820175621 | -3.71032553 | 0.002736816 | 0.01263037 | -2.104354143 |
| C20orf27 | -1.123140837 | 11.58319505 | -3.708591316 | 0.002745746 | 0.012663867 | -2.107630344 |
| HLA-DMB | -1.729872702 | 13.20102179 | -3.707940348 | 0.002749106 | 0.012675504 | -2.108860148 |
| LARS2 | 1.0094165 | 7.458582174 | 3.704831973 | 0.002765207 | 0.012737781 | -2.11473263 |
| ZNF736 | 1.439228826 | 7.131099973 | 3.704683926 | 0.002765976 | 0.012737781 | -2.115012335 |
| DRP2 | 1.053272073 | 10.34211183 | 3.703599438 | 0.002771617 | 0.01275947 | -2.11706127 |
| HTR1A | 1.169340909 | 3.27079128 | 3.701299635 | 0.00278362 | 0.012807357 | -2.12140642 |
| PEPD | -1.214459383 | 11.23971029 | -3.700876892 | 0.002785832 | 0.012813644 | -2.122205148 |
| DCTN3 | -1.085046704 | 11.32830053 | -3.69964384 | 0.002792294 | 0.012838512 | -2.1245349 |
| NT5DC3 | 2.78416017 | 4.210492608 | 3.699522052 | 0.002792934 | 0.012838512 | -2.124765012 |
| ZNF90 | -1.223955931 | 2.969062337 | -3.698875572 | 0.002796329 | 0.01285022 | -2.125986503 |
| CARD14 | -1.878817505 | 2.69632142 | -3.697047856 | 0.00280595 | 0.012878808 | -2.129439941 |
| OR52N4 | -1.526797406 | 3.190214198 | -3.69676217 | 0.002807457 | 0.012881822 | -2.129979749 |
| GDF15 | 3.085696906 | 8.497993771 | 3.69591229 | 0.002811945 | 0.012894604 | -2.131585621 |
| ACP5 | -1.839168859 | 11.14820247 | -3.694493772 | 0.002819451 | 0.012917589 | -2.134265994 |
| HLA-G | -1.128536645 | 16.99330791 | -3.694481824 | 0.002819515 | 0.012917589 | -2.134288571 |
| GET4 | 1.168651526 | 9.428605892 | 3.693456494 | 0.002824954 | 0.012935645 | -2.136226026 |
| BARHL1 | 1.042833624 | 7.079209603 | 3.693417002 | 0.002825163 | 0.012935645 | -2.136300652 |
| PI4KA | 1.10328487 | 10.78435068 | 3.692512815 | 0.002829969 | 0.012951483 | -2.138009221 |
| KIAA1549 | 2.925812097 | 3.979974256 | 3.691338848 | 0.002836221 | 0.012967402 | -2.140227605 |
| TSPAN2 | -3.20969897 | 4.997462303 | -3.691309211 | 0.002836379 | 0.012967402 | -2.140283608 |
| PRADC1 | -1.415172288 | 8.501350365 | -3.690718218 | 0.002839531 | 0.012975 | -2.141400391 |
| PPP4R2 | 1.087119738 | 6.772687845 | 3.690676635 | 0.002839753 | 0.012975 | -2.141478971 |
| TTC13 | -1.576132882 | 6.964635434 | -3.689909801 | 0.00284385 | 0.0129898 | -2.142928051 |
| SH3RF1 | 2.443803819 | 5.075282964 | 3.687884935 | 0.002854696 | 0.013031483 | -2.146754497 |
| ARMT1 | -1.486562356 | 7.931782074 | -3.685742115 | 0.002866219 | 0.013073935 | -2.150803962 |
| PUM2 | 1.004147571 | 10.30901019 | 3.685514359 | 0.002867447 | 0.013073935 | -2.151234379 |
| PDP2 | 1.268178018 | 5.746414926 | 3.683381664 | 0.002878968 | 0.013118214 | -2.15526484 |
| RBM43 | 1.305739347 | 9.04533166 | 3.680972468 | 0.00289204 | 0.013160203 | -2.159817986 |
| UST | -1.743346266 | 2.846156826 | -3.680897506 | 0.002892448 | 0.013160203 | -2.15995966 |
| CCR7 | -2.324700091 | 11.80899419 | -3.679064184 | 0.002902437 | 0.013197726 | -2.163424563 |
| ZFP69 | -1.072894059 | 5.746544943 | -3.678890163 | 0.002903387 | 0.013198084 | -2.16375346 |
| CIT | 1.973330159 | 2.756469177 | 3.678558239 | 0.0029052 | 0.013202364 | -2.164380793 |
| C20orf194 | 1.722581421 | 7.878571198 | 3.677720881 | 0.002909779 | 0.013215244 | -2.165963404 |
| HLA-DOB | -1.214328127 | 9.654858156 | -3.67710515 | 0.00291315 | 0.013226591 | -2.16712715 |
| APOBEC2 | 1.756685151 | 3.249464662 | 3.676756725 | 0.00291506 | 0.013226643 | -2.167785685 |
| POP4 | -1.699028563 | 4.921285306 | -3.676625274 | 0.00291578 | 0.013226643 | -2.168034132 |
| RAB11FIP4 | -1.150308637 | 8.795373795 | -3.676352855 | 0.002917275 | 0.013229461 | -2.168549018 |
| FBXO32 | -2.334296305 | 5.89257343 | -3.675789397 | 0.002920368 | 0.013239525 | -2.169613985 |
| FDXACB1 | -1.041430698 | 6.078704595 | -3.670962267 | 0.002947005 | 0.013342723 | -2.178737849 |
| PLS3 | -1.603337154 | 2.40697885 | -3.6705632 | 0.002949218 | 0.013342723 | -2.179492161 |
| OR51D1 | 1.157385929 | 6.136113695 | 3.670549189 | 0.002949296 | 0.013342723 | -2.179518644 |
| ZNF485 | -1.168506957 | 5.218610214 | -3.669812799 | 0.002953384 | 0.013354665 | -2.180910567 |
| TMEM87A | 1.114993339 | 6.260439323 | 3.669722712 | 0.002953885 | 0.013354665 | -2.18108085 |
| HOXA4 | 1.293354815 | 3.396733527 | 3.667600929 | 0.002965699 | 0.013400928 | -2.185091519 |
| SLC22A18AS | -2.711987238 | 5.210633791 | -3.666624713 | 0.002971151 | 0.01341669 | -2.186936833 |
| SPC24 | 2.81320098 | 5.733993964 | 3.666606432 | 0.002971254 | 0.01341669 | -2.186971389 |
| TAPT1 | 1.419350203 | 8.446569662 | 3.664743144 | 0.002981689 | 0.013457123 | -2.190493572 |
| PLCG1 | -2.432978014 | 5.899617533 | -3.663719049 | 0.00298744 | 0.013475051 | -2.192429459 |
| PTCHD4 | 2.249779329 | 4.378264126 | 3.661152497 | 0.003001903 | 0.01353223 | -2.197281219 |
| MRPS18A | -1.013565672 | 9.184107967 | -3.659896509 | 0.003009006 | 0.013556773 | -2.199655567 |
| ZNF324 | 1.002835772 | 8.294315299 | 3.6592119 | 0.003012885 | 0.013569628 | -2.200949783 |
| STAT5A | 1.689148998 | 9.589551193 | 3.65747326 | 0.00302276 | 0.013606013 | -2.204236634 |
| BTBD3 | 1.075016531 | 6.534962665 | 3.657284308 | 0.003023835 | 0.01360681 | -2.204593846 |
| RAD54L2 | 1.430331624 | 10.93269078 | 3.656963399 | 0.003025662 | 0.013609908 | -2.205200524 |
| SDF2 | 1.142167977 | 8.177234287 | 3.656690124 | 0.003027219 | 0.013609908 | -2.205717153 |
| B3GALNT2 | 1.021066928 | 7.699887494 | 3.656451648 | 0.003028578 | 0.013611979 | -2.206167993 |
| AC010616.1 | 1.014952033 | 8.968855393 | 3.656135935 | 0.003030378 | 0.013616031 | -2.206764855 |
| TRIM33 | 1.033985773 | 8.630013116 | 3.653064014 | 0.003047951 | 0.013690929 | -2.212572489 |
| ZNF606 | 1.389652612 | 4.035729279 | 3.65222395 | 0.003052774 | 0.013708532 | -2.21416071 |
| KBTBD8 | -1.831358235 | 4.734326418 | -3.65162614 | 0.003056212 | 0.013719901 | -2.215290937 |
| SCML4 | -2.660998329 | 8.803234772 | -3.650721175 | 0.003061423 | 0.013739223 | -2.217001891 |
| TGFB3 | -1.297891394 | 3.814345181 | -3.648472049 | 0.003074413 | 0.013789351 | -2.221254227 |
| ANK3 | -1.278101003 | 3.637354539 | -3.647144989 | 0.003082104 | 0.013815667 | -2.223763296 |
| DNMT3A | 1.219270921 | 7.81031062 | 3.645434388 | 0.003092046 | 0.013856136 | -2.226997582 |
| LYRM4 | -1.352097123 | 9.382999528 | -3.644748962 | 0.00309604 | 0.013869928 | -2.228293553 |
| HOXC4 | -2.098401305 | 6.725693265 | -3.644439501 | 0.003097844 | 0.01387391 | -2.228878671 |
| EIF4G3 | 1.031054817 | 8.061727668 | 3.644237691 | 0.003099021 | 0.013875081 | -2.229260248 |
| NBPF3 | 1.067424848 | 8.401161958 | 3.644049394 | 0.00310012 | 0.013875216 | -2.229616276 |
| IFI44 | -1.446159375 | 10.16841843 | -3.639218707 | 0.00312845 | 0.013973806 | -2.238750256 |
| RHCG | 1.12021569 | 9.940526843 | 3.638023526 | 0.0031355 | 0.014000966 | -2.241010203 |
| ERVV-1 | 1.123755546 | 4.431627667 | 3.637874693 | 0.003136379 | 0.014000966 | -2.241291632 |
| NUP188 | 1.023046802 | 7.811968648 | 3.637393301 | 0.003139224 | 0.014009538 | -2.242201896 |
| NUSAP1 | 2.422159177 | 8.475149644 | 3.636813465 | 0.003142654 | 0.014013634 | -2.243298316 |
| GSTM3 | -1.571520559 | 3.219114535 | -3.63661286 | 0.003143842 | 0.014013634 | -2.243677645 |
| TRPC4 | 1.353531869 | 2.663006094 | 3.636417661 | 0.003144998 | 0.014014664 | -2.244046751 |
| LCA5 | 1.519471436 | 4.760220099 | 3.63504561 | 0.003153136 | 0.014046797 | -2.246641216 |
| FGF3 | 1.759654633 | 6.497790086 | 3.634707409 | 0.003155145 | 0.014049244 | -2.247280739 |
| HSFX1 | 1.154249857 | 7.147599728 | 3.634641011 | 0.003155539 | 0.014049244 | -2.247406296 |
| IL1RAP | 1.275998328 | 6.940943106 | 3.629937454 | 0.003183623 | 0.014163096 | -2.25630074 |
| IGFBP7 | 1.44363445 | 8.01044273 | 3.629852819 | 0.00318413 | 0.014163096 | -2.256460789 |
| FOSL2 | 2.58623647 | 10.50173284 | 3.629732766 | 0.00318485 | 0.014163096 | -2.256687816 |
| ZNF415 | -2.152683476 | 3.873630754 | -3.629344218 | 0.003187182 | 0.014169308 | -2.25742258 |
| LIPH | -2.349350329 | 4.431038904 | -3.628499996 | 0.003192256 | 0.014187699 | -2.25901906 |
| UGT2B11 | 1.803655994 | 2.691044793 | 3.627469165 | 0.003198461 | 0.014210286 | -2.260968448 |
| TENT5B | 1.030796503 | 5.260020116 | 3.627336706 | 0.00319926 | 0.014210286 | -2.26121894 |
| BUB1 | 2.974444497 | 6.333053123 | 3.626987785 | 0.003201363 | 0.014211503 | -2.261878784 |
| WDR73 | 1.153555151 | 6.048040903 | 3.625878862 | 0.003208059 | 0.014232886 | -2.263975879 |
| TC2N | -3.519255423 | 6.715208657 | -3.624837405 | 0.00321436 | 0.014252495 | -2.26594541 |
| ZNF251 | 1.219580751 | 6.781970507 | 3.624505719 | 0.00321637 | 0.014257233 | -2.266572675 |
| PTCD3 | 1.755578029 | 3.373286473 | 3.623260868 | 0.003223923 | 0.014282357 | -2.268926882 |
| LILRB2 | -3.392564225 | 10.96974234 | -3.622712171 | 0.003227258 | 0.014292952 | -2.269964561 |
| SHF | 1.298262703 | 5.600604772 | 3.621492965 | 0.003234681 | 0.014321641 | -2.272270306 |
| LTBP3 | -1.241422532 | 7.818801426 | -3.620821535 | 0.003238777 | 0.014335584 | -2.273540116 |
| BNIP3 | -2.099917857 | 9.92618513 | -3.619495087 | 0.003246883 | 0.014352688 | -2.276048721 |
| SDC1 | 1.744377822 | 6.813570655 | 3.616733497 | 0.003263826 | 0.014412772 | -2.281271587 |
| FBXO25 | -1.060135614 | 9.277292003 | -3.616478837 | 0.003265392 | 0.01441549 | -2.281753219 |
| SIRPB1 | -1.175318601 | 8.49216831 | -3.616183436 | 0.003267211 | 0.014419316 | -2.282311906 |
| ZNF695 | 1.569411796 | 2.916919872 | 3.615407556 | 0.003271992 | 0.014436213 | -2.283779321 |
| L3MBTL3 | -2.253906725 | 5.212224744 | -3.614121699 | 0.003279932 | 0.014458612 | -2.286211273 |
| TPD52 | -1.767515598 | 5.462054405 | -3.612014909 | 0.003292983 | 0.014511922 | -2.290195919 |
| GPR135 | 1.658601688 | 5.827312589 | 3.608371835 | 0.003315676 | 0.014590709 | -2.297086355 |
| FZD7 | 1.020842179 | 2.01042309 | 3.607671239 | 0.003320059 | 0.014605751 | -2.29841147 |
| GNB4 | 1.247006166 | 10.10710792 | 3.605230831 | 0.00333537 | 0.014664592 | -2.303027347 |
| CDCA8 | 2.416056954 | 8.775117205 | 3.603411497 | 0.003346831 | 0.014706448 | -2.30646856 |
| MAST3 | -1.265184812 | 8.763566344 | -3.601755389 | 0.003357299 | 0.014748167 | -2.309601076 |
| SOX5 | -1.020222033 | 3.188240537 | -3.601315528 | 0.003360084 | 0.014756127 | -2.310433076 |
| TFF1 | 1.101804911 | 9.440329214 | 3.599907204 | 0.00336902 | 0.01479108 | -2.31309695 |
| THAP3 | -1.244148196 | 8.99108605 | -3.599161665 | 0.00337376 | 0.014807599 | -2.314507164 |
| SLC7A1 | 1.118852831 | 6.247076839 | 3.598757077 | 0.003376335 | 0.01481461 | -2.315272461 |
| PHKA2 | 2.284241137 | 10.20241019 | 3.598104241 | 0.003380494 | 0.014824274 | -2.316507334 |
| CA6 | -1.13025377 | 5.415812199 | -3.597720802 | 0.003382939 | 0.014830705 | -2.31723263 |
| PAQR9 | 1.639416287 | 3.142566759 | 3.595136108 | 0.003399471 | 0.014894557 | -2.322121782 |
| TSR2 | -1.016034412 | 10.96625316 | -3.586007973 | 0.003458512 | 0.015107607 | -2.339389052 |
| DDX58 | -1.383782247 | 6.396920848 | -3.585923336 | 0.003459065 | 0.015107607 | -2.33954916 |
| CD82 | 1.467211492 | 11.23893274 | 3.584760839 | 0.003466659 | 0.015132054 | -2.341748276 |
| HAUS4 | -1.397763328 | 8.411349705 | -3.580990371 | 0.00349141 | 0.015209416 | -2.348881046 |
| IER5L | -2.45543003 | 6.221983292 | -3.580328786 | 0.003495771 | 0.015215539 | -2.350132614 |
| RAI14 | 1.17148487 | 4.933975777 | 3.580260194 | 0.003496224 | 0.015215539 | -2.350262374 |
| TPSAB1 | 5.161844412 | 5.983804985 | 3.580204768 | 0.003496589 | 0.015215539 | -2.350367227 |
| ARHGAP27 | -1.301020659 | 8.708721048 | -3.580167935 | 0.003496832 | 0.015215539 | -2.350436907 |
| HES4 | -2.445759985 | 11.29719799 | -3.579912792 | 0.003498516 | 0.015218495 | -2.35091958 |
| DPEP1 | 1.142268545 | 3.97134253 | 3.579452264 | 0.003501558 | 0.015222985 | -2.351790799 |
| ITPRIPL2 | 1.103892654 | 4.774115614 | 3.579116076 | 0.00350378 | 0.015228276 | -2.352426796 |
| CFHR3 | -2.03957157 | 5.167755619 | -3.578687992 | 0.003506611 | 0.015233766 | -2.35323664 |
| RNF39 | 1.054930011 | 4.207717341 | 3.578578558 | 0.003507336 | 0.015233766 | -2.353443666 |
| TNNI2 | -2.882995948 | 8.907916215 | -3.578469209 | 0.003508059 | 0.015233766 | -2.353650532 |
| BCAS1 | -1.012216204 | 2.497817376 | -3.576533032 | 0.003520901 | 0.015267649 | -2.357313403 |
| LRRC31 | 1.112528091 | 6.693604186 | 3.57486793 | 0.003531982 | 0.015308405 | -2.360463484 |
| KY | 1.034969864 | 2.397894756 | 3.574387618 | 0.003535186 | 0.015312063 | -2.361372155 |
| NADK | -1.102567341 | 9.807353738 | -3.573595205 | 0.003540477 | 0.015326217 | -2.362871276 |
| LY9 | -1.600780931 | 8.370331207 | -3.571839897 | 0.003552226 | 0.015359523 | -2.366192064 |
| NEFH | -2.438138967 | 5.987856493 | -3.570212068 | 0.003563157 | 0.015389221 | -2.369271705 |
| FGFR3 | 1.030014541 | 5.137503684 | 3.568139332 | 0.003577125 | 0.015445147 | -2.373193092 |
| PYCARD | -1.128454979 | 13.38267778 | -3.566789118 | 0.003586254 | 0.015463951 | -2.375747568 |
| SLN | 1.002227211 | 2.22516695 | 3.566589769 | 0.003587604 | 0.015463951 | -2.376124718 |
| PCGF5 | -1.960606749 | 8.502492933 | -3.565491837 | 0.003595048 | 0.015487797 | -2.378201915 |
| HECTD1 | 1.133474631 | 10.54506608 | 3.565453939 | 0.003595305 | 0.015487797 | -2.378273616 |
| TIAM1 | -3.550631621 | 7.744534894 | -3.565311675 | 0.003596271 | 0.015487797 | -2.378542768 |
| HSPB11 | 1.618992705 | 4.351820289 | 3.564494496 | 0.003601823 | 0.015496057 | -2.380088811 |
| SLC14A2 | 1.076632125 | 10.29745728 | 3.564297776 | 0.003603161 | 0.015496057 | -2.380460991 |
| SNAPC3 | 1.426311459 | 4.727458996 | 3.56413851 | 0.003604244 | 0.015496057 | -2.380762312 |
| DUPD1 | -1.384382889 | 4.064357173 | -3.564136583 | 0.003604258 | 0.015496057 | -2.380765959 |
| DNAH2 | -1.153374376 | 6.216888187 | -3.563050654 | 0.003611654 | 0.015523454 | -2.38282047 |
| SEC23IP | 1.311309483 | 6.016789687 | 3.562853348 | 0.003613 | 0.015524834 | -2.383193761 |
| NMNAT3 | 1.745628637 | 6.54018277 | 3.561512759 | 0.003622156 | 0.015555355 | -2.385730089 |
| SUPT3H | 1.703680108 | 6.248524653 | 3.560360781 | 0.003630043 | 0.015580393 | -2.387909587 |
| MKI67 | 2.191143914 | 5.966276588 | 3.559400487 | 0.003636631 | 0.01559983 | -2.389726433 |
| TRAT1 | -3.345903917 | 8.468265162 | -3.555881867 | 0.003660874 | 0.015676936 | -2.396383615 |
| SLC29A1 | 1.542875525 | 9.126024288 | 3.555659824 | 0.003662409 | 0.015676936 | -2.396803721 |
| BBS12 | -1.877787258 | 3.22222025 | -3.554852016 | 0.003668001 | 0.015694402 | -2.398332097 |
| PPP1R27 | 2.529989103 | 5.139604409 | 3.553358144 | 0.003678363 | 0.015725418 | -2.401158523 |
| KCTD13 | 1.243345682 | 11.50260268 | 3.55215389 | 0.003686739 | 0.015743454 | -2.403436998 |
| RHCE | 4.601435606 | 5.819159542 | 3.551570471 | 0.003690803 | 0.01575637 | -2.404540843 |
| RAB11A | -1.041453843 | 8.240584496 | -3.550874939 | 0.003695655 | 0.015772637 | -2.405856813 |
| KLF12 | -2.514156834 | 8.703526691 | -3.549694564 | 0.003703903 | 0.015803388 | -2.408090129 |
| ADAP2 | -2.005305343 | 9.870996201 | -3.54488039 | 0.003737737 | 0.015916374 | -2.417198823 |
| HLCS | 1.026065379 | 5.98385787 | 3.544313016 | 0.003741745 | 0.01592449 | -2.418272335 |
| EIF3C | 1.348205824 | 10.03842252 | 3.543381989 | 0.003748332 | 0.015943565 | -2.420033911 |
| RRS1 | -1.077939953 | 8.826959295 | -3.542390118 | 0.003755362 | 0.015968985 | -2.421910613 |
| TREML2 | 1.067248932 | 9.72750838 | 3.541809157 | 0.003759486 | 0.015982035 | -2.423009841 |
| ENO2 | -2.29369912 | 6.714115827 | -3.541339514 | 0.003762823 | 0.015991734 | -2.423898448 |
| SFT2D3 | 1.440513905 | 5.771499362 | 3.540916463 | 0.003765832 | 0.016000033 | -2.424698899 |
| F2R | -2.001470589 | 8.154171872 | -3.540626939 | 0.003767892 | 0.016004299 | -2.425246707 |
| LTA4H | -1.576290134 | 12.54659421 | -3.540113693 | 0.003771547 | 0.016015335 | -2.426217817 |
| TCTA | -1.289508111 | 9.142934399 | -3.539568324 | 0.003775435 | 0.016027353 | -2.427249708 |
| CYFIP2 | -1.225290335 | 8.826076573 | -3.539351853 | 0.00377698 | 0.016029418 | -2.427659294 |
| PRELID3A | 1.268051664 | 7.348176101 | 3.538719178 | 0.003781497 | 0.016038621 | -2.42885638 |
| GRB2 | -1.02314268 | 9.868910338 | -3.538709041 | 0.00378157 | 0.016038621 | -2.42887556 |
| POU6F1 | -1.66370491 | 4.160352449 | -3.538603463 | 0.003782324 | 0.016038621 | -2.429075324 |
| ORM2 | -1.633955796 | 6.936791638 | -3.538162082 | 0.00378548 | 0.016047511 | -2.429910464 |
| LUC7L3 | 1.202453229 | 9.906447462 | 3.537082715 | 0.003793208 | 0.016075773 | -2.431952744 |
| SPTBN2 | 1.016417234 | 5.634646778 | 3.53641812 | 0.003797974 | 0.016091471 | -2.433210233 |
| SLC39A9 | 1.169257632 | 7.067182959 | 3.536226036 | 0.003799352 | 0.016092812 | -2.433573677 |
| TMED3 | 1.153855004 | 2.88530101 | 3.535883847 | 0.00380181 | 0.01609872 | -2.434221139 |
| HOMER1 | 1.651139689 | 3.294525855 | 3.535280662 | 0.003806145 | 0.016108075 | -2.435362436 |
| TUFT1 | -1.510342912 | 7.01217714 | -3.53425475 | 0.003813531 | 0.016134824 | -2.437303586 |
| TEKT2 | 1.769906461 | 5.101370713 | 3.533799114 | 0.003816816 | 0.016144213 | -2.438165706 |
| RIPOR3 | 3.244795534 | 7.598641859 | 3.53359252 | 0.003818306 | 0.01614601 | -2.438556607 |
| ABHD6 | -1.470981879 | 7.025620878 | -3.53320514 | 0.003821102 | 0.016153325 | -2.43928958 |
| FCGBP | -1.340872053 | 7.260766908 | -3.532891162 | 0.00382337 | 0.016158403 | -2.439883666 |
| NBPF12 | 1.257795267 | 8.557794598 | 3.532393234 | 0.003826969 | 0.016169104 | -2.440825811 |
| PRODH2 | 1.218542398 | 3.169988172 | 3.531430251 | 0.003833939 | 0.016185014 | -2.442647903 |
| MSR1 | -1.909514686 | 3.524312388 | -3.529244188 | 0.003849811 | 0.016238443 | -2.446784235 |
| FOSB | 1.271277543 | 8.863936381 | 3.528397939 | 0.003855973 | 0.016259908 | -2.448385461 |
| SPIN4 | 1.524919556 | 5.883477384 | 3.527373164 | 0.003863448 | 0.016286897 | -2.450324484 |
| DNAJC8 | -1.140687305 | 8.945028274 | -3.527121495 | 0.003865287 | 0.016290113 | -2.45080068 |
| HEG1 | -2.688663634 | 6.468687635 | -3.526786686 | 0.003867733 | 0.016295892 | -2.451434188 |
| TAS2R3 | 1.09909233 | 2.102939651 | 3.526269436 | 0.003871516 | 0.016307296 | -2.452412902 |
| IL27RA | -1.36109264 | 9.215702678 | -3.524450504 | 0.003884849 | 0.016345279 | -2.455854602 |
| HHEX | 1.005118931 | 9.514131012 | 3.52396444 | 0.00388842 | 0.016355761 | -2.45677431 |
| AP1M1 | -1.008767499 | 10.27569776 | -3.523307198 | 0.003893253 | 0.016371547 | -2.458017916 |
| SULT1A2 | -1.427279391 | 11.10522862 | -3.521580215 | 0.003905983 | 0.016407407 | -2.461285645 |
| ATP13A4 | 1.334863164 | 2.528402687 | 3.521562654 | 0.003906113 | 0.016407407 | -2.461318874 |
| APOE | 1.52987748 | 8.260301314 | 3.521310136 | 0.003907978 | 0.016410691 | -2.461796678 |
| NCAPG2 | 1.165687329 | 6.045143339 | 3.520593664 | 0.003913274 | 0.016428379 | -2.46315236 |
| OR1J1 | 1.141245076 | 4.30832583 | 3.51849908 | 0.0039288 | 0.016484422 | -2.467115662 |
| HNRNPH1 | 1.217306417 | 5.856191756 | 3.517063193 | 0.003939479 | 0.016521382 | -2.469832604 |
| CPNE4 | -1.078953867 | 3.261012606 | -3.515415899 | 0.003951767 | 0.016567024 | -2.472949565 |
| H2AFY | 1.314429872 | 14.40548343 | 3.513181866 | 0.003968494 | 0.016623349 | -2.477176743 |
| SPESP1 | -1.833191368 | 2.771856131 | -3.507491693 | 0.004011423 | 0.016770715 | -2.487943551 |
| UQCC1 | 1.127921951 | 8.087711621 | 3.5041883 | 0.004036561 | 0.016864224 | -2.494194152 |
| GCM1 | 1.194742474 | 9.631876489 | 3.504113948 | 0.004037129 | 0.016864224 | -2.494334839 |
| MLLT3 | -1.709440181 | 7.142838635 | -3.501837829 | 0.004054545 | 0.016932307 | -2.498641652 |
| SLFN12L | -1.380349128 | 3.865320764 | -3.501443677 | 0.004057569 | 0.016939456 | -2.499387455 |
| SLA2 | -1.035283278 | 10.56254555 | -3.501138769 | 0.004059909 | 0.016939456 | -2.499964394 |
| ARPP21 | 2.899394433 | 3.299482212 | 3.501126847 | 0.004060001 | 0.016939456 | -2.499986953 |
| TRAM1L1 | 1.316794349 | 2.148997973 | 3.500892671 | 0.004061799 | 0.016939456 | -2.500430053 |
| ZNF37A | 1.305693328 | 6.07136423 | 3.498732702 | 0.004078427 | 0.016989897 | -2.504517083 |
| BACH2 | -1.625377905 | 5.411500242 | -3.49839188 | 0.004081057 | 0.01699618 | -2.505161975 |
| C1QB | -2.506972366 | 7.957623535 | -3.498194434 | 0.004082582 | 0.016997856 | -2.505535577 |
| PSMD12 | 1.226012724 | 7.144460867 | 3.497619214 | 0.004087026 | 0.017011158 | -2.506623989 |
| PLOD2 | -1.705350231 | 3.530426095 | -3.497189725 | 0.004090348 | 0.017011158 | -2.507436654 |
| CUEDC1 | -1.007343631 | 6.512866924 | -3.497129273 | 0.004090816 | 0.017011158 | -2.507551041 |
| SYCP2 | -1.828328053 | 3.905711845 | -3.496793902 | 0.004093412 | 0.017014892 | -2.508185617 |
| SGIP1 | 1.71563275 | 3.603270183 | 3.496331555 | 0.004096993 | 0.017025108 | -2.509060455 |
| DDX31 | 1.367346647 | 7.062582056 | 3.49412354 | 0.004114141 | 0.017072954 | -2.513238378 |
| KLF1 | 2.779389434 | 10.6344959 | 3.491823208 | 0.004132084 | 0.017138023 | -2.517590969 |
| ZNF212 | 1.178996923 | 7.961602605 | 3.490595983 | 0.004141688 | 0.017173158 | -2.519913066 |
| PDE7A | 1.200780231 | 5.19537056 | 3.489923174 | 0.004146964 | 0.017190327 | -2.521186122 |
| CBFA2T3 | 2.391424301 | 9.873087278 | 3.48910784 | 0.004153365 | 0.017212155 | -2.522728855 |
| RHOG | -1.103272062 | 10.66173881 | -3.488645651 | 0.004156999 | 0.017222502 | -2.523603385 |
| ZNF221 | 1.03780244 | 2.659533994 | 3.487222544 | 0.004168207 | 0.017254782 | -2.526296107 |
| ADA | 1.39618202 | 13.70807086 | 3.486794874 | 0.004171581 | 0.017256948 | -2.527105318 |
| C6orf52 | -1.278086485 | 5.453704355 | -3.486515261 | 0.004173789 | 0.017259026 | -2.527634386 |
| RAB9A | -1.290733675 | 10.76602368 | -3.485816674 | 0.004179309 | 0.017272426 | -2.528956208 |
| GCA | -1.243468065 | 12.01291373 | -3.484301258 | 0.00419131 | 0.017305184 | -2.531823577 |
| TDRKH | -1.471735378 | 3.639004795 | -3.480617384 | 0.00422063 | 0.017386285 | -2.53879392 |
| CNDP2 | 1.035666175 | 10.19520922 | 3.480285877 | 0.004223278 | 0.017388543 | -2.539421169 |
| CDKN2D | -1.813011742 | 13.35401882 | -3.4802615 | 0.004223473 | 0.017388543 | -2.539467294 |
| CYP7A1 | 1.557249324 | 4.568428754 | 3.479817277 | 0.004227025 | 0.017398439 | -2.540307814 |
| ARSG | -2.182448135 | 4.862818857 | -3.477326725 | 0.004246995 | 0.017471144 | -2.545020202 |
| MT-ND1 | 1.118297454 | 14.29302788 | 3.476877994 | 0.004250603 | 0.017481241 | -2.545869246 |
| PMCH | -1.536332347 | 4.055071035 | -3.475313024 | 0.004263212 | 0.017514078 | -2.548830315 |
| SLC7A3 | 1.80284888 | 2.615964709 | 3.474319568 | 0.004271235 | 0.017542284 | -2.55071002 |
| CXADR | 1.079462768 | 2.45899809 | 3.473471808 | 0.004278094 | 0.017564336 | -2.552314053 |
| SYNGR1 | 1.00226473 | 9.385902415 | 3.470374881 | 0.004303245 | 0.017635507 | -2.558173658 |
| CTSV | -1.248323035 | 2.647540907 | -3.46996951 | 0.004306549 | 0.017644272 | -2.558940644 |
| ZPBP | 1.527430503 | 4.16608698 | 3.468458348 | 0.004318885 | 0.017685251 | -2.561799845 |
| SLC6A19 | 1.359873613 | 5.265062806 | 3.467526058 | 0.004326514 | 0.017706918 | -2.56356378 |
| PACSIN1 | -2.660547148 | 7.582302066 | -3.466819279 | 0.004332306 | 0.017725837 | -2.564901037 |
| RAPGEF2 | 1.676475338 | 7.963110911 | 3.46252934 | 0.004367634 | 0.017836664 | -2.573017704 |
| QDPR | -1.119503751 | 8.205175523 | -3.460479436 | 0.004384618 | 0.017896376 | -2.576896126 |
| HRASLS2 | -4.144407079 | 7.516533861 | -3.459509872 | 0.004392674 | 0.017923736 | -2.57873053 |
| SF3B3 | 1.248269941 | 8.394805428 | 3.459388109 | 0.004393687 | 0.017923736 | -2.578960904 |
| PER3 | -1.493817798 | 2.751631526 | -3.459047076 | 0.004396525 | 0.017930485 | -2.579606134 |
| FSTL1 | -1.562700079 | 7.596792118 | -3.457728859 | 0.004407512 | 0.017970458 | -2.58210017 |
| ARHGAP11A | 1.546112255 | 4.54162554 | 3.456397511 | 0.004418637 | 0.018006125 | -2.584619035 |
| MICA | -1.229013126 | 9.360939494 | -3.451849873 | 0.004456853 | 0.01813833 | -2.593222898 |
| PHACTR1 | 1.868021104 | 6.0888488 | 3.451824643 | 0.004457066 | 0.01813833 | -2.593270631 |
| CCP110 | 1.181230907 | 7.495728549 | 3.451310992 | 0.004461403 | 0.018146234 | -2.594242417 |
| KRTAP13-4 | 1.325443041 | 7.400227271 | 3.450543524 | 0.004467892 | 0.018155394 | -2.595694399 |
| CCDC112 | -2.07967071 | 5.699459675 | -3.45044233 | 0.004468749 | 0.018155394 | -2.595885848 |
| GTPBP4 | 1.120907374 | 10.32027514 | 3.450336206 | 0.004469647 | 0.018155394 | -2.596086626 |
| ATP7B | 2.126841992 | 7.188108613 | 3.448510146 | 0.004485131 | 0.0181939 | -2.599541344 |
| SERPINA6 | 1.032939765 | 10.12173623 | 3.444234484 | 0.004521599 | 0.018312415 | -2.607630335 |
| NUF2 | 1.90710134 | 6.231563439 | 3.44394068 | 0.004524116 | 0.018312818 | -2.608186166 |
| SDHAF4 | -1.103386724 | 7.848328581 | -3.442921741 | 0.004532856 | 0.018343294 | -2.610113834 |
| C19orf84 | 1.136297587 | 9.191662068 | 3.440969713 | 0.004549647 | 0.018396503 | -2.613806724 |
| CPT1B | 2.066535944 | 10.75712378 | 3.439570146 | 0.004561724 | 0.018435776 | -2.616454431 |
| TLE2 | -2.093395106 | 7.105778501 | -3.439562211 | 0.004561793 | 0.018435776 | -2.616469442 |
| PCDHGB7 | 1.00400193 | 4.410522711 | 3.438495445 | 0.004571021 | 0.018463219 | -2.61848754 |
| PATE1 | 1.262774762 | 3.13449243 | 3.437891436 | 0.004576254 | 0.018474506 | -2.619630193 |
| ADCY3 | 1.861154819 | 10.27864857 | 3.437703269 | 0.004577885 | 0.018476169 | -2.619986162 |
| C12orf66 | 1.019236187 | 5.067660069 | 3.437185723 | 0.004582376 | 0.018487103 | -2.620965242 |
| CUZD1 | -1.683808771 | 4.190998146 | -3.437109814 | 0.004583035 | 0.018487103 | -2.621108843 |
| AVPR1A | -2.272157893 | 3.81301648 | -3.435147382 | 0.004600105 | 0.018546085 | -2.624821289 |
| ZNF678 | 1.115865023 | 6.302518134 | 3.434113403 | 0.004609125 | 0.018572566 | -2.626777307 |
| C16orf96 | 1.323441834 | 5.698134095 | 3.432930213 | 0.004619468 | 0.018604349 | -2.629015578 |
| CSNK1E | 1.103675556 | 11.11085469 | 3.429824544 | 0.00464673 | 0.018704198 | -2.63489057 |
| OR5AP2 | 1.03276739 | 2.48294031 | 3.429568542 | 0.004648984 | 0.018706922 | -2.635374843 |
| TFPI2 | -1.209884121 | 2.370214509 | -3.429467304 | 0.004649876 | 0.018706922 | -2.635566353 |
| PRKAR2B | -2.383383886 | 9.188847838 | -3.42918236 | 0.004652387 | 0.018712056 | -2.636105374 |
| PPID | -1.315964443 | 9.188156482 | -3.424689722 | 0.004692161 | 0.018847011 | -2.644603836 |
| DSTYK | 1.17840564 | 8.600312433 | 3.422881297 | 0.004708268 | 0.018896679 | -2.648024652 |
| CMTM8 | -2.540533672 | 5.475032308 | -3.421258969 | 0.004722765 | 0.018939812 | -2.651093407 |
| SPI1 | -1.103490193 | 11.97237424 | -3.420404813 | 0.004730416 | 0.018965475 | -2.652709092 |
| ROCK1 | 1.615181623 | 4.319170803 | 3.420174064 | 0.004732485 | 0.018968751 | -2.653145566 |
| GLYATL1B | -1.219012021 | 2.858814497 | -3.419635795 | 0.004737315 | 0.018978069 | -2.654163726 |
| ZMYM6 | 1.138206897 | 6.028974428 | 3.419495675 | 0.004738573 | 0.018978091 | -2.654428767 |
| LARP4B | 1.068269977 | 9.428022765 | 3.418769275 | 0.004745101 | 0.018999213 | -2.655802778 |
| TRPM4 | -2.368554944 | 4.033873279 | -3.416858663 | 0.004762314 | 0.019044762 | -2.659416726 |
| CCDC54 | 1.330406362 | 3.921424669 | 3.416612701 | 0.004764534 | 0.019044762 | -2.659881962 |
| VASN | 1.816009879 | 3.232456692 | 3.414772532 | 0.004781181 | 0.019087718 | -2.66336261 |
| MYO1D | -1.530492074 | 2.934187279 | -3.414646463 | 0.004782323 | 0.019087718 | -2.663601064 |
| TUBG1 | 1.960489737 | 10.76053422 | 3.414013781 | 0.004788061 | 0.019105588 | -2.664797758 |
| SYNE2 | -2.299441392 | 6.933981738 | -3.412473142 | 0.004802063 | 0.019151372 | -2.66771179 |
| USHBP1 | 1.58786021 | 3.515750244 | 3.411498274 | 0.004810945 | 0.019176698 | -2.669555679 |
| FAM84B | -2.675527637 | 4.353145922 | -3.410631355 | 0.004818857 | 0.019203183 | -2.671195379 |
| HELQ | -1.283443932 | 6.847138846 | -3.409832889 | 0.004826156 | 0.019227212 | -2.672705594 |
| PIP4K2C | 1.057917885 | 10.24798802 | 3.40950498 | 0.004829157 | 0.019233974 | -2.673325797 |
| SLITRK1 | 1.383439333 | 3.390042462 | 3.407600035 | 0.004846626 | 0.019288477 | -2.676928752 |
| BRI3BP | 1.069308603 | 8.191695152 | 3.406303611 | 0.004858552 | 0.019315643 | -2.679380734 |
| KANK1 | 1.158298089 | 5.885876792 | 3.405105373 | 0.0048696 | 0.01935449 | -2.681646987 |
| GYPE | 2.262309197 | 6.474995032 | 3.403088729 | 0.004888253 | 0.01942353 | -2.685461056 |
| FDCSP | 1.204502845 | 3.180471071 | 3.401963466 | 0.004898692 | 0.019449708 | -2.687589228 |
| RPUSD2 | -1.083289112 | 10.47589906 | -3.400672127 | 0.0049107 | 0.019490284 | -2.690031467 |
| TTLL3 | 1.089713681 | 7.196704603 | 3.397380407 | 0.004941443 | 0.019588647 | -2.696256785 |
| RNF122 | 1.149786315 | 9.571860676 | 3.397002881 | 0.004944982 | 0.019596103 | -2.696970751 |
| IGF2BP2 | 2.252157515 | 8.449615814 | 3.396625429 | 0.004948522 | 0.019601322 | -2.697684574 |
| TROAP | 1.316105987 | 9.173262856 | 3.395807573 | 0.004956202 | 0.019626611 | -2.699231267 |
| ADAMTSL1 | -1.030883136 | 3.378551702 | -3.394881439 | 0.004964913 | 0.019650834 | -2.700982713 |
| BEX3 | -3.610979609 | 10.80434166 | -3.394533161 | 0.004968193 | 0.019653547 | -2.70164135 |
| RRM2 | 2.965521923 | 6.042657006 | 3.394390912 | 0.004969533 | 0.019653717 | -2.70191036 |
| DNAJC15 | -1.423633344 | 8.601924715 | -3.39311054 | 0.004981612 | 0.019696349 | -2.704331682 |
| TSTD2 | 1.125319323 | 6.987101743 | 3.392751758 | 0.004985003 | 0.019704612 | -2.705010172 |
| TECPR1 | -2.304415016 | 5.501121328 | -3.390995725 | 0.00500163 | 0.019765178 | -2.708330955 |
| TPX2 | 1.974621107 | 8.10349348 | 3.390639068 | 0.005005014 | 0.019773393 | -2.709005412 |
| FRMD4A | 1.029991081 | 3.856954628 | 3.390198408 | 0.005009198 | 0.019774166 | -2.709838719 |
| PPP1R18 | -1.119580347 | 9.690767514 | -3.390118388 | 0.005009958 | 0.019774166 | -2.70999004 |
| DSTN | -1.448528274 | 11.7048007 | -3.390081708 | 0.005010306 | 0.019774166 | -2.710059403 |
| CBLN3 | -2.438403231 | 5.190664443 | -3.390068697 | 0.00501043 | 0.019774166 | -2.710084008 |
| SORT1 | -2.197734138 | 7.8085873 | -3.389629193 | 0.005014608 | 0.019782425 | -2.710915123 |
| PEAK1 | 1.040790414 | 7.063235037 | 3.389573841 | 0.005015134 | 0.019782425 | -2.711019795 |
| OR4K1 | 1.050146006 | 2.722453099 | 3.389256537 | 0.005018153 | 0.01978918 | -2.711619826 |
| BCKDHB | 1.166153645 | 6.12161191 | 3.388108805 | 0.005029087 | 0.019827138 | -2.713790197 |
| GNE | 1.06155693 | 9.331218554 | 3.387924003 | 0.005030849 | 0.019828928 | -2.714139657 |
| TOP1MT | 1.200911146 | 11.25428698 | 3.386837625 | 0.005041225 | 0.019863631 | -2.716193979 |
| LSM14B | 1.05984376 | 6.894359683 | 3.383601156 | 0.005072263 | 0.019950636 | -2.722313946 |
| UNC93A | 1.324101066 | 2.767994599 | 3.383294512 | 0.005075213 | 0.019957061 | -2.72289378 |
| TDRD7 | -1.026804574 | 9.767489809 | -3.382037448 | 0.005087328 | 0.019999508 | -2.725270745 |
| TKTL1 | -2.13543598 | 9.062171189 | -3.380932573 | 0.005098 | 0.020031066 | -2.727359911 |
| TNFAIP3 | -2.156178076 | 9.679292904 | -3.37982557 | 0.005108715 | 0.020067964 | -2.729453075 |
| CRAT | -1.026707076 | 9.423508579 | -3.378571411 | 0.005120882 | 0.020103069 | -2.731824455 |
| MAP3K13 | -2.38718476 | 6.251525126 | -3.378357596 | 0.005122959 | 0.020103069 | -2.732228736 |
| CD79B | -2.326096944 | 9.29738123 | -3.377221399 | 0.005134011 | 0.020141224 | -2.734377038 |
| NDRG3 | -1.111206748 | 8.943053679 | -3.376687817 | 0.00513921 | 0.020151183 | -2.735385915 |
| FKTN | 1.836069506 | 3.125084576 | 3.375927772 | 0.005146625 | 0.020175034 | -2.736822971 |
| SUGCT | -1.185800023 | 3.812330265 | -3.374794934 | 0.005157696 | 0.020202752 | -2.73896486 |
| UGT3A2 | 1.934148615 | 9.622399888 | 3.374627111 | 0.005159338 | 0.020203961 | -2.739282167 |
| RPAP3 | -1.207931622 | 7.549687195 | -3.373655246 | 0.005168858 | 0.020229217 | -2.741119673 |
| JPT2 | 1.142909367 | 7.263055156 | 3.372137102 | 0.005183765 | 0.020278653 | -2.743989989 |
| GAB3 | 1.271513622 | 8.527764314 | 3.36626791 | 0.005241805 | 0.020476243 | -2.75508622 |
| ZNF107 | 1.015324752 | 7.172622185 | 3.366189493 | 0.005242585 | 0.020476243 | -2.755234469 |
| CDR2 | -1.290870365 | 8.796433944 | -3.365546637 | 0.005248983 | 0.020486905 | -2.756449795 |
| HVCN1 | -1.801744485 | 9.042799548 | -3.36520677 | 0.005252369 | 0.020494112 | -2.757092313 |
| NHLRC4 | 1.241463819 | 4.97562258 | 3.360767971 | 0.005296789 | 0.020640858 | -2.765483603 |
| HHAT | 1.585949893 | 7.018396014 | 3.358976063 | 0.005314829 | 0.020704255 | -2.768870962 |
| UMOD | 1.088862823 | 10.92757422 | 3.358880661 | 0.005315791 | 0.020704255 | -2.769051304 |
| SYCE3 | -2.284178665 | 5.23112384 | -3.356892692 | 0.005335881 | 0.020767007 | -2.772809193 |
| NEU4 | 3.009152697 | 4.258575078 | 3.354684957 | 0.005358281 | 0.020837607 | -2.776982389 |
| PIGV | 1.212497067 | 7.405004555 | 3.3535178 | 0.005370162 | 0.020857042 | -2.779188569 |
| ARRB1 | -1.070090528 | 8.785785256 | -3.353246604 | 0.005372926 | 0.02086243 | -2.779701184 |
| GGT5 | 2.225581772 | 7.742398248 | 3.352866707 | 0.005376801 | 0.020872126 | -2.780419262 |
| MAP2K1 | -1.399526622 | 9.719239624 | -3.35179481 | 0.005387749 | 0.020898562 | -2.782445331 |
| ADD3 | -1.394254541 | 11.06806529 | -3.350266201 | 0.005403401 | 0.020943188 | -2.785334613 |
| JMJD1C | 1.348062765 | 8.295837476 | 3.348763972 | 0.005418827 | 0.020992239 | -2.788173971 |
| ZNF829 | -1.505390709 | 2.50349434 | -3.347198294 | 0.005434952 | 0.021038569 | -2.79113319 |
| POLR3G | 1.117828855 | 5.622246559 | 3.345511058 | 0.005452383 | 0.02108988 | -2.794322085 |
| JUND | 1.401919198 | 11.27783519 | 3.344942847 | 0.005458266 | 0.021101862 | -2.795395993 |
| NDUFB1 | -1.104106545 | 13.19982502 | -3.344460797 | 0.005463262 | 0.021110403 | -2.796307053 |
| FKBP7 | 1.103388203 | 8.422624066 | 3.344314051 | 0.005464784 | 0.021110899 | -2.796584396 |
| GPRASP1 | -1.862507272 | 6.368169588 | -3.34303959 | 0.005478019 | 0.021151239 | -2.798993048 |
| VPS41 | 1.248085054 | 7.419069993 | 3.342531779 | 0.005483301 | 0.021166241 | -2.799952766 |
| ARNT2 | 1.564875745 | 5.958688616 | 3.341467827 | 0.005494385 | 0.021198225 | -2.80196352 |
| PTX4 | 1.90974059 | 3.498977682 | 3.340435552 | 0.005505161 | 0.021228987 | -2.803914377 |
| FCGR1A | -1.137986145 | 7.612404468 | -3.337634737 | 0.005534505 | 0.021309604 | -2.809207375 |
| BPIFB2 | -1.294699419 | 7.627294392 | -3.33491103 | 0.005563193 | 0.021382025 | -2.814354437 |
| LACTB2 | -1.343639171 | 6.737915587 | -3.333933922 | 0.005573521 | 0.021416288 | -2.816200852 |
| ERP44 | 1.196490288 | 5.934676527 | 3.332955718 | 0.00558388 | 0.021449364 | -2.81804931 |
| OR1I1 | 1.115061331 | 2.3971699 | 3.332538799 | 0.005588301 | 0.021456755 | -2.818837128 |
| TRIML2 | 1.632827332 | 3.470312871 | 3.329452392 | 0.00562114 | 0.021555527 | -2.824669111 |
| PYCR1 | 1.337315912 | 2.638943022 | 3.328459132 | 0.005631749 | 0.021585284 | -2.826545885 |
| HIST2H3A | 2.804408578 | 11.47837381 | 3.326882359 | 0.005648632 | 0.021629245 | -2.829525149 |
| ZNF117 | 1.386722126 | 6.916845342 | 3.32424657 | 0.005676969 | 0.021687283 | -2.834505221 |
| RBFOX2 | 1.912639022 | 2.966808688 | 3.32259239 | 0.005694826 | 0.021739056 | -2.837630524 |
| LMO2 | 1.478496937 | 12.80793273 | 3.321180496 | 0.005710113 | 0.021791919 | -2.840298 |
| MAPT | -1.129323577 | 2.283139188 | -3.320695316 | 0.005715375 | 0.021801019 | -2.84121463 |
| PRODH | 2.470814588 | 6.627020767 | 3.319835891 | 0.005724709 | 0.021831126 | -2.842838287 |
| TAS2R31 | 1.541488207 | 8.53768242 | 3.318783684 | 0.005736157 | 0.021865923 | -2.844826124 |
| ZBTB37 | 1.107861149 | 6.420267172 | 3.318672111 | 0.005737373 | 0.021865923 | -2.845036906 |
| POMT1 | 1.094664913 | 10.60204356 | 3.318599535 | 0.005738163 | 0.021865923 | -2.845174014 |
| CHRNB4 | 1.138105717 | 2.463308502 | 3.317833595 | 0.005746514 | 0.02189224 | -2.846621005 |
| SERPINI1 | -1.64462014 | 4.918410559 | -3.316952436 | 0.005756137 | 0.021923386 | -2.848285643 |
| PLEKHN1 | -1.373193094 | 4.459822418 | -3.316313668 | 0.005763123 | 0.021938962 | -2.849492351 |
| CMTM6 | -1.116180143 | 10.59716239 | -3.315232164 | 0.00577497 | 0.021972946 | -2.851535411 |
| ERV3-1 | 1.744587489 | 6.097525401 | 3.315101689 | 0.005776401 | 0.021972946 | -2.851781887 |
| CCNB2 | 2.767866884 | 9.299484604 | 3.313459755 | 0.005794438 | 0.02202497 | -2.854883569 |
| MELTF | 1.047667787 | 4.920625742 | 3.312702951 | 0.005802771 | 0.022051113 | -2.856313174 |
| DPH3 | -1.092842109 | 8.126829319 | -3.312449843 | 0.005805561 | 0.022056181 | -2.85679129 |
| NANOS3 | 1.035977611 | 3.620905105 | 3.309898739 | 0.005833753 | 0.022157732 | -2.861610167 |
| CAST | -2.290704885 | 8.137783437 | -3.309728391 | 0.005835641 | 0.022159346 | -2.861931934 |
| SOST | 1.537750719 | 3.80846125 | 3.308668711 | 0.005847396 | 0.022198419 | -2.863933532 |
| CAPN14 | 1.212723227 | 2.12758938 | 3.308423766 | 0.005850116 | 0.022200336 | -2.864396198 |
| RAB7B | 3.984239866 | 6.017145279 | 3.307467046 | 0.005860754 | 0.022226856 | -2.866203276 |
| ITSN2 | 1.200079917 | 8.395018907 | 3.307021989 | 0.00586571 | 0.022240083 | -2.867043902 |
| MACC1 | 1.75117498 | 2.693619536 | 3.305538455 | 0.005882259 | 0.022283121 | -2.869845956 |
| OPTN | -2.604587358 | 7.983689002 | -3.305495806 | 0.005882735 | 0.022283121 | -2.86992651 |
| SMIM19 | -1.056534215 | 10.11114469 | -3.305477125 | 0.005882944 | 0.022283121 | -2.869961792 |
| PRSS42 | 1.453127215 | 2.990833665 | 3.30497376 | 0.00588857 | 0.022287717 | -2.870912515 |
| RTN4RL1 | 1.129943674 | 5.772683523 | 3.304197062 | 0.005897263 | 0.02230947 | -2.872379474 |
| MTRNR2L7 | 1.025162192 | 10.75700858 | 3.301572055 | 0.005926736 | 0.022403327 | -2.877337197 |
| LRIF1 | -1.482583441 | 7.178799625 | -3.300786754 | 0.005935582 | 0.022420842 | -2.878820309 |
| ABCC1 | 1.239878449 | 7.722306463 | 3.300425925 | 0.005939651 | 0.022430599 | -2.879501759 |
| PLEKHA5 | 2.12419442 | 4.653322772 | 3.300185416 | 0.005942364 | 0.022430599 | -2.879955975 |
| BARX2 | 1.485547674 | 3.582007682 | 3.300164008 | 0.005942606 | 0.022430599 | -2.879996406 |
| MED20 | 1.478099963 | 4.247173387 | 3.300030399 | 0.005944114 | 0.022430703 | -2.880248734 |
| CILP2 | 2.253528401 | 3.238905174 | 3.299454792 | 0.005950616 | 0.022438472 | -2.881335791 |
| KIFC2 | 1.229974289 | 6.034588378 | 3.298771471 | 0.005958344 | 0.022456434 | -2.882626252 |
| METTL13 | 1.411624472 | 10.13737836 | 3.29827609 | 0.005963952 | 0.022471982 | -2.883561779 |
| ALOX12 | -1.040583385 | 6.882280762 | -3.297322068 | 0.005974768 | 0.022501545 | -2.885363419 |
| GOLGA8N | 2.207886911 | 8.304504415 | 3.295688329 | 0.005993337 | 0.022543458 | -2.88844861 |
| ZNF84 | 1.118882023 | 7.341636268 | 3.291380426 | 0.006042578 | 0.022706126 | -2.896583279 |
| ZNF30 | -1.641831527 | 5.452677312 | -3.290979241 | 0.006047185 | 0.02271217 | -2.897340807 |
| PIN4 | -1.145762666 | 6.226700523 | -3.287673865 | 0.006085272 | 0.022832579 | -2.90358187 |
| KIF24 | 1.796327008 | 4.454350629 | 3.284135904 | 0.006126307 | 0.022958119 | -2.910261629 |
| SMIM3 | 1.906835818 | 12.89036581 | 3.283164661 | 0.00613762 | 0.022989143 | -2.912095275 |
| GPR137B | -1.227472159 | 7.082775425 | -3.282558824 | 0.006144688 | 0.023009928 | -2.91323904 |
| TMEM9 | -1.026203831 | 7.50636707 | -3.282202089 | 0.006148854 | 0.023015241 | -2.913912515 |
| USP16 | 1.192027235 | 10.2558809 | 3.282177154 | 0.006149145 | 0.023015241 | -2.913959589 |
| SCRN1 | -1.491830563 | 4.512674881 | -3.281951068 | 0.006151787 | 0.023019441 | -2.914386413 |
| SLAMF1 | -1.307107979 | 8.162092042 | -3.281174525 | 0.006160868 | 0.023024992 | -2.915852413 |
| ZNF587B | 1.590638099 | 6.24657091 | 3.280919306 | 0.006163856 | 0.023030477 | -2.916334225 |
| RETN | -2.26515947 | 10.8105983 | -3.278966311 | 0.006186768 | 0.023081929 | -2.920021074 |
| MTSS1 | -1.967919341 | 5.813008078 | -3.277443279 | 0.006204694 | 0.023138494 | -2.922896137 |
| NEK2 | 2.194898335 | 3.302798652 | 3.277144403 | 0.006208218 | 0.023141916 | -2.923460321 |
| CELSR1 | 1.169447231 | 4.663568941 | 3.277081907 | 0.006208956 | 0.023141916 | -2.923578293 |
| IGKV1D-33 | -2.506446964 | 5.510668845 | -3.276707789 | 0.00621337 | 0.023148447 | -2.924284504 |
| REEP3 | -1.872168232 | 5.439492002 | -3.275561076 | 0.006226921 | 0.023186968 | -2.926449087 |
| PIP4K2A | -1.423383109 | 11.26909393 | -3.273698972 | 0.006248989 | 0.023222575 | -2.929963954 |
| TXNIP | -1.746123745 | 12.42884143 | -3.270861632 | 0.006282767 | 0.023342374 | -2.935319386 |
| SOCS1 | -1.673070651 | 8.265932237 | -3.269900985 | 0.006294245 | 0.023373554 | -2.937132516 |
| PRKD1 | 1.02728625 | 2.983843534 | 3.269654302 | 0.006297195 | 0.023378781 | -2.9375981 |
| ADAMTS3 | 3.577839005 | 3.572474581 | 3.269458618 | 0.006299537 | 0.023381746 | -2.937967429 |
| RBMS2 | 1.098967585 | 6.57375166 | 3.268265486 | 0.006313834 | 0.023423335 | -2.940219278 |
| HES5 | 1.068282763 | 5.768034476 | 3.267961729 | 0.006317479 | 0.02343112 | -2.940792561 |
| SERTAD2 | 1.123772196 | 9.169248148 | 3.267071528 | 0.006328173 | 0.023459299 | -2.942472624 |
| NFIX | 1.901581148 | 5.648265207 | 3.266877393 | 0.006330508 | 0.023462213 | -2.942839008 |
| IFT81 | 1.137691802 | 4.009696082 | 3.266388271 | 0.006336394 | 0.023478285 | -2.943762102 |
| POLR2J2 | -1.009888631 | 11.36298277 | -3.265389633 | 0.006348429 | 0.023517125 | -2.945646749 |
| TNIK | -2.519313662 | 6.939695692 | -3.26516522 | 0.006351136 | 0.023521404 | -2.946070259 |
| APBA2 | -2.29176026 | 5.666486288 | -3.263256533 | 0.006374212 | 0.023601095 | -2.949672229 |
| FZD2 | -1.049908398 | 5.909791196 | -3.261454392 | 0.006396076 | 0.023664232 | -2.953072988 |
| POU5F1B | 1.069268357 | 6.622619187 | 3.261336332 | 0.006397511 | 0.023664232 | -2.953295771 |
| SPRED2 | 1.679586244 | 2.939634782 | 3.259272949 | 0.006422644 | 0.02373402 | -2.957189329 |
| PECAM1 | -1.365662978 | 10.50385684 | -3.257471705 | 0.006444665 | 0.023786387 | -2.960588085 |
| CA13 | -2.497394654 | 5.267600726 | -3.256322098 | 0.006458759 | 0.0238326 | -2.962757195 |
| CDC42EP3 | -1.938007501 | 4.583241554 | -3.255778708 | 0.006465432 | 0.023844879 | -2.963782458 |
| DPH5 | -1.146679182 | 10.10228036 | -3.253495617 | 0.006493543 | 0.023920173 | -2.968090032 |
| FAM110A | -1.120309723 | 8.074132766 | -3.251618202 | 0.006516752 | 0.02399448 | -2.971632031 |
| TRIR | -1.787081733 | 8.57334002 | -3.249335798 | 0.006545079 | 0.024080742 | -2.975937884 |
| CDC42EP4 | -1.470278551 | 5.897957478 | -3.249156725 | 0.006547307 | 0.02408309 | -2.976275702 |
| OR7G3 | 1.112674527 | 3.533456234 | 3.248434049 | 0.006556305 | 0.024104484 | -2.977639004 |
| KCTD12 | -1.916019664 | 8.081538673 | -3.248043507 | 0.006561173 | 0.024104584 | -2.978375739 |
| ADCK2 | -1.645900362 | 6.354232874 | -3.24803712 | 0.006561253 | 0.024104584 | -2.978387788 |
| HMMR | 1.979466562 | 5.418491653 | 3.247921376 | 0.006562696 | 0.024104584 | -2.97860613 |
| WDFY3 | -2.151285356 | 5.367702535 | -3.246105552 | 0.006585382 | 0.024165019 | -2.982031472 |
| ERVFRD-1 | 1.318347313 | 2.481708225 | 3.245212466 | 0.006596569 | 0.024199666 | -2.98371612 |
| PABPC4L | 1.160803233 | 3.74109629 | 3.244005839 | 0.006611714 | 0.024249352 | -2.985992147 |
| CD22 | -2.733797757 | 6.690728474 | -3.242471551 | 0.006631021 | 0.024302517 | -2.988886137 |
| HLA-DQB2 | -1.206742861 | 11.00673272 | -3.242167529 | 0.006634854 | 0.024306382 | -2.989459573 |
| TRPM1 | 1.016610319 | 2.024349938 | 3.242055312 | 0.006636269 | 0.024306382 | -2.989671232 |
| PEX5 | 1.024091559 | 5.990231987 | 3.240873133 | 0.006651196 | 0.024352894 | -2.991900971 |
| COL23A1 | 2.087185506 | 7.926909343 | 3.239920956 | 0.006663244 | 0.02439111 | -2.993696849 |
| MORN2 | -1.050701902 | 8.031034207 | -3.239449711 | 0.006669214 | 0.024407069 | -2.994585637 |
| PNISR | 1.018938322 | 9.180792928 | 3.237939999 | 0.006688378 | 0.024471291 | -2.997432946 |
| C11orf42 | 1.219982596 | 9.626070596 | 3.236970624 | 0.006700712 | 0.024498671 | -2.999261126 |
| ONECUT1 | 1.182988201 | 3.21047488 | 3.236067167 | 0.006712228 | 0.024534855 | -3.000964948 |
| CFAP70 | 1.405131204 | 4.796459673 | 3.235204139 | 0.006723248 | 0.02455144 | -3.002592489 |
| TLR2 | -1.725088594 | 10.65650071 | -3.233873086 | 0.006740278 | 0.02459488 | -3.005102583 |
| PPP6R3 | 1.09307265 | 11.93757226 | 3.233228636 | 0.00674854 | 0.024610351 | -3.006317853 |
| PLEKHF2 | -1.05981602 | 9.823505174 | -3.233182936 | 0.006749126 | 0.024610351 | -3.006404032 |
| TMA7 | -1.021922903 | 13.93187408 | -3.232013287 | 0.006764147 | 0.024635479 | -3.008609641 |
| HIST1H1T | 1.989445562 | 2.976492483 | 3.230144594 | 0.006788215 | 0.024705321 | -3.012133299 |
| IL15 | -1.390569878 | 5.026649809 | -3.228141663 | 0.006814108 | 0.024775751 | -3.01590989 |
| CIR1 | -1.086495436 | 10.04444892 | -3.227900427 | 0.006817234 | 0.024781167 | -3.016364735 |
| RAD51AP2 | 1.033125686 | 10.23493423 | 3.227176177 | 0.006826625 | 0.024803404 | -3.017730274 |
| PANX1 | -1.145493825 | 8.142258973 | -3.226576804 | 0.006834407 | 0.024819775 | -3.018860344 |
| SNX32 | 1.167237996 | 11.47964509 | 3.225621174 | 0.006846833 | 0.024858942 | -3.020662069 |
| HRH1 | -1.732537618 | 5.449146014 | -3.224092838 | 0.006866753 | 0.024925293 | -3.023543469 |
| ATP6V1B1 | 1.113243972 | 3.154485107 | 3.222853918 | 0.006882943 | 0.024960142 | -3.025879142 |
| RIC3 | -1.643293672 | 3.666841465 | -3.221064018 | 0.006906402 | 0.025033228 | -3.029253411 |
| CCDC42 | -1.588847411 | 4.22200136 | -3.219617473 | 0.006925419 | 0.025096155 | -3.031980277 |
| MORC4 | -2.710208977 | 5.404647324 | -3.219459361 | 0.006927501 | 0.025097696 | -3.032278327 |
| DENND1B | 1.48495163 | 8.199776061 | 3.2163072 | 0.006969136 | 0.025236467 | -3.038220033 |
| HSBP1L1 | -2.279275701 | 7.74021609 | -3.215782335 | 0.006976093 | 0.025252109 | -3.039209334 |
| CRABP1 | 3.36744692 | 3.530731234 | 3.215602027 | 0.006978484 | 0.025252109 | -3.039549188 |
| CENPM | 1.352655725 | 8.620997055 | 3.215478577 | 0.006980122 | 0.025252109 | -3.03978187 |
| MAPK14 | 1.302330302 | 8.98748174 | 3.212400924 | 0.007021081 | 0.025379551 | -3.045582502 |
| CHAF1B | 1.378108123 | 9.116818046 | 3.21220536 | 0.007023691 | 0.025379551 | -3.045951075 |
| SLC45A2 | -1.149701532 | 6.433236866 | -3.21220283 | 0.007023725 | 0.025379551 | -3.045955842 |
| RGL4 | -1.998354141 | 10.02867893 | -3.211478681 | 0.007033401 | 0.025408454 | -3.047320608 |
| MPZL3 | -2.332208826 | 8.013119415 | -3.208528126 | 0.007072964 | 0.02552703 | -3.052881075 |
| RGS20 | 1.722206388 | 2.386365659 | 3.208144868 | 0.007078119 | 0.025533472 | -3.053603309 |
| CD38 | 1.928143308 | 6.604860692 | 3.20467887 | 0.007124913 | 0.025653411 | -3.060134488 |
| TMEM255A | -1.558669414 | 2.357953595 | -3.203529741 | 0.007140496 | 0.025691201 | -3.062299713 |
| ALG14 | -1.527497254 | 7.128076985 | -3.202819128 | 0.007150149 | 0.025713721 | -3.063638637 |
| KLF2 | -1.303946999 | 11.71013303 | -3.202419826 | 0.007155579 | 0.025721038 | -3.064390983 |
| CKAP2 | 1.221886774 | 7.437260346 | 3.20117605 | 0.007172519 | 0.025763594 | -3.066734388 |
| KRT10 | -1.083201446 | 12.49718326 | -3.2007475 | 0.007178366 | 0.025778483 | -3.067541802 |
| DIP2B | 1.020248793 | 7.621799657 | 3.199793249 | 0.007191401 | 0.025819173 | -3.069339632 |
| FRRS1 | 2.26377437 | 4.542370642 | 3.197936679 | 0.007216829 | 0.025879807 | -3.072837307 |
| FAM83B | 1.507006206 | 3.401562321 | 3.197481405 | 0.007223079 | 0.025896088 | -3.073694988 |
| BTC | 2.581462079 | 3.207806603 | 3.195792802 | 0.007246306 | 0.025973214 | -3.076876011 |
| ZNF749 | 1.718613449 | 3.967463602 | 3.195456745 | 0.007250937 | 0.025983668 | -3.077509063 |
| ZC2HC1C | 1.222636666 | 3.375805707 | 3.194637828 | 0.007262235 | 0.026011851 | -3.079051683 |
| SUCLG2 | -2.143839725 | 5.205814652 | -3.193878571 | 0.007272727 | 0.026030966 | -3.080481885 |
| MAFB | -2.383353525 | 11.00011996 | -3.191486488 | 0.007305879 | 0.026118775 | -3.084987606 |
| IL12A | -1.364665353 | 4.024995238 | -3.189777985 | 0.00732965 | 0.026179049 | -3.088205537 |
| CCL23 | 2.638279668 | 5.879737272 | 3.189590529 | 0.007332263 | 0.026182209 | -3.088558596 |
| PUS7 | 1.035905188 | 5.208063584 | 3.188626917 | 0.007345709 | 0.02621169 | -3.090373456 |
| ABCA6 | -1.659639388 | 3.671439303 | -3.18701259 | 0.007368291 | 0.026282002 | -3.093413749 |
| POLR2H | -1.115619268 | 9.457934372 | -3.186970312 | 0.007368883 | 0.026282002 | -3.09349337 |
| MLKL | -1.435158111 | 10.04948611 | -3.185632482 | 0.007387651 | 0.02634274 | -3.096012813 |
| STAT4 | -2.194419456 | 7.898498139 | -3.184714001 | 0.007400564 | 0.026376369 | -3.097742465 |
| STK32B | 3.03960415 | 4.021497438 | 3.184349332 | 0.007405697 | 0.026388457 | -3.098429183 |
| PITPNA | -1.154075817 | 10.28917072 | -3.183980009 | 0.0074109 | 0.02639088 | -3.099124659 |
| NECAB2 | -1.588298716 | 2.946353183 | -3.183930114 | 0.007411603 | 0.02639088 | -3.099218615 |
| TMCO2 | 1.371265214 | 8.260075441 | 3.183316802 | 0.007420251 | 0.026415465 | -3.100373528 |
| C4BPB | -1.371067558 | 2.763598642 | -3.183031595 | 0.007424276 | 0.026423586 | -3.100910586 |
| PEX11G | 1.094669426 | 4.503560061 | 3.182718444 | 0.007428698 | 0.026426005 | -3.10150026 |
| RGS19 | -1.306762526 | 12.74637601 | -3.182536091 | 0.007431274 | 0.026426005 | -3.101843634 |
| AKAP5 | -1.790731894 | 3.297583059 | -3.180343247 | 0.007462324 | 0.026506745 | -3.105972642 |
| C1orf216 | -1.430002936 | 4.522753509 | -3.180268317 | 0.007463387 | 0.026506745 | -3.106113726 |
| BCL7A | 2.006439159 | 8.679192163 | 3.179438728 | 0.007475169 | 0.026530488 | -3.107675719 |
| TNKS1BP1 | 1.448436896 | 3.309287316 | 3.17942773 | 0.007475325 | 0.026530488 | -3.107696426 |
| PATE3 | 1.14938303 | 8.2939212 | 3.178859421 | 0.007483408 | 0.026546736 | -3.108766446 |
| SLC10A6 | 1.028192318 | 2.465645858 | 3.177667292 | 0.00750039 | 0.026588304 | -3.111010935 |
| CHN2 | -1.343860706 | 9.281819692 | -3.177133732 | 0.007508003 | 0.026602844 | -3.112015473 |
| AKAP12 | 1.684350041 | 5.784858596 | 3.176789497 | 0.007512919 | 0.026614039 | -3.112663556 |
| CENPI | 1.264945498 | 4.071375281 | 3.175884516 | 0.007525859 | 0.026651389 | -3.114367313 |
| PRR22 | 1.080990677 | 5.396507788 | 3.175806093 | 0.007526981 | 0.026651389 | -3.114514954 |
| ZNF703 | -2.279674023 | 5.016450015 | -3.174142989 | 0.007550822 | 0.026729557 | -3.117645847 |
| SRSF12 | 2.545865833 | 2.777226521 | 3.170041002 | 0.007609949 | 0.02690115 | -3.12536737 |
| TBL1XR1 | 1.061967563 | 9.378324689 | 3.169490728 | 0.007617916 | 0.026923032 | -3.126403119 |
| ZNF286B | -1.290293094 | 7.352576439 | -3.168669297 | 0.007629824 | 0.026953459 | -3.127949217 |
| ADGRG5 | 1.03440193 | 7.974752008 | 3.166851591 | 0.007656242 | 0.027026961 | -3.131370358 |
| PLGRKT | -1.071399379 | 9.754691261 | -3.165904055 | 0.007670049 | 0.027069395 | -3.133153653 |
| CDKN2A | 1.740432899 | 9.442929418 | 3.165631457 | 0.007674026 | 0.027077124 | -3.133666683 |
| OR5H6 | 1.13997112 | 6.202634106 | 3.161519559 | 0.007734265 | 0.027257933 | -3.141404694 |
| LIN28B | 1.026872713 | 2.492520553 | 3.16098272 | 0.007742165 | 0.027279428 | -3.142414873 |
| CASC10 | 2.470594865 | 5.667328455 | 3.15999605 | 0.007756705 | 0.027311605 | -3.144271454 |
| TSTD3 | -1.242337314 | 7.928064825 | -3.158718522 | 0.007775572 | 0.027369571 | -3.146675242 |
| TES | -1.756889468 | 8.585625538 | -3.158161517 | 0.007783812 | 0.027387955 | -3.147723267 |
| HYAL3 | 1.571048631 | 8.366821769 | 3.157689085 | 0.007790808 | 0.027406207 | -3.148612149 |
| C9orf43 | 2.461132583 | 4.733843243 | 3.154918667 | 0.007831961 | 0.027519026 | -3.153824408 |
| VTI1B | -1.170551394 | 5.451652005 | -3.153810417 | 0.007848484 | 0.027564298 | -3.155909328 |
| TLE1 | -1.178389197 | 6.531228587 | -3.15355558 | 0.007852288 | 0.02756488 | -3.156388733 |
| RFPL2 | -1.883158103 | 4.390683656 | -3.149326828 | 0.007915689 | 0.027761712 | -3.164343368 |
| FANCC | 1.053430426 | 6.390233042 | 3.148729383 | 0.007924687 | 0.027775622 | -3.165467117 |
| SLC16A6 | -1.228720164 | 4.6369535 | -3.147897849 | 0.007937228 | 0.027805074 | -3.167031132 |
| S100A10 | -1.971129039 | 12.93614505 | -3.147588328 | 0.007941902 | 0.027815014 | -3.167613292 |
| ZNF408 | -1.176520377 | 5.49409389 | -3.147250361 | 0.007947008 | 0.027822307 | -3.168248946 |
| SIGLEC10 | -2.605321207 | 8.001792911 | -3.147207422 | 0.007947657 | 0.027822307 | -3.168329706 |
| LMBR1 | 1.7436469 | 3.168627768 | 3.14618678 | 0.007963098 | 0.027863485 | -3.170249301 |
| MEF2A | 1.325504169 | 9.516287053 | 3.145371928 | 0.007975447 | 0.027893812 | -3.171781802 |
| ALS2 | 1.683625917 | 5.526356222 | 3.143968538 | 0.00799676 | 0.027955448 | -3.174421068 |
| KRT79 | 3.687998634 | 5.679191901 | 3.143812902 | 0.007999127 | 0.027957273 | -3.174713755 |
| SPART | 1.604344786 | 4.84606305 | 3.140844236 | 0.008044414 | 0.02809611 | -3.180296272 |
| MRPL14 | -1.263768871 | 9.635934304 | -3.13972253 | 0.008061593 | 0.028149618 | -3.182405463 |
| UBA7 | 1.028812889 | 10.75649747 | 3.137366667 | 0.008097792 | 0.02826921 | -3.186835015 |
| TBC1D4 | -3.001665911 | 5.864071109 | -3.136802273 | 0.008106488 | 0.028286825 | -3.187896146 |
| PLAG1 | -1.356565345 | 4.654646596 | -3.134913913 | 0.008135652 | 0.02837552 | -3.191446341 |
| POLR2K | -1.138103192 | 10.05603549 | -3.133228513 | 0.00816177 | 0.028460064 | -3.194614756 |
| AJUBA | 1.161359061 | 2.07214503 | 3.132539972 | 0.008172464 | 0.028490797 | -3.195909101 |
| MOB3A | -1.113030168 | 10.71142081 | -3.130991422 | 0.008196567 | 0.028550422 | -3.198820001 |
| RTP2 | -1.251383643 | 2.551493254 | -3.129069997 | 0.008226573 | 0.028633312 | -3.202431586 |
| AC067752.1 | 1.031382543 | 4.938257222 | 3.128745405 | 0.008231653 | 0.028640531 | -3.203041676 |
| DMRTA1 | 1.41721679 | 7.557640673 | 3.128653064 | 0.008233098 | 0.028640531 | -3.203215236 |
| IGF2 | 1.04279653 | 4.953336963 | 3.128575168 | 0.008234318 | 0.028640531 | -3.203361643 |
| TNNT2 | 1.53890715 | 2.75920834 | 3.126613226 | 0.008265099 | 0.028727807 | -3.207049051 |
| BHLHA15 | 1.311802673 | 7.944744732 | 3.124507542 | 0.008298263 | 0.028829851 | -3.211006314 |
| SPATA31D1 | -1.33898611 | 2.57053038 | -3.122348364 | 0.008332408 | 0.028915325 | -3.215063783 |
| CNN3 | -1.804650083 | 3.036025609 | -3.121192205 | 0.008350749 | 0.028965704 | -3.21723627 |
| PXK | 1.37556845 | 10.11822937 | 3.118764488 | 0.008389393 | 0.029049811 | -3.221797771 |
| SLC35D3 | -2.002037582 | 3.957327297 | -3.118719501 | 0.008390111 | 0.029049811 | -3.221882295 |
| TSPO2 | 1.998807811 | 8.271044608 | 3.118498009 | 0.008393646 | 0.029054632 | -3.222298441 |
| C12orf60 | -1.437200176 | 3.971780786 | -3.115563917 | 0.008440613 | 0.029203865 | -3.227810759 |
| TFCP2L1 | -1.525141823 | 2.725715501 | -3.115324735 | 0.008444453 | 0.029210481 | -3.228260087 |
| GRM8 | 1.043817733 | 3.234313386 | 3.112852579 | 0.008484248 | 0.029341438 | -3.23290403 |
| INSYN2B | 1.195258307 | 3.163230715 | 3.110091809 | 0.00852891 | 0.029449102 | -3.238089605 |
| NUP210L | 1.120714424 | 2.321179056 | 3.11008718 | 0.008528986 | 0.029449102 | -3.2380983 |
| NIPAL4 | -1.815297463 | 4.776359543 | -3.107244135 | 0.008575225 | 0.029573024 | -3.243437821 |
| TMTC1 | -1.74153285 | 4.116155939 | -3.107160636 | 0.008576587 | 0.029573024 | -3.24359463 |
| HAND2 | 1.059440079 | 2.386106107 | 3.101699227 | 0.00866613 | 0.029847816 | -3.253849969 |
| OR1M1 | 1.002544867 | 3.888712873 | 3.101358233 | 0.008671752 | 0.02986039 | -3.254490209 |
| CCNJ | 1.100966117 | 7.82608019 | 3.100536812 | 0.008685309 | 0.02989106 | -3.256032443 |
| G2E3 | 1.293521948 | 4.17388135 | 3.100460025 | 0.008686578 | 0.02989106 | -3.256176608 |
| FGF22 | 1.132244526 | 4.146858892 | 3.099454344 | 0.008703207 | 0.029927216 | -3.258064718 |
| PSMA8 | -2.612579823 | 5.071793548 | -3.098252089 | 0.008723129 | 0.02996919 | -3.260321785 |
| NKTR | 1.674476804 | 10.11847064 | 3.097620171 | 0.008733618 | 0.029998425 | -3.261508078 |
| CPOX | 2.057598917 | 8.830458779 | 3.096101 | 0.008758887 | 0.03006477 | -3.264359876 |
| ANKS6 | -1.878637258 | 8.423028457 | -3.095642971 | 0.00876652 | 0.030070532 | -3.265219656 |
| PBLD | 1.500625168 | 9.165898974 | 3.093228277 | 0.008806869 | 0.030195265 | -3.269752085 |
| ADTRP | -1.612614405 | 4.810754882 | -3.091686725 | 0.008832726 | 0.030263371 | -3.272645373 |
| YBEY | -1.172356622 | 8.707197314 | -3.089369506 | 0.008871735 | 0.030369557 | -3.276994134 |
| ATP5MPL | -1.174515841 | 12.02549815 | -3.08742919 | 0.008904532 | 0.03047494 | -3.280635232 |
| NPDC1 | -1.311022735 | 8.145424773 | -3.086768421 | 0.008915728 | 0.030492595 | -3.28187513 |
| TNFSF4 | -1.984519992 | 7.514133791 | -3.086563383 | 0.008919205 | 0.030497603 | -3.282259865 |
| POC1A | 1.017554439 | 8.929573382 | 3.084191312 | 0.008959531 | 0.030607858 | -3.286710605 |
| RETREG1 | -3.005485473 | 7.002159661 | -3.081110245 | 0.009012181 | 0.030759981 | -3.292490972 |
| TUBA3C | -2.369749813 | 7.97414572 | -3.08026009 | 0.009026763 | 0.030788944 | -3.294085807 |
| GALK1 | -1.140553538 | 8.67666224 | -3.080072381 | 0.009029986 | 0.030793004 | -3.29443793 |
| COL6A5 | 1.081948074 | 3.423786527 | 3.079127216 | 0.009046231 | 0.030834521 | -3.296210916 |
| ULK2 | -1.475017073 | 7.373361 | -3.078596297 | 0.009055369 | 0.030851787 | -3.297206806 |
| NABP2 | -1.263995972 | 7.052650609 | -3.07737276 | 0.009076463 | 0.030909754 | -3.299501816 |
| PBOV1 | 1.12150243 | 3.949306118 | 3.076764973 | 0.00908696 | 0.030938547 | -3.300641807 |
| ASPA | 1.047724297 | 2.48437073 | 3.076336544 | 0.009094367 | 0.030956807 | -3.301445369 |
| CAMLG | -1.676104075 | 5.658276359 | -3.075169514 | 0.009114572 | 0.031018617 | -3.303634176 |
| FTO | 1.007763317 | 4.304901876 | 3.074657653 | 0.009123448 | 0.031041852 | -3.304594155 |
| RNASE2 | -1.262542227 | 10.5919853 | -3.07446344 | 0.009126818 | 0.031046346 | -3.304958388 |
| OR56B1 | 1.450844191 | 2.774344364 | 3.07104006 | 0.009186428 | 0.031224489 | -3.311378218 |
| MMEL1 | -1.552957175 | 4.566570341 | -3.070914663 | 0.009188619 | 0.031224489 | -3.311613356 |
| PFKP | 1.595887691 | 11.43189096 | 3.070864708 | 0.009189492 | 0.031224489 | -3.311707028 |
| HLA-DRB5 | -1.279764101 | 12.61445254 | -3.070270721 | 0.009199878 | 0.031252769 | -3.312820819 |
| DUOX1 | 1.353306452 | 3.269060259 | 3.068028571 | 0.009239187 | 0.031358183 | -3.317024833 |
| TRAF1 | -1.823065036 | 6.322589896 | -3.067620154 | 0.009246366 | 0.031368493 | -3.317790567 |
| CDYL2 | 1.307424031 | 6.776158461 | 3.064432486 | 0.009302584 | 0.031530974 | -3.323766592 |
| CHIC1 | -1.004112877 | 5.518928993 | -3.064184005 | 0.009306981 | 0.031534312 | -3.324232391 |
| IL34 | -1.558906521 | 4.541058211 | -3.06402401 | 0.009309813 | 0.031534312 | -3.324532313 |
| RHOXF1 | -1.755715359 | 4.705060645 | -3.063648639 | 0.009316461 | 0.031549775 | -3.325235964 |
| TBC1D2 | -1.418954103 | 9.179590198 | -3.063470503 | 0.009319617 | 0.031553412 | -3.325569885 |
| FAM111B | 1.259256141 | 6.629521171 | 3.062456125 | 0.009337611 | 0.031588124 | -3.32747131 |
| NOV | -1.333227182 | 5.308380419 | -3.060455862 | 0.009373196 | 0.031677555 | -3.331220499 |
| KNCN | 1.230264511 | 5.69776244 | 3.06034896 | 0.009375101 | 0.031677555 | -3.33142086 |
| YOD1 | 1.508436174 | 9.535011015 | 3.059478206 | 0.009390637 | 0.031708835 | -3.333052843 |
| PNMA3 | 1.199677753 | 2.804644675 | 3.058669514 | 0.009405089 | 0.031743484 | -3.334568449 |
| C22orf46 | 1.082241481 | 9.951599779 | 3.058321353 | 0.009411317 | 0.031757432 | -3.335220936 |
| RNF144B | -2.973949483 | 7.329585224 | -3.058011216 | 0.009416869 | 0.031761764 | -3.335802153 |
| KHDRBS3 | 1.333361586 | 4.516479826 | 3.057995403 | 0.009417152 | 0.031761764 | -3.335831787 |
| CD300LF | -1.910335029 | 8.908739808 | -3.056581471 | 0.009442506 | 0.031834313 | -3.338481485 |
| ZNF605 | 1.521284032 | 5.096527938 | 3.055611455 | 0.009459938 | 0.031878899 | -3.34029919 |
| ZNF121 | 1.369642017 | 8.005334466 | 3.053251319 | 0.009502488 | 0.03198175 | -3.344721487 |
| SPICE1 | 1.663093771 | 4.060574784 | 3.050109091 | 0.009559434 | 0.032154148 | -3.350608479 |
| FOXC1 | 1.993825244 | 6.656791068 | 3.050038484 | 0.009560718 | 0.032154148 | -3.350740752 |
| MAK | -1.709478613 | 4.436176895 | -3.048183871 | 0.009594493 | 0.032246264 | -3.354214971 |
| RAB27B | -2.395624619 | 6.520495504 | -3.046424802 | 0.009626638 | 0.032339953 | -3.35750993 |
| CAMSAP1 | 1.4570203 | 4.479643795 | 3.043905453 | 0.009672863 | 0.032452069 | -3.362228513 |
| OCIAD2 | -1.5373669 | 12.20400199 | -3.042091251 | 0.009706287 | 0.032542587 | -3.365626053 |
| OR4C3 | 1.260587833 | 3.459699289 | 3.040060727 | 0.009743833 | 0.032632364 | -3.369428361 |
| TENT4A | 1.052804346 | 7.998375192 | 3.039174151 | 0.009760272 | 0.032680194 | -3.371088427 |
| NMU | 2.988827581 | 6.33273549 | 3.037965253 | 0.009782732 | 0.03274816 | -3.373351907 |
| SUSD4 | -1.431309561 | 2.227767225 | -3.03757456 | 0.009790002 | 0.03275802 | -3.374083392 |
| ZNF429 | 1.149311036 | 11.91849324 | 3.036723743 | 0.009805851 | 0.03280381 | -3.375676311 |
| NUB1 | -1.0163899 | 6.460013222 | -3.036466924 | 0.009810641 | 0.032812586 | -3.37615712 |
| ITGA8 | 1.074516147 | 4.738246303 | 3.036148611 | 0.00981658 | 0.032817879 | -3.37675305 |
| P3H2 | -1.409547989 | 6.355705188 | -3.036077803 | 0.009817902 | 0.032817879 | -3.376885611 |
| KISS1R | -1.179791478 | 2.239357227 | -3.032640937 | 0.009882266 | 0.032986609 | -3.383319322 |
| CCDC7 | -1.115059175 | 5.466604011 | -3.032393169 | 0.009886923 | 0.032994882 | -3.383783094 |
| KCNH2 | 3.57829411 | 6.804895142 | 3.032076613 | 0.009892875 | 0.033007476 | -3.384375615 |
| SMC5 | -1.119300426 | 5.25651128 | -3.03080507 | 0.00991682 | 0.033059635 | -3.386755568 |
| TAS2R20 | 1.436325962 | 2.618094365 | 3.03023178 | 0.009927635 | 0.033087021 | -3.387828549 |
| TMEM41B | 1.036272329 | 8.892140739 | 3.028833162 | 0.009954068 | 0.03316053 | -3.390446103 |
| ASCC3 | 1.377232984 | 9.151888716 | 3.026345938 | 0.01000125 | 0.03328843 | -3.39510057 |
| FEN1 | 1.304723227 | 6.870810186 | 3.023263459 | 0.010060032 | 0.033426808 | -3.40086817 |
| ZNF177 | 1.85407921 | 5.052570012 | 3.023221382 | 0.010060836 | 0.033426808 | -3.400946894 |
| HSPB3 | -1.176444801 | 5.354970771 | -3.023141586 | 0.010062363 | 0.033426808 | -3.401096188 |
| PPIP5K1 | 1.031552935 | 5.740899358 | 3.018666439 | 0.010148336 | 0.033587414 | -3.409467969 |
| PTH1R | 1.467960542 | 4.11528572 | 3.018420649 | 0.010153079 | 0.033587414 | -3.40992772 |
| XAGE1B | -1.184588971 | 2.642186159 | -3.017580077 | 0.010169316 | 0.033625083 | -3.411499972 |
| OR11H6 | 1.227625656 | 2.757027243 | 3.015369851 | 0.010212134 | 0.033745703 | -3.415633778 |
| SLC34A2 | 1.641119541 | 5.976555504 | 3.015220112 | 0.010215042 | 0.03374795 | -3.415913819 |
| C2orf15 | -1.690049552 | 3.922403377 | -3.012600514 | 0.010266038 | 0.033886871 | -3.420812638 |
| VAMP3 | -1.015485488 | 9.910490564 | -3.006338771 | 0.010388965 | 0.034216897 | -3.432519833 |
| SMURF1 | 1.414306599 | 5.070186393 | 3.006242758 | 0.010390862 | 0.034216897 | -3.432699314 |
| TBC1D16 | 1.433642234 | 4.996592459 | 3.005483577 | 0.010405867 | 0.034236547 | -3.434118443 |
| SLC2A8 | -1.506354762 | 8.992782019 | -3.004672518 | 0.010421923 | 0.034274484 | -3.435634485 |
| ZNF518B | -1.30117274 | 7.352652395 | -3.003942366 | 0.010436397 | 0.034307193 | -3.436999238 |
| HK2 | 1.16028501 | 6.704592128 | 3.00170582 | 0.010480859 | 0.034416016 | -3.441179325 |
| DDX43 | -2.995700496 | 5.741245661 | -2.999741642 | 0.010520062 | 0.034514129 | -3.444849953 |
| MYOF | -1.426398144 | 6.240530886 | -2.997460476 | 0.010565775 | 0.03462731 | -3.449112486 |
| MRGPRX4 | 1.947894386 | 5.598718667 | 2.996832518 | 0.010578393 | 0.034651048 | -3.450285781 |
| SERPINB13 | 1.036418023 | 4.33361864 | 2.996348991 | 0.01058812 | 0.034663051 | -3.451189191 |
| ERO1B | 1.029939406 | 5.468735122 | 2.99529122 | 0.010609428 | 0.03471031 | -3.453165425 |
| ABHD8 | -1.067556079 | 10.34538983 | -2.994786781 | 0.010619605 | 0.034725217 | -3.454107829 |
| ISCA1 | -1.008804145 | 7.46747988 | -2.993689102 | 0.010641784 | 0.034778617 | -3.456158448 |
| SNRK | -1.082309493 | 7.991074331 | -2.99262667 | 0.010663294 | 0.03482638 | -3.458143107 |
| PTH2R | 3.039605051 | 3.291110662 | 2.99211143 | 0.010673742 | 0.034852988 | -3.459105552 |
| FAM20B | 1.073078488 | 9.808281071 | 2.990990356 | 0.010696509 | 0.034917304 | -3.461199574 |
| DHRS9 | -1.498457781 | 6.342442756 | -2.990711407 | 0.010702181 | 0.034923271 | -3.461720595 |
| NID1 | -3.231505733 | 6.131781287 | -2.989742321 | 0.010721911 | 0.034957536 | -3.463530594 |
| HASPIN | 1.355056333 | 4.356923791 | 2.988702119 | 0.010743128 | 0.035019178 | -3.465473313 |
| ELOB | -1.217738788 | 9.335329886 | -2.98761147 | 0.01076542 | 0.035065817 | -3.467510132 |
| CTHRC1 | 2.185157372 | 3.474377692 | 2.98754921 | 0.010766694 | 0.035065817 | -3.4676264 |
| OSBPL11 | -1.113612209 | 7.242146975 | -2.98692334 | 0.010779508 | 0.035076994 | -3.468795172 |
| STK38L | 1.518026207 | 3.278892692 | 2.98681629 | 0.010781702 | 0.035076994 | -3.468995077 |
| PCYOX1 | -1.073538179 | 6.998741634 | -2.985961233 | 0.010799236 | 0.035118959 | -3.470591766 |
| DIO1 | 1.03917736 | 3.038593414 | 2.985220048 | 0.010814459 | 0.035145832 | -3.471975756 |
| SHD | 1.812554446 | 9.798855704 | 2.982170212 | 0.010877322 | 0.035312257 | -3.477670024 |
| F2RL2 | 1.603023871 | 2.315620866 | 2.98185437 | 0.010883853 | 0.035325889 | -3.47825967 |
| LSG1 | 1.004268699 | 9.03534873 | 2.980750859 | 0.010906701 | 0.035381631 | -3.480319734 |
| INPP5D | 1.164476592 | 13.09316687 | 2.980686624 | 0.010908032 | 0.035381631 | -3.480439645 |
| MT-ND5 | 1.601360541 | 12.91451732 | 2.976882236 | 0.010987179 | 0.035585025 | -3.487540789 |
| CHRAC1 | -1.00720839 | 7.94894525 | -2.97615337 | 0.011002407 | 0.035619118 | -3.488901094 |
| OR13C5 | 1.47790053 | 4.188229967 | 2.975355671 | 0.011019098 | 0.035650299 | -3.490389802 |
| MINPP1 | 2.083890313 | 7.100347555 | 2.975085387 | 0.011024759 | 0.035656752 | -3.490894204 |
| NKD2 | 3.33830798 | 5.872802408 | 2.97488246 | 0.011029011 | 0.035659527 | -3.4912729 |
| HYAL1 | 1.038462801 | 2.863926121 | 2.974626954 | 0.011034367 | 0.035669233 | -3.491749712 |
| DCAF7 | 1.019860008 | 8.812826233 | 2.974070806 | 0.011046034 | 0.035691718 | -3.492787542 |
| PRXL2A | -2.194617906 | 6.134020522 | -2.971400728 | 0.011102221 | 0.03585033 | -3.497769735 |
| BLVRA | -1.959744556 | 12.55530781 | -2.969286881 | 0.011146904 | 0.035957971 | -3.501713502 |
| GPR19 | -2.057188811 | 3.914438118 | -2.969262409 | 0.011147422 | 0.035957971 | -3.501759156 |
| MMP7 | 2.515602329 | 2.807425786 | 2.968992407 | 0.011153143 | 0.035968766 | -3.50226286 |
| RTP3 | 1.935343471 | 2.701684469 | 2.968221874 | 0.011169484 | 0.0360138 | -3.503700288 |
| LRRC36 | -1.280163519 | 7.185994419 | -2.967250879 | 0.01119011 | 0.036051054 | -3.505511588 |
| ERICH1 | -1.011037324 | 9.023093618 | -2.967229928 | 0.011190556 | 0.036051054 | -3.505550669 |
| PRICKLE4 | 1.199207708 | 4.297643134 | 2.96390044 | 0.011261573 | 0.036256713 | -3.511760745 |
| CSN1S1 | 1.030012922 | 6.338198446 | 2.959290558 | 0.01136064 | 0.036484102 | -3.520357015 |
| COL6A2 | -1.57265578 | 6.377000247 | -2.955759044 | 0.011437118 | 0.036658298 | -3.526940844 |
| GP5 | -1.376257089 | 2.294535543 | -2.953338943 | 0.011489822 | 0.036780541 | -3.53145187 |
| ZNF33B | 1.455662888 | 9.112463891 | 2.951936915 | 0.011520466 | 0.036847493 | -3.534064934 |
| SP140 | -1.249803609 | 7.721508699 | -2.950644565 | 0.011548784 | 0.036899118 | -3.536473388 |
| GALNT11 | -2.071147826 | 6.204377007 | -2.950285363 | 0.011556667 | 0.03691652 | -3.537142774 |
| ZNF814 | 1.102804476 | 7.282353005 | 2.950142196 | 0.01155981 | 0.036918778 | -3.537409565 |
| BTBD17 | -2.013826513 | 2.929259008 | -2.948258128 | 0.011601257 | 0.037004345 | -3.540920326 |
| ZNF304 | 1.278698137 | 5.111756797 | 2.947953731 | 0.011607967 | 0.037008898 | -3.5414875 |
| MAMDC2 | 2.424214411 | 2.828973437 | 2.946904893 | 0.011631117 | 0.037060579 | -3.543441692 |
| GPR42 | -1.279233 | 4.722369142 | -2.946632644 | 0.011637133 | 0.037071953 | -3.543948927 |
| MSGN1 | 1.166232091 | 3.366307212 | 2.945011745 | 0.011673019 | 0.037162829 | -3.54696869 |
| TMEM74 | -1.37579994 | 4.128804376 | -2.942254852 | 0.011734306 | 0.037310732 | -3.552104156 |
| PLIN2 | 1.653601556 | 10.7181386 | 2.940641627 | 0.011770317 | 0.037394018 | -3.555108831 |
| NOM1 | 1.095537105 | 6.653288481 | 2.939824323 | 0.011788603 | 0.037436539 | -3.556630971 |
| KIAA1755 | 1.312405816 | 3.55166377 | 2.937667037 | 0.011837004 | 0.037566496 | -3.560648316 |
| GPR12 | 1.423400198 | 3.387087473 | 2.937013187 | 0.011851713 | 0.0376053 | -3.561865825 |
| HMSD | -1.989678125 | 4.454892981 | -2.935222583 | 0.011892087 | 0.037699307 | -3.565199791 |
| LMX1A | 1.277511967 | 3.800681057 | 2.935103398 | 0.011894779 | 0.037699307 | -3.565421692 |
| CAMK2B | 1.820011788 | 2.485741107 | 2.934967318 | 0.011897854 | 0.037699307 | -3.565675045 |
| DEPDC1B | 2.269272956 | 5.288043791 | 2.934927301 | 0.011898758 | 0.037699307 | -3.565749548 |
| THBD | -1.452906364 | 5.544045199 | -2.933537944 | 0.011930196 | 0.037759433 | -3.568336126 |
| NPHP4 | 1.52517354 | 7.207284464 | 2.931050398 | 0.01198669 | 0.037867044 | -3.572966656 |
| FAM221A | -1.219763488 | 4.724553511 | -2.929289512 | 0.012026841 | 0.037978048 | -3.576244089 |
| CAMK2D | -2.185582803 | 6.029572836 | -2.928685731 | 0.012040639 | 0.038005776 | -3.577367788 |
| EPCAM | 4.36704143 | 4.631641441 | 2.926431137 | 0.012092301 | 0.038105336 | -3.581563449 |
| UBE2O | 1.114535177 | 10.27350197 | 2.926020144 | 0.012101742 | 0.03812141 | -3.582328219 |
| CTTN | -2.836558881 | 8.722987173 | -2.925880571 | 0.01210495 | 0.03812141 | -3.582587929 |
| BIRC5 | 2.40217072 | 9.557385943 | 2.923259232 | 0.012165355 | 0.038271863 | -3.587465167 |
| ENC1 | -1.289856236 | 7.073405212 | -2.922843229 | 0.012174968 | 0.038272815 | -3.588239105 |
| KRTAP25-1 | 1.230325215 | 4.761814868 | 2.92278049 | 0.012176419 | 0.038272815 | -3.588355823 |
| OR4Q3 | 1.204890075 | 5.108976541 | 2.922748596 | 0.012177156 | 0.038272815 | -3.588415158 |
| UBQLNL | 2.023221164 | 5.666257952 | 2.921796661 | 0.012199187 | 0.038330544 | -3.590186062 |
| SLC12A6 | 1.276332914 | 9.089085287 | 2.919709603 | 0.012247627 | 0.03845603 | -3.594068289 |
| PCDHGB2 | 1.059057995 | 6.11102191 | 2.919638625 | 0.012249277 | 0.03845603 | -3.594200308 |
| TPSD1 | 3.960481659 | 5.547986063 | 2.912035222 | 0.01242739 | 0.038886285 | -3.608339296 |
| SGCB | -2.382060102 | 4.319714445 | -2.911400321 | 0.012442379 | 0.038902715 | -3.60951962 |
| CLDN22 | -1.131721932 | 2.335664924 | -2.910858934 | 0.012455174 | 0.038928134 | -3.610526058 |
| ICAM3 | -1.836217114 | 13.36588782 | -2.909409885 | 0.012489484 | 0.039008075 | -3.613219664 |
| ACY3 | 1.634137657 | 6.589382404 | 2.908791211 | 0.012504161 | 0.039045867 | -3.614369626 |
| MS4A4E | 2.745672295 | 4.742207334 | 2.907406353 | 0.012537078 | 0.039124462 | -3.616943568 |
| IPP | -1.052309337 | 3.871598203 | -2.907272517 | 0.012540263 | 0.039125638 | -3.617192308 |
| TTC28 | 1.029486842 | 6.814513827 | 2.906679631 | 0.012554385 | 0.039138166 | -3.618294184 |
| INMT | 1.356644567 | 3.88304787 | 2.906510159 | 0.012558425 | 0.039142705 | -3.618609139 |
| UGT2B7 | 3.96670245 | 3.609095952 | 2.90489604 | 0.012596963 | 0.039230543 | -3.621608725 |
| NYNRIN | 1.025284444 | 6.480915063 | 2.903882944 | 0.012621212 | 0.039289907 | -3.623491243 |
| PTP4A3 | -1.201384953 | 7.847988371 | -2.899410879 | 0.012728804 | 0.039535488 | -3.631799657 |
| NFKBIL1 | -1.005117914 | 8.430870227 | -2.898536689 | 0.012749942 | 0.039584912 | -3.633423482 |
| PLD2 | -2.740285183 | 8.129085671 | -2.897639444 | 0.012771673 | 0.039636138 | -3.635090032 |
| CCL1 | 1.681282449 | 5.603290199 | 2.896838464 | 0.012791104 | 0.039688311 | -3.636577697 |
| CD83 | -1.58138026 | 11.10342264 | -2.895962322 | 0.012812392 | 0.039724907 | -3.638204869 |
| SLC6A9 | 2.419717153 | 6.382848726 | 2.895072622 | 0.012834045 | 0.039764543 | -3.639857124 |
| RRBP1 | 1.49998064 | 8.919861245 | 2.894338225 | 0.012851946 | 0.03978746 | -3.641220893 |
| RAB27A | -1.012076515 | 9.22984751 | -2.891450759 | 0.012922566 | 0.039952608 | -3.64658224 |
| LRSAM1 | 1.088995424 | 9.95290288 | 2.891402503 | 0.01292375 | 0.039952608 | -3.646671831 |
| KCNK6 | -2.172536252 | 5.92381373 | -2.891103708 | 0.01293108 | 0.039967115 | -3.647226563 |
| TNFRSF9 | -1.092496093 | 5.008517344 | -2.890650696 | 0.012942202 | 0.039977024 | -3.648067586 |
| LENG9 | 1.395997763 | 7.555555761 | 2.890400582 | 0.012948346 | 0.039980073 | -3.648531914 |
| FAM153C | -1.97995737 | 5.728125694 | -2.890395691 | 0.012948467 | 0.039980073 | -3.648540995 |
| PTAR1 | 1.117702026 | 7.857806664 | 2.889674031 | 0.012966212 | 0.040019266 | -3.64988069 |
| DSG4 | 1.07516727 | 3.365503246 | 2.889557303 | 0.012969084 | 0.040019266 | -3.650097379 |
| MT-ND3 | 2.371163717 | 12.05112324 | 2.886206741 | 0.013051806 | 0.040212339 | -3.656316481 |
| ZBTB5 | 1.101461731 | 9.138648702 | 2.886116712 | 0.013054036 | 0.040212339 | -3.656483569 |
| RAB44 | 1.496863297 | 8.723869985 | 2.885099686 | 0.013079253 | 0.040252643 | -3.658371017 |
| PTPN7 | 1.835930653 | 12.40013779 | 2.884517874 | 0.013093701 | 0.040288923 | -3.659450714 |
| CFC1 | 1.570169204 | 3.998278164 | 2.883354971 | 0.013122625 | 0.040361529 | -3.661608642 |
| ICA1 | -1.812156094 | 8.347376778 | -2.882397502 | 0.013146487 | 0.040418512 | -3.663385229 |
| C8A | 1.087732938 | 4.488619101 | 2.879651812 | 0.013215154 | 0.040554755 | -3.668479218 |
| ANKRD18B | 1.076877362 | 3.785301237 | 2.87955551 | 0.013217569 | 0.040554755 | -3.668657867 |
| ETV5 | 1.848538772 | 3.53599718 | 2.87873155 | 0.013238248 | 0.040593542 | -3.670186335 |
| ODR4 | 1.010682992 | 8.2314638 | 2.873780583 | 0.013363179 | 0.040910392 | -3.679368673 |
| ANKH | -1.601533363 | 4.732624136 | -2.872808711 | 0.01338784 | 0.040977608 | -3.681170791 |
| ZNF7 | 1.562667008 | 6.06562613 | 2.868776134 | 0.013490645 | 0.041241883 | -3.688646976 |
| ACRV1 | 1.140556658 | 2.484035866 | 2.868592647 | 0.013495341 | 0.041241883 | -3.688987101 |
| RAB33B | -1.456619534 | 2.729161378 | -2.868176109 | 0.013506008 | 0.041255954 | -3.689759209 |
| ERAS | 1.28353423 | 3.172967592 | 2.86794442 | 0.013511945 | 0.04126577 | -3.690188667 |
| VMO1 | -2.447023528 | 5.946333055 | -2.86463335 | 0.013597072 | 0.04142532 | -3.696325265 |
| TSPYL5 | -1.288653184 | 5.454691092 | -2.864206133 | 0.013608095 | 0.041440526 | -3.697116946 |
| INHBE | 1.021029373 | 7.39414887 | 2.863182498 | 0.01363454 | 0.04148131 | -3.69901376 |
| TANK | -1.026758086 | 9.249227508 | -2.863061894 | 0.01363766 | 0.04148247 | -3.699237231 |
| C10orf105 | -1.870545179 | 5.184522005 | -2.862005249 | 0.013665018 | 0.041549003 | -3.701195049 |
| JCHAIN | -2.361890315 | 11.54033701 | -2.861625622 | 0.01367486 | 0.041570587 | -3.701898409 |
| OR6K2 | 1.069755111 | 8.392294885 | 2.861212182 | 0.013685587 | 0.041586508 | -3.702664396 |
| TEKT4 | -2.057648889 | 5.43447075 | -2.858122498 | 0.013766014 | 0.041797371 | -3.70838798 |
| FIGLA | -1.393936786 | 2.474867027 | -2.857252073 | 0.013788756 | 0.041841268 | -3.710000197 |
| DDTL | -1.218582763 | 5.868114633 | -2.854470422 | 0.013861684 | 0.041998683 | -3.715151742 |
| IER3IP1 | -1.082673017 | 9.103610174 | -2.85210301 | 0.013924051 | 0.042125326 | -3.719535306 |
| HAPLN4 | 1.095706901 | 4.89188382 | 2.850998293 | 0.013953248 | 0.042196739 | -3.721580573 |
| THSD7A | 1.519550813 | 5.721797498 | 2.850118509 | 0.013976544 | 0.042241894 | -3.72320928 |
| WTIP | 1.223215987 | 3.367226454 | 2.848268031 | 0.014025668 | 0.042356566 | -3.726634651 |
| OR6B2 | 1.214342406 | 3.976460399 | 2.848149687 | 0.014028815 | 0.042357628 | -3.726853699 |
| CNRIP1 | 2.711513451 | 3.170777338 | 2.847785323 | 0.01403851 | 0.042370013 | -3.727528103 |
| CCNL2 | 1.177062021 | 8.476139852 | 2.847288005 | 0.014051753 | 0.042401536 | -3.728448562 |
| HIST1H2BA | -1.077624847 | 2.791954181 | -2.846959498 | 0.014060508 | 0.04241106 | -3.729056559 |
| SLC22A7 | 1.445397751 | 6.964347317 | 2.845852507 | 0.014090048 | 0.042474796 | -3.731105262 |
| SUCNR1 | 3.099669256 | 4.345166485 | 2.844801817 | 0.014118143 | 0.04254256 | -3.733049613 |
| SH2D1A | -2.174282953 | 7.588200974 | -2.844208407 | 0.014134035 | 0.042573513 | -3.734147679 |
| MRPL36 | -1.020114655 | 10.74842897 | -2.84317618 | 0.014161721 | 0.042614058 | -3.736057631 |
| ZNF700 | 1.193759046 | 5.026919123 | 2.835058647 | 0.014381319 | 0.043146805 | -3.751072556 |
| NINJ2 | -1.435199678 | 8.32918642 | -2.832238199 | 0.014458403 | 0.043309462 | -3.756287368 |
| KLHL42 | 1.372272706 | 5.003299916 | 2.829576244 | 0.014531528 | 0.04349411 | -3.761208113 |
| CSF1R | -2.169928803 | 13.2628829 | -2.827613127 | 0.01458569 | 0.043638979 | -3.764836384 |
| CYTH3 | 1.309362563 | 9.165016368 | 2.823813066 | 0.014691099 | 0.043928329 | -3.771858182 |
| CXCL8 | -2.292738524 | 7.170016491 | -2.82288337 | 0.014717002 | 0.043997099 | -3.773575771 |
| DEFB1 | 1.609796741 | 7.015189023 | 2.822285299 | 0.014733689 | 0.044038296 | -3.774680628 |
| PRKD3 | -1.06047298 | 7.638795677 | -2.820846734 | 0.014773903 | 0.044105116 | -3.777337976 |
| ARMCX2 | -1.766392316 | 4.892458958 | -2.819878145 | 0.014801041 | 0.044169897 | -3.779127006 |
| CTSW | -2.106040176 | 10.25339826 | -2.819599307 | 0.014808862 | 0.044181851 | -3.779642008 |
| CCNA2 | 2.319662292 | 6.461572898 | 2.818723762 | 0.014833448 | 0.044240466 | -3.781259031 |
| OIP5 | 1.157169968 | 7.612665975 | 2.817714359 | 0.014861842 | 0.044298991 | -3.783123137 |
| SLC37A3 | -2.587376382 | 7.434791154 | -2.817021778 | 0.014881355 | 0.044339708 | -3.784402068 |
| MYO1F | -1.057261021 | 13.03786474 | -2.814933332 | 0.014940349 | 0.044454288 | -3.788258212 |
| MS4A5 | 1.208416016 | 5.119058275 | 2.813247364 | 0.014988143 | 0.044561489 | -3.791370751 |
| PIGZ | 1.027085305 | 8.044944376 | 2.812026024 | 0.015022859 | 0.044638425 | -3.793625262 |
| ATP8A2 | -2.020944167 | 3.928261975 | -2.80868132 | 0.015118337 | 0.044881518 | -3.799798248 |
| GJA9-MYCBP | -1.088976225 | 10.22202153 | -2.80864134 | 0.015119482 | 0.044881518 | -3.799872023 |
| AGTPBP1 | -1.049703176 | 8.02978979 | -2.808496673 | 0.015123625 | 0.044885023 | -3.800138983 |
| PLXNA3 | 1.014447572 | 8.85866967 | 2.807064894 | 0.015164693 | 0.044998094 | -3.802780935 |
| NUFIP2 | 1.165889045 | 9.424120765 | 2.806416326 | 0.015183333 | 0.045044581 | -3.803977588 |
| MARCKS | -1.936421169 | 9.1183661 | -2.806165697 | 0.015190542 | 0.045048326 | -3.804440001 |
| LDHAL6A | 1.54862821 | 12.88274538 | 2.80595864 | 0.0151965 | 0.045057176 | -3.804822015 |
| SLC24A4 | -2.126997831 | 8.705301275 | -2.803394655 | 0.015270471 | 0.045241085 | -3.809551968 |
| MTFR2 | 1.657417189 | 6.532977301 | 2.802466626 | 0.015297332 | 0.045311804 | -3.811263727 |
| RGS9BP | 1.938059112 | 2.631467277 | 2.802265492 | 0.01530316 | 0.045320206 | -3.811634702 |
| AMMECR1 | 1.259084311 | 5.060014043 | 2.801792955 | 0.01531686 | 0.045325497 | -3.812506237 |
| FAM72A | 1.704880579 | 6.323113538 | 2.801635922 | 0.015321416 | 0.045325497 | -3.812795858 |
| OR4S1 | 1.1589278 | 2.343661123 | 2.800955072 | 0.015341182 | 0.045361877 | -3.814051527 |
| LAMB3 | -1.750435364 | 4.401636911 | -2.799099798 | 0.015395174 | 0.045486013 | -3.817472797 |
| SNAP29 | -1.229965381 | 6.317588208 | -2.796398184 | 0.015474129 | 0.045692561 | -3.822453865 |
| PRICKLE1 | 1.487769232 | 4.553713504 | 2.795963307 | 0.015486876 | 0.045721288 | -3.823255561 |
| PDGFA | -1.57747818 | 6.558493505 | -2.795360529 | 0.015504561 | 0.045746757 | -3.824366738 |
| WDR86 | -2.525040977 | 7.055688146 | -2.794402788 | 0.015532701 | 0.045811943 | -3.826132149 |
| PNPLA4 | -1.287685135 | 4.430562356 | -2.793827222 | 0.015549636 | 0.045852966 | -3.827193029 |
| MED12L | 2.165510463 | 2.954261971 | 2.79211394 | 0.015600156 | 0.04591446 | -3.830350644 |
| TCP11L2 | -1.206928992 | 8.113798118 | -2.791846661 | 0.015608051 | 0.045926896 | -3.830843204 |
| HTR3C | 1.266645628 | 2.706832772 | 2.791587191 | 0.01561572 | 0.045940539 | -3.831321363 |
| GABRR1 | 1.006829612 | 3.164962167 | 2.791187582 | 0.015627538 | 0.045957459 | -3.832057756 |
| FAM200A | -1.100055918 | 4.260971658 | -2.790545121 | 0.015646557 | 0.045995534 | -3.833241621 |
| MGAM | -1.535971542 | 3.986570235 | -2.783195404 | 0.015865756 | 0.046540574 | -3.846780506 |
| CHRM4 | -1.417373231 | 5.404577545 | -2.781382787 | 0.015920278 | 0.046652497 | -3.850118252 |
| GSTA4 | -1.473902062 | 5.877433002 | -2.781192892 | 0.015926001 | 0.046652497 | -3.850467895 |
| HIST2H2BF | 1.901448614 | 6.378130456 | 2.780178553 | 0.015956603 | 0.046716616 | -3.852335445 |
| SULT4A1 | 1.097379166 | 5.714107151 | 2.779444682 | 0.015978779 | 0.046762245 | -3.853686511 |
| EIF5 | 1.247629452 | 9.720544538 | 2.777088504 | 0.016050185 | 0.0469 | -3.858023708 |
| CA1 | 4.025901474 | 10.76220078 | 2.776966638 | 0.016053887 | 0.0469 | -3.858248011 |
| MTMR7 | 1.487278429 | 2.746117106 | 2.776884385 | 0.016056386 | 0.0469 | -3.858399405 |
| REP15 | 1.4698238 | 3.279414432 | 2.775960241 | 0.016084489 | 0.046945885 | -3.860100283 |
| TEX44 | 1.060849524 | 4.583004105 | 2.774815955 | 0.016119353 | 0.047038583 | -3.862206147 |
| DLGAP5 | 2.516161433 | 6.096178256 | 2.774269859 | 0.016136018 | 0.047057319 | -3.863211071 |
| FBLN1 | 1.195837605 | 6.840848056 | 2.774063463 | 0.016142321 | 0.047057319 | -3.863590868 |
| LIMS4 | 1.749870018 | 3.929684401 | 2.77382738 | 0.016149533 | 0.047057319 | -3.864025287 |
| F3 | 1.875078425 | 2.608633883 | 2.772384044 | 0.016193697 | 0.047158354 | -3.866680987 |
| RNF152 | 1.796421516 | 3.272862495 | 2.770115344 | 0.016263355 | 0.047312997 | -3.870854679 |
| RAB42 | 1.018744538 | 3.97061167 | 2.768792351 | 0.016304112 | 0.047413359 | -3.873288195 |
| ME3 | 1.498503817 | 7.67491899 | 2.768232604 | 0.016321386 | 0.047454485 | -3.874317713 |
| CALHM6 | -1.986095976 | 11.06397154 | -2.766215167 | 0.016383795 | 0.047626799 | -3.878027883 |
| TPM1 | -1.323169824 | 5.249329505 | -2.765677253 | 0.016400475 | 0.047656999 | -3.879017026 |
| RACGAP1 | 1.108850851 | 5.414185832 | 2.764910093 | 0.016424292 | 0.047710372 | -3.88042764 |
| TNFAIP6 | -1.218978934 | 2.309787032 | -2.764223492 | 0.016445637 | 0.047760753 | -3.881690046 |
| C21orf62 | 1.654731336 | 3.306672071 | 2.761796726 | 0.016521299 | 0.047962101 | -3.886151376 |
| OR10A7 | -1.119691742 | 2.378940151 | -2.758701978 | 0.016618284 | 0.048174727 | -3.891839354 |
| HIST1H2AD | 1.171248319 | 14.18444608 | 2.757503699 | 0.016655986 | 0.048251405 | -3.894041313 |
| CSF1 | 1.031056233 | 6.350813043 | 2.75415378 | 0.016761834 | 0.048515654 | -3.900195909 |
| FBXO7 | 1.052991675 | 12.91559186 | 2.753065888 | 0.01679635 | 0.048578499 | -3.902194237 |
| SOX6 | 2.560490595 | 3.233004554 | 2.749558123 | 0.016908119 | 0.048843557 | -3.908636276 |
| LDLRAD2 | 3.701606521 | 5.536034453 | 2.748639052 | 0.016937524 | 0.048889393 | -3.910323827 |
| STX19 | 1.240511837 | 4.149032343 | 2.747932019 | 0.016960179 | 0.048926823 | -3.911621952 |
| RGPD2 | -2.020329143 | 4.275580515 | -2.747747585 | 0.016966094 | 0.048934569 | -3.911960562 |
| CCNDBP1 | -1.063115928 | 4.925034862 | -2.740646157 | 0.017195378 | 0.049453972 | -3.924994153 |
| ZNF575 | -1.258390301 | 7.393025279 | -2.739867559 | 0.017220701 | 0.049480532 | -3.926422646 |
| PAQR4 | 1.257630787 | 8.319027284 | 2.736740447 | 0.017322776 | 0.049707838 | -3.932158949 |
| PDZK1IP1 | -1.918384628 | 8.74827605 | -2.73452669 | 0.017395396 | 0.049831282 | -3.936218832 |
| EVI5 | -1.435601443 | 5.933535737 | -2.734203999 | 0.017406007 | 0.049852251 | -3.936810558 |
| PLPP3 | 1.317170146 | 3.612098207 | 2.733851202 | 0.017417614 | 0.049876068 | -3.937457469 |
| HIGD1B | 1.169298664 | 3.946392308 | 2.73280913 | 0.017451945 | 0.049964931 | -3.939368161 |
