# Supplementary material for: Regulatory function and mechanism research for m6A modification WTAP via SUCLG2-AS1- miR-17-5p-JAK1 axis in AML
Source: BMC Cancer. 2024 Jan 17;24:98. doi: 10.1186/s12885-023-11687-4 (PMC10795285; doi:10.1186/s12885-023-11687-4)
Supplement: Supplementary file 4 — Additional file 4: Supplementary Table S4. GO biological process terms and KEGG enriched pathways for ceRNA network-related DEmRNAs. [file 12885_2023_11687_MOESM4_ESM.docx]

**Supplementary Table S4 GO biological process terms and KEGG enriched pathways for ceRNA network-related DEmRNAs**

| **Term** | **Genes** | **P-value** |
| --- | --- | --- |
| GO biological process |  |  |
| regulation of Ras protein signal transduction | ARHGEF3/CSF1/ABL2/CYTH3/STARD13 | 2.76E-05 |
| regulation of hemopoiesis | GPR137B/CSF1/MAPK14/HMGB3/FNIP1/RUNX1 | 0.040732843 |
| regulation of myeloid cell differentiation | GPR137B/CSF1/MAPK14/HMGB3/RUNX1 | 0.027432388 |
| regulation of small GTPase mediated signal transduction | ARHGEF3/CSF1/ABL2/CYTH3/STARD13 | 0.000303653 |
| positive regulation of cell adhesion | JAK1/CSF1/CYTH3/RUNX1/FUT4 | 0.001087235 |
| myeloid leukocyte differentiation | GPR137B/CSF1/MAPK14/RUNX1 | 0.000596019 |
| response to mechanical stimulus | KCNJ2/TXNIP/MAPK14/STRBP | 0.000585434 |
| body morphogenesis | SGPL1/GREM2/ANKRD11 | 0.00010164 |
| positive regulation of myeloid cell differentiation | CSF1/MAPK14/RUNX1 | 0.000860647 |
| osteoclast differentiation | GPR137B/CSF1/MAPK14 | 0.000788477 |
| KEGG pathway |  |  |
| Osteoclast differentiation | AKT3/JAK1/CSF1/MAPK14 | 0.000191 |
| PD-L1 expression and PD-1 checkpoint pathway in cancer | AKT3/JAK1/MAPK14 | 0.001092 |
| Th17 cell differentiation | JAK1/MAPK14/RUNX1 | 0.001859 |
| TNF signaling pathway | AKT3/CSF1/MAPK14 | 0.002119 |
| Toxoplasmosis | AKT3/JAK1/MAPK14 | 0.002119 |
| Sphingolipid signaling pathway | AKT3/SGPL1/MAPK14 | 0.00252 |
| Signaling pathways regulating pluripotency of stem cells | AKT3/JAK1/MAPK14 | 0.004238 |
| NOD-like receptor signaling pathway | TXNIP/JAK1/MAPK14 | 0.008159 |
| Acute myeloid leukemia | AKT3/RUNX1 | 0.010516 |
| Rap1 signaling pathway | AKT3/CSF1/MAPK14 | 0.012235 |
