# Supplementary figures and images for: Regulatory function and mechanism research for m6A modification WTAP via SUCLG2-AS1- miR-17-5p-JAK1 axis in AML
Source: BMC Cancer. 2024 Jan 17;24:98. doi: 10.1186/s12885-023-11687-4 (PMC10795285; doi:10.1186/s12885-023-11687-4)

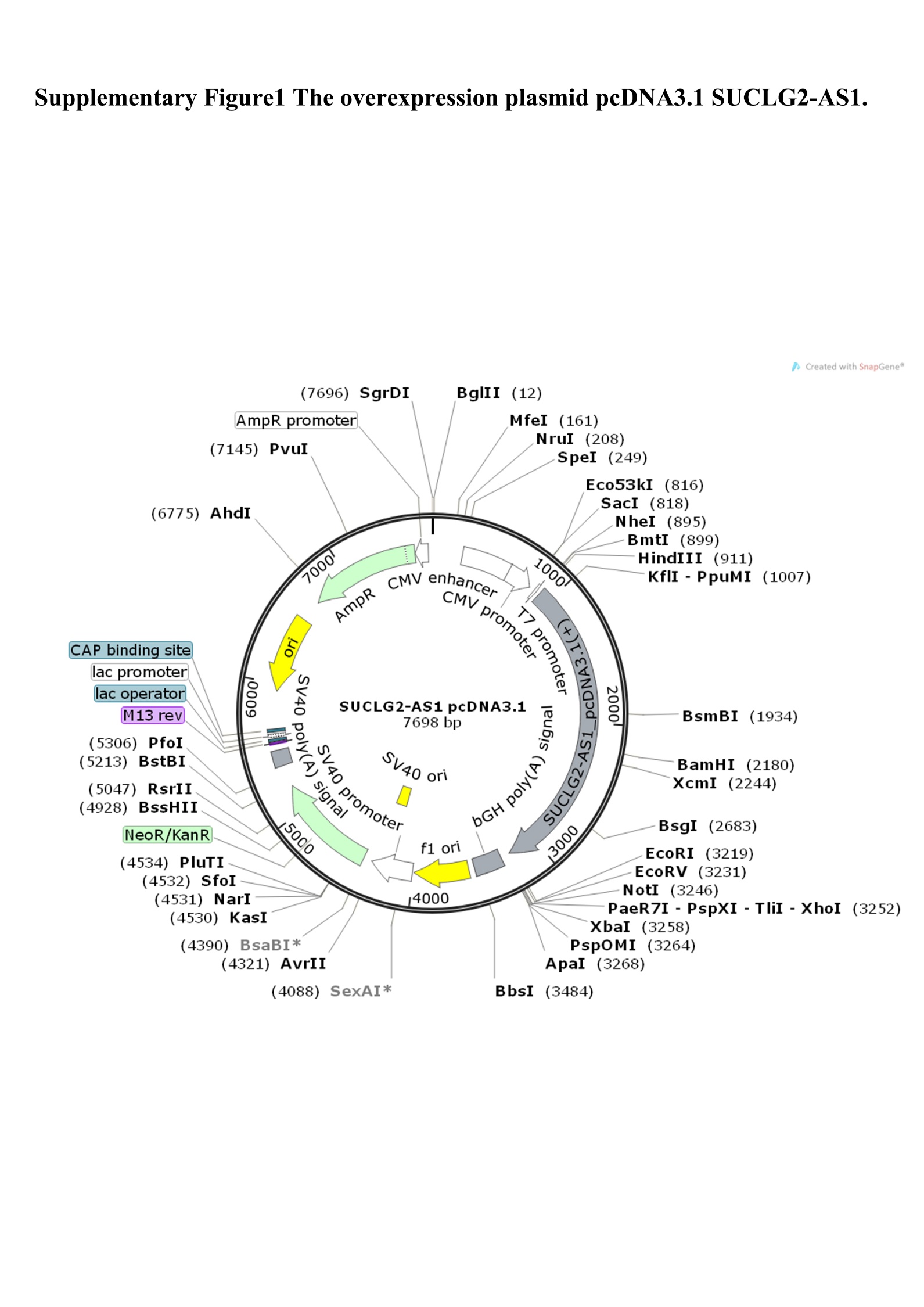


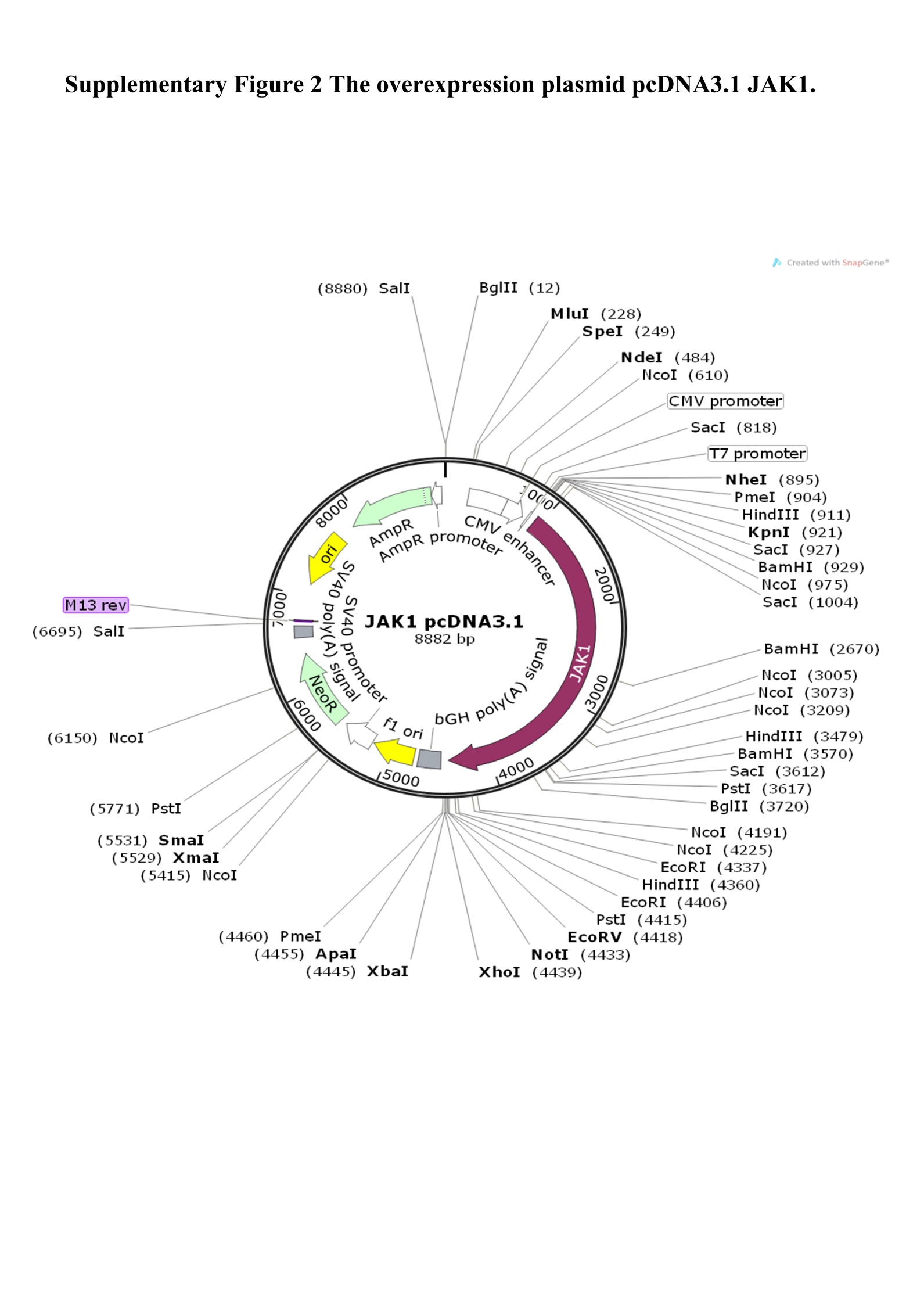


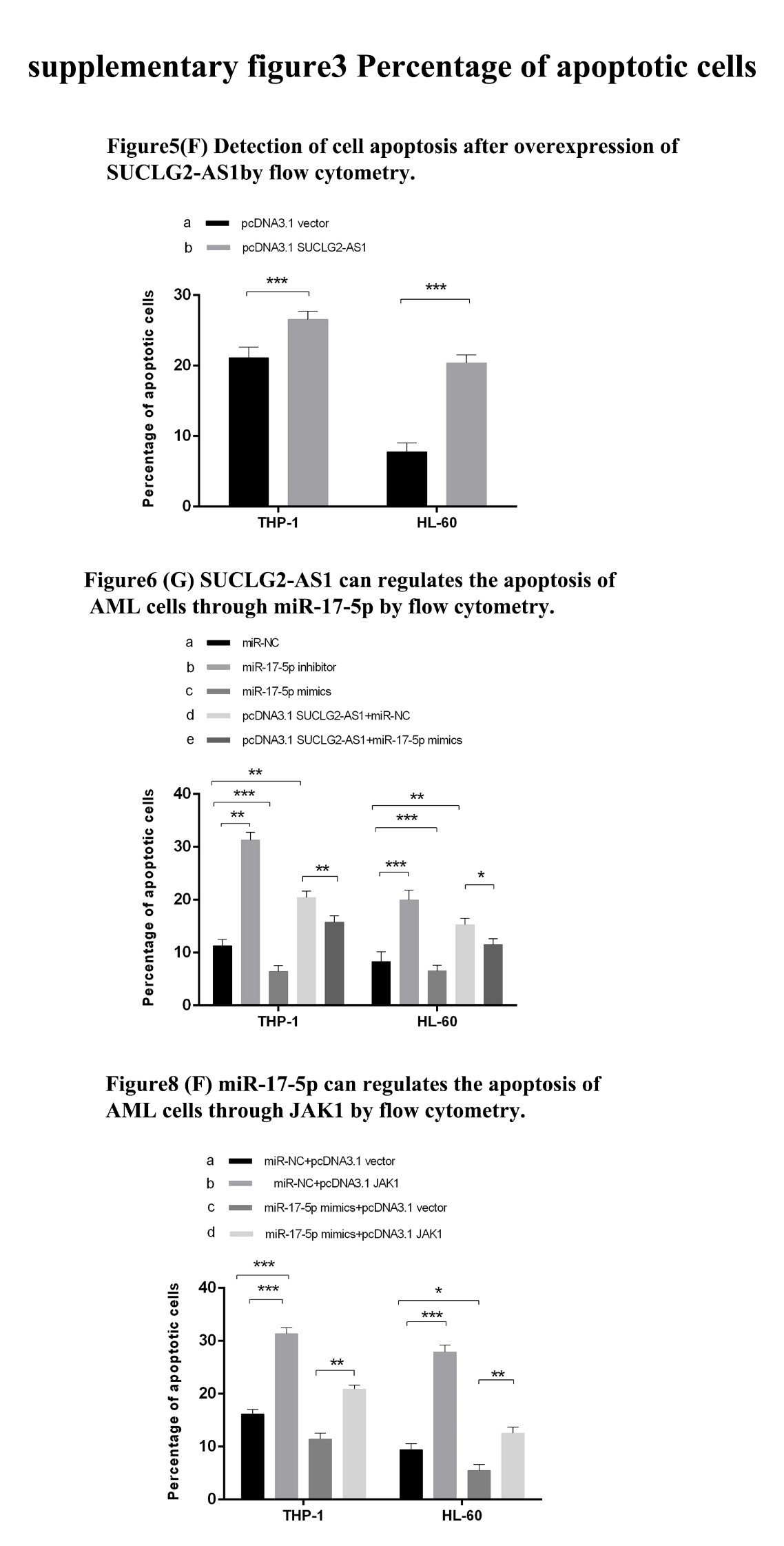

Supplement: Supplementary file 5 — Additional file 5: Supplementary Figure 1. The overexpression plasmid pcDNA3.1 SUCLG2-AS1. Supplementary Figure 2. The overexpression plasmid pcDNA3.1 JAKI. Supplementary Figure 3. Percentage of apoptotic cells. [file 12885_2023_11687_MOESM5_ESM.docx]
