# Supplementary material for: Regulatory function and mechanism research for m6A modification WTAP via SUCLG2-AS1- miR-17-5p-JAK1 axis in AML
Source: BMC Cancer. 2024 Jan 17;24:98. doi: 10.1186/s12885-023-11687-4 (PMC10795285; doi:10.1186/s12885-023-11687-4)

**Fig5：**

**β-actin-THP-1**


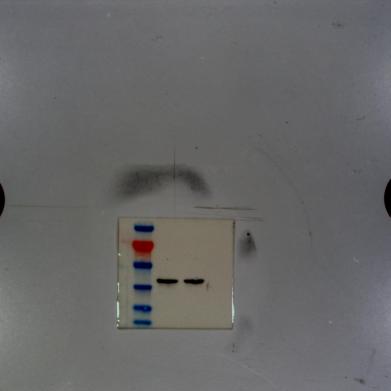

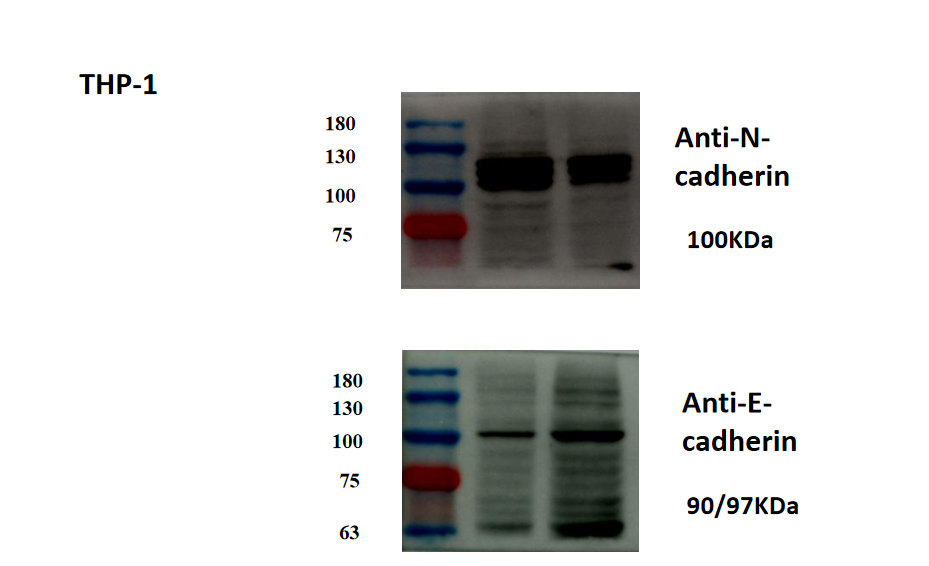


**β-actin-HL-60**


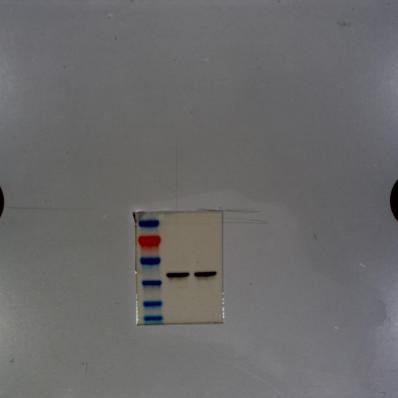

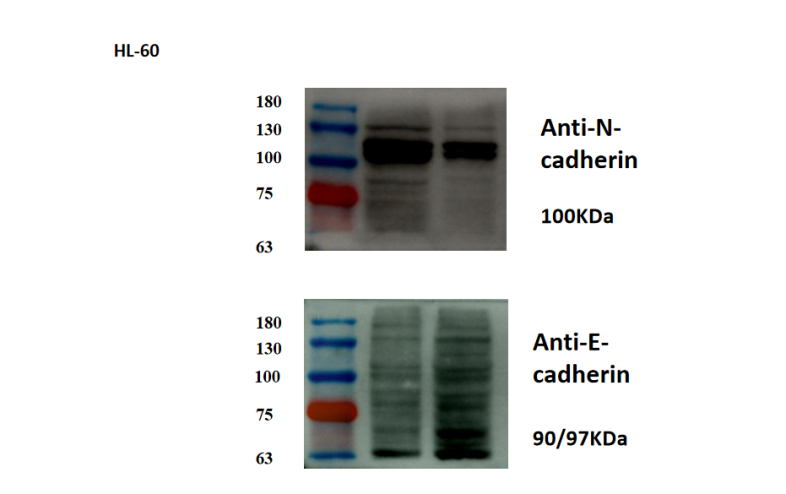


**Fig7：**

**β-actin JKA1**

**
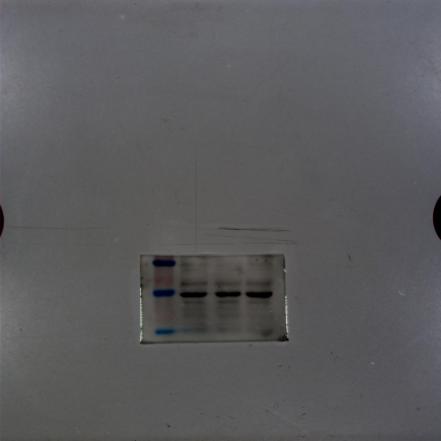

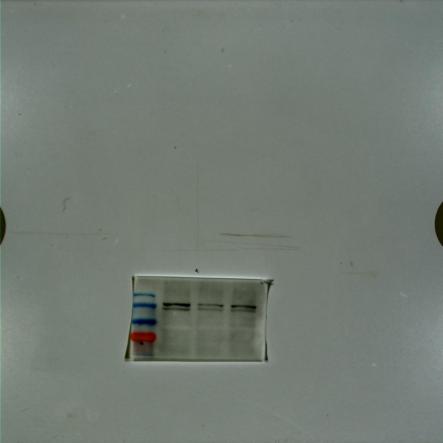
**

**THP-1** **β-actin JAK1**


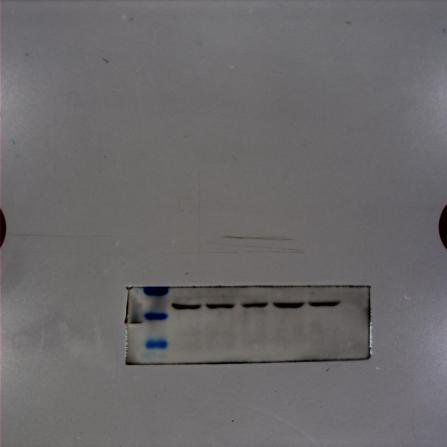

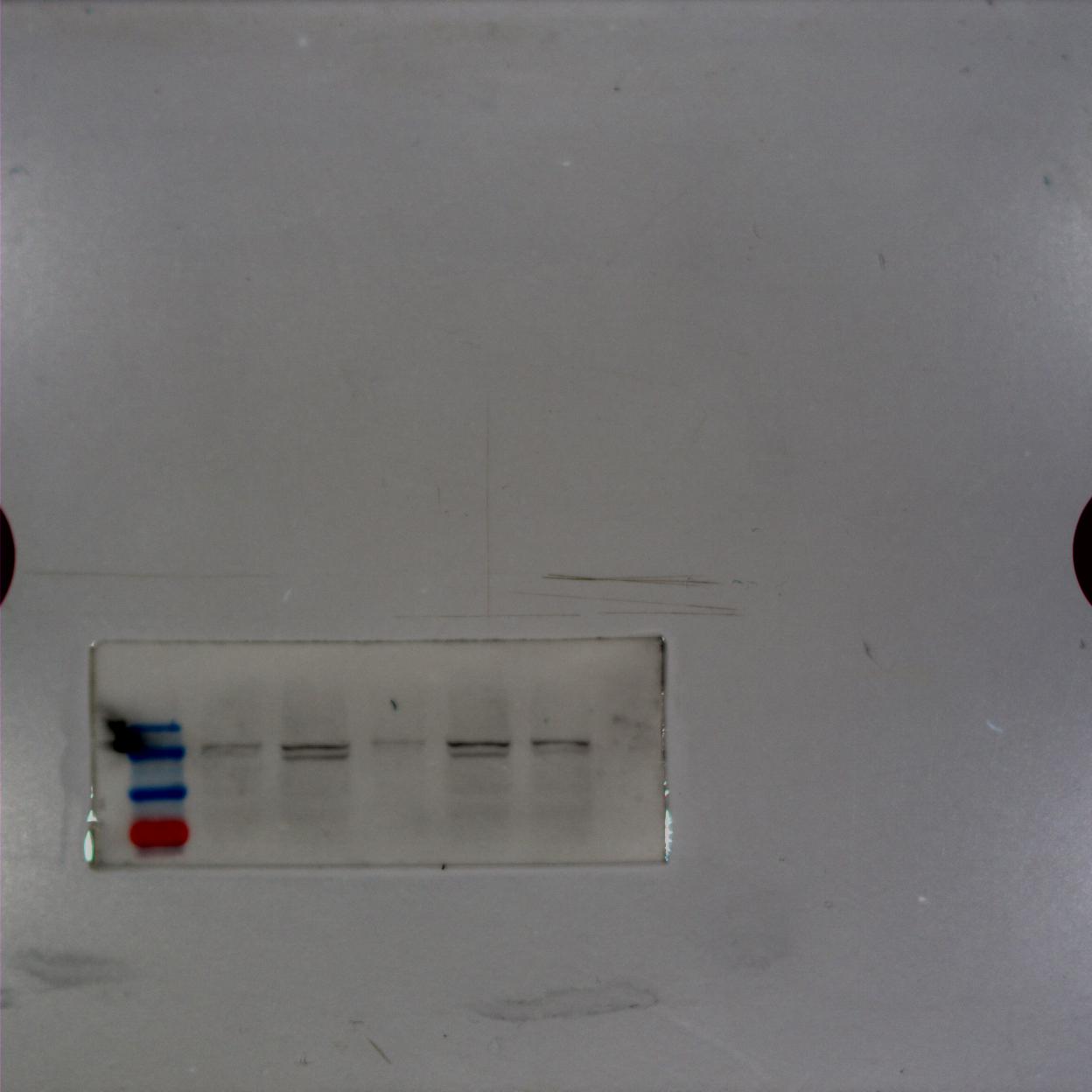


HL-60 **β-actin JAK1**


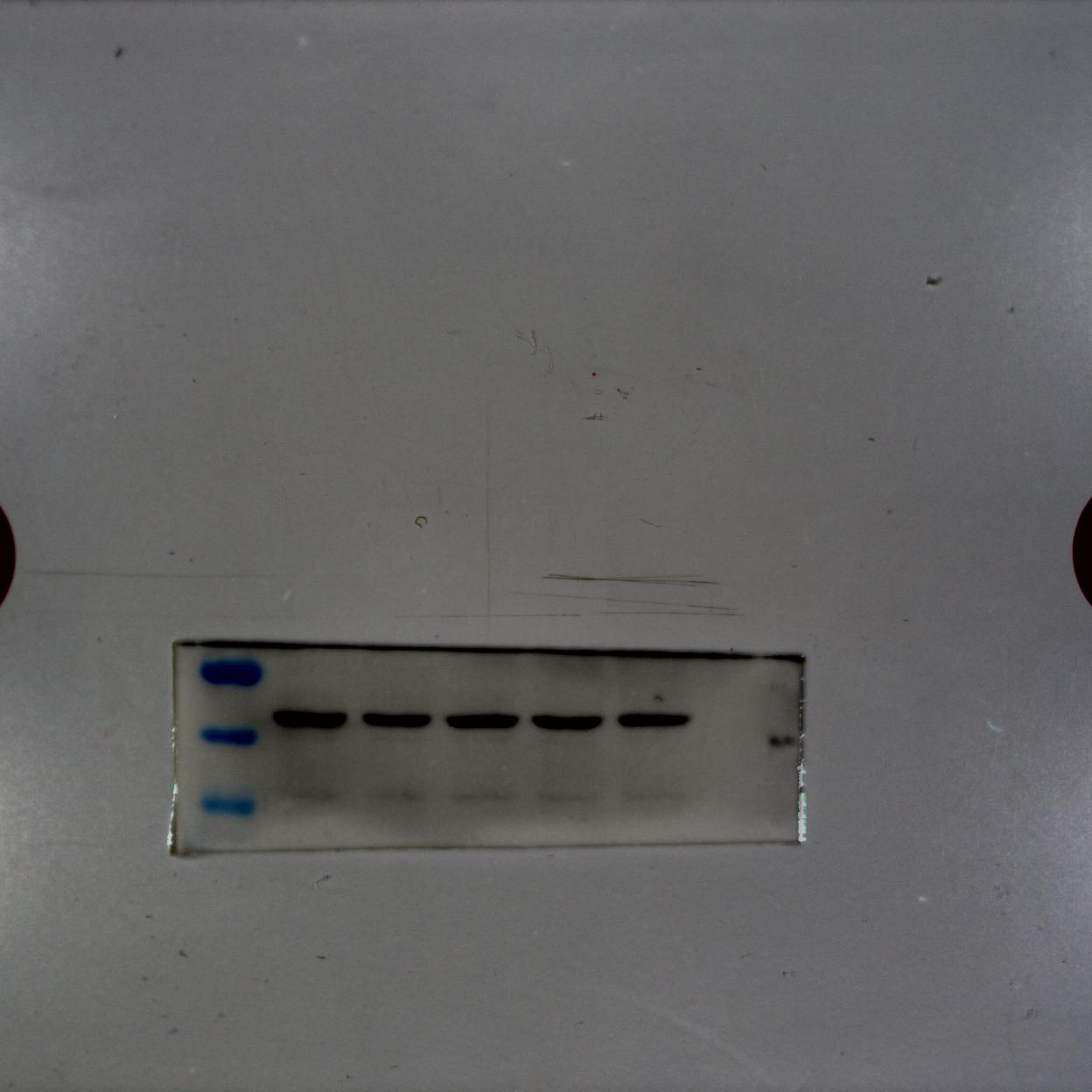

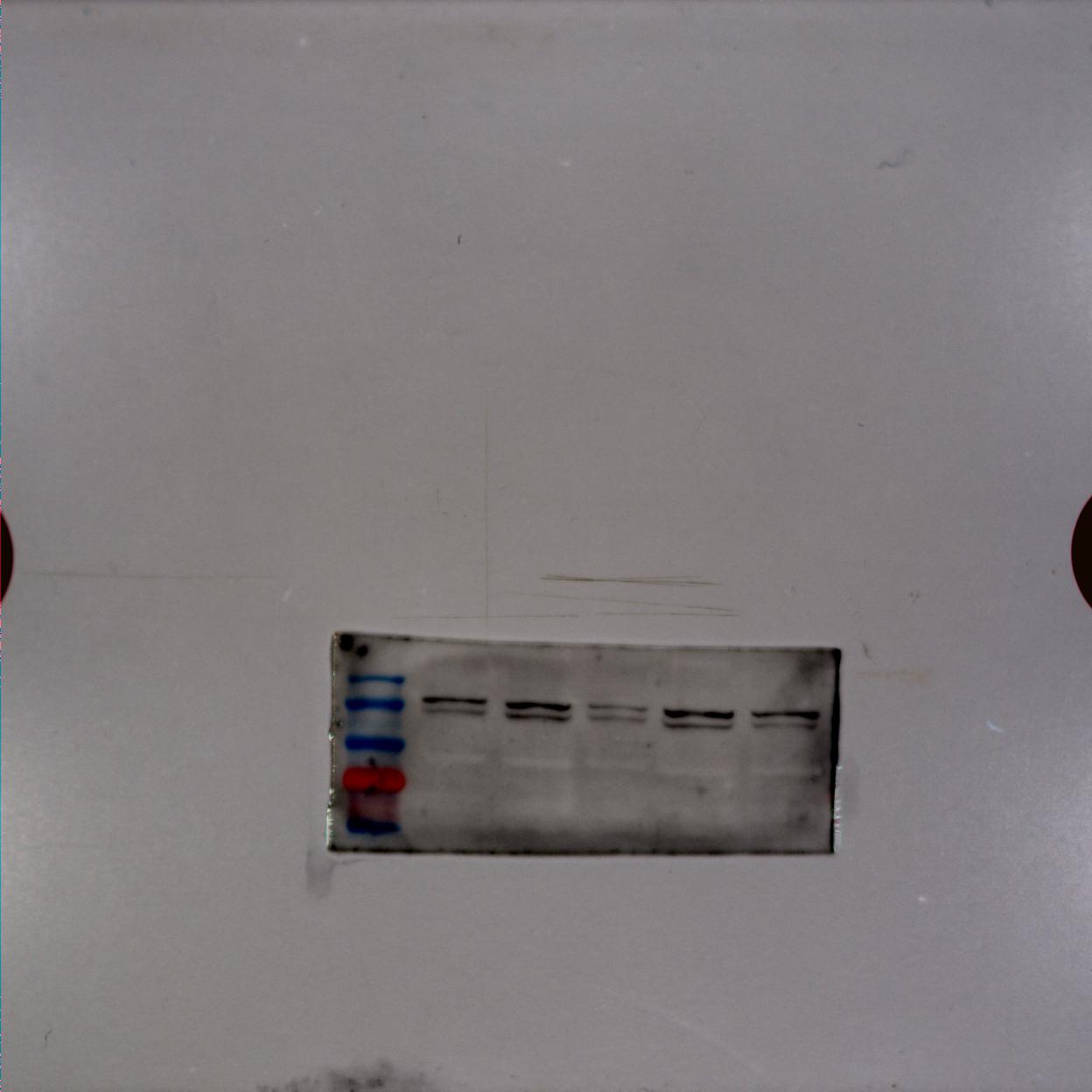


**Fig8：**

**THP-1** **β-actin JAK1**

**
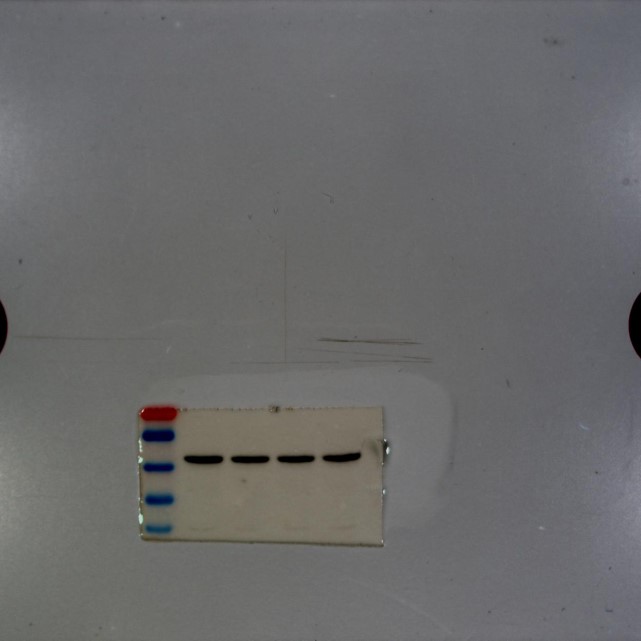

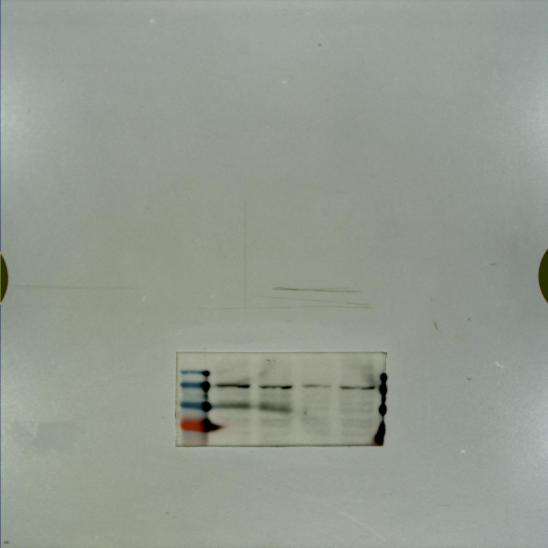
**

**HL-60** **β-actin JAK1**

**
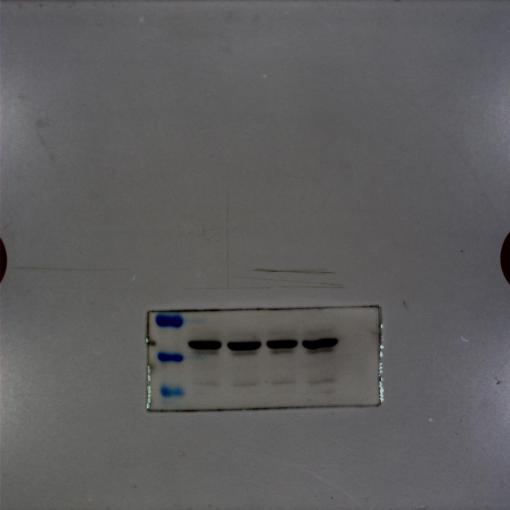

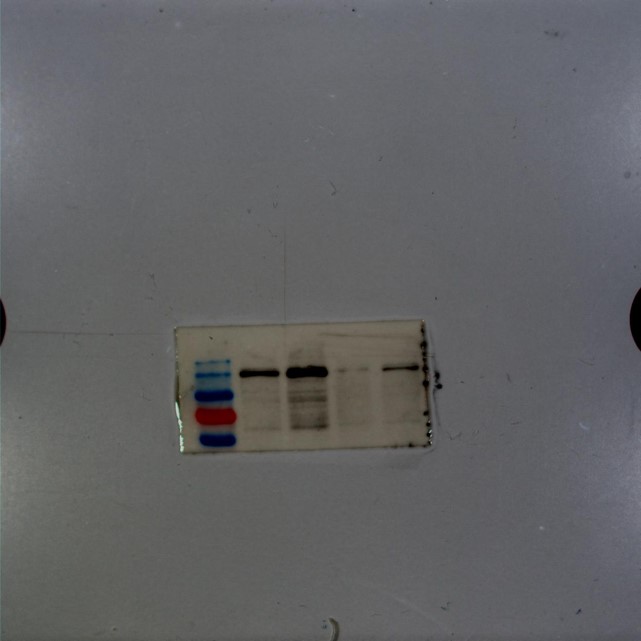
**

**Fig9：**

**β-actin WTAP**


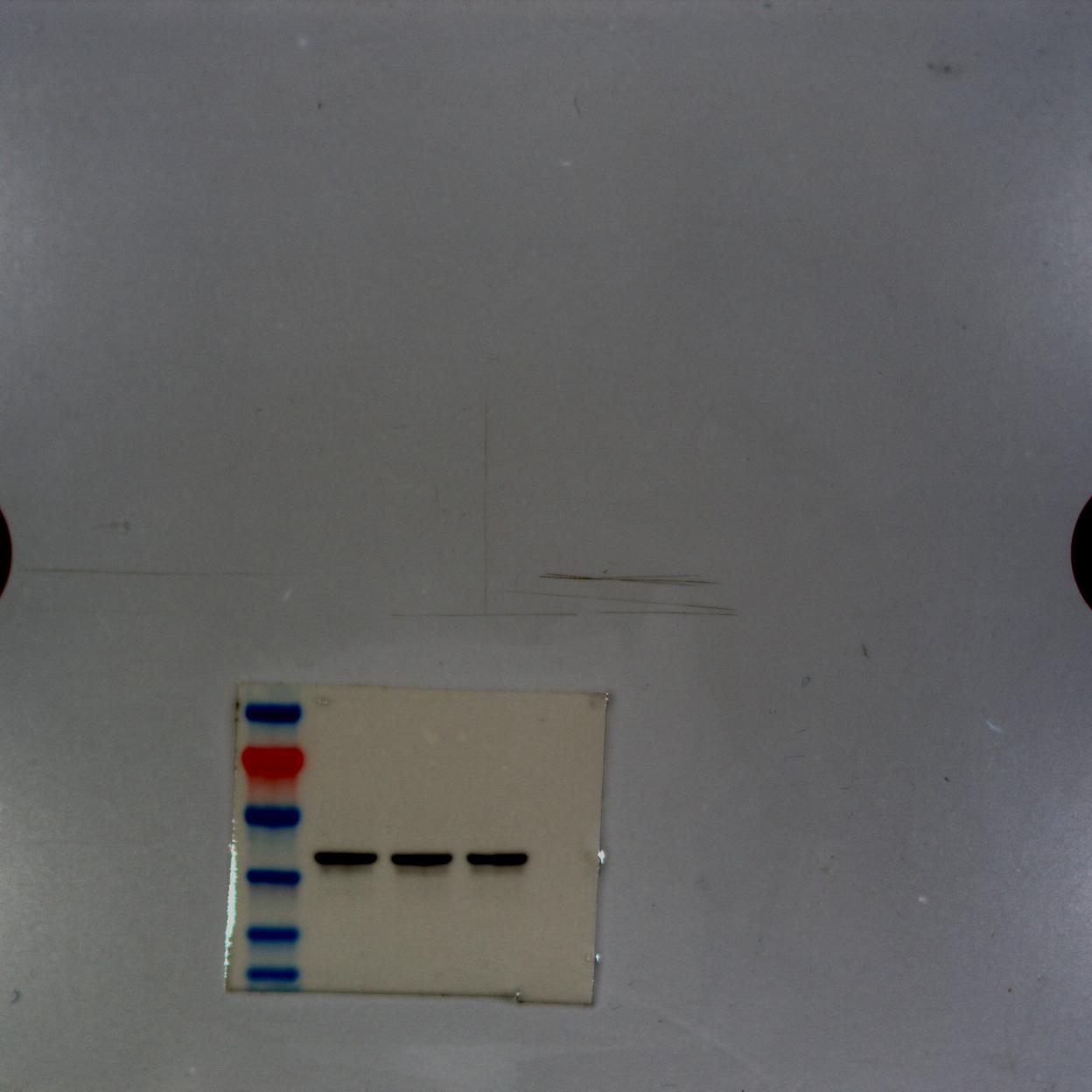

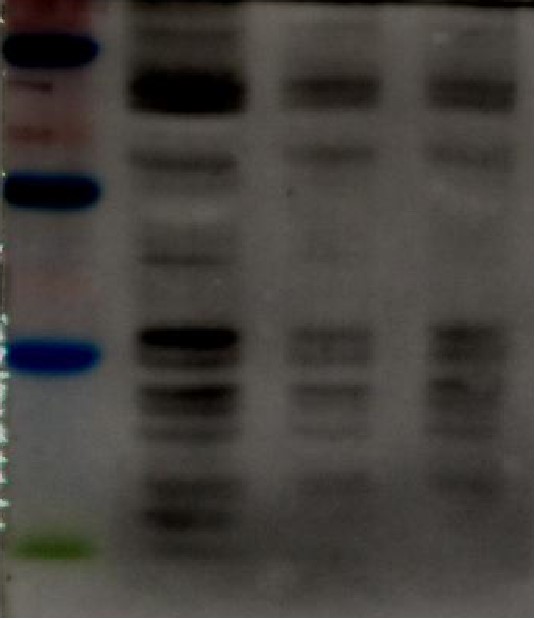

Supplement: Supplementary file 6 — Additional file 6. [file 12885_2023_11687_MOESM6_ESM.docx]
